# Chemoselective Boronic Ester Synthesis by Controlled Speciation**

**DOI:** 10.1002/anie.201406714

**Published:** 2014-09-29

**Authors:** James W B Fyfe, Ciaran P Seath, Allan J B Watson

**Affiliations:** WestCHEM, Department of Pure and Applied Chemistry, University of Strathclyde295 Cathedral Street, Glasgow, G1 1XL (UK)

**Keywords:** boron, chemoselectivity, cross-coupling, oligomerization, palladium

## Abstract

Control of boronic acid solution speciation is presented as a new strategy for the chemoselective synthesis of boronic esters. Manipulation of the solution equilibria within a cross-coupling milieu enables the formal homologation of aryl and alkenyl boronic acid pinacol esters. The generation of a new, reactive boronic ester in the presence of an active palladium catalyst also facilitates streamlined iterative catalytic C=C bond formation and provides a method for the controlled oligomerization of sp^2^-hybridized boronic esters.

Boronic acids and their associated derivatives are extensively used for C=C and C=X bond formation.[1,2] Many of these chemistries rely upon passive control of the solution speciation, that is, a catalyst chemoselectively engages one component of a larger mixture with the associated solution equilibria, enabling full conversion into the subsequent intermediate or product. An excellent exemplar of this is the Suzuki–Miyaura reaction which is contingent upon chemoselective engagement of a specific boron species by a transient palladium(II) intermediate.[3,4] Boron solution speciation can be complex and the deliberate and chemoselective control of the equilibria associated with a mixture of boron species has, to our knowledge, not been described.

Common methods for the preparation of sp^2^ boronic acid pinacol (BPin) esters generally rely upon the stoichiometric manipulation of a single boron species (Figure 1 a). Typical processes include treatment of a nucleophilic organometallic species with, for example, B(OEt)_3_ followed by hydrolysis and esterification with pinacol.[2,5] More contemporary processes use transition-metal catalysts with B_2_Pin_2_ or HBPin to furnish the same target compounds either by C=X cross-coupling or C=H activation processes.[6] In contrast, chemoselective control of a mixture of boron species is more challenging, requiring simultaneous manipulation of multiple equilibria. Accordingly, chemoselective synthesis of a boronic ester based on the control of speciation is a concept that would represent a fundamental advance in the field and provide new opportunities for iterative synthesis by facilitating access to high value components which may be further elaborated.

**Figure 1:**
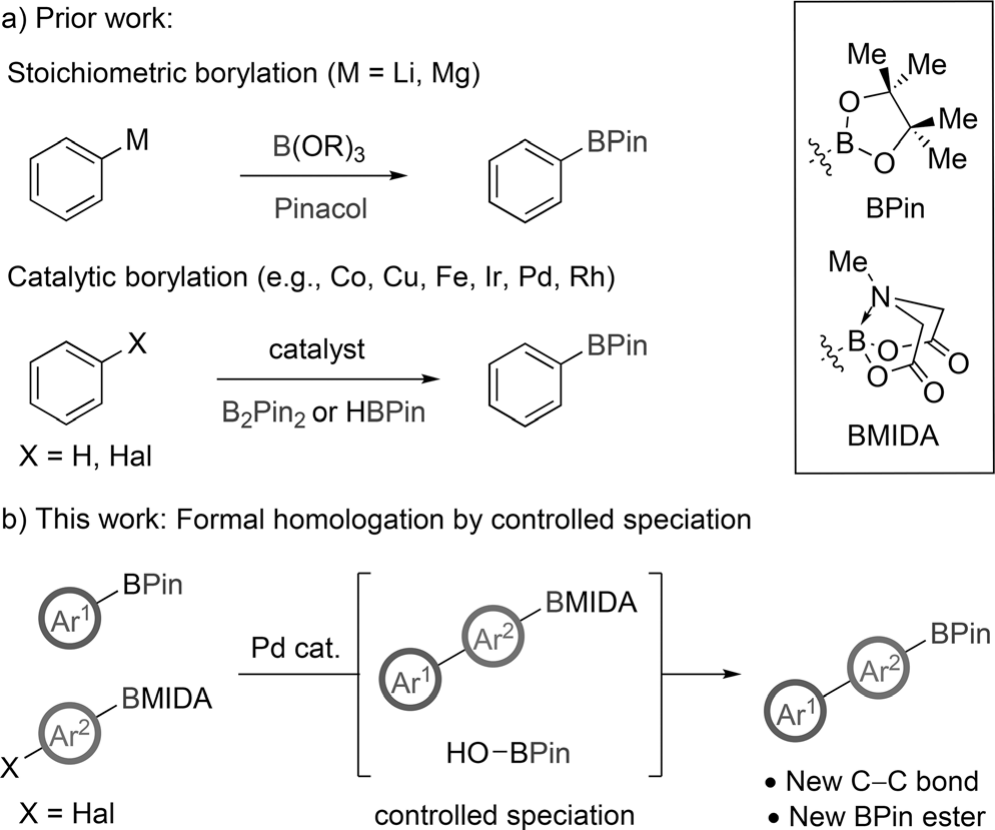
Strategy for chemoselective synthesis of BPin esters by controlled speciation. Hal=halogen, Pin=pinacolato, MIDA=*N*-methyliminodiacetic acid.

Herein we show that controlling the solution equilibria of boronic acid pinacol (BPin) and *N*-methyliminodiacetic acid (BMIDA) esters[7] during the course of a Suzuki–Miyaura cross-coupling event enables chemoselective formal homologation[8] of BPin esters (Figure 1 b). We also demonstrate the utility of this method to facilitate efficient iterative C=C bond formation[9] and to enable the controlled oligomerization of sp^2^-hybridized BPin esters.

We first examined the formal homologation reaction in a benchmark process with PhBPin (**1**), 4-bromophenylboronic acid MIDA ester (**2**), a conventional palladium catalyst ([PdCl_2_dppf]), using 10:1 THF/H_2_O (22 equiv H_2_O), and K_3_PO_4_ or Cs_2_CO_3_ (3 equiv) as the base (Scheme 1).[10,11] Pleasingly, we found that K_3_PO_4_ provided the desired product **3 a** in 30 % conversion with the mass balance consisting of oligomeric material (**6**): no BMIDA ester (**4**) or boronic acid (**5**) was observed. Cs_2_CO_3_ was slightly less effective (27 % conversion into **3 a**).

**Scheme 1:**
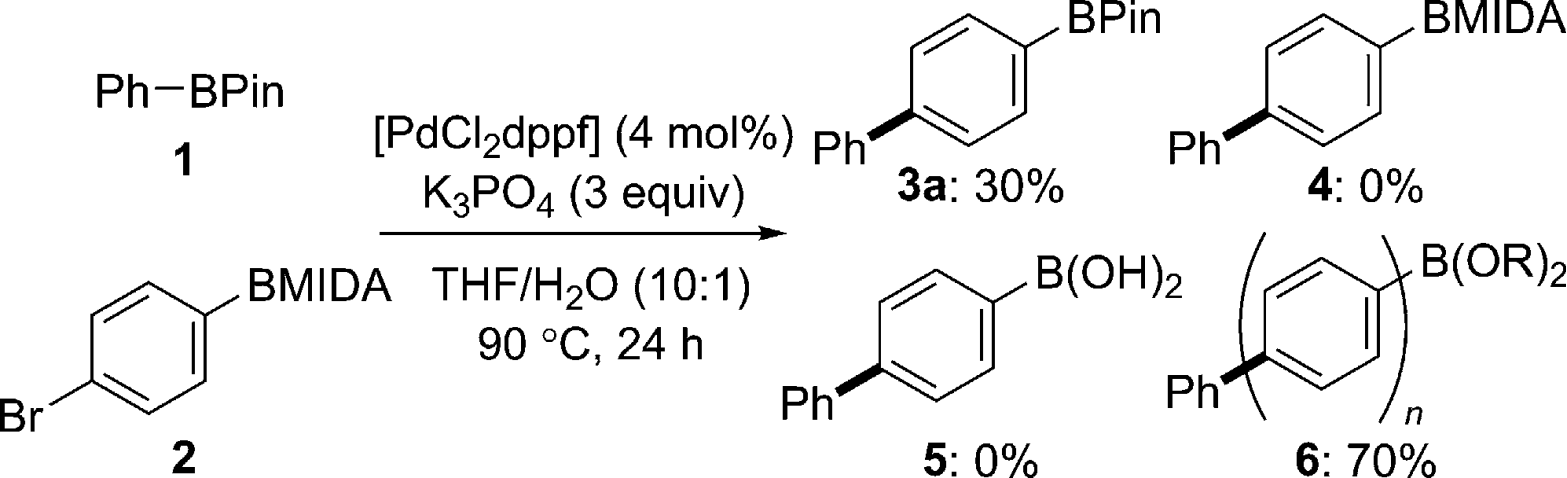
Initial studies of the formal homologation reaction.[11] dppf=1,1′-bis(diphenylphosphino)ferrocene, THF=tetrahydrofuran.

Based on these initial results, a survey of potassium bases demonstrated that K_3_PO_4_ was optimal, with those of higher and lower p*K*_aH_ providing significantly less control and leading to either poor cross-coupling or oligomeric products.[11] Similarly, variation of the phosphate countercation was not tolerated. As expected, the reaction efficiency was found to rely primarily on the stoichiometry of the base and H_2_O (Table 1).[11] An in depth analysis of H_2_O and K_3_PO_4_ stoichiometry revealed that both the cross-coupling and subsequent boron solution speciation could be effectively controlled using 5 equivalents H_2_O with 3 equivalents K_3_PO_4_ to provide 96 % conversion into the desired product (entry 6). Decreasing the quantity of K_3_PO_4_ (entries 2 and 3) and significantly increasing the quantity of H_2_O (entries 1 and 4) was not tolerated and led to increased oligomerization. Marginal changes to the H_2_O loading (entries 5 and 7) were tolerated but slightly less effective. Analysis of the reaction time course revealed that under the optimized reaction conditions the initial cross-coupling event was complete in approximately 1 hour with the remaining reaction time (approx. 23 h) required to channel the equilibria to the desired product. Similarly, heating to 90 °C was essential to drive the equilibria to the desired BPin product **3 a**: lower temperatures delivered mixtures of **3 a**, **4**, and **5**.[11]

**Table 1:** Optimization of the formal homologation reaction.[a]

Entry	Base	Base equiv	H_2_O equiv	3 aYield [%][b]
1	K_3_PO_4_	3	22	30
2	K_3_PO_4_	2	22	24
3	K_3_PO_4_	1	22	13
4	K_3_PO_4_	3	50	26
5	K_3_PO_4_	3	10	90
6	K_3_PO_4_	3	5	96
7	K_3_PO_4_	3	1	87

[a] All reactions run on a 0.25 mmol scale in 1 mL THF.[11]

[b] Determined by HPLC analysis using an internal standard.

Having identified the optimal reaction conditions for this speciation controlled chemoselective boronic ester synthesis, we next examined the scope of both the BPin and BMIDA coupling partners (Figure 2). A broad range of common functional groups was tolerated including ethers, esters, amides, nitriles, olefins, and heterocyclic residues (**3 a**–**r**). In addition, the reaction was also amenable to the synthesis of olefinic BPin adducts (**3 s**–**x**), which progress through the protodeboronation-prone vinyl boronic acid intermediates.[7u] For reactions where the initial cross-coupling was found to be slow, uncontrolled oligomerization was problematic. However, this was readily resolved through use of a more active catalyst system (Pd(OAc)_2_, SPhos; **3 c**, **3 j**, **3 o**, and **3 q**).

**Figure 2:**
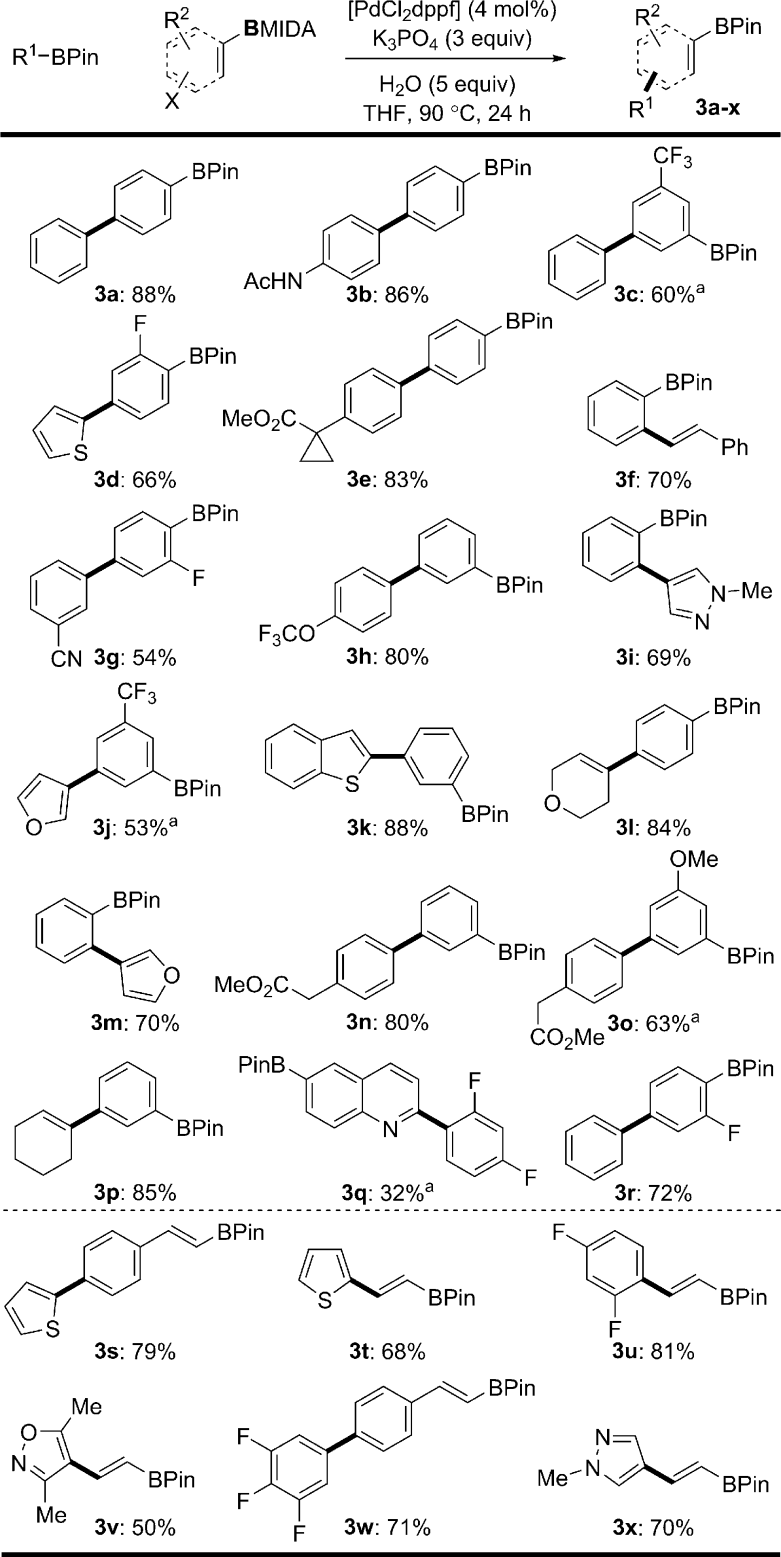
Scope of the formal homologation reaction with aryl boronic acid MIDA esters. All yields are those of the isolated products. X=Cl, Br, I. [a] Using Pd(OAc)_2_ (4 mol %) and SPhos (8 mol %).[11]

The ability to generate a new, reactive boronic ester in the presence of an active palladium catalyst offers opportunities for the development of a streamlined iterative catalytic bond formation. Current approaches to iterative bond formation using MIDA boronic esters have relied upon the cross-coupling of a conjunctive MIDA boronic ester, deprotection, and a subsequent cross-coupling.[9] In contrast, generation of a new BPin using our controlled speciation approach provides a more step-efficient process (Scheme 2). Following completion of the formal homologation reaction, addition of a second aryl bromide to the reaction vessel provides expedient access to triaryl adducts, such as the methyl ester of an LPA_1_ antagonist (**7 a**),[12] without the requirement for additional catalyst or additives and avoiding any intervening isolation, intermediate modification (e.g., deprotection), or purification steps.[13]

**Scheme 2:**
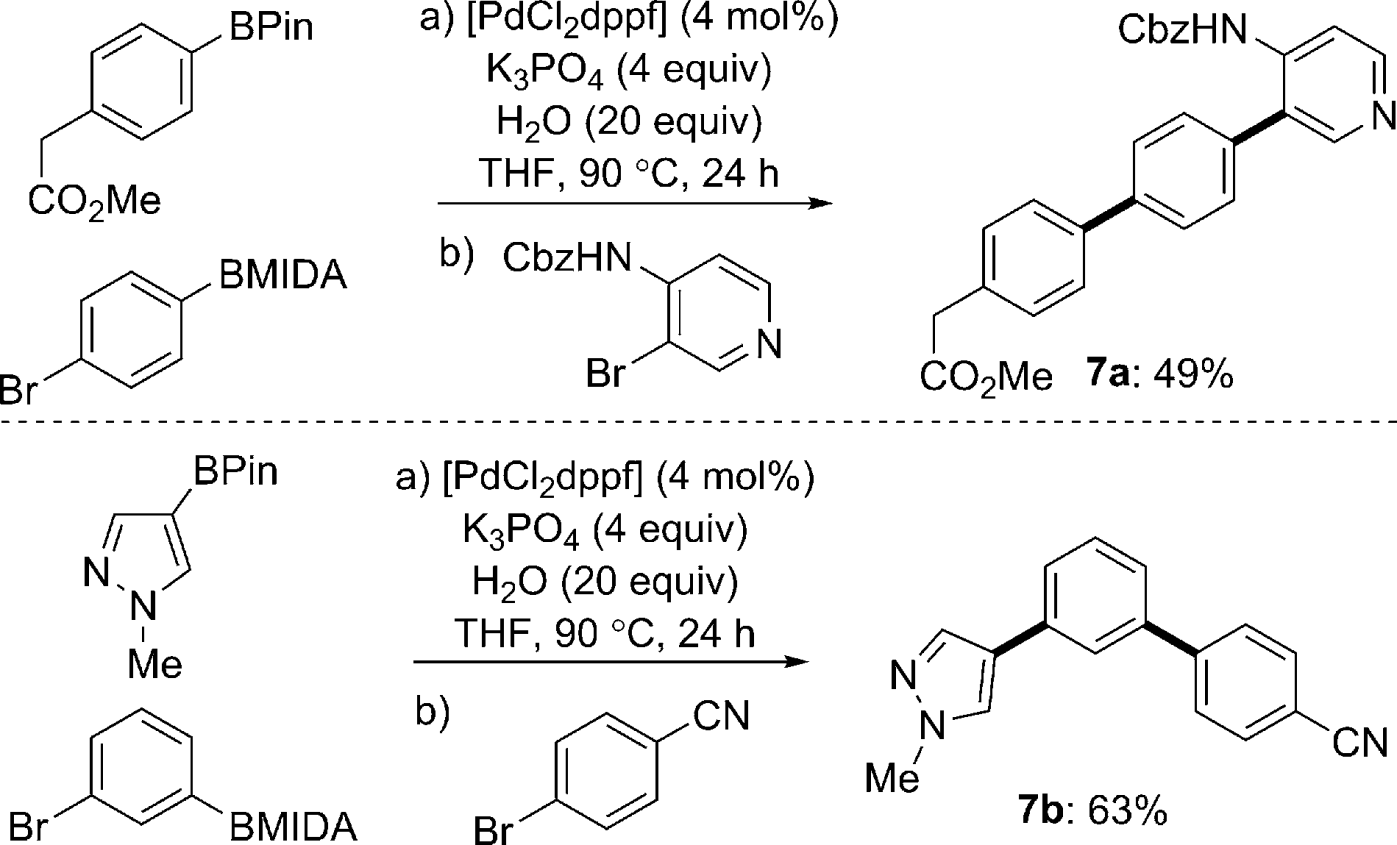
Streamlined iterative arylation enabled by controlled speciation.[11] Cbz=benzyloxycarbonyl.

To further challenge the applicability of our methodology, we sought to establish a method for controlled oligomerization (Scheme 3). Reaction of a BPin with two haloaryl BMIDA partners enables a double formal sp^2^ BPin homologation to provide the products **8** and **9**. In this process, two new C=C bonds are formed and pinacol is chemoselectively and stoichiometrically transferred over multiple boronic/boric ester species.

**Scheme 3:**
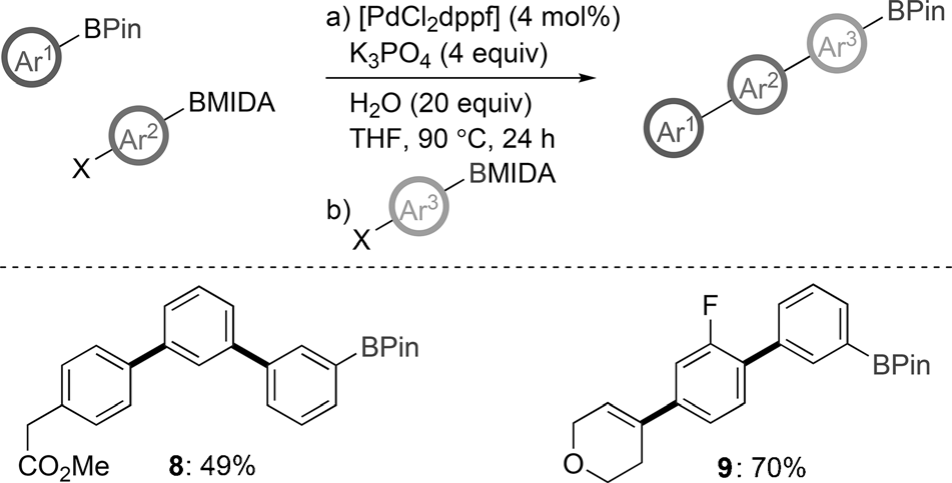
Controlled oligomerization by sequential formal homologation.[11]

Careful control of both the nature of the base and the stoichiometric relationship between base and H_2_O was crucial to the development of our reaction. MIDA boronic esters are base labile and require anhydrous cross-coupling conditions to avoid premature hydrolysis, which would lead to uncontrolled oligomerization. Accordingly, the base employed for this reaction was key and played three critical roles: 1) enabling suitably efficient cross-coupling, 2) sequestering H_2_O to ensure the integrity of the MIDA esters during cross-coupling, and 3) governing the speciation events.

The base profoundly impacts upon the transmetalation and reductive elimination events of the Suzuki–Miyaura reaction with the countercation being non-innocent in these processes.[4] In accordance with previous studies,[4] the rate of cross-coupling was found to be diminished when using bases weaker than PO_4_^3−^ (e.g., F_3_CCO_2_^−^, AcO^−^), with stronger bases (HO^−^, *t*BuO^−^) proving incompatible with the MIDA esters. The effect of counterions other than K^+^ was remarkable: harder cations (Li^+^, Na^+^) were detrimental and Cs^+^ also exhibited a negative effect.[4e, [11] The key to reconciling the stability of the BMIDA esters with the aqueous base required to control speciation downstream of the cross-coupling event was found through establishing an internal H_2_O reservoir by exploiting the hygroscopicity of the inorganic base and the associated aqueous biphase. Many common inorganic bases are hygroscopic and generate stable hydrates as well as saturated aqueous solutions of low relative humidity.[10] We have found that a suitable quantity of K_3_PO_4_, which forms a stable tetrahydrate,[10] possesses the ideal balance of hygroscopicity to sequester a controlled quantity of H_2_O to efficiently mitigate the hydrolysis of the MIDA esters and provide effective cross-coupling while simultaneously generating a basic biphase of sufficient pH to control the base-dependent boric acid and boronic acid equilibria.

In summary, we have shown that the solution speciation of boronic acids can be chemoselectively controlled to enable the formal homologation of boronic acid pinacol esters. The reaction is tolerant of aryl and vinyl functionality as both the pinacol donor and acceptor, respectively, and enables streamlined iterative cross-coupling as well as a method for controlled oligomerization. This study provides a conceptually new approach for the preparation of boronic acid derivatives to facilitate efficient iterative bond formation.
