# Supplementary material for: Chemoselective Boronic Ester Synthesis by Controlled Speciation
Source: Angew Chem Int Ed Engl. 2014 Sep 29;53(45):12077–80. doi: 10.1002/anie.201406714 (PMC4501314; doi:10.1002/anie.201406714)
Supplement: Supplementary file 1 [file anie0053-12077-sd1.pdf]

Angewandte Chemie
Eine Zeitschrift der Gesellschaft Deutscher Chemiker

Supporting Information
© Wiley-VCH 2014
69451 Weinheim, Germany

Chemoselective Boronic Ester Synthesis by Controlled Speciation**

*James W. B. Fyfe, Ciaran P. Seath, and Allan J. B. Watson**

anie_201406714_sm_miscellaneous_information.pdf

Contents

1. General

2. General experimental procedures

3. Reaction optimization data

3.1 Variation of the base

3.2 Variation of K_3PO_4 loading

3.3 Variation of H_2O loading

3.4 Variation of BPin:BMIDA stoichiometry

3.5 Variation of the Pd catalyst

3.6 Variation of catalyst loading

3.7 Variation of solvent

3.8 Variation of concentration

3.9 Variation of temperature

3.10 H_2O loading/time study

4. Compound characterization data

4.1 Intermediates

4.2 Products from Figure 2, Scheme 1, and Scheme 2

5. References

6. NMR and HRMS spectra for intermediates and products

1. General

All reagents and solvents were obtained from commercial suppliers and were used without further purification unless otherwise stated. Purification was carried out according to standard laboratory methods.[1]

1.1 Purification of Solvents

Dry solvents for reactions were either obtained from a PureSolv SPS-400-5 solvent purification system (THF, PhMe) or via distillation over a suitable drying agent following the prescribed methods (1,4-dioxane, EtOH, DCE, MeCN).[1] These solvents were transferred to and stored in a septum-sealed oven-dried flask over previously activated 4 Å molecular sieves and purged with and stored under nitrogen. CH_2Cl_2, Et_2O, EtOAc, MeCN, and petroleum ether 40-60° for purification purposes were used as obtained from suppliers without further purification.

1.2 Drying of Inorganic Bases

Inorganic bases were dried in a Heraeus Vacutherm oven at 60 °C under vacuum for a minimum of 24 hours before use.

1.3 Experimental Details

Reactions were carried out using conventional glassware (preparation of intermediates) or in capped 5 mL microwave vials (optimization reactions and reactions for Figure 2, Scheme 1, and Scheme 2). The glassware was oven-dried (140 °C) and purged with N_2 before use. Purging refers to a vacuum/nitrogen-refilling procedure. Room temperature was generally 18 °C. Reactions were carried out at elevated temperatures using a temperature-regulated hotplate/stirrer.

1.4 Purification of Products

Thin layer chromatography was carried out using Merck silica plates coated with fluorescent indicator UV254. These were analyzed under 254 nm UV light or developed using potassium permanganate solution. Normal phase flash chromatography was carried out using ZEOprep 60 HYD 40-63 µm silica gel. Reverse phase flash chromatography was carried out using IST Isolute C18 cartridges.

1.5 Analysis of Products

Fourier Transformed Infra-Red (FTIR) spectra were obtained on a Shimadzu IRAffinity-1 machine. ^{19}F NMR spectra were obtained on a Bruker AV 400 spectrometer at 376 MHz. ^{11}B NMR spectra were obtained on a Bruker AV 400 spectrometer at 128 MHz. ^{1}H and ^{13}C, NMR spectra were obtained on a Bruker AV 400 at 400 MHz and 125 MHz, respectively. Chemical shifts are reported in ppm and coupling constants are reported in Hz with $CDCl_3$ referenced at 7.26 (^{1}H) and 77.0 ppm (^{13}C) and DMSO-d_6 referenced at 2.50 (^{1}H) and 39.5 (^{13}C). In all ^{13}C spectra for boron containing compounds, the signal for the carbon bearing boron was not observed. High-resolution mass spectra were obtained through analysis at the EPSRC UK National Mass Spectrometry Facility at Swansea University.

2. General Procedures

General Procedure A: Optimized reaction (Figure 2)

For example, for the preparation of 2-([1,1'-biphenyl]-4-yl)-4,4,5,5-tetramethyl-1,3,2-dioxaborolane, **3a**

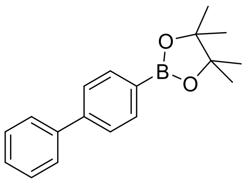

To an oven-dried 5 mL microwave vial was added 4-bromophenylboronic acid MIDA ester (78 mg, 0.25 mmol, 1 equiv), 4,4,5,5-tetramethyl-2-phenyl-1,3,2-dioxaborolane (76 mg, 0.375 mmol, 1.5 equiv), Pd(dppf)Cl_2·CH_2Cl_2 (8.2 mg, 0.01 mmol, 4 mol%), and K_3PO_4 (159 mg, 0.75 mmol, 3 equiv). The vial was then capped and purged with N_2 before addition of THF (1 mL, 0.25 M) and H_2O (22.5 µL, 1.25 mmol, 5 equiv). The reaction mixture was then heated to 90 °C for 24 h in a sand bath. The mixture was allowed to cool to room temperature and diluted with H_2O (4 mL) before loading directly onto a C18 column. The crude mixture was then purified by reverse phase chromatography (20-75% MeCN in H_2O). Fractions containing product were collected and the volatile organics were removed under vacuum. The resulting aqueous mixture was then extracted with EtOAc (2×100 mL). The combined organics were dried (Na_2SO_4) and concentrated under vacuum to give the desired product as an off-white solid (56 mg, 88%).

υ_{max} (film): 2978, 1396, 1359, 1143, 1091 cm^{-1}.

^{1}H NMR ($CDCl_3$, 400 MHz): δ 7.92 (d, *J* = 8.2 Hz, 2H), 7.63-7.66 (m, 4H), 7.45-7.49 (m, 2H), 7.38, (tt, *J* = 7.4, 1.2 Hz, 1H), 1.39 (s, 12H).

^{13}C NMR ($CDCl_3$, 126 MHz): δ 143.4, 140.6, 134.8, 128.3, 127.0, 126.7, 126.0, 83.3, 24.4.

^{11}B NMR ($CDCl_3$, 128 MHz): δ 31.3.

HRMS: exact mass calculated for $[M+H]^+$ ($C_{18}H_{22}BO_2$) requires *m/z* 281.1707, found *m/z* 281.1709.

General Procedure B: Optimized reaction (Figure 2)

For example, for the preparation of 2-(3-bromo-5-(trifluoromethyl)phenyl)-4,4,5,5-tetramethyl-1,3,2-dioxaborolane, **3c**

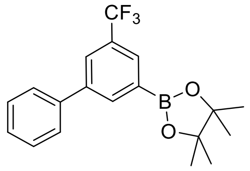

To an oven-dried 5 mL microwave vial was added 3-bromo-5-(trifluoromethyl)phenylboronic acid MIDA ester (88 mg, 0.25 mmol, 1 equiv), 4,4,5,5-tetramethyl-2-phenyl-1,3,2-dioxaborolane (76 mg, 0.375 mmol, 1.5 equiv), $Pd(OAc)_2$ (2.3 mg, 0.01 mmol, 4 mol%), SPhos (8.2 mg, 0.02 mmol, 8 mol%), and K_3PO_4 (159 mg, 0.75 mmol, 3 equiv). The vial was then capped and purged with N_2 before addition of THF (1 mL, 0.25 M) and H_2O (22.5 μL, 1.25 mmol, 5 equiv). The reaction mixture was then heated to 90 °C for 24 h. The mixture was allowed to cool to room temperature and diluted with H_2O (4 mL) before loading directly onto a C18 column. The crude mixture was then purified by reverse phase chromatography (20-75% MeCN in H_2O). Fractions containing product were collected and the volatile organics were removed under vacuum. The resulting aqueous mixture was then extracted with EtOAc (2×100 mL). The combined organics were dried (Na_2SO_4) and concentrated under vacuum to give the desired product as a yellow gum (53 mg, 60%).

υ_{max} (film): 2980, 2929, 1600, 1471 cm^{-1}.

^{1}H NMR ($CDCl_3$, 400 MHz): δ 8.22 (s, 1H), 8.06 (s, 1H), 7.93 (s, 1H), 7.62-7.69 (m, 2H), 7.46-7.53 (m, 2H), 7.42 (d, *J* = 7.3 Hz, 1H), 1.40 (s, 12H).

^{13}C NMR ($CDCl_3$, 126 MHz): δ 141.3, 139.7, 136.7, 130.1 (d, $^{3}J_{C-F}$ = 3.4 Hz), 128.9, 127.9, 127.3, 126.4 (d, $^{3}J_{C-F}$ = 3.4 Hz), 84.4, 24.9. CF_3 carbon and carbon bearing CF_3 not observed.

^{11}B NMR ($CDCl_3$, 128 MHz): δ 31.0.

^{19}F NMR ($CDCl_3$, 376 MHz): δ –62.4.

HRMS: exact mass calculated for $[M+H]^+$ ($C_{19}H_{21}BF_3O_2$) requires *m/z* 349.1581, found *m/z* 349.1581.

General Procedure C: One-pot iterative arylation/controlled oligomerization

For example, for the preparation of methyl 2-(4'-(4-(((benzyloxy)carbonyl)amino)pyridin-3-yl)-[1,1'-biphenyl]-4-yl)acetate, **7a**

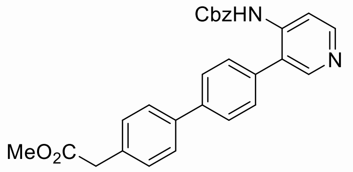

To an oven-dried 5 mL microwave vial was added 4-bromophenylboronic acid MIDA ester (78 mg, 0.25 mmol, 1 equiv), methyl 2-(4-(4,4,5,5-tetramethyl-1,3,2-dioxaborolan-2-yl)phenyl)acetate (104 mg, 0.375 mmol, 1.5 equiv), $Pd(dppf)Cl_2{\cdot}CH_2Cl_2$ (8.2 mg, 0.01 mmol, 4 mol%), and K_3PO_4 (212 mg, 1 mmol, 4 equiv). The vial was then capped and purged with N_2 before addition of THF (1 mL, 0.25 M) and H_2O (90 µL, 5 mmol, 20 equiv). The reaction mixture was then heated to 90 °C for 24 h. The reaction mixture was allowed to cool to room temperature before adding benzyl (3-bromopyridin-4-yl)carbamate (115 mg, 0.375 mmol, 1.5 equiv). The vial was then recapped and purged again with N_2 before being heated to 90 °C for a further 24 h. The reaction mixture was then cooled to room temperature before being quenched with H_2O (20 mL) and extracted with EtOAc (2×20 mL). The combined organics were collected, dried (Na_2SO_4), filtered, and concentrated under vacuum to a residue that was purified by reverse phase preparative HPLC (20-95% MeCN in H_2O) to afford the desired product as a white solid (55 mg, 49%).

υ_{max} (film): 1732, 1498, 1193, 1155, 1139, 1049 cm^{-1}.

^1H NMR ($CDCl_3$, 400 MHz): δ 8.85 (d, *J* = 6.8 Hz, 1H), 8.68 (d, *J* = 6.8 Hz, 1H), 8.64 (s, 1H), 7.81 (d, *J* = 8.1 Hz, 2H), 7.61 (d, *J* = 8.1 Hz, 2H), 7.54 (s, 1H), 7.39-7.46 (m, 8H), 5.26 (s, 2H), 3.75 (s, 3H), 3.72 (s, 2H).

^{13}C NMR ($CDCl_3$, 126 MHz): δ 171.9, 161.5, 161.1, 151.6, 150.2, 143.4, 141.5, 138.2, 134.3, 134.1, 130.1, 129.4, 129.2, 129.1, 129.0, 128.9, 128.4, 127.6, 127.4, 113.3,69.1, 52.2, 40.8.

HRMS: exact mass calculated for $[M+H]^+$ ($C_{28}H_{25}N_2O_4$) requires *m/z* 453.1809, found *m/z* 453.1804.

General Procedure D: Synthesis of MIDA esters from boronic acids

For example, for the preparation of 3-bromo-5-(trifluoromethyl)phenylboronic acid MIDA ester, **S1**

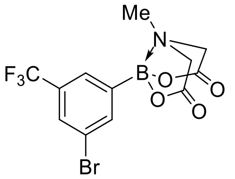

A mixture of 3-bromo-5-(trifluoromethyl)phenylboronic acid (2.0 g, 7.6 mmol, 1 equiv), *N*-methyliminodiacetic acid (1.17 g, 8 mmol, 1.05 equiv) in DMF (100 mL) was heated to 90 °C for 18 h under air. The reaction mixture was allowed to cool to room temperature and concentrated under vacuum to give an off-white slurry. EtOAc (100 mL) was added and the resulting precipitate was collected by filtration. The precipitate was washed with H_2O (2×50 mL) and Et_2O (2×50 mL) before being dried under vacuum to give the desired product as a white crystalline solid (1.63 g, 57%).

υ_{max} (film): 3344, 3014, 2978, 1760, 1323, 1286, 1201, 1159, 1103, 1035, 864 cm^{-1}.

^1H NMR (DMSO-d_6, 400 MHz): δ 7.96 (s, 1H), 7.92 (s, 1H), 7.79 (s, 1H), 4.38 (d, J = 17.2 Hz, 2H), 4.21 (d, J = 17.2 Hz, 2H), 2.62 (s, 3H).

^{13}C NMR (DMSO-d_6, 126 MHz): δ 169.2, 139.4, 130.4 (d, $^2J_{C\text{-}F}$ = 31.9 Hz), 128.3 (d, $^3J_{C\text{-}F}$ = 3.7 Hz), 128.0 (d, $^3J_{C\text{-}F}$ = 3.1 Hz), 123.4 (d, $^1J_{C\text{-}F}$ = 272.8 Hz), 122.2, 62.4, 48.0.

^{11}B NMR (DMSO-d_6, 128 MHz) δ 10.0.

^{19}F NMR (DMSO-d_6, 376 MHz): δ –61.0.

HRMS: exact mass calculated for $[M+H]^+$ ($C_{12}H_{11}BBrF_3NO_4$) requires *m/z* 379.9911, found *m/z* 379.9911.

General Procedure E: Miyaura borylation of aryl bromides

For example, for the preparation of methyl 2-(4-(4,4,5,5-tetramethyl-1,3,2-dioxaborolan-2-yl)phenyl)acetate, **S2**

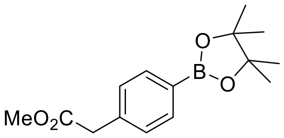

A mixture of methyl 2-(4-bromophenyl)acetate (3.0 g, 13.2 mmol, 1 equiv), bis(pinacolato)diboron (3.38 g, 13.3 mmol, 1.01 equiv), and KOAc (3.87 g, 39.5 mmol, 3 equiv) in 1,4-dioxane (65 mL), was degassed for 30 min by bubbling N_2 through the mixture. Pd(dppf)Cl_2·CH_2Cl_2 (323, 0.4 mmol, 3 mol%), was then added and the vessel was purged with N_2 before being heated to 100°C for 18 h. The reaction mixture was allowed to cool to room temperature and filtered through celite, washing with Et_2O. The filtrate was concentrated under vacuum to a residue that was dissolved in Et_2O (100 mL) and washed with H_2O (3×100 mL) and brine (4×50 mL). The organic extract was dried (Na_2SO_4), filtered, and concentrated under vacuum to a residue that was purified by column chromatography on silica (3-7% Et_2O in petroleum ether) to afford the desired product as an off-white solid (2.74 g, 75%).

υ_{max} (film): 2978, 1737, 1616, 1519, 1479 cm^{-1}.

^{1}H NMR ($CDCl_3$, 400 MHz): δ 7.80 (d, *J* = 7.8 Hz, 2 H), 7.32 (d, *J* = 7.8 Hz, 2 H), 3.71 (s, 3 H), 3.67 (s, 2 H), 1.37 (s, 12 H).

^{13}C NMR ($CDCl_3$, 126 MHz): δ 171.7, 137.1, 135.1, 128.6, 83.8, 52.0, 41.4, 24.8.

^{1}B NMR ($CDCl_3$, 128 MHz): δ 31.2.

HRMS: exact mass calculated for $[M+Na]^+$ (($C_{15}H_{21}BO_4Na$) requires *m/z* 299.1425, found *m/z* 299.1423.

General Procedure F: General procedure for optimization process

For example, for the preparation of 2-([1,1'-biphenyl]-4-yl)-4,4,5,5-tetramethyl-1,3,2-dioxaborolane, **3a**

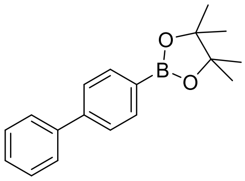

To an oven-dried 5 mL microwave vial was added 4-bromophenylboronic acid MIDA ester (78 mg, 0.25 mmol, 1 equiv), 4,4,5,5-tetramethyl-2-phenyl-1,3,2-dioxaborolane (76 mg, 0.375 mmol, 1.5 equiv), Pd(dppf)Cl_2·CH_2Cl_2 (8.2 mg, 0.01 mmol, 4 mol%), and K_3PO_4 (159 mg, 0.75 mmol, 3 equiv). The vial was then capped and purged with nitrogen before addition of THF/H_2O (10:1, 1 mL). The reaction mixture was then heated to 90 °C for 24 h. The reaction mixture was allowed to cool to room temperature before analysis by HPLC (30% conversion).

3. Reaction optimization data

3.1 Variation of the base

Reactions were carried out according to General Procedure F using 4-bromophenylboronic acid MIDA ester (78 mg, 0.25 mmol, 1 equiv), 4,4,5,5-tetramethyl-2-phenyl-1,3,2-dioxaborolane (76 mg, 0.375 mmol, 1.5 equiv), Pd(dppf)Cl_2·CH_2Cl_2 (8.2 mg, 0.01 mmol, 4 mol%), **Base** (0.75 mmol, 3 equiv), and THF/H_2O (10:1, 1 mL).

Entry	Base (mass)	Conversion
**1**	K_3PO_4 (159 mg)	30%
**2**	Cs_2CO_3 (244 mg)	27%
**3**	KOH (42 mg)	21%
**4**	KOAc (74 mg)	19%
**5**	KO*t*-Bu (84mg)	21%
**6**	K_2CO_3 (104 mg)	12%
**7**	KO_2CCF_3 (114 mg)	-
**8**	KH_2PO_4 (102 mg)	-
**9**	K_2HPO_4 (131 mg)	11%
**10**	Li_3PO_4 (87 mg)	-
**11**	Na_3PO_4 (123 mg)	16%
**12**	Cs_3PO_4 (370 mg)	8%

**13**	$Mg_3(PO_4)_2$ (305 mg)	-
**14**	$Ca_3(PO_4)_2$ (233mg)	-

3.2 Variation of K_3PO_4 loading

Reactions were carried out according to General Procedure F using 4-bromophenylboronic acid MIDA ester (78 mg, 0.25 mmol, 1 equiv), 4,4,5,5-tetramethyl-2-phenyl-1,3,2-dioxaborolane (76 mg, 0.375 mmol, 1.5 equiv), Pd(dppf)Cl_2·CH_2Cl_2 (8.2 mg, 0.01 mmol, 4 mol%), and K_3PO_4 (**X** equiv), and THF/H_2O (10:1, 1 mL).

Entry	K_3PO_4 equiv (mass)	Conversion
**1**	1 (53 mg)	24%
**2**	2 (106 mg)	13%
**3**	3 (159 mg)	30%

3.3 Variation of H_2O loading

Reactions were carried out according to General Procedure F using 4-bromophenylboronic acid MIDA ester (71 mg, 0.226 mmol, 1 equiv), 4,4,5,5-tetramethyl-2-phenyl-1,3,2-dioxaborolane (69 mg, 0.339 mmol, 1.5 equiv), Pd(dppf)Cl_2·CH_2Cl_2 (7.4 mg, 0.01 mmol, 4 mol%), K_3PO_4 (144 mg, 0.678 mmol, 3 equiv), THF (0.9 mL, 0.25 M), and H_2O (**X** equiv).

Entry	H_2O equiv (volume)	Conversion
**1**	0 (0 mL)	61%
**2**	1 (4.1 µL)	87%
**3**	3 (12.2 µL)	91%
**4**	5 (20.4 µL)	96%
**5**	10 (40.7 µL)	90%
**6**	15 (61.1 µL)	87%
**7**	20 (81.4 µL)	32%
**8**	25 (0.102 mL)	25%
**9**	30 (0.122 mL)	34%
**10**	50 (0.203 mL)	26%
**11**	75 (0.305 mL)	18%

**12**	100 (0.407 mL)	19%

3.4 Variation of BPin:BMIDA stoichiometry

Reactions were carried out according to General Procedure F using 4-bromophenylboronic acid MIDA ester (71 mg, 0.226 mmol, 1 equiv), 4,4,5,5-tetramethyl-2-phenyl-1,3,2-dioxaborolane (**X** equiv), Pd(dppf)Cl_2·CH_2Cl_2 (7.4 mg, 0.01 mmol, 4 mol%), K_3PO_4 (144 mg, 0.678 mmol, 3 equiv), THF (0.9 mL, 0.25 M), and H_2O (20.4 µL, 1.13 mmol, 5 equiv).

Entry	BPin Equiv (mass)	Conversion
**1**	1 (46 mg)	74%
**2**	1.1 (51 mg)	77%
**3**	1.2 (55 mg)	78%
**4**	1.3 (60 mg)	78%
**5**	1.4 (64 mg)	78%
**6**	1.5 (69 mg)	96%

3.5 Variation of the Pd catalyst

Reactions were carried out according to General Procedure F using 4-bromophenylboronic acid MIDA ester (71 mg, 0.226 mmol, 1 equiv), 4,4,5,5-tetramethyl-2-phenyl-1,3,2-dioxaborolane (69 mg, 0.339 mmol, 1.5 equiv), **Catalyst** (0.01 mmol, 4 mol%), K_3PO_4 (144 mg, 0.678 mmol, 3 equiv), THF (0.9 mL, 0.25 M), and H_2O (20.4 µL, 1.13 mmol, 5 equiv).

Entry	Catalyst (mass)	Conversion
**1**	Pd(dppf)Cl_2·CH_2Cl_2 (7.4 mg)	96%
**2**	Pd(PPh_3)$_4$ (10.4 mg)	36%
**3**	Pd(OAc)$_2$ (2 mg)	5%
**4**	Pd_2(dba)$_3$ (8.3 mg)	7%
**5**	Pd(PPh_3)$_2Cl_2$ (6.4 mg)	63%

3.6 Variation of the catalyst loading

Reactions were carried out according to General Procedure F using 4-bromophenylboronic acid MIDA ester (71 mg, 0.226 mmol, 1 equiv), 4,4,5,5-tetramethyl-2-phenyl-1,3,2-dioxaborolane (69 mg, 0.339 mmol, 1.5 equiv), Pd(dppf)Cl_2·CH_2Cl_2 (**X** mol%), K_3PO_4 (144 mg, 0.678 mmol, 3 equiv), THF (0.9 mL, 0.25 M), and H_2O (20.4 µL, 1.13 mmol, 5 equiv).

Entry	Catalyst Loading (mass)	Conversion
**1**	1 mol% (1.9 mg)	67%
**2**	2 mol% (3.7 mg)	84%

3.7 Variation of solvent

Reactions were carried out according to General procedure F using 4-bromophenylboronic acid MIDA ester (71 mg, 0.226 mmol, 1 equiv), 4,4,5,5-tetramethyl-2-phenyl-1,3,2-dioxaborolane (69 mg, 0.339 mmol, 1.5 equiv), Pd(dppf)Cl_2·CH_2Cl_2 (7.4 mg, 0.01 mmol, 4 mol%), K_3PO_4 (144 mg, 0.678 mmol, 3 equiv), **Solvent** (0.9 mL, 0.25 M), and H_2O (20.4 µL, 1.13 mmol, 5 equiv).

Entry	Solvent	Conversion
**1**	THF	96%
**2**	MeCN	70%
**3**	1,4-Dioxane	76%
**4**	PhMe	10%
**5**	DCE	60%
**6**	EtOH	25%

3.8 Variation of concentration

Reactions were carried out according to General Procedure F using 4-bromophenylboronic acid MIDA ester (71 mg, 0.226 mmol, 1 equiv), 4,4,5,5-tetramethyl-2-phenyl-1,3,2-dioxaborolane (69 mg, 0.339 mmol, 1.5 equiv), Pd(dppf)Cl_2·CH_2Cl_2 (7.4 mg, 0.01 mmol, 4 mol%), K_3PO_4 (144 mg, 0.678 mmol, 3 equiv), THF (**X** M), and H_2O (20.4 µL, 1.13 mmol, 5 equiv).

Entry	Concentration (Volume)	Conversion
**1**	0.125 M (1.8 mL)	86%
**2**	0.25 M (0.9 mL)	96%
**3**	0.5 M (0.45 mL)	89%

3.9 Variation of temperature

Reactions were carried out according to General Procedure F using 4-bromophenylboronic acid MIDA ester (71 mg, 0.226 mmol, 1 equiv), 4,4,5,5-tetramethyl-2-phenyl-1,3,2-dioxaborolane (69 mg, 0.339 mmol, 1.5 equiv), Pd(dppf)Cl_2·CH_2Cl_2 (7.4 mg, 0.01 mmol, 4 mol%), K_3PO_4 (144 mg, 0.678 mmol, 3 equiv), THF (0.9 mL**,** 0.25 M), and H_2O (20.4 µL, 1.13 mmol, 5 equiv).

Entry	Temperature	Conversion
**1**	RT	-
**2**	40 °C	17%
**3**	50 °C	45%
**4**	60 °C	75%
**5**	70 °C	78%
**6**	80 °C	83%
**7**	90 °C	96%
**8**	100 °C	75%

3.10 H_2O loading/time study

Reactions were carried out according to General Procedure F using 4-bromophenylboronic acid MIDA ester (71 mg, 0.226 mmol, 1 equiv), 4,4,5,5-tetramethyl-2-phenyl-1,3,2-dioxaborolane (69 mg, 0.339 mmol, 1.5 equiv), Pd(dppf)Cl_2·CH_2Cl_2 (7.4 mg, 0.01 mmol, 4 mol%), K_3PO_4 (144 mg, 0.678 mmol, 3 equiv), THF (0.9 mL, 0.25 M), and H_2O (**X** equiv) for **X** h.

0 equiv H_2O (0 mL)

Entry	Time (h)	Conversion
**1**	1	42%
**2**	2	44%
**3**	4	45%
**4**	6	60%
**5**	12	63%
**6**	18	67%
**7**	24	61%

1 equiv H_2O (4.1 µL)

Entry	Time (h)	Conversion
**1**	1	25%
**2**	2	50%
**3**	4	52%
**4**	6	68%
**5**	12	77%
**6**	18	77%
**7**	24	87%

3 equiv H_2O (12.2 µL)

Entry	Time (h)	Conversion
**1**	1	42%
**2**	2	48%
**3**	4	61%
**4**	6	76%
**5**	12	87%
**6**	18	78%
**7**	24	91%

5 equiv H_2O (20.4 µL)

Entry	Time (h)	Conversion
**1**	1	47%
**2**	2	53%
**3**	4	76%
**4**	6	78%
**5**	12	87%
**6**	18	82%
**7**	24	96%

10 equiv H_2O (40.7 µL)

Entry	Time (h)	Conversion
**1**	1	36%
**2**	2	56%
**3**	4	61%
**4**	6	75%
**5**	12	82%
**6**	18	80%
**7**	24	90%

15 equiv H_2O (61.1 µL)

Entry	Time (h)	Conversion
**1**	1	36%
**2**	2	67%
**3**	4	70%
**4**	6	81%
**5**	12	49%
**6**	18	61%
**7**	24	87%

20 equiv H_2O (81.4 µL)

**Entry**	**Time (h)**	**Conversion**
**1**	1	42%
**2**	2	71%
**3**	4	75%
**4**	6	71%
**5**	12	54%
**6**	18	48%
**7**	24	32%

25 equiv H_2O (0.102 mL)

**Entry**	**Time (h)**	**Conversion**
**1**	1	40%
**2**	2	56%
**3**	4	62%
**4**	6	52%
**5**	12	36%
**6**	18	44%
**7**	24	25%

30 equiv H_2O (0.122 mL)

**Entry**	**Time (h)**	**Conversion**
**1**	1	20%
**2**	2	31%
**3**	4	39%
**4**	6	33%
**5**	12	42%
**6**	18	40%
**7**	24	34%

50 equiv H_2O (0.203 mL)

Entry	Time (h)	Conversion
**1**	1	26%
**2**	2	29%
**3**	4	27%
**4**	6	22%
**5**	12	26%
**6**	18	29%
**7**	24	26%

75 equiv H_2O (0.305 mL)

Entry	Time (h)	Conversion
**1**	1	15%
**2**	2	23%
**3**	4	19%
**4**	6	19%
**5**	12	25%
**6**	18	20%
**7**	24	18%

100 equiv H_2O (0.407 mL)

Entry	Time (h)	Conversion
**1**	1	23%
**2**	2	22%
**3**	4	21%
**4**	6	23%
**5**	12	26%
**6**	18	24%
**7**	24	19%

4. Compound Characterization Data

4.1 Intermediates

3-Bromo-5-(trifluoromethyl)phenylboronic acid MIDA ester, **S1**

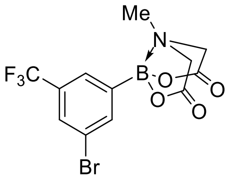

Prepared according to General Procedure D using 3-bromo-5-(trifluoromethyl)phenylboronic acid (2.0 g, 7.6 mmol, 1 equiv), *N*-methyliminodiacetic acid (1.17 g, 8 mmol, 1.05 equiv) in DMF (100 mL) to afford the desired product as a white crystalline solid (1.63 g, 57%).

υ_{max} (film): 3344, 3014, 2978, 1760, 1323, 1286, 1201, 1159, 1103, 1035, 864 cm^{-1}.

^{1}H NMR (DMSO-d_6, 400 MHz): δ 7.96 (s, 1H), 7.92 (s, 1H), 7.79 (s, 1H), 4.38 (d, J = 17.2 Hz, 2H), 4.21 (d, J = 17.2 Hz, 2H), 2.62 (s, 3H).

^{13}C NMR (DMSO-d_6, 126 MHz): δ 169.2, 139.4, 130.4 (d, $^{2}J_{C-F}$ = 31.9 Hz), 128.3 (d, $^{3}J_{C-F}$ = 3.7 Hz), 128.0 (d, $^{3}J_{C-F}$ = 3.1 Hz), 123.4 (d, $^{1}J_{C-F}$ = 272.8 Hz), 122.2, 62.4, 48.0.

^{11}B NMR (DMSO-d_6, 128 MHz) δ 10.0.

^{19}F NMR (DMSO-d_6, 376 MHz): δ –61.0.

HRMS: exact mass calculated for $[M+H]^+$ ($C_{12}H_{11}BBrF_3NO_4$) requires *m/z* 379.9911, found *m/z* 379.9911.

Methyl 2-(4-(4,4,5,5-tetramethyl-1,3,2-dioxaborolan-2-yl)phenyl)acetate, **S2**

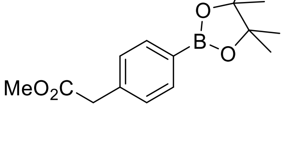

Prepared according to General Procedure E using methyl 2-(4-bromophenyl)acetate (3.0 g, 13.2 mmol, 1 equiv), bis(pinacolato)diboron (3.38 g, 13.3 mmol, 1.01 equiv), and KOAc (3.87 g, 39.5 mmol, 3 equiv) in 1,4-dioxane (65 mL). After 18 h, the reaction mixture was

subjected to the purification outlined in the General Procedure (silica gel, 3-7% Et_2O in petroleum ether) to afford the desired product as an off-white solid (2.74 g, 75%).

υ_{max} (film): 2978, 1737, 1616, 1519, 1479 cm^{-1}.

^{1}H NMR ($CDCl_3$, 400 MHz): δ 7.80 (d, *J* = 7.8 Hz, 2 H), 7.32 (d, *J* = 7.8 Hz, 2 H), 3.71 (s, 3 H), 3.67 (s, 2 H), 1.37 (s, 12 H).

^{13}C NMR ($CDCl_3$, 126 MHz): δ 171.7, 137.1, 135.1, 128.6, 83.8, 52.0, 41.4, 24.8.

^{1}B NMR ($CDCl_3$, 128 MHz): δ 31.2.

HRMS: exact mass calculated for $[M+Na]^+$ (($C_{15}H_{21}BO_4Na$) requires *m/z* 299.1425, found *m/z* 299.1423.

4-Bromo-2-fluorophenylboronic acid MIDA ester, **S3**

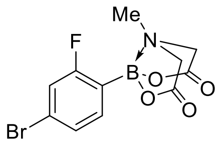

Prepared according to General Procedure D using 4-bromo-2-fluorophenylboronic acid (875 mg, 4 mmol, 1 equiv), *N*-methyliminodiacetic acid (618 mg, 4.2 mmol, 1.05 equiv), and DMF (50 mL) to afford the desired product as a white crystalline solid (994 mg, 75%).

υ_{max} (film): 3014, 2978, 1761, 1575, 1340, 1292, 1255, 1193, 1033, 999, 871, 815 cm^{-1}.

^{1}H NMR (DMSO-d_6, 400 MHz): δ 7.40-7.48 (m, 3H), 4.42 (d, *J* = 17.3 Hz, 2H), 4.10 (d, *J* = 17.3 Hz, 2H), 2.63 (s, 3H).

^{13}C NMR (DMSO-d_6, 126 MHz): δ 168.8, 165.3 (d, $^1J_{C\text{-}F}$ = 246.1), 136.4 (d, $^3J_{C\text{-}F}$ = 10.0 Hz), 127.4, 123.3 (d, $^3J_{C\text{-}F}$ = 10.0 Hz), 118.3 (d, $^2J_{C\text{-}F}$ = 28.8 Hz), 62.4, 47.5.

^{11}B NMR (DMSO-d_6, 128 MHz): δ 10.7.

^{19}F NMR (DMSO-d_6, 376 MHz): δ –102.9.

HRMS: exact mass calculated for $[M+H]^+$ ($C_{11}H_{11}BBrFNO_4$) requires *m/z* 329.9943, found *m/z* 329.9944.

3-Chloro-5-methoxyphenylboronic acid MIDA ester, **S4**

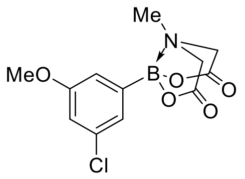

Prepared according to General Procedure D using 3-chloro-5-methoxyphenylboronic acid (1.82 g, 9.8 mmol, 1 equiv), *N*-methyliminodiacetic acid (1.58 g, 10.7 mmol, 1.05 equiv), and DMF (50 mL) to afford the desired product as a white crystalline solid (2.8 g, 96%).

υ_{max} (film): 1747, 1332, 1292, 1271, 1238, 1193, 1178, 1031, 1002, 835 cm^{-1}.

^{1}H NMR (DMSO-d$_6$, 400 MHz): δ 6.98-7.02 (m, 2H), 6.89-6.93 (m, 1H), 4.33 (d, *J* = 17.2 Hz, 2H), 4.14 (d, *J* = 17.2 Hz, 2H), 3.78 (s, 3H), 2.56 (s, 3H).

^{13}C NMR (DMSO-d$_6$, 126 MHz): δ 169.2, 159.8, 133.5, 124.1, 116.9, 114.2, 62.0, 55.4, 47.6.

^{11}B NMR (DMSO-d$_6$, 128 MHz): δ 11.0.

HRMS: exact mass calculated for $[M+H]^+$ ($C_{12}H_{14}BClNO_5$) requires *m/z* 298.0648, found *m/z*298.0651.

(2-Chloroquinolin-6-yl)boronic acid MIDA ester, **S5**

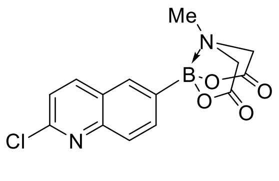

Prepared according to General Procedure D using (2-chloroquinolin-6-yl)boronic acid (300 mg, 1.45 mmol, 1 equiv), *N*-methyliminodiacetic acid (234 mg, 1.6 mmol, 1.05 equiv), and DMF (10 mL) to afford the desired product as a white solid (420 mg, 91%).

υ_{max} (film): 1768, 1743, 1446, 1178, 1141, 1029, 1001, 835 cm^{-1}.

^{1}H NMR (DMSO-d$_6$, 400 MHz): δ 8.49 (d, *J* = 8.5 Hz, 1H), 8.13 (s, 1H), 7.94 (d, *J* = 8.5 Hz, 1H), 7.88 (dd, *J* = 8.5, 1.2 Hz, 1H), 7.59 (d, *J* = 8.5 Hz, 1H), 4.41 (d, *J* = 17.3 Hz, 2H), 4.20 (d, *J* = 17.3 Hz, 2H), 2.55 (s, 3H).

^{13}C NMR (DMSO-d$_6$, 126 MHz): δ 169.3, 150.0, 147.7, 140.3, 134.5, 132.9, 126.7, 126.2, 122.3, 62.0, 47.7.

^{11}B NMR (DMSO-d$_6$, 128 MHz): δ 10.7.

HRMS: exact mass calculated for $[M+H]^+$ ($C_{14}H_{13}BClN_2O_4$) requires *m/z* 319.0651, found *m/z* 319.0652.

Methyl 1-(4-(4,4,5,5-tetramethyl-1,3,2-dioxaborolan-2-yl)phenyl)cyclopropane-1-carboxylate, **S6**

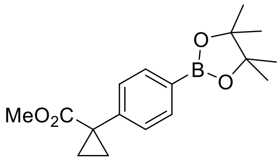

Prepared according to General Procedure E using methyl 1-(4-bromophenyl)cyclopropanecarboxylate (2.50 g, 9.8 mmol, 1 equiv), bis(pinacolato)diboron (2.51 g, 9.9 mmol, 1.01 equiv), KOAc (2.88 g, 29.4 mmol, 3 equiv), Pd(dppf)Cl_2·CH_2Cl_2 (240 mg, 0.29 mmol, 0.03 equiv), and 1,4-dioxane (49 mL, 0.2 M). After 18 h, the reaction mixture was subjected to the purification outlined in the General Procedure (silica gel, 3-8% Et_2O in petroleum ether) to afford the desired product as a white solid (1.2 g, 40%).

υ_{max} (film): 2978, 1708, 1614, 1372, 1298, 1168, 1101, 858 cm^{-1}.

^{1}H NMR (CD_3CN, 400 MHz): δ 7.79 (d, *J* = 8.1 Hz, 2H), 7.38 (d, *J* = 8.1 Hz, 2H), 3.64 (s, 3H), 1.63 (q, *J* = 4.0 Hz, 2H), 1.36 (s, 12H), 1.22 (q, *J* = 4.0 Hz, 2H).

^{13}C NMR (CD_3CN, 126 MHz): δ 174.4, 142.1, 134.2, 129.4, 83.3, 51.9, 28.6, 24.3, 16.2.

^{11}B NMR ($CDCl_3$, 128 MHz): δ 31.0.

HRMS: exact mass calculated for $[M+H]^+$ ($C_{17}H_{24}BO_4$) requires *m/z* 303.1762, found *m/z* 313.1767.

2-(Benzo[b]thiophen-2-yl)-4,4,5,5-tetramethyl-1,3,2-dioxaborolane, **S7**

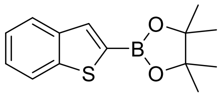

A mixture of benzo[b]thiophene-2-boronic acid (1.25 g, 7 mmol, 1 equiv), pinacol (830 mg, 7 mmol, 1 equiv), and trifluoroacetic acid (53.7 µL, 0.7 mmol, 0.1 equiv) in Et_2O (35 mL, 0.2 M) was stirred at room temperature for 2 h under N_2. The mixture was then concentrated under vacuum to give a residue that was diluted with hexane (30 mL), filtered, and concentrated under vacuum to afford the desired product as a beige solid (1.79 g, 98%).

υ_{max} (film): 2978, 1526, 1348, 1338, 1137 cm^{-1}.

^1H NMR (CD_3CN, 400 MHz): δ 7.91-7.93 (m, 2H), 7.86-7.88 (m, 1H), 7.35-7.40 (m, 2H), 1.40 (s, 12H).

^{13}C NMR (CD_3CN, 126 MHz): δ 143.8, 140.5, 134.5, 125.3, 124.4, 124.1, 122.5, 84.5, 24.8.

^{11}B NMR ($CDCl_3$, 128 MHz): δ 29.1.

HRMS: exact mass calculated for $[M+H]^+$ ($C_{14}H_{18}BO_2S$) requires *m/z* 261.1115, found *m/z* 261.1115.

Benzyl (3-bromopyridin-4-yl)carbamate, **S8**

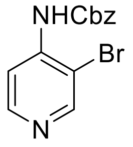

4-Amino-3-bromopyrdine (3.0 g, 17.3 mmol, 1 equiv) taken up in dry CH_2Cl_2 (90 mL), purged with N_2 and cooled to 0 °C. Triethylamine (6.05 mL, 43.3 mmol, 2.5 equiv) was then added followed by the dropwise addition of benzyl chloroformate (2.7 mL, 19.1 mmol, 1.1 equiv). The reaction was stirred for 30 minutes before being allowed to warm to room temperature and was stirred for a further 16 h. The reaction mixture was quenched with aqueous sodium bicarbonate (20 mL) and then diluted with CH_2Cl_2 (100 mL). The solution was washed with sat. aq. $NaHCO_3$ solution (200 mL). The aqueous extract was re-extracted with CH_2Cl_2 (100 mL). The combined organics were dried over Na_2SO_4, filtered, and concentrated to a residue that was dry-loaded onto silica and purified by flash chromatography (10-30% EtOAc in petroleum ether) to afford the desired compound as a white solid. (5.1 g, 80%).

υ_{max} (solid): 3391, 3262, 1742, 1662, 1582, 1506 cm^{-1}.

^{1}H NMR ($CDCl_3$, 400 MHz): δ 8.62 (s, 1 H), 8.43 (d, J = 5.5 Hz, 1 H), 8.20 (d, J = 5.5 Hz, 1 H), 7.39-7.47 (m, 6 H), 5.27 (s, 2 H).

^{13}C NMR ($CDCl_3$, 126 MHz): δ 151.8, 151.2, 149.1, 142.2, 134.6, 128.3, 128.2, 128.1, 112.8, 109.3, 67.5.

HRMS: exact mass calculated for $[M+H]^+$ ($C_{13}H_{12}BrN_2O_2$) requires *m/z* 307.0077, found *m/z* 307.0077.

4.2 Products from Figure 2, Scheme 1, and Scheme 2

2-([1,1'-Biphenyl]-4-yl)-4,4,5,5-tetramethyl-1,3,2-dioxaborolane, **3a**

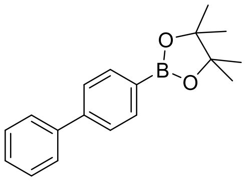

Prepared according to General Procedure A using 4-bromophenylboronic acid MIDA ester (78 mg, 0.25 mmol, 1 equiv), 4,4,5,5-tetramethyl-2-phenyl-1,3,2-dioxaborolane (76 mg, 0.375 mmol, 1.5 equiv), $Pd(dppf)Cl_2{\cdot}CH_2Cl_2$ (8.2 mg, 0.01 mmol, 4 mol%), K_3PO_4 (159 mg, 0.75 mmol, 3 equiv), THF (1 mL, 0.25 M) and H_2O (22.5 µL, 1.25 mmol, 5 equiv). After 24 h, the reaction mixture was subjected to the purification outlined in the General Procedure (C18 silica gel, 20-75% MeCN in H_2O) to afford the desired product as a beige solid (56 mg, 88%).

υ_{max} (film): 2978, 1396, 1359, 1143, 1091 cm^{-1}.

^{1}H NMR ($CDCl_3$, 400 MHz): δ 7.92 (d, J = 8.2 Hz, 2H), 7.63-7.66 (m, 4H), 7.45-7.49 (m, 2H), 7.38, (tt, J = 7.4, 1.2 Hz, 1H), 1.39 (s, 12H).

^{13}C NMR ($CDCl_3$, 126 MHz): δ 143.4, 140.6, 134.8, 128.3, 127.0, 126.7, 126.0, 83.3, 24.4.

^{11}B NMR ($CDCl_3$, 128 MHz): δ 31.3.

HRMS: exact mass calculated for $[M+H]^+$ ($C_{18}H_{22}BO_2$) requires *m/z* 281.1707, found *m/z* 281.1709.

N-(4'-(4,4,5,5-Tetramethyl-1,3,2-dioxaborolan-2-yl)-[1,1'-biphenyl]-4-yl)acetamide, **3b**

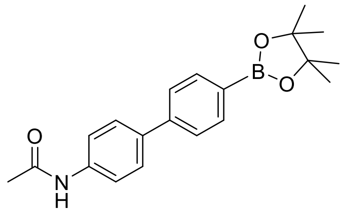

Prepared according to General Procedure A using 4-bromophenylboronic acid MIDA ester (78 mg, 0.25 mmol, 1 equiv), *N*-(4-(4,4,5,5-tetramethyl-1,3,2-dioxaborolan-2-yl)phenyl)acetamide (98 mg, 0.375 mmol, 1.5 equiv), Pd(dppf)Cl_2·CH_2Cl_2 (8.2 mg, 0.01 mmol, 4 mol%), K_3PO_4 (159 mg, 0.75 mmol, 3 equiv), THF (1 mL, 0.25 M), and H_2O (22.5 µL, 1.25 mmol, 5 equiv). After 24 h, the reaction mixture was subjected to the purification outlined in the general procedure (C18 silica gel, 20-50% MeCN in H_2O) to afford the desired product as a beige solid (72 mg, 86%).

υ_{max} (film): 3317, 2978, 2926, 1662, 1529, 1396, 1357, 1321, 1143, 1091, 819 cm^{-1}.

^{1}H NMR ($CDCl_3$, 400 MHz): δ 7.89 (d, *J* = 8.1 Hz, 2H), 7.59-7.61 (m, 6H), 2.23 (s, 3H), 1.39 (s, 12H).

^{13}C NMR ($CDCl_3$, 126 MHz): δ 167.7, 142.6, 136.9, 134.8, 127.2, 125.6, 119.5, 83.3, 29.2, 24.4.

^{11}B NMR ($CDCl_3$, 128 MHz): δ 32.0.

HRMS: exact mass calculated for $[M]^+$ ($C_{20}H_{24}BNO_3$) requires *m/z* 337.1958, found *m/z* 337.1962.

2-(3-Bromo-5-(trifluoromethyl)phenyl)-4,4,5,5-tetramethyl-1,3,2-dioxaborolane, **3c**

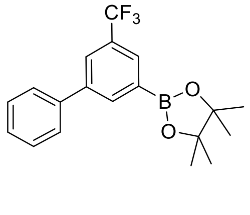

Prepared according to General Procedure B using 3-bromo-5-(trifluoromethyl)phenylboronic acid MIDA ester (88 mg, 0.25 mmol, 1 equiv), 4,4,5,5-tetramethyl-2-phenyl-1,3,2-dioxaborolane (76 mg, 0.375 mmol, 1.5 equiv), $Pd(OAc)_2$ (2.3 mg, 0.01 mmol, 4 mol%), SPhos (8.2 mg, 0.02 mmol, 8 mol%), K_3PO_4 (159 mg, 0.75 mmol, 3 equiv), THF (1 mL, 0.25 M), and H_2O (22.5 µL, 1.25 mmol, 5 equiv). After 24 h, the reaction mixture was subjected to the purification outlined in the general procedure (20-75% MeCN in H_2O) to afford the desired product as a yellow gum (53 mg, 60%).

υ_{max} (film): 2980, 2929, 1600, 1471 cm^{-1}.

^{1}H NMR ($CDCl_3$, 400 MHz): δ 8.22 (s, 1H), 8.06 (s, 1H), 7.93 (s, 1H), 7.62-7.69 (m, 2H), 7.46-7.53 (m, 2H), 7.42 (d, J = 7.3 Hz, 1H), 1.40 (s, 12H).

^{13}C NMR ($CDCl_3$, 126 MHz): δ 141.3, 139.7, 136.7, 130.1 (d, $^3J_{C\text{-}F}$ = 3.4 Hz), 128.9, 127.9, 127.3, 126.4 (d, $^3J_{C\text{-}F}$ = 3.4 Hz), 84.4, 24.9. CF_3 carbon and carbon bearing CF_3 not observed.

^{11}B NMR ($CDCl_3$, 128 MHz): δ 31.0.

^{19}F NMR ($CDCl_3$, 376 MHz): δ –62.4.

HRMS: exact mass calculated for $[M+H]^+$ ($C_{19}H_{21}BF_3O_2$) requires *m/z* 349.1581, found *m/z* 349.1581.

2-(2-Fluoro-4-(thiophen-2-yl)phenyl)-4,4,5,5-tetramethyl-1,3,2-dioxaborolane, **3d**

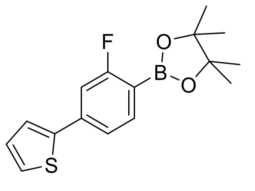

Prepared according to General Procedure A using 4-bromo-2-fluorophenylboronic acid MIDA ester (82 mg, 0.25 mmol, 1 equiv), 4,4,5,5-tetramethyl-2-(thiophen-2-yl)-1,3,2-dioxaborolane (79 mg, 0.375 mmol, 1.5 equiv), $Pd(dppf)Cl_2{\cdot}CH_2Cl_2$ (8.2 mg, 0.01 mmol, 4 mol%), K_3PO_4 (159 mg, 0.75 mmol, 3 equiv), THF (1 mL, 0.25 M), and H_2O (22.5 µL, 1.25 mmol, 5 equiv). After 24 h, the reaction mixture was subjected to the purification outlined in the General Procedure (C18 silica gel, 20-70% MeCN in H_2O) to afford the desired product as a brown solid (50 mg, 66%).

υ_{max} (film): 2978, 1618, 1413, 1386, 1354, 1325, 1134, 1070 cm^{-1}.

^{1}H NMR ($CDCl_3$, 400 MHz): δ 7.76 (dd, *J* = 7.7, 6.4 Hz, 1H), 7.38-7.42 (m, 2H), 7.29-7.37 (m, 2H), 7.12 (dd, *J* = 5.1, 3.6 Hz, 1H), 1.40 (s, 12H).

^{13}C NMR ($CDCl_3$, 126 MHz): δ 167.6 (d, $^{1}J_{C\text{-}F}$ = 250.8 Hz), 142.8, 139.4 (d, $^{3}J_{C\text{-}F}$ = 9.4 Hz), 137.4 (d, $^{3}J_{C\text{-}F}$ = 9.4 Hz), 128.2, 126.0, 124.3, 120.9, 112.3 (d, $^{2}J_{C\text{-}F}$ = 25.9 Hz), 83.9, 24.8.

^{11}B NMR ($CDCl_3$, 128 MHz): δ 30.2.

^{19}F NMR ($CDCl_3$, 376 MHz): δ –102.6.

HRMS: exact mass calculated for $[M]^+$ ($C_{16}H_{18}BFO_2S$) requires *m/z* 303.1138, found *m/z* 303.1138.

Methyl 1-(4'-(4,4,5,5-tetramethyl-1,3,2-dioxaborolan-2-yl)-[1,1'-biphenyl]-4-yl)cyclopropane-1-carboxylate, **3e**

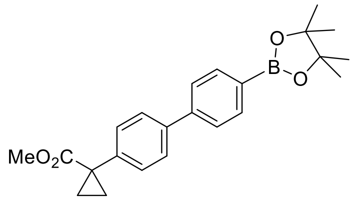

Prepared according to General Procedure A using 4-bromophenylboronic acid MIDA ester (78 mg, 0.25 mmol, 1 equiv), methyl 1-(4-(4,4,5,5-tetramethyl-1,3,2-dioxaborolan-2-yl)phenyl)cyclopropane-1-carboxylate (113 mg, 0.375 mmol, 1.5 equiv), Pd(dppf)Cl_2·CH_2Cl_2 (8.2 mg, 0.01 mmol, 4 mol%), K_3PO_4 (159 mg, 0.75 mmol, 3 equiv), THF (1 mL, 0.25 M), and H_2O (22.5 µL, 1.25 mmol, 5 equiv). After 24 h, the reaction mixture was subjected to the purification outlined in the General Procedure (C18 silica gel, 20-60% MeCN in H_2O) to afford the desired product as a beige solid (79 mg, 83%).

υ_{max} (film): 1724, 1359, 1298, 1166, 1143, 1093 cm^{-1}.

^{1}H NMR ($CDCl_3$, 400 MHz): δ 7.90 (d, *J* = 8.2 Hz, 2H), 7.61 (dd, *J* = 13.8, 8.2 Hz, 4H), 7.44 (d, *J* = 8.2 Hz, 2H), 3.68 (s, 3H), 1.66 (q, *J* = 3.9 Hz, 2H), 1.39 (s, 12H), 1.25 (q, *J* = 3.9 Hz, 2H).

^{13}C NMR ($CDCl_3$, 126 MHz): δ 175.0, 143.4, 139.9, 138.9, 135.2, 130.9, 127.0, 126.4, 83.8, 52.4, 29.7, 28.7, 24.9, 16.8.

^{11}B NMR ($CDCl_3$, 128 MHz): δ 31.5.

HRMS: exact mass calculated for $[M+H]^+$ ($C_{23}H_{28}BO_4$) requires *m/z* 379.2075, found *m/z* 379.2074.

trans-4,4,5,5-Tetramethyl-2-(2-styrylphenyl)-1,3,2-dioxaborolane, **3f**

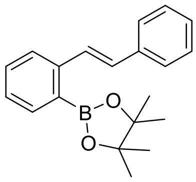

Prepared according to General Procedure A using 2-bromophenylboronic acid MIDA ester (78 mg, 0.25 mmol, 1 equiv), *trans*-4,4,5,5-tetramethyl-2-styryl-1,3,2-dioxaborolane (86 mg, 0.375 mmol, 1.5 equiv), $Pd(dppf)Cl_2·CH_2Cl_2$ (8.2 mg, 0.01 mmol, 4 mol%), K_3PO_4 (159 mg, 0.75 mmol, 3 equiv), THF (1 mL, 0.25 M), and H_2O (22.5 µL, 1.25 mmol, 5 equiv). After 24 h, the reaction mixture was subjected to the purification outlined in the General Procedure (C18 silica gel, 20-75% MeCN in H_2O) to afford the desired product as a brown solid (54 mg, 70%).

υ_{max} (film): 2978, 2360, 1373, 1346, 1313, 1143, 763 cm^{-1}.

^{1}H NMR ($CDCl_3$, 400 MHz): δ 8.08 (d, *J* = 16.3 Hz, 1H), 7.85 (dd, *J* = 7.5, 1.2 Hz, 1H), 7.77 (d, *J* = 7.9 Hz, 1H), 7.56-7.58 (m, 2H), 7.44-7.48 (m, 1H), 7.37-7.41 (m, 2H), 7.26-7.30 (m, 2H), 7.06 (d, *J* = 16.3 Hz, 1H), 1.42 (s, 12H).

^{13}C NMR ($CDCl_3$, 126 MHz): δ 143.5, 138.1, 136.2, 131.1, 129.9, 129.3, 128.7, 127.3, 126.6, 124.5, 83.8, 25.0.

^{11}B NMR ($CDCl_3$, 128 MHz): δ 31.1.

HRMS: exact mass calculated for $[M+H]^+$ ($C_{20}H_{24}BO_2$) requires *m/z* 307.1864, found *m/z* 307.1863.

3'-Fluoro-4'-(4,4,5,5-tetramethyl-1,3,2-dioxaborolan-2-yl)-[1,1'-biphenyl]-3-carbonitrile, **3g**

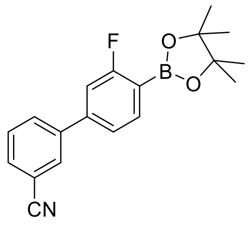

Prepared according to general procedure A using 4-bromo-2-fluorophenylboronic acid MIDA ester (82 mg, 0.25 mmol, 1 equiv), 3-(4,4,5,5-tetramethyl-1,3,2-dioxaborolan-2-yl)benzonitrile (86 mg, 0.375 mmol, 1.5 equiv), Pd(dppf)Cl_2·CH_2Cl_2 (8.2 mg, 0.01 mmol, 4 mol%), K_3PO_4 (159 mg, 0.75 mmol, 3 equiv), THF (1 mL, 0.25 M), and H_2O (22.5 µL, 1.25 mmol, 5 equiv). After 24 h, the reaction mixture was subjected to the purification outlined in the General Procedure (C18 silica gel, 20-75% MeCN in H_2O) to afford the desired product as a brown solid (43 mg, 54%).

υ_{max} (film): 2980, 2927, 2229, 1622, 1390, 1354, 1330, 1138, 1080 cm^{-1}.

^{1}H NMR ($CDCl_3$, 400 MHz): δ 7.81-7.88 (m, 3H), 7.66-7.68 (m, 1H), 7.57 (t, J = 7.8 Hz, 1H), 7.36 (dd, J = 7.8, 1.6 Hz, 1H), 7.25-7.27 (m, 1H), 1.39 (s, 12H).

^{13}C NMR ($CDCl_3$, 126 MHz): δ 167.6 (d, $^1J_{C\text{-}F}$ = 251.8 Hz), 143.9 (d, $^3J_{C\text{-}F}$ = 8.7 Hz), 140.9, 137.7 (d, $^3J_{C\text{-}F}$ = 8.7 Hz), 131.5, 131.4, 130.7, 129.8, 122.2, 118.5, 113.9, 113.5 (d, $^2J_{C\text{-}F}$ = 47.8 Hz), 84.1, 24.8.

^{11}B NMR ($CDCl_3$, 128 MHz): δ 30.9.

^{19}F NMR ($CDCl_3$, 376 MHz): δ –101.8.

HRMS: exact mass calculated for $[M]^+$ ($C_{19}H_{19}BFNO_2$) requires m/z 322.1520, found m/z 322.1524.

4,4,5,5-Tetramethyl-2-(4'-(trifluoromethoxy)-[1,1'-biphenyl]-3-yl)-1,3,2-dioxaborolane, **3h**

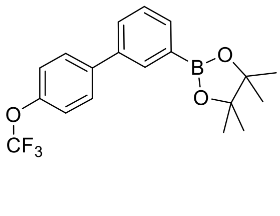

Prepared according to General Procedure A using 3-bromophenylboronic acid MIDA ester (78 mg, 0.25 mmol, 1 equiv), 4,4,5,5-tetramethyl-2-(4-(trifluoromethoxy)phenyl)-1,3,2-dioxaborolane (108 mg, 0.375 mmol, 1.5 equiv), Pd(dppf)Cl_2·CH_2Cl_2 (8.2 mg, 0.01 mmol, 4 mol%), K_3PO_4 (159 mg, 0.75 mmol, 3 equiv), THF (1 mL, 0.25 M), and H_2O (22.5 µL, 1.25 mmol, 5 equiv). After 24 h, the reaction mixture was subjected to the purification outlined in the General Procedure (C18 silica gel, 20-60% MeCN in H_2O) to afford the desired product as a brown solid (73 mg, 80%).

υ_{max} (film): 1359, 1255, 1213, 1165, 1143 cm^{-1}.

^{1}H NMR ($CDCl_3$, 400 MHz): δ 7.84 (dt, *J* = 7.3, 1.1 Hz, 1H), 7.65-7.69 (m, 3H), 7.48 (t, *J* = 7.5 Hz, 1H), 7.28-7.31 (m, 3H), 1.39 (s, 12H).

^{13}C NMR ($CDCl_3$, 126 MHz): δ 140.0, 139.2, 136.5, 134.0, 133.5, 129.9, 128.6, 128.3, 121.1, 84.0, 24.9. CF_3 carbon not observed.

^{11}B NMR ($CDCl_3$, 128 MHz): δ 31.3.

^{19}F NMR ($CDCl_3$, 376 MHz): δ –57.8 (s, 3F).

HRMS: exact mass calculated for $[M+H]^+$ ($C_{19}H_{21}BF_3O_3$) requires *m/z* 365.1530, found *m/z* 365.1530.

1-Methyl-4-(2-(4,4,5,5-tetramethyl-1,3,2-dioxaborolan-2-yl)phenyl)-1*H*-pyrazole, **3i**

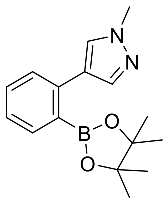

Prepared according to General Procedure A using 2-bromophenylboronic acid MIDA ester (78 mg, 0.25 mmol, 1 equiv), 1-methyl-4-(4,4,5,5-tetramethyl-1,3,2-dioxaborolan-2-yl)-1*H*-pyrazole (78 mg, 0.375 mmol, 1.5 equiv), Pd(dppf)Cl_2·CH_2Cl_2 (8.2 mg, 0.01 mmol, 4 mol%), K_3PO_4 (159 mg, 0.75 mmol, 3 equiv), THF (1 mL, 0.25 M), and H_2O (22.5 µL, 1.25 mmol, 5 equiv). After 24 h, the reaction mixture was subjected to the purification outlined in the General Procedure (C18 silica gel, 10-40% MeCN in H_2O) to afford the desired product as a colourless oil (48 mg, 68%).

υ_{max} (film): 2978, 2926, 1381, 1350, 1317, 1143, 858 cm^{-1}.

^{1}H NMR ($CDCl_3$, 400 MHz): δ 7.81 (dd, J = 7.3, 1.2 Hz, 1H), 7.41-7.49 (m, 3H), 7.28 (dd, J = 7.3, 1.2 Hz, 1H), 6.19 (s, 1H), 3.67 (s, 3H), 1.19 (s, 12H).

^{13}C NMR ($CDCl_3$, 126 MHz): δ 137.4, 134.8, 130.4, 130.0, 128.1, 106.7, 83.8, 36.7, 24.6.

^{11}B NMR ($CDCl_3$, 128 MHz): δ 31.1.

HRMS: exact mass calculated for $[M+H]^+$ ($C_{16}H_{22}BN_2O_2$) requires *m/z* 285.1769, found *m/z* 285.1768.

2-(3-(Furan-3-yl)-5-(trifluoromethyl)phenyl)-4,4,5,5-tetramethyl-1,3,2-dioxaborolane, **3j**

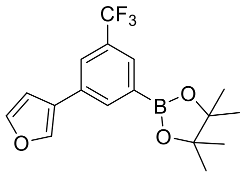

Prepared according to General Procedure B using 3-bromo-5-(trifluoromethyl)phenylboronic acid MIDA ester (88 mg, 0.25 mmol, 1 equiv), 2-(furan-3-yl)-4,4,5,5-tetramethyl-1,3,2-dioxaborolane (73 mg, 0.375 mmol, 1.5 equiv), $Pd(OAc)_2$ (2.3 mg, 0.01 mmol, 4 mol%), SPhos (8.2 mg, 0.02 mmol, 8 mol%), K_3PO_4 (159 mg, 0.75 mmol, 3 equiv), THF (1 mL, 0.25 M), and H_2O (22.5 μL, 1.25 mmol, 5 equiv). After 24 h, the reaction mixture was subjected to the purification outlined in the General Procedure (C18 silica gel, 10-75% MeCN in H_2O) to afford the desired product as a brown gum (45 mg, 53%).

υ_{max} (film): 2980, 2932, 1304, 1279, 1166, 1125, 871 cm^{-1}.

^{1}H NMR ($CDCl_3$, 400 MHz): δ 8.09 (s, 1H), 7.97 (s, 1H), 7.84-7.85 (m, 1H), 7.81 (br. s, 1H), 7.52 (t, J = 1.7 Hz, 1H), 6.79 (dd, J = 1.7, 0.9 Hz, 1H), 1.40 (s, 12H).

^{13}C NMR ($CDCl_3$, 126 MHz): δ 144.0, 139.2, 135.2, 132.6, 129.8 (d, $^3J_{C-F}$ = 4.0 Hz), 125.3, 125.0 (d, $^3J_{C-F}$ = 4.0 Hz), 108.7, 103.6, 84.4, 24.9. CF_3 carbon not observed.

^{11}B NMR ($CDCl_3$, 128 MHz): δ 31.2.

^{19}F NMR ($CDCl_3$, 376 MHz): δ –62.7.

HRMS: exact mass calculated for $[M]^+$ ($C_{17}H_{18}BF_3O_3$) requires *m/z* 337.1334, found *m/z* 337.1332.

2-(3-(Benzo[b]thiophen-2-yl)phenyl)-4,4,5,5-tetramethyl-1,3,2-dioxaborolane, **3k**

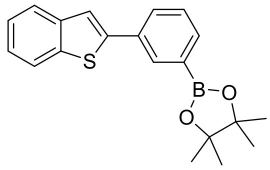

Prepared according to General Procedure A using 3-bromophenylboronic acid MIDA ester (78 mg, 0.25 mmol, 1 equiv), 2-(benzo[b]thiophen-2-yl)-4,4,5,5-tetramethyl-1,3,2-dioxaborolane (98 mg, 0.375 mmol, 1.5 equiv), Pd(dppf)Cl_2·CH_2Cl_2 (8.2 mg, 0.01 mmol, 4 mol%), K_3PO_4 (159 mg, 0.75 mmol, 3 equiv), THF (1 mL, 0.25 M), and H_2O (22.5 μL, 1.25 mmol, 5 equiv). After 24 h, the reaction mixture was subjected to the purification outlined in the General Procedure (C18 silica gel, 20-80% MeCN in H_2O) to afford the desired product as a brown solid (74 mg, 88%).

υ_{max} (film): 2976, 1355, 1317, 1141 cm^{-1}.

^{1}H NMR ($CDCl_3$, 400 MHz): δ 8.20 (br. s, 1H), 7.79-7.87 (m, 4H), 7.64 (s, 1H), 7.46 (t, *J* = 7.6 Hz, 1H), 7.31-7.40 (m, 2H), 1.40 (s, 12H).

^{13}C NMR ($CDCl_3$, 126 MHz): δ 144.3, 140.7, 139.6, 134.6, 133.7, 132.7, 129.3, 128.3, 124.4, 124.2, 123.5, 122.3, 119.6, 84.0, 24.9.

^{11}B NMR ($CDCl_3$, 128 MHz): δ 31.4.

HRMS: exact mass calculated for $[M+H]^+$ ($C_{20}H_{22}BO_2S$) requires *m/z* 337.1428, found *m/z* 337.1427.

2-(4-(3,6-Dihydro-2*H*-pyran-4-yl)phenyl)-4,4,5,5-tetramethyl-1,3,2-dioxaborolane, **3l**

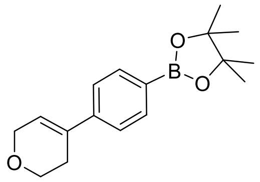

Prepared according to General Procedure A using 4-bromophenylboronic acid MIDA ester (78 mg, 0.25 mmol, 1 equiv), 2-(3,6-dihydro-2*H*-pyran-4-yl)-4,4,5,5-tetramethyl-1,3,2-dioxaborolane (79 mg, 0.375 mmol, 1.5 equiv), Pd(dppf)Cl_2·CH_2Cl_2 (8.2 mg, 0.01 mmol, 4 mol%), K_3PO_4 (159 mg, 0.75 mmol, 3 equiv), THF (1 mL, 0.25 M), and H_2O (22.5 μL, 1.25 mmol, 5 equiv). After 24 h, the reaction mixture was subjected to the purification outlined in the General Procedure (C18 silica gel, 20-60% MeCN in H_2O) to afford the desired product as a brown solid (60 mg, 84%).

υ_{max} (film): 2976, 2926, 1606, 1398, 1357, 1323, 1143, 1091 cm^{-1}.

^{1}H NMR ($CDCl_3$, 400 MHz): δ 7.81 (d, *J* = 8.3 Hz, 2H), 7.42 (d, *J* = 8.3 Hz, 2H), 6.22 (tt, *J* = 2.8, 1.5 Hz, 1H), 4.36 (q, *J* = 2.8 Hz, 2H), 3.96 (t, *J* = 5.5 Hz, 2H), 2.54-2.58 (m, 2H), 1.37 (s, 12H).

^{13}C NMR ($CDCl_3$, 126 MHz): δ 142.9, 134.9, 134.2, 124.0, 123.3, 83.8, 65.9, 64.4, 27.1, 24.9.

^{11}B NMR ($CDCl_3$, 128 MHz): δ 31.2.

HRMS: exact mass calculated for $[M+H]^+$ ($C_{17}H_{24}BO_3$) requires *m/z* 287.1813, found *m/z* 287.1810.

2-(2-(Furan-3-yl)phenyl)-4,4,5,5-tetramethyl-1,3,2-dioxaborolane, **3m**

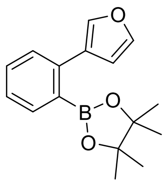

Prepared according to General Procedure A using 2-bromophenylboronic acid MIDA ester (78 mg, 0.25 mmol, 1 equiv), 2-(furan-3-yl)-4,4,5,5-tetramethyl-1,3,2-dioxaborolane (73 mg, 0.375 mmol, 1.5 equiv), Pd(dppf)Cl_2·CH_2Cl_2 (8.2 mg, 0.01 mmol, 4 mol%), K_3PO_4 (159 mg, 0.75 mmol, 3 equiv), THF (1 mL, 0.25 M), and H_2O (22.5 μL, 1.25 mmol, 5 equiv). After 24 h, the reaction mixture was subjected to the purification outlined in the General Procedure (C18 silica gel, 20-60% MeCN in H_2O) to afford the desired product as a brown liquid (47 mg, 70%).

υ_{max} (film): 2978, 1483, 1438, 1348, 1311, 1143, 873, 759 cm^{-1}.

^{1}H NMR ($CDCl_3$, 400 MHz): δ 7.71 (dd, J = 7.4, 1.1 Hz, 1H), 7.60 (dd, J = 1.5, 0.9 Hz, 1H), 7.44 (t, J = 1.7 Hz, 1H), 7.42 (dd, J = 7.5, 1.5 Hz, 1H), 7.36 (dd, J = 7.7, 0.7 Hz, 1H), 7.30 (td, J = 7.4, 1.3 Hz, 1H), 6.60 (dd, J = 1.8, 0.8 Hz, 1H), 1.31 (s, 12H).

^{13}C NMR ($CDCl_3$, 126 MHz): δ 142.1, 140.0, 137.7, 134.8, 130.3, 128.9, 127.4, 126.3, 112.1, 83.8, 24.7.

^{11}B NMR ($CDCl_3$, 128 MHz): δ 31.7.

HRMS: exact mass calculated for $[M+H]^+$ ($C_{16}H_{20}BO_3$) requires *m/z* 271.1500, found *m/z* 271.1498.

Methyl 2-(3'-(4,4,5,5-tetramethyl-1,3,2-dioxaborolan-2-yl)-[1,1'-biphenyl]-4-yl)acetate, **3n**

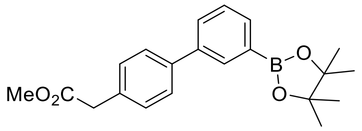

Prepared according to General Procedure A using 3-bromophenylboronic acid MIDA ester (78 mg, 0.25 mmol, 1 equiv), methyl 2-(4-(4,4,5,5-tetramethyl-1,3,2-dioxaborolan-2-yl)phenyl)acetate (104 mg, 0.375 mmol, 1.5 equiv), $Pd(dppf)Cl_2{\cdot}CH_2Cl_2$ (8.2 mg, 0.01 mmol, 4 mol%), K_3PO_4 (159 mg, 0.75 mmol, 3 equiv), THF (1 mL, 0.25 M), and H_2O (22.5 µL, 1.25 mmol, 5 equiv). After 24 h, the reaction mixture was subjected to the purification outlined in the General Procedure (C18 silica gel, 20-50% MeCN in H_2O) to afford the desired product as an orange/brown oil (71 mg, 80%).

υ_{max} (film): 2978, 1737, 1431, 1355, 1317, 1257, 1143 cm^{-1}.

^{1}H NMR ($CDCl_3$, 400 MHz): δ 8.06 (br. s, 1H), 7.81 (dt, J = 7.3, 1.1 Hz, 1H), 7.70 (ddd, J = 7.8, 2.0, 1.3 Hz, 1H), 7.60-7.63 (m, 2H), 7.45-7.49 (m, 1H), 7.36-7.38 (m, 2H), 3.74 (s, 3H), 3.70 (s, 2H), 1.39 (s, 12H).

^{13}C NMR ($CDCl_3$, 126 MHz): δ 172.0, 140.1, 135.1, 133.7, 133.5, 132.9, 129.9, 129.6, 128.7, 128.2, 127.5, 83.9, 52.1, 40.9, 24.9.

^{11}B NMR ($CDCl_3$, 128 MHz): δ 31.2.

HRMS: exact mass calculated for $[M+H]^+$ ($C_{21}H_{26}BO_4$) requires *m/z* 353.1919, found *m/z* 353.1924.

Methyl 2-(3'-methoxy-5'-(4,4,5,5-tetramethyl-1,3,2-dioxaborolan-2-yl)-[1,1'-biphenyl]-4-yl)acetate, **3o**

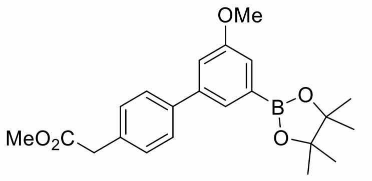

Prepared according to General Procedure B using (3-chloro-5-methoxyphenyl)boronic acid MIDA ester (74 mg, 0.25 mmol, 1 equiv), methyl 2-(4-(4,4,5,5-tetramethyl-1,3,2-dioxaborolan-2-yl)phenyl)acetate (104 mg, 0.375 mmol, 1.5 equiv), $Pd(OAc)_2$ (2.3 mg, 0.01 mmol, 4 mol%), SPhos (8.2 mg, 0.02 mmol, 8 mol%), K_3PO_4 (159 mg, 0.75 mmol, 3 equiv), THF (1 mL, 0.25 M), and H_2O (22.5 µL, 1.25 mmol, 5 equiv). After 24 h, the reaction mixture was subjected to the purification outlined in the General Procedure (C18 silica gel, 20-60% MeCN in H_2O) to afford the desired product as an off-white solid (60 mg, 63%).

υ_{max} (film): 2978, 2358, 1373, 1575, 1452, 1369 cm^{-1}.

^1H NMR (CD_3CN, 400 MHz): δ 7.66 (dd, *J* = 1.7, 0.7 Hz, 1H), 7.61 (d, *J* = 8.3 Hz, 2H), 7.36 (d, *J* = 8.3 Hz, 2H), 7.33 (dd, *J* = 2.6, 0.7 Hz, 1H), 7.24 (dd, *J* = 2.6, 1.7 Hz, 1H), 3.91 (s, 3H), 3.74 (s, 3H), 3.69 (s, 2H), 1.38 (s, 12H).

^{13}C NMR (CD_3CN, 126 MHz): δ 172.0, 159.5, 141.7, 139.8, 133.1, 129.5, 127.5, 126.1, 117.5, 116.6,83.9, 55.4, 52.1, 40.9, 24.9.

^{11}B NMR (CD_3CN, 128 MHz): δ 31.7.

HRMS: exact mass calculated for $[M+Na]^+$ ($C_{22}H_{27}BO_5Na$) requires *m/z* 405.1844, found *m/z* 405.1846.

4,4,5,5-Tetramethyl-2-(2',3',4',5'-tetrahydro-[1,1'-biphenyl]-3-yl)-1,3,2-dioxaborolane, **3p**

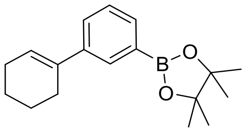

Prepared according to General Procedure A using 3-bromophenylboronic acid MIDA ester (78 mg, 0.25 mmol, 1 equiv), 2-(cyclohex-1-en-1-yl)-4,4,5,5-tetramethyl-1,3,2-dioxaborolane (108 mg, 0.375 mmol, 1.5 equiv), Pd(dppf)Cl_2·CH_2Cl_2 (8.2 mg, 0.01 mmol, 4 mol%), K_3PO_4 (159 mg, 0.75 mmol, 3 equiv), THF (1 mL, 0.25 M), and H_2O (22.5 µL, 1.25 mmol, 5 equiv). After 24 h, the reaction mixture was subjected to the purification outlined in the General Procedure (C18 silica gel, 20-90% MeCN in H_2O) to afford the desired product as a beige solid (60 mg, 85%).

υ_{max} (film): 2976, 2927, 1356, 1311, 1271, 1143 cm^{-1}.

^{1}H NMR ($CDCl_3$, 400 MHz): δ 7.85 (br. s, 1H), 7.67-7.69 (m, 1H), 7.47-7.50 (m, 1H), 7.33 (t, J = 7.5 Hz, 1H), 6.13-6.16 (m, 1H), 2.43-2.48 (m, 2H), 2.20-2.25 (m, 2H), 1.77-1.83 (m, 2H), 1.65-1.71 (m, 2H), 1.37 (s, 12H).

^{13}C NMR ($CDCl_3$, 126 MHz): δ 142.1, 136.7, 132.9, 131.4, 128.0, 127.6, 124.9, 83.7, 27.5, 25.8, 24.9, 23.1, 22.2.

^{11}B NMR ($CDCl_3$, 128 MHz): δ 31.3.

HRMS: exact mass calculated for $[M+H]^+$ ($C_{18}H_{26}BO_2$) requires *m/z* 285.2020, found *m/z* 285.2020.

2-(2,4-Difluorophenyl)-6-(4,4,5,5-tetramethyl-1,3,2-dioxaborolan-2-yl)quinolone, **3q**

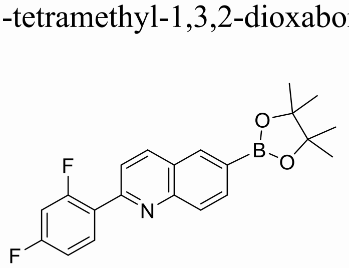

Prepared according to General Procedure B using (2-chloroquinolin-6-yl)boronic acid MIDA ester (80 mg, 0.25 mmol, 1 equiv), 2-(2,4-difluorophenyl)-4,4,5,5-tetramethyl-1,3,2-dioxaborolane (90 mg, 0.375 mmol, 1.5 equiv), Pd(OAc)$_2$ (2.3 mg, 0.01 mmol, 4 mol%), SPhos (8.2 mg, 0.02 mmol, 8 mol%), K_3PO_4 (159 mg, 0.75 mmol, 3 equiv), THF (1 mL, 0.25

M), and H_2O (22.5 µL, 1.25 mmol, 5 equiv). After 24 h, the reaction mixture was subjected to the purification outlined in the General Procedure (C18 silica gel, 10-60% MeCN in H_2O) to afford the desired product as an off-white solid (29 mg, 32%).

υ_{max} (film): 2978, 2358, 1600, 1471, 1357, 1300, 1261, 1141, 1103, 850 cm^{-1}.

^{1}H NMR (CD_3CN, 400 MHz): δ 8.41 (d, J = 8.7 Hz, 1H), 8.36 (s, 1H), 8.02-8.20 (m, 3H), 7.91 (dd, J = 8.7, 2.7 Hz, 1H), 7.10-7.19 (m, 2H), 1.40 (s, 12H).

^{13}C NMR (CD_3CN, 126 MHz): δ 163.3 (dd, $^1J_{C\text{-}F}$ = 249.5 ,$^3J_{C\text{-}F}$ = 12.1 Hz), 160.6 (dd, $^1J_{C\text{-}F}$ = 251.3, $^3J_{C\text{-}F}$ = 12.3 Hz), 154.8, 142.5, 140.9, 139.5, 138.2, 133.9, 133.4, 131.9, 129.5 (dd, $J_{C\text{-}F}$ = 12.2, $J_{C\text{-}F}$ = 3.7 Hz), 127.4, 117.3 (dd, $J_{C\text{-}F}$ = 21.3, $J_{C\text{-}F}$ = 3.3 Hz), 109.7 (t, $J_{C\text{-}F}$ = 26.7 Hz), 89.5, 29.6.

^{11}B NMR ($CDCl_3$, 128 MHz): δ 36.5.

^{19}F NMR ($CDCl_3$, 376 MHz): δ –104.9 (d, $J_{F\text{-}F}$ = 8.7 Hz, 1F), –108.4 (d, $J_{F\text{-}F}$ = 8.7 Hz, 1F).

HRMS: exact mass calculated for $[M+H]^+$ ($C_{21}H_{21}BF_2NO_2$) requires *m/z* 368.1628, found *m/z* 368.1629.

2-(3-Fluoro-[1,1'-biphenyl]-4-yl)-4,4,5,5-tetramethyl-1,3,2-dioxaborolane, **3r**

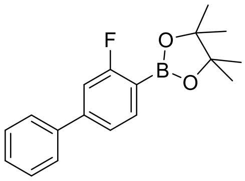

Prepared according to General Procedure A using 4-bromo-2-fluorophenylboronic acid MIDA ester (82 mg, 0.25 mmol, 1 equiv), 4,4,5,5-tetramethyl-2-phenyl-1,3,2-dioxaborolane (76 mg, 0.375 mmol, 1.5 equiv), Pd(dppf)Cl_2·CH_2Cl_2 (8.2 mg, 0.01 mmol, 4 mol%), K_3PO_4 (159 mg, 0.75 mmol, 3 equiv), THF (1 mL, 0.25 M), and H_2O (22.5 µL, 1.25 mmol, 5 equiv). After 24 h, the reaction mixture was subjected to the purification outlined in the General Procedure (C18 silica gel, 20-80% MeCN in H_2O) to afford the desired product as a brown solid (53 mg, 72%).

υ_{max} (film): 2978, 1620, 1404, 1384, 1354, 1325, 1136, 1078 cm^{-1}.

^{1}H NMR ($CDCl_3$, 400 MHz): δ 7.84 (t, J = 7.0 Hz, 1H), 7.63 (d, J = 7.3 Hz, 2H), 7.41-7.50 (m, 5H), 7.30-7.33 (m, 1H), 1.42 (s, 12H).

^{13}C NMR ($CDCl_3$, 126 MHz): δ 167.7 (d, $^{1}J_{C\text{-}F}$ = 250.6 Hz), 146.6 (d, $^{3}J_{C\text{-}F}$ = 8.6 Hz), 139.6, 137.2 (d, $^{3}J_{C\text{-}F}$ = 8.6 Hz), 128.9, 128.2, 127.1, 122.2, 113.7 (d, $^{2}J_{C\text{-}F}$ = 25.0 Hz), 83.9, 24.8.

^{11}B NMR ($CDCl_3$, 128 MHz): δ 29.9.

^{19}F NMR ($CDCl_3$, 376 MHz): δ –102.6.

HRMS: exact mass calculated for $[M+H]^+$ ($C_{18}H_{21}BFO_2$) requires *m/z* 299.1616, found *m/z* 299.1613.

trans-4,4,5,5-Tetramethyl-2-(4-(thiophen-2-yl)styryl)-1,3,2-dioxaborolane, **3s**

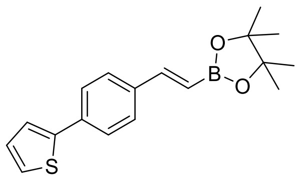

Prepared according to General Procedure A using *trans*-2-(4-bromophenyl)vinylboronic acid MIDA ester (85 mg, 0.25 mmol, 1 equiv), 4,4,5,5-tetramethyl-2-(thiophen-2-yl)-1,3,2-dioxaborolane (79 mg, 0.375 mmol, 1.5 equiv), $Pd(dppf)Cl_2{\cdot}CH_2Cl_2$ (8.2 mg, 0.01 mmol, 4 mol%), K_3PO_4 (159 mg, 0.75 mmol, 3 equiv), THF (1 mL, 0.25 M), and H_2O (22.5 μL, 1.25 mmol, 5 equiv). After 24 h, the reaction mixture was subjected to the purification outlined in the General Procedure (C18 silica gel, 20-75% MeCN in H_2O) to afford the desired product as a beige solid (62 mg, 79%).

υ_{max} (film): 2978, 2358, 1622, 1357, 1323, 1213, 1143, 808 cm^{-1}.

^{1}H NMR ($CDCl_3$, 400 MHz): δ 7.51-7.63 (m, 4H), 7.42 (d, J = 18.4 Hz, 1H), 7.36 (dd, J = 3.6, 1.1 Hz, 1H), 7.31 (dd, J = 5.1, 1.1 Hz, 1H), 7.11 (dd, J = 5.1, 3.6 Hz, 1H), 6.20 (d, J = 18.4 Hz, 1H), 1.35 (s, 12H).

^{13}C NMR ($CDCl_3$, 126 MHz): δ 148.8, 144.0, 136.6, 134.8, 128.1, 127.6, 126.0, 125.1, 123.3, 83.4, 24.8.

^{11}B NMR ($CDCl_3$, 128 MHz): δ 30.7.

HRMS: exact mass calculated for $[M+H]^+$ ($C_{20}H_{21}BF_3O_2$) requires *m/z* 361.1581, found *m/z* 361.1577.

trans-4,4,5,5-Tetramethyl-2-(2-(thiophen-2-yl)vinyl)-1,3,2-dioxaborolane, **3t**

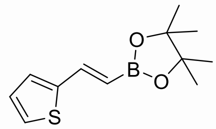

Prepared according to General Procedure A using *trans*-2-iodovinylboronic acid MIDA ester (77 mg, 0.25 mmol, 1 equiv), 4,4,5,5-tetramethyl-2-(thiophen-2-yl)-1,3,2-dioxaborolane (79 mg, 0.375 mmol, 1.5 equiv), $Pd(dppf)Cl_2{\cdot}CH_2Cl_2$ (8.2 mg, 0.01 mmol, 4 mol%), K_3PO_4 (159 mg, 0.75 mmol, 3 equiv), THF (1 mL, 0.25 M), and H_2O (22.5 µL, 1.25 mmol, 5 equiv). After 24 h, the reaction mixture was subjected to purification by reverse phase preparative HPLC (20-95% MeCN in H_2O) to afford the desired product as a colourless liquid (40 mg, 68%).

υ_{max} (film): 2978, 2926, 2854, 1616, 1371, 1325, 1143, 848 cm^{-1}.

^{1}H NMR ($CDCl_3$, 400 MHz): δ 7.48 (d, J = 18.1 Hz, 1H), 7.25 (d, *J* = 5.0 Hz, 1H), 7.09 (d, *J* = 3.1 Hz, 1H), 6.99 (dd, *J* = 5.0 Hz, 1H), 5..92 (d, *J* = 18.1 Hz, 1H), 1.31 (s, 12H).

^{13}C NMR ($CDCl_3$, 126 MHz): δ 143.9, 141.8, 127.7, 127.6, 126.3, 83.4, 24.8.

^{11}B NMR ($CDCl_3$, 128 MHz): δ 30.3.

HRMS: exact mass calculated for $[M+H]^+$ ($C_{12}H_{17}BO_2S$) requires *m/z* 237.1115, found *m/z* 237.1114.

trans-2-(2,4-Difluorostyryl)-4,4,5,5-tetramethyl-1,3,2-dioxaborolane, **3u**

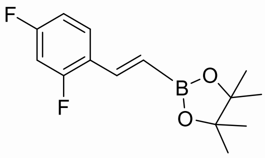

Prepared according to General Procedure A using *trans*-2-bromovinylboronic acid MIDA ester (65 mg, 0.25 mmol, 1 equiv), 2-(2,4-difluorophenyl)-4,4,5,5-tetramethyl-1,3,2-dioxaborolane (90 mg, 0.375 mmol, 1.5 equiv), Pd(dppf)Cl_2·CH_2Cl_2 (8.2 mg, 0.01 mmol, 4 mol%), K_3PO_4 (159 mg, 0.75 mmol, 3 equiv), THF (1 mL, 0.25 M), and H_2O (22.5 µL, 1.25 mmol, 5 equiv). After 24 h, the reaction mixture was subjected to purification by reverse phase preparative HPLC (20-95% MeCN in H_2O) to afford the desired product as a yellow oil (54 mg, 81%).

υ_{max} (film): 2980, 1627, 1500, 1350, 1328, 1141, 968, 850 cm^{-1}.

^{1}H NMR ($CDCl_3$, 400 MHz): δ 7.48-7.57 (m, 2H), 6.89-6.84 (m, 1H), 6.83-6.77 (m, 1H), 6.17 (d, J = 18.6 Hz, 1H), 1.32 (s, 12H).

^{13}C NMR ($CDCl_3$, 126 MHz): δ 163.2 (dd, $^1J_{C-F}$ = 226.8, $^3J_{C-F}$ = 12.0 Hz), 160.7 (dd, $^1J_{C-F}$ = 230.6, $^3J_{C-F}$ = 12.0 Hz), 140.3 (d, $^3J_{C-F}$ = 2.6 Hz), 128.4 (dd, J_{C-F} = 9.6, J_{C-F} = 4.9 Hz), 121.9 (dd, J_{C-F} = 11.9, J_{C-F} = 3.8 Hz), 111.6 (dd, J_{C-F} = 21.6, J_{C-F} = 3.4 Hz), 104.1 (t, J_{C-F} = 25.6 Hz), 83.5, 24.8.

^{11}B NMR ($CDCl_3$, 128 MHz): δ 30.2.

^{19}F NMR ($CDCl_3$, 376 MHz): δ –111.3 (d, J_{F-F} = 7.5 Hz, 1F), –113.2 (d, J_{F-F} = 7.5 Hz, 1F).

HRMS: exact mass calculated for $[M+H]^+$ ($C_{14}H_{18}BF_2O_2$) requires *m/z* 267.1362, found *m/z* 267.1363.

trans-3,5-Dimethyl-4-(2-(4,4,5,5-tetramethyl-1,3,2-dioxaborolan-2-yl)vinyl)isoxazole, **3v**

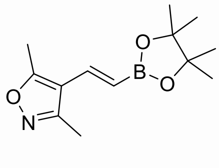

Prepared according to General Procedure A using *trans*-2-iodovinylboronic acid MIDA ester (65 mg, 0.25 mmol, 1 equiv), 3,5-dimethyl-4-(4,4,5,5-tetramethyl-1,3,2-dioxaborolan-2-yl)isoxazole (84 mg, 0.375 mmol, 1.5 equiv), Pd(dppf)Cl_2·CH_2Cl_2 (8.2 mg, 0.01 mmol, 4 mol%), K_3PO_4 (159 mg, 0.75 mmol, 3 equiv), THF (1 mL, 0.25 M), and H_2O (22.5 µL, 1.25

mmol, 5 equiv). After 24 h, the reaction mixture was subjected to purification *via* column chromatography on silica (10-20% EtOAc in petroleum ether) to afford the desired product as a beige solid (31 mg, 50%).

υ_{max} (film): 2978, 1641, 1344, 1325, 1269, 1141, 968, 850 cm^{-1}.

^{1}H NMR ($CDCl_3$, 400 MHz): δ 7.11 (d, *J* = 18.8 Hz, 1H), 5.83 (d, *J* = 18.8 Hz, 1H), 2.47 (s, 3H), 2.37 (s, 3H), 1.32 (s, 12H).

^{13}C NMR ($CDCl_3$, 126 MHz): δ 167.5, 158.5, 137.3, 113.9, 83.5, 24.8, 11.8, 11.6.

^{11}B NMR ($CDCl_3$, 128 MHz): δ 29.5.

HRMS: exact mass calculated for $[M+H]^+$ ($C_{13}H_{21}BNO_3$) requires *m/z* 250.1609, found *m/z* 250.1605.

trans-4,4,5,5-Tetramethyl-2-(2-(3',4',5'-trifluoro-[1,1'-biphenyl]-4-yl)vinyl)-1,3,2-dioxaborolane, **3w**

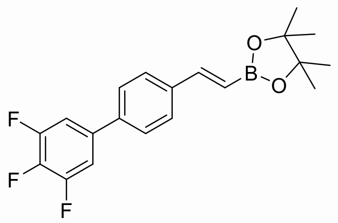

Prepared according to General Procedure A using *trans*-2-(4-bromophenyl)vinylboronic acid MIDA ester (85 mg, 0.25 mmol, 1 equiv), 4,4,5,5-tetramethyl-2-(3,4,5-trifluorophenyl)-1,3,2-dioxaborolane (97 mg, 0.375 mmol, 1.5 equiv), $Pd(dppf)Cl_2{\cdot}CH_2Cl_2$ (8.2 mg, 0.01 mmol, 4 mol%), K_3PO_4 (159 mg, 0.75 mmol, 3 equiv), THF (1 mL, 0.25 M), and H_2O (22.5 μL, 1.25 mmol, 5 equiv). After 24 h, the reaction mixture was subjected to the purification outlined in the General Procedure (C18 silica gel, 20-70% MeCN in H_2O) to afford the desired product as a brown liquid (64 mg, 71%).

υ_{max} (film): 2981, 1618, 1537, 1508, 1381, 1358, 1323, 1247, 1141, 1043, 806 cm^{-1}.

^{1}H NMR ($CDCl_3$, 400 MHz): δ 7.48-7.61 (m, 4H), 7.44 (d, *J* = 18.4 Hz, 1H), 7.22 (dd, *J* = 8.9, 6.5 Hz, 2H), 6.25 (d, *J* = 18.4 Hz, 1H), 1.35 (s, 12H).

^{13}C NMR ($CDCl_3$, 126 MHz): δ 151.5 (ddd, $^{1}J_{C\text{-}F}$ = 249.4, $^{2}J_{C\text{-}F}$ = 10.0, $^{3}J_{C\text{-}F}$ = 3.9 Hz,), 148.3, 139.3 (dt, $^{1}J_{C\text{-}F}$ = 252.3, $^{2}J_{C\text{-}F}$ = 15.1 Hz,), 138.4, 137.6, 136.7 (dd, $^{2}J_{C\text{-}F}$ = 12.3, $^{3}J_{C\text{-}F}$ = 7.7 Hz), 127.7, 127.0, 110.9 (dd, $^{2}J_{C\text{-}F}$ = 15.9, $^{3}J_{C\text{-}F}$ = 5.9 Hz), 83.5, 24.8.

^{11}B NMR ($CDCl_3$, 128 MHz): δ 30.7.

^{19}F NMR ($CDCl_3$, 376 MHz): δ –134.1 (d, $J_{F\text{-}F}$ = 20.5 Hz, 2F), –162.4 (t, $J_{F\text{-}F}$ = 20.5 Hz, 1F).

HRMS: exact mass calculated for $[M+H]^+$ ($C_{20}H_{21}BF_3O_2$) requires *m/z* 361.1581, found *m/z* 361.1577.

trans-1-Methyl-4-(2-(4,4,5,5-tetramethyl-1,3,2-dioxaborolan-2-yl)vinyl)-1*H*-pyrazole, **3x**

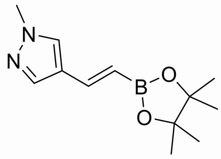

Prepared according to General Procedure A using *trans*-2-bromovinylboronic acid MIDA ester (65 mg, 0.25 mmol, 1 equiv), 1-methyl-4-(4,4,5,5-tetramethyl-1,3,2-dioxaborolan-2-yl)-1*H*-pyrazole (78 mg, 0.375 mmol, 1.5 equiv), Pd(dppf)Cl_2·CH_2Cl_2 (8.2 mg, 0.01 mmol, 4 mol%), K_3PO_4 (159 mg, 0.75 mmol, 3 equiv), THF (1 mL, 0.25 M), and H_2O (22.5 μL, 1.25 mmol, 5 equiv). After 24 h, the reaction mixture was subjected to purification *via* column chromatography on silica (10-40% EtOAc in petroleum ether) to afford the desired product as a beige solid (41 mg, 70%).

υ_{max} (film): 2978, 2927, 1626, 1373, 1344, 1325, 1143, 850 cm^{-1}.

^{1}H NMR ($CDCl_3$, 400 MHz): δ 7.42 (d, *J* = 1.9 Hz, 1H), 7.25 (d, *J* = 18.3 Hz, 1H), 6.50 (d, *J* = 1.9 Hz, 1H), 6.07 (d, *J* = 18.3 Hz, 1H), 3.94 (s, 3H), 1.33 (s, 12H).

^{13}C NMR ($CDCl_3$, 126 MHz): δ 141.3, 138.4, 134.5, 104.4, 83.6, 36.8, 24.8.

^{11}B NMR ($CDCl_3$, 128 MHz): δ 29.5.

HRMS: exact mass calculated for $[M+H]^+$ ($C_{12}H_{20}BN_2O_2$) requires *m/z* 235.1612, found *m/z* 235.1610.

Methyl 2-(4'-(4-(((benzyloxy)carbonyl)amino)pyridin-3-yl)-[1,1'-biphenyl]-4-yl)acetate, **7a**

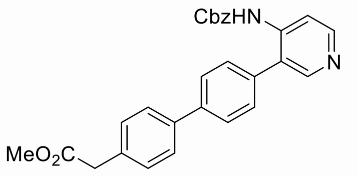

Prepared according to General Procedure C using 4-bromophenylboronic acid MIDA ester (78 mg, 0.25 mmol, 1 equiv), methyl 2-(4-(4,4,5,5-tetramethyl-1,3,2-dioxaborolan-2-yl)phenyl)acetate (104 mg, 0.375 mmol, 1.5 equiv), $Pd(dppf)Cl_2 \cdot CH_2Cl_2$ (8.2 mg, 0.01 mmol, 4 mol%), K_3PO_4 (212 mg, 1 mmol, 4 equiv), THF (1 mL, 0.25 M), H_2O (90 μL, 5 mmol, 20 equiv), and benzyl (3-bromopyridin-4-yl)carbamate (115 mg, 0.375 mmol, 1.5 equiv). After 48 h, the reaction mixture was subjected to purification by reverse phase preparative HPLC (20-95% MeCN in H_2O) to afford the desired product as a white solid (55 mg, 49%).

υ_{max} (film): 1732, 1498, 1193, 1155, 1139, 1049 cm^{-1}.

^1H NMR ($CDCl_3$, 400 MHz): δ 8.85 (d, J = 6.8 Hz, 1H), 8.68 (d, J = 6.8 Hz, 1H), 8.64 (s, 1H), 7.81 (d, J = 8.1 Hz, 2H), 7.61 (d, J = 8.1 Hz, 2H), 7.54 (s, 1H), 7.39-7.46 (m, 8H), 5.26 (s, 2H), 3.75 (s, 3H), 3.72 (s, 2H).

^{13}C NMR ($CDCl_3$, 126 MHz): δ 171.9, 161.5, 161.1, 151.6, 150.2, 143.4, 141.5, 138.2, 134.3, 134.1, 130.1, 129.4, 129.2, 129.1, 129.0, 128.9, 128.4, 127.6, 127.4, 113.3, 69.1, 52.2, 40.8.

HRMS: exact mass calculated for $[M+H]^+$ ($C_{28}H_{25}N_2O_4$) requires m/z 453.1809, found m/z 453.1804.

3'-(1-Methyl-1*H*-pyrazol-4-yl)-[1,1'-biphenyl]-4-carbonitrile, **7b**

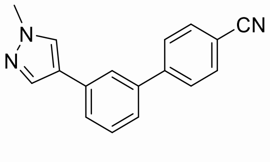

Prepared according to General Procedure C using 3-bromophenylboronic acid MIDA ester (78 mg, 0.25 mmol, 1 equiv), 1-methyl-4-(4,4,5,5-tetramethyl-1,3,2-dioxaborolan-2-yl)-1*H*-pyrazole (78 mg, 0.375 mmol, 1.5 equiv), $Pd(dppf)Cl_2 \cdot CH_2Cl_2$ (8.2 mg, 0.01 mmol, 4 mol%), K_3PO_4 (212 mg, 1 mmol, 4 equiv), THF (1 mL, 0.25 M), H_2O (90 μL, 5 mmol, 20 equiv),

and 4-bromobenzonitrile (68 mg, 0.375 mmol, 1.5 equiv). After 48 h, the reaction mixture was subjected to the purification outlined in the General Procedure to afford the desired product as a white solid (40 mg, 63%).

υ_{max} (film): 2226, 1452, 1369, 1174, 1143, 1107, 844, 783, 700 cm^{-1}.

^{1}H NMR ($CDCl_3$, 400 MHz): δ 7.76 (dd, J =22.8, 8.4 Hz, 4H), 7.49-7.69 (m, 5H), 6.43 (s, 1H), 3.98 (s, 3H).

^{13}C NMR ($CDCl_3$, 126 MHz): δ 144.7, 140.0, 132.8, 131.1, 129.6, 128.9, 127.8, 127.7, 127.6, 118.7, 111.6, 106.6, 37.2.

HRMS: exact mass calculated for $[M+H]^+$ ($C_{17}H_{14}N_3$) requires *m/z* 260.1182, found *m/z* 260.1183.

Methyl 2-(3''-(4,4,5,5-tetramethyl-1,3,2-dioxaborolan-2-yl)-[1,1':3',1''-terphenyl]-4-yl)acetate, **8**

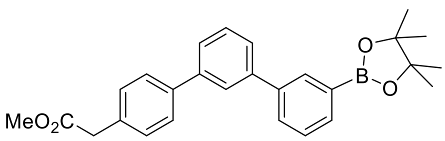

Prepared according to General Procedure C using 3-bromophenylboronic acid MIDA ester (78 mg, 0.25 mmol, 1 equiv), methyl 2-(4-(4,4,5,5-tetramethyl-1,3,2-dioxaborolan-2-yl)phenyl)acetate (104 mg, 0.375 mmol, 1.5 equiv), $Pd(dppf)Cl_2·CH_2Cl_2$ (8.2 mg, 0.01 mmol, 4 mol%), K_3PO_4 (212 mg, 1 mmol, 4 equiv), THF (1 mL, 0.25 M), H_2O (90 µL, 5 mmol, 20 equiv), and 3-bromophenylboronic acid MIDA ester (78 mg, 0.25 mmol, 1 equiv), After 48 h, the reaction mixture was subjected to the purification outlined in the General Procedure to afford the desired product as an off-white solid (52 mg, 49%).

υ_{max} (film): 2924, 1735, 1431, 1357, 1257, 1143, 707 cm^{-1}.

^{1}H NMR ($CDCl_3$, 400 MHz): δ 8.06 (s, 1H), 7.81 (dt, J = 7.2, 1.1 Hz, 1H), 7.68-7.71 (m, 2H), 7.62 (d, J = 8.3 Hz, 3H), 7.47 (t, J = 7.7 Hz, 2H), 7.36-7.39 (m, 3H), 3.74 (s, 3H), 3.70 (s, 2H), 1.39 (s, 12H).

^{13}C NMR ($CDCl_3$, 126 MHz): δ 172.0, 140.1, 133.7, 133.4, 132.9, 132.4, 129.9, 129.8, 129.7, 129.6, 128.8, 127.5, 127.4, 127.2, 126.0, 83.9, 52.1, 40.9, 24.9.

^{11}B NMR ($CDCl_3$, 128 MHz): δ 31.1.

HRMS: exact mass calculated for $[M+H]^+$ ($C_{27}H_{30}BO_4$) requires *m/z* 429.2232, found *m/z* 429.2227.

2-(4'-(3,6-Dihydro-2*H*-pyran-4-yl)-2'-fluoro-[1,1'-biphenyl]-3-yl)-4,4,5,5-tetramethyl-1,3,2-dioxaborolane, **9**

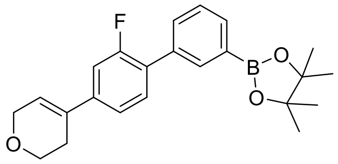

Prepared according to General Procedure C using 4-bromo-2-fluorophenylboronic acid MIDA ester (82 mg, 0.25 mmol, 1 equiv), 2-(3,6-dihydro-2*H*-pyran-4-yl)-4,4,5,5-tetramethyl-1,3,2-dioxaborolane (79 mg, 0.375 mmol, 1.5 equiv), $Pd(dppf)Cl_2·CH_2Cl_2$ (8.2 mg, 0.01 mmol, 4 mol%), K_3PO_4 (212 mg, 1 mmol, 4 equiv), THF (1 mL, 0.25 M), H_2O (90 µL, 5 mmol, 20 equiv), and 3-bromophenylboronic acid MIDA ester (78 mg, 0.25 mmol, 1 equiv), After 48 h, the reaction mixture was subjected to the purification outlined in the General Procedure to afford the desired product as an brown oil (67 mg, 70%).

$υ_{max}$ (film): 2978, 2927, 1355, 1141, 1130, 906, 729, 705, 677 cm^{-1}.

^1H NMR ($CDCl_3$, 400 MHz): δ 7.98 (d, *J* = 1.0 Hz, 1H), 7.82 (dt, *J* = 7.3, 1.1 Hz, 1H), 7.67 (ddd, *J* = 7.7, 3.2, 1.8 Hz, 1H), 7.45 (td, *J* = 7.9, 3.0 Hz, 2H), 7.24 (dd, *J* = 8.1, 1.8 Hz, 1H), 7.17 (dd, *J* = 12.3, 1.7 Hz, 1H), 6.19 – 6.23 (m, 1H), 4.38 (dd, *J* = 5.5, 2.7 Hz, 2H), 3.99 (t, *J* = 5.5 Hz, 2H), 2.52 – 2.57 (m, 1H), 1.36 (s, 12H).

^{13}C NMR ($CDCl_3$, 126 MHz): δ 159.9 (d, $^1J_{C-F}$ = 247.4 Hz), 141.2 (d, $^3J_{C-F}$ = 7.5 Hz), 135.1, 134.9, 134.1, 132.9, 131.9 (d, $^3J_{C-F}$ = 3.3 Hz), 130.7, 127.8, 127.7 (d, $^2J_{C-F}$ = 13.8 Hz), 123.1, 120.4 (d, $^3J_{C-F}$ = 3.3 Hz), 112.2 (d, $^2J_{C-F}$ = 24.1 Hz), 84.0, 65.7, 64.4, 27.0, 24.9.

^{11}B NMR ($CDCl_3$, 128 MHz): δ 31.9.

^{19}F NMR ($CDCl_3$, 376 MHz): δ – 118.24.

HRMS: exact mass calculated for $[M+NH_4]^+$ ($C_{23}H_{29}BFNO_3$) requires *m/z* 398.2298, found *m/z* 398.2298.

5. References

1. W. L. F. Armarego, C. Chai, *Purification of Laboratory Chemicals, 7th ed.*, Elsevier, Oxford, 2013.

6. NMR and HRMS spectra for intermediates and products

^{1}H NMR of S1

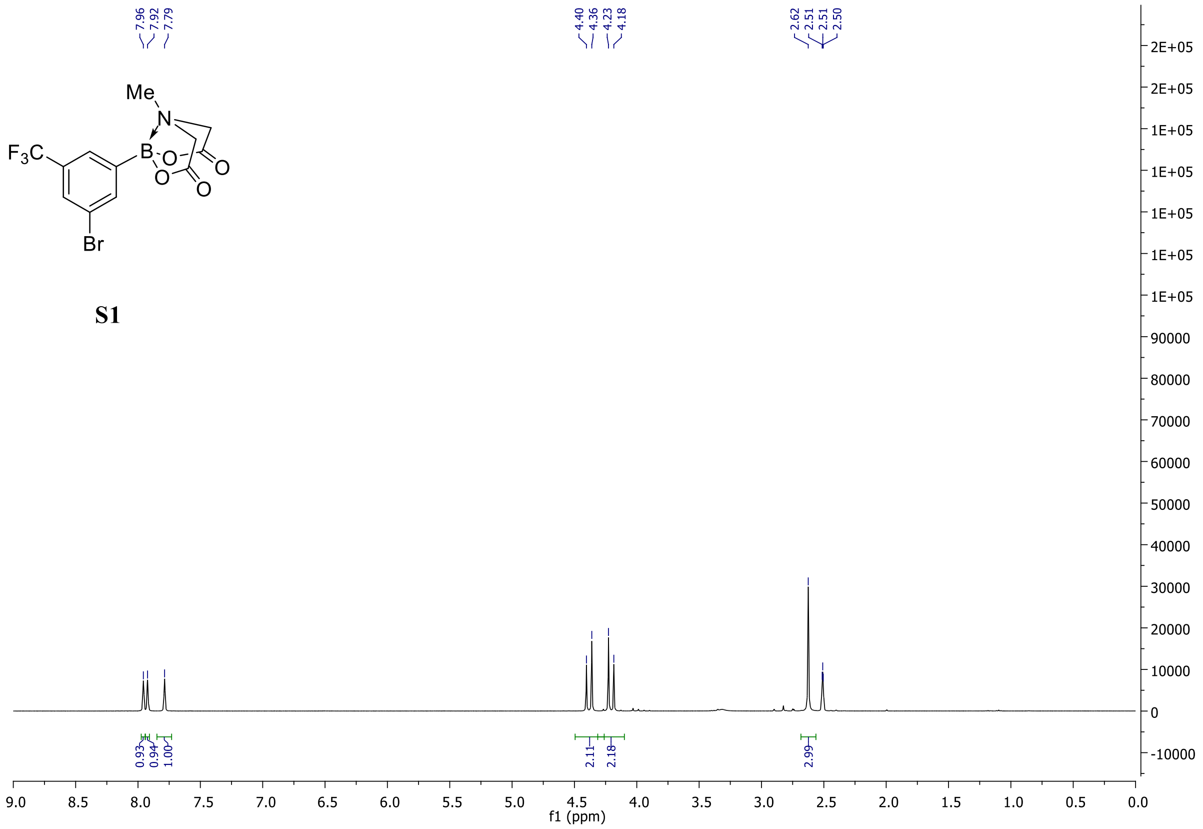

^{13}C NMR of S1

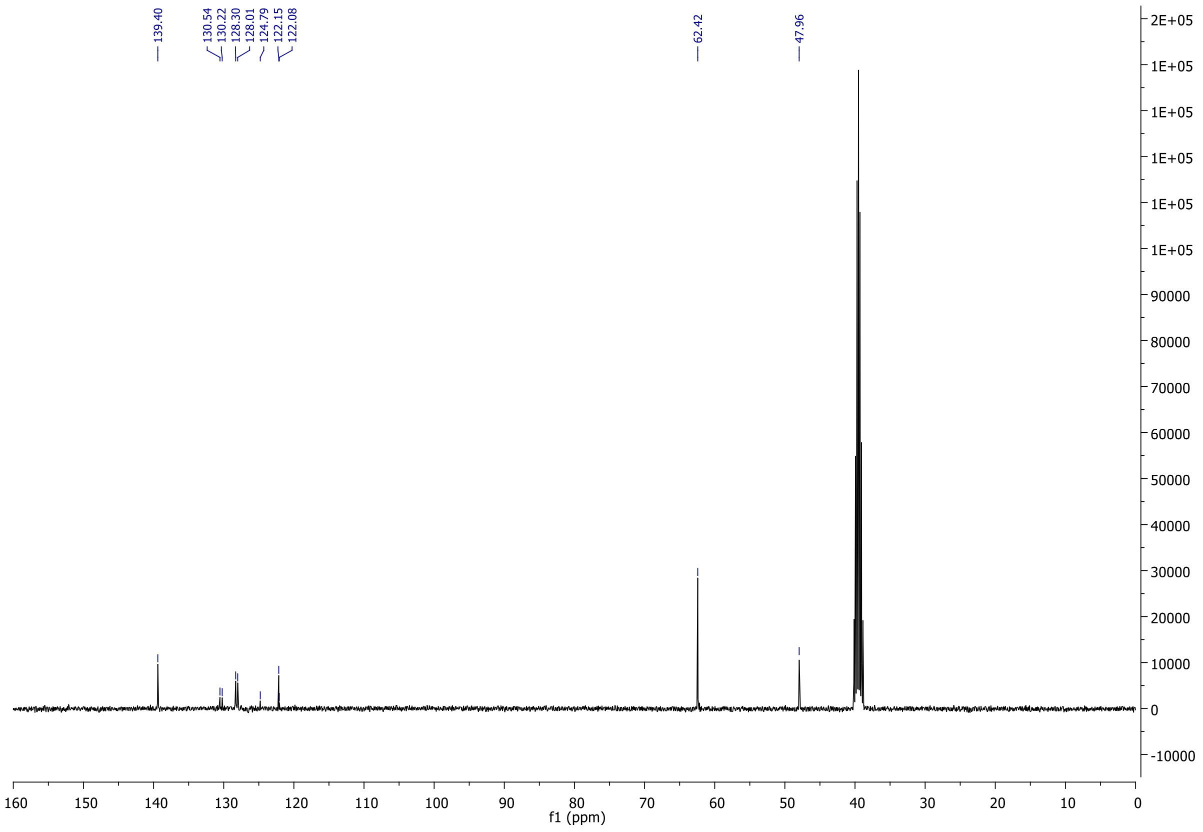

^{11}B NMR of S1

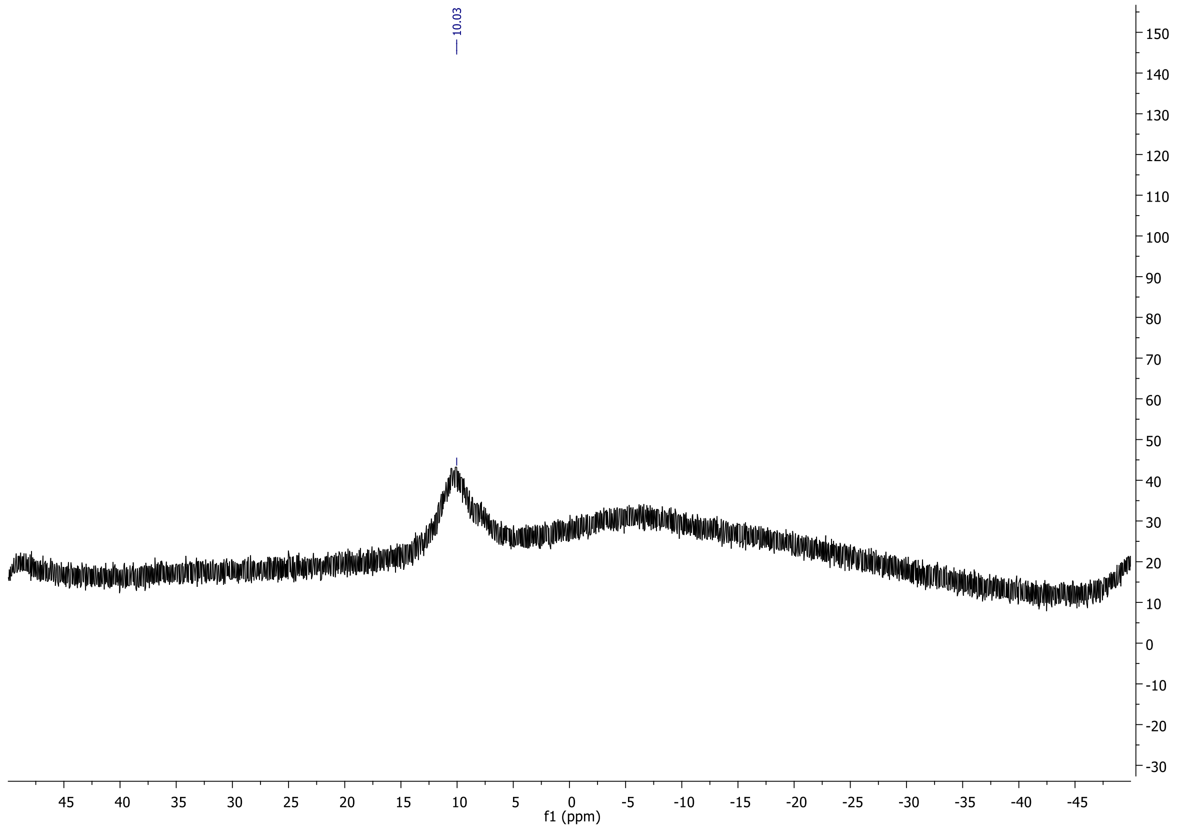

^{19}F NMR of S1

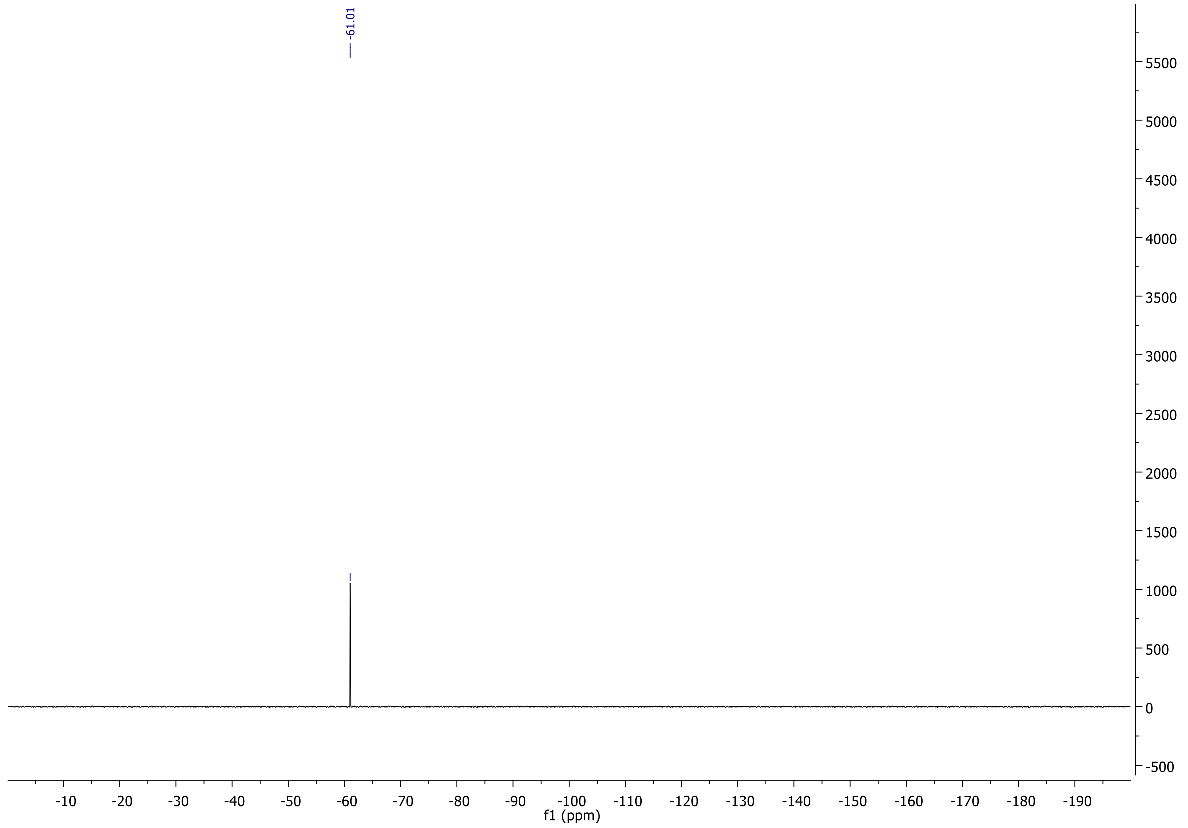

HRMS of S1

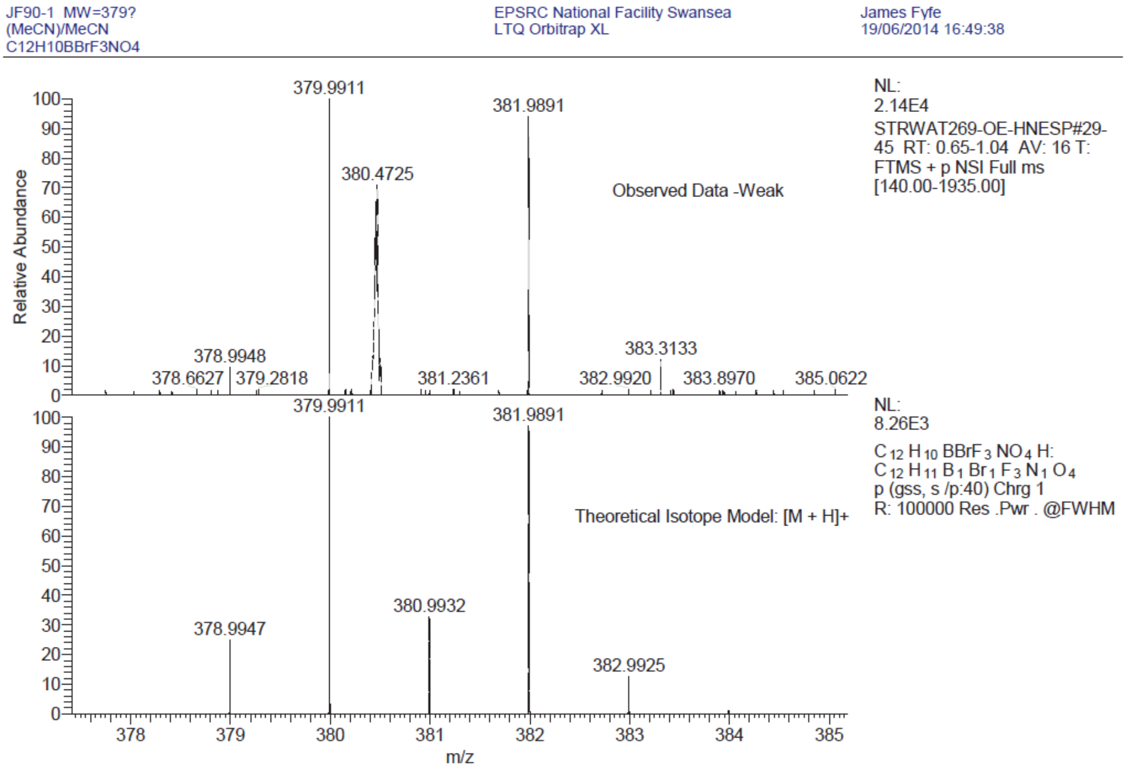

^{1}H NMR of S2

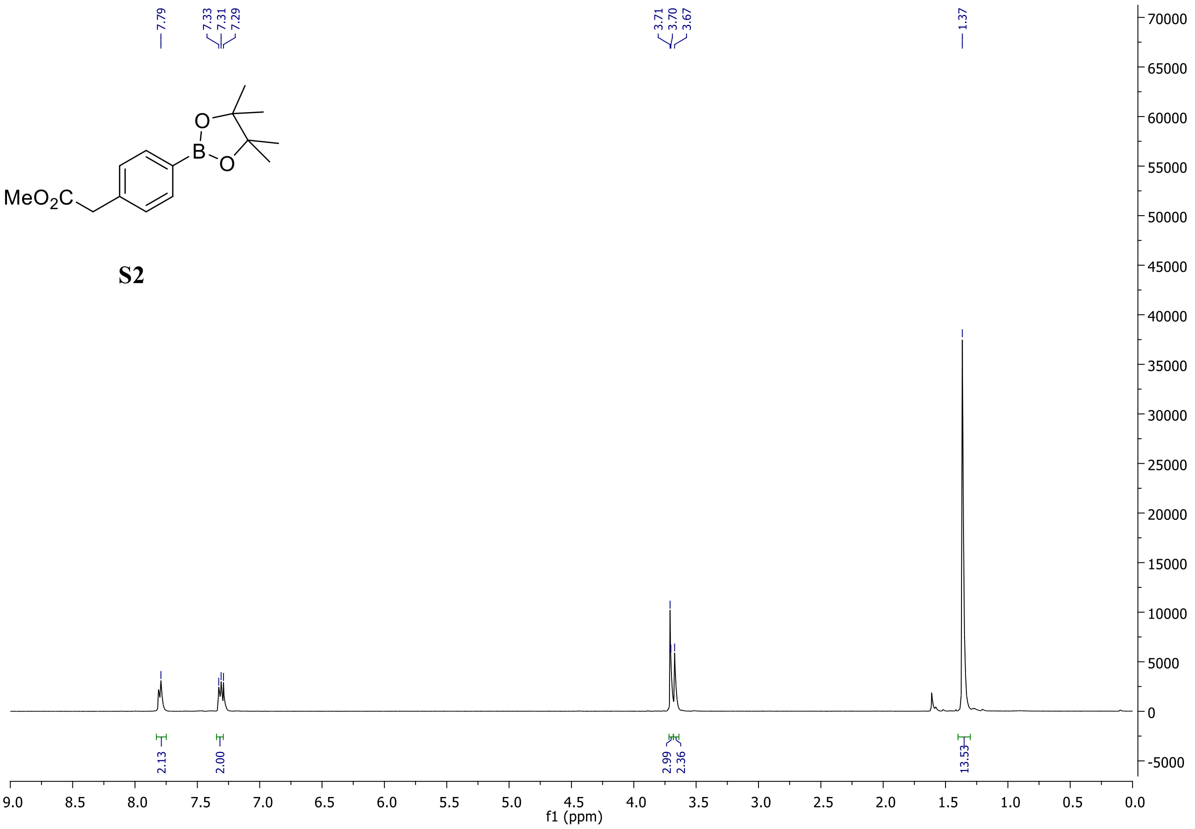

^{13}C NMR of S2

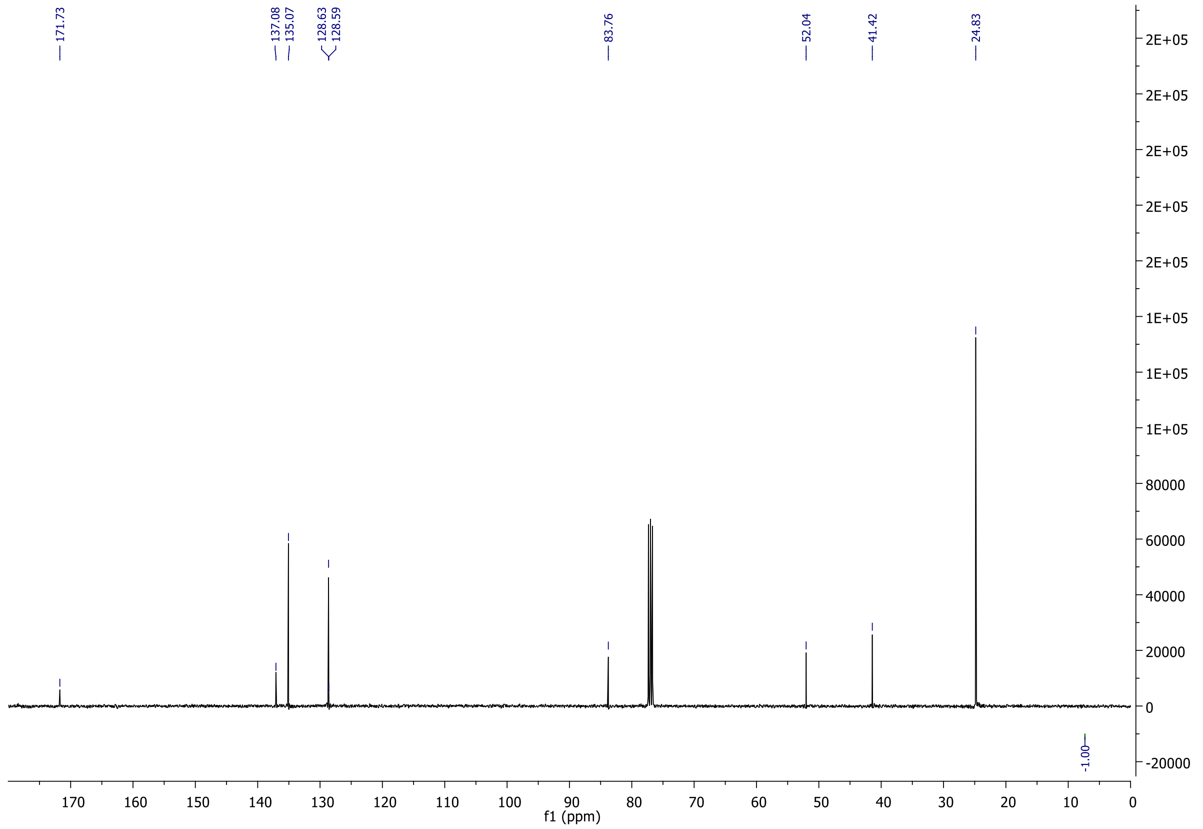

^{11}B NMR of S2

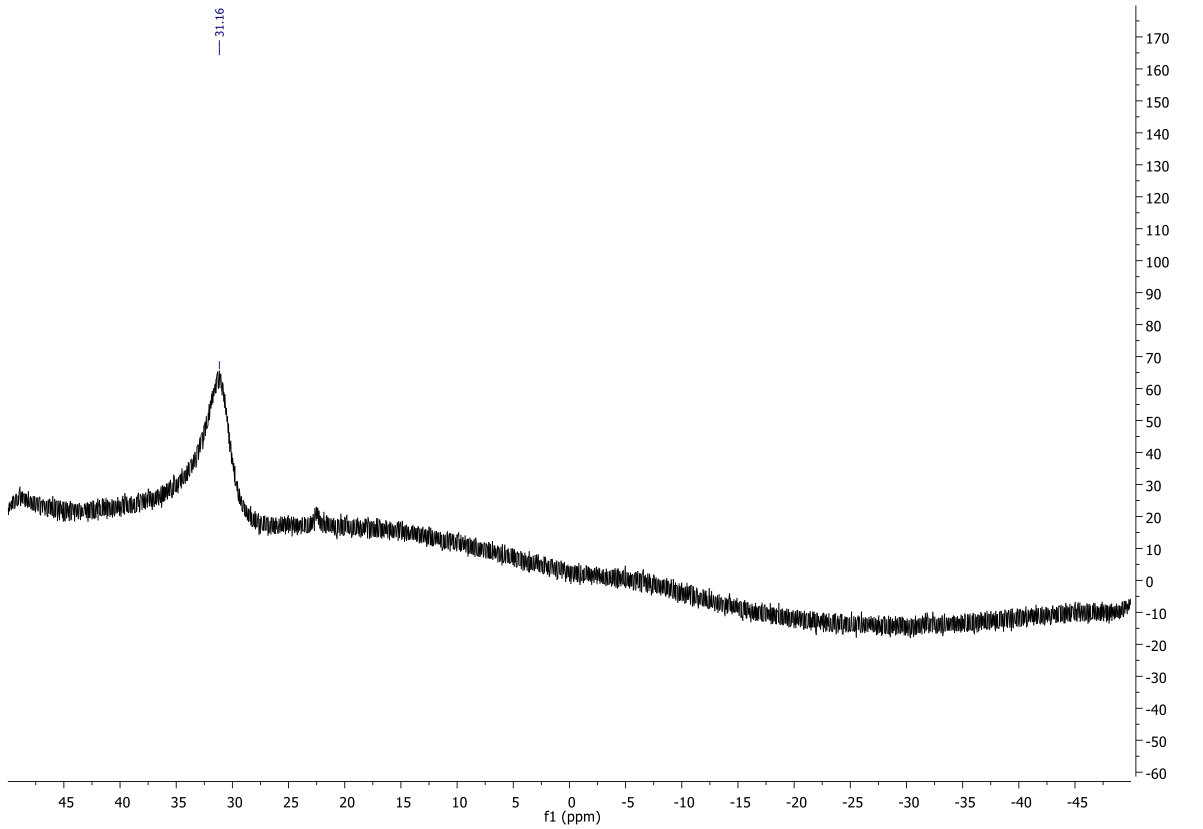

HRMS of S2

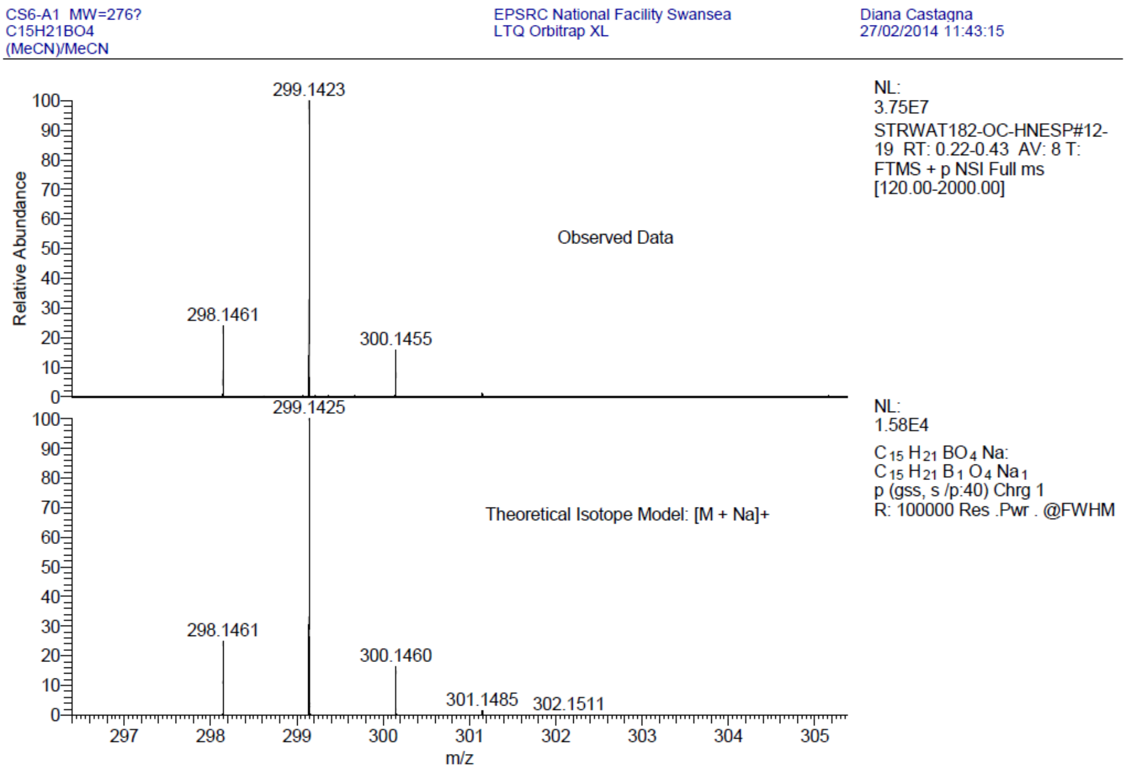

^{1}H NMR of S3

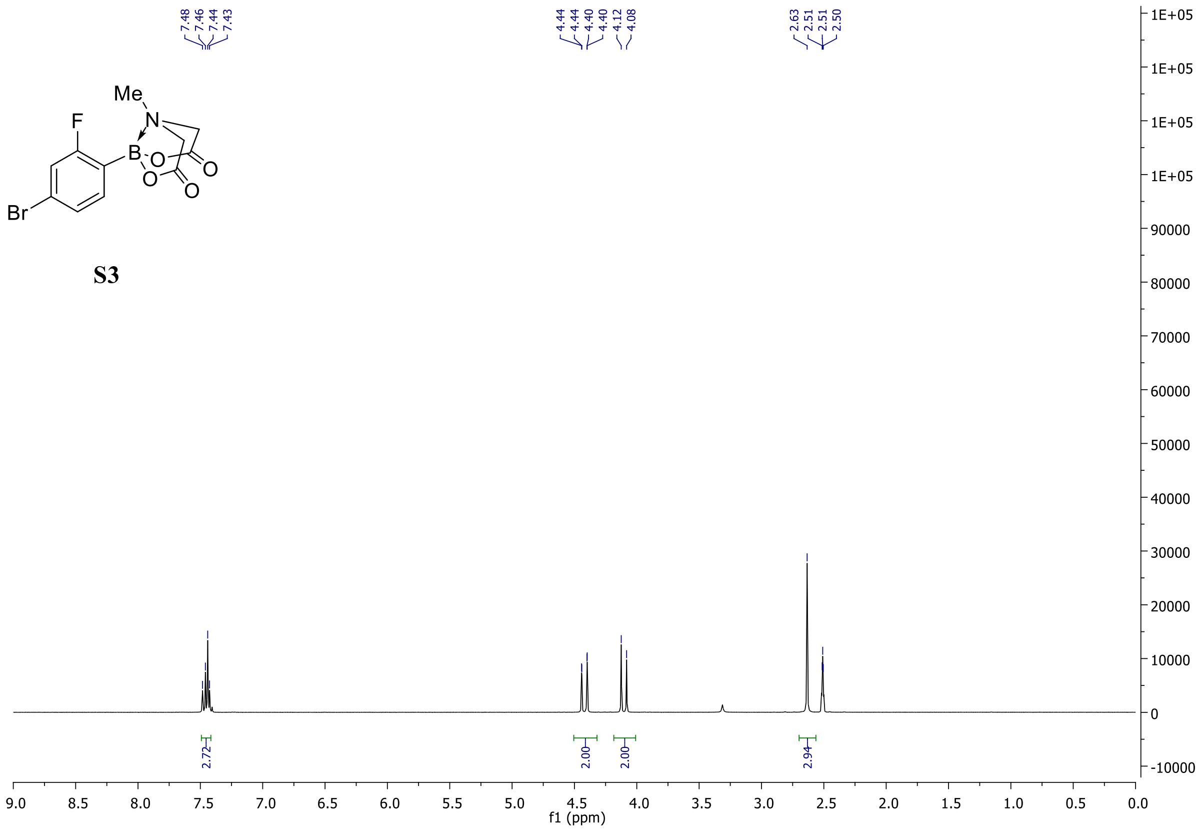

^{13}C NMR of S3

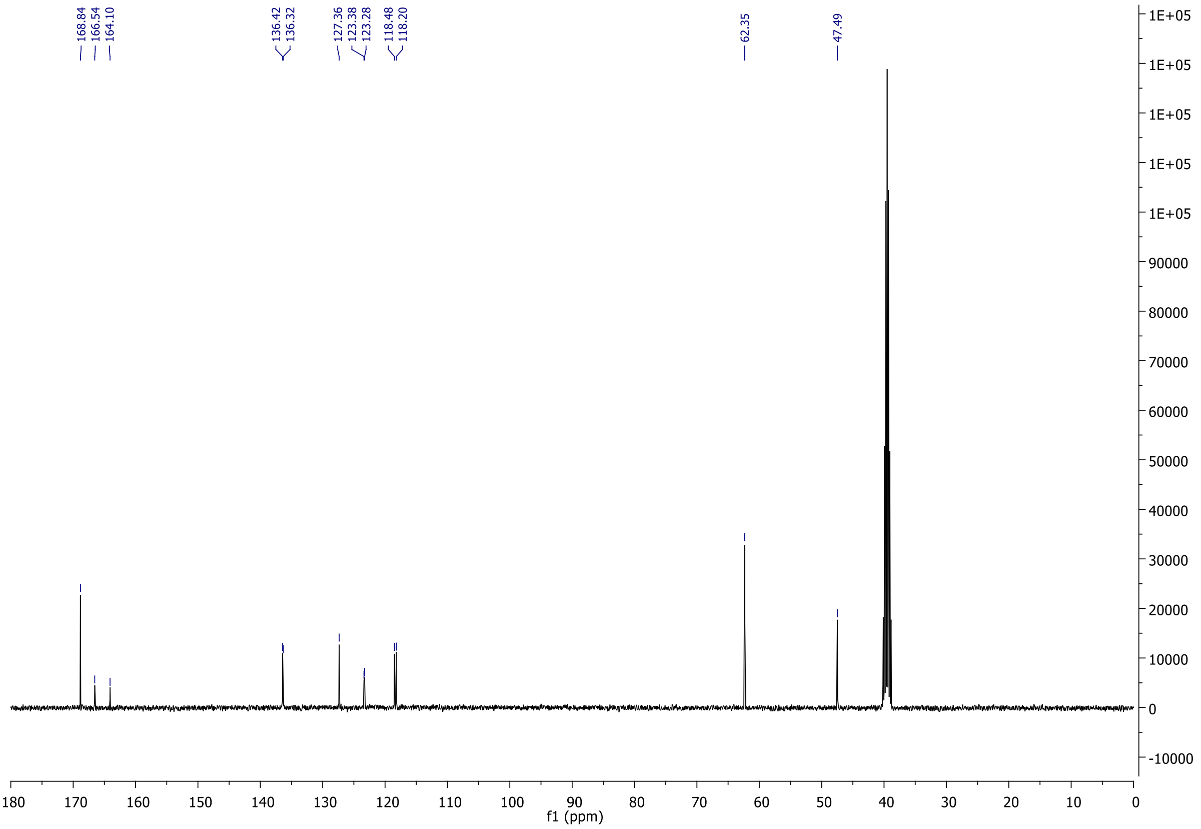

^{11}B NMR of S3

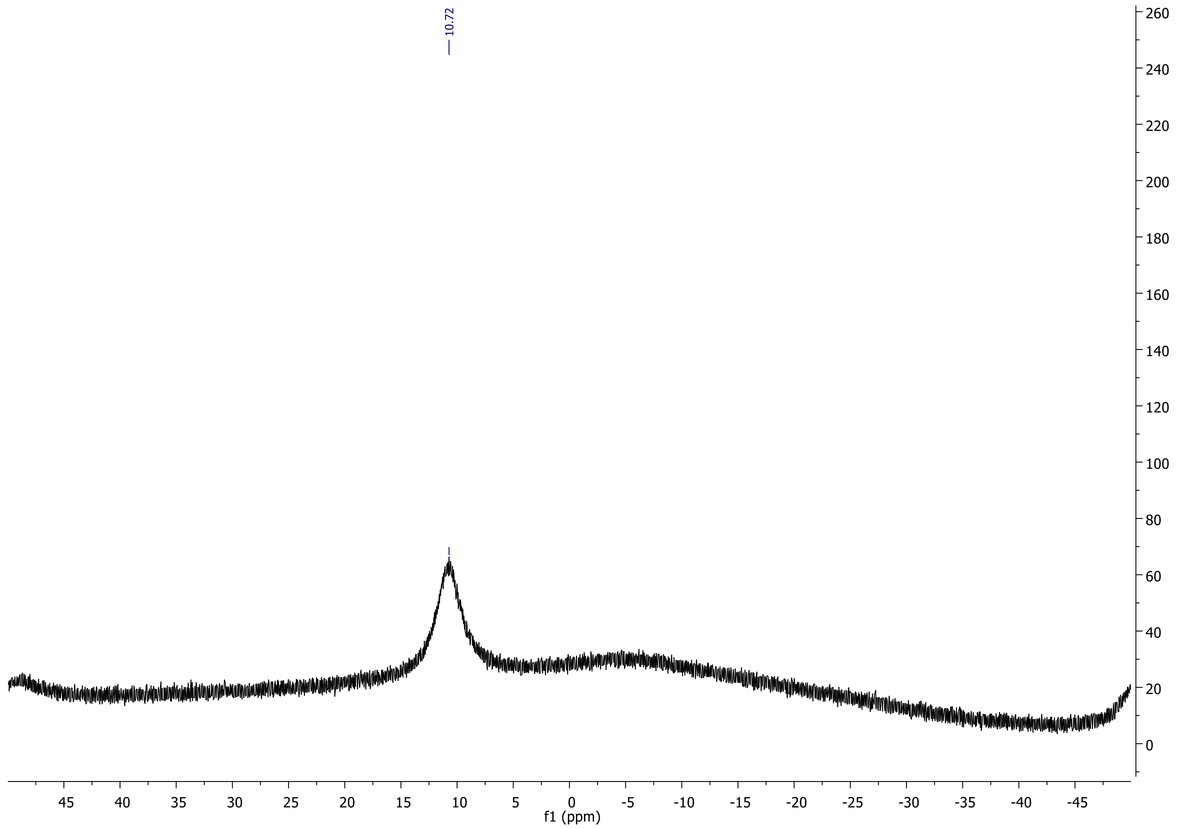

^{19}F NMR of S3

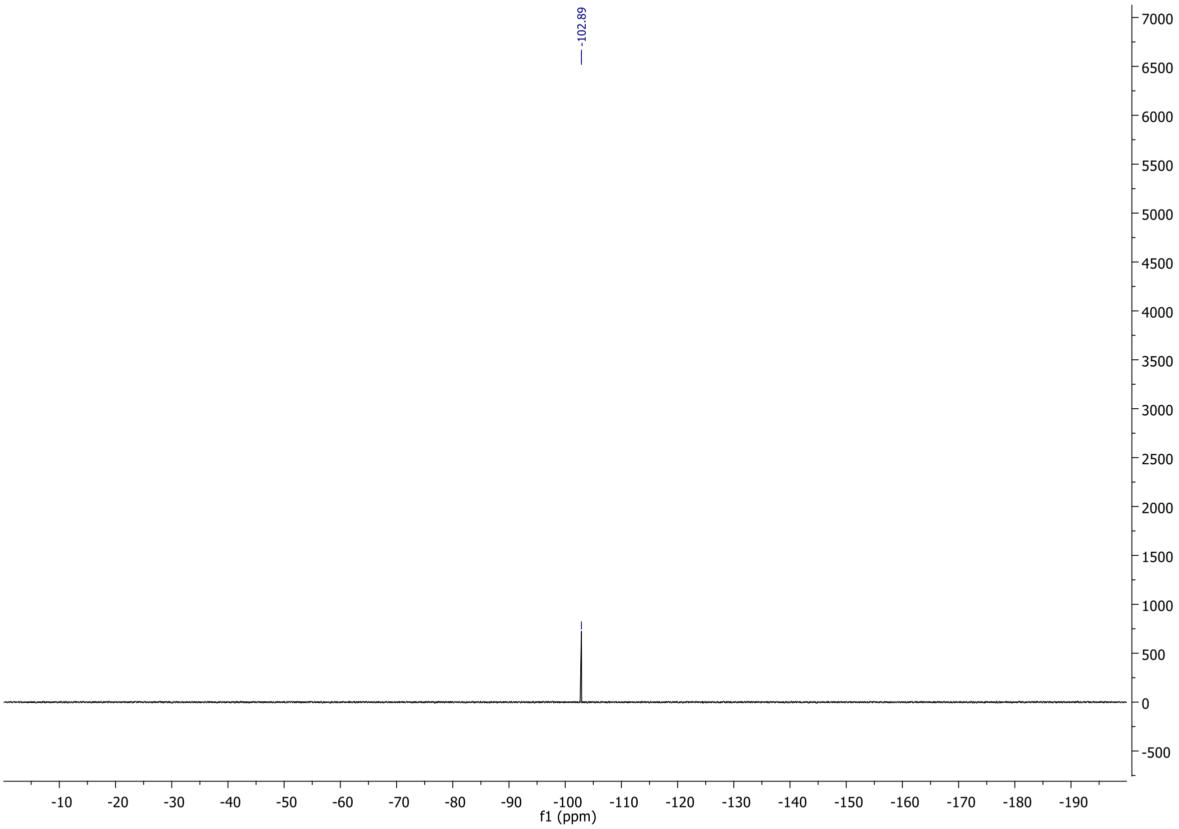

HRMS of S3

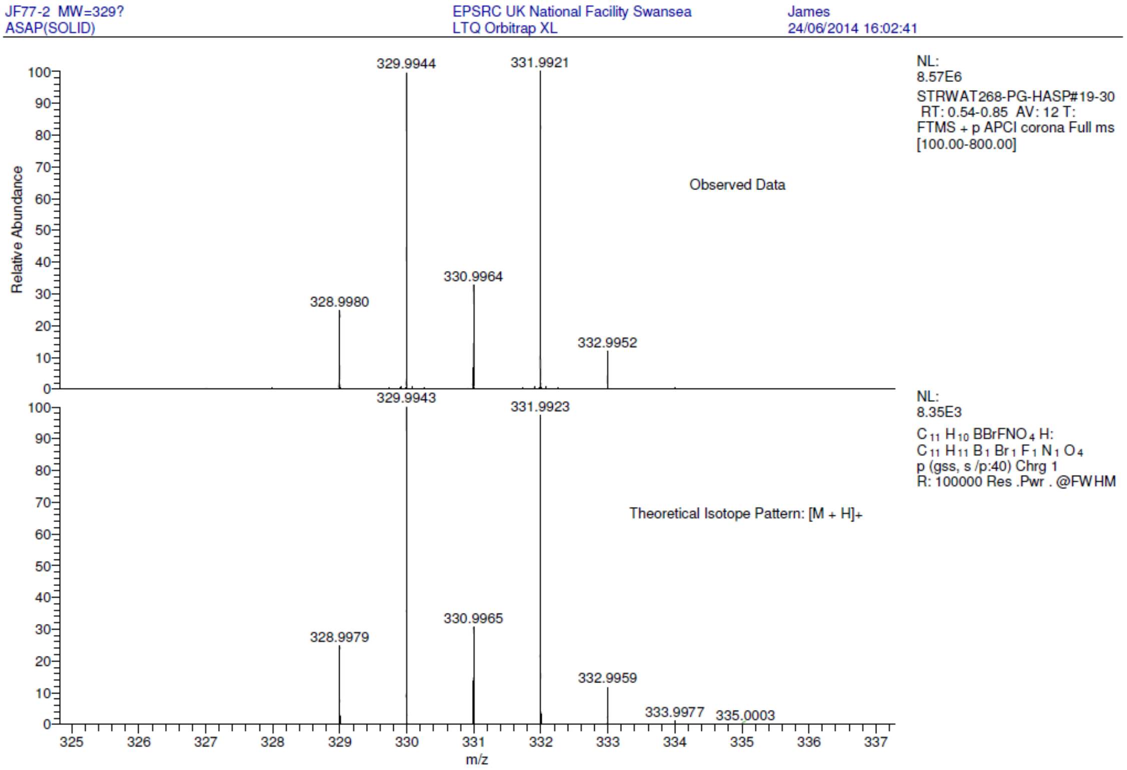

^{1}H NMR of S4

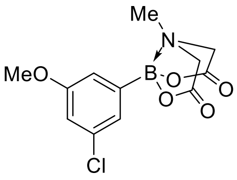

S4

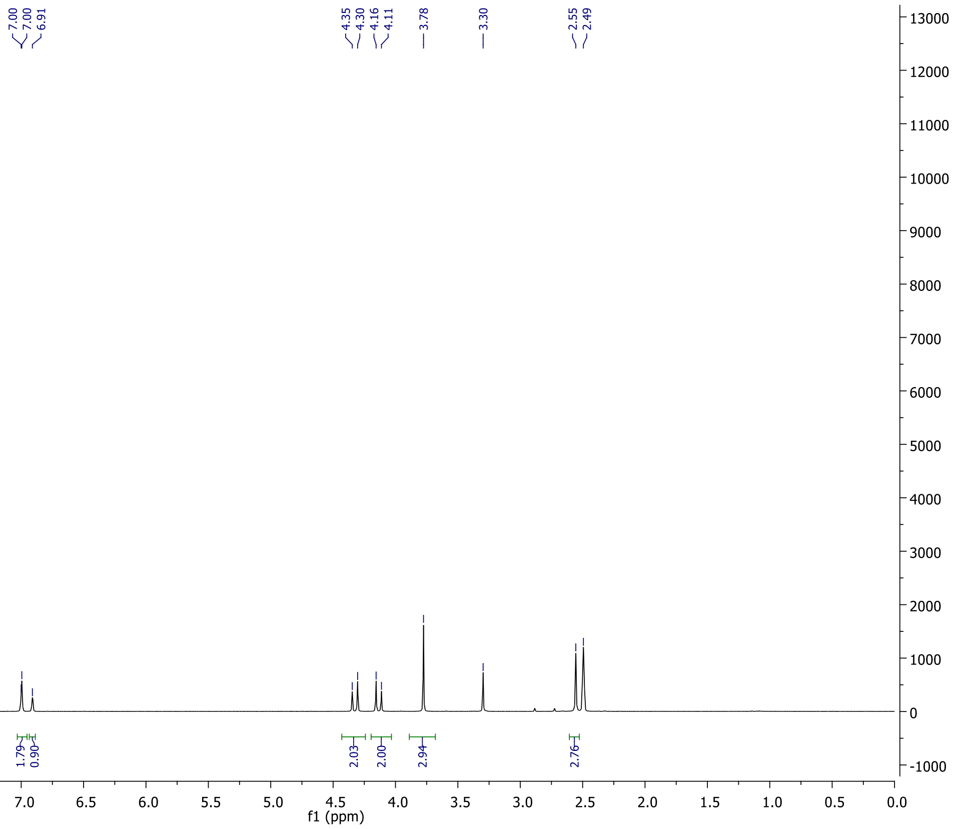

^{13}C NMR of S4

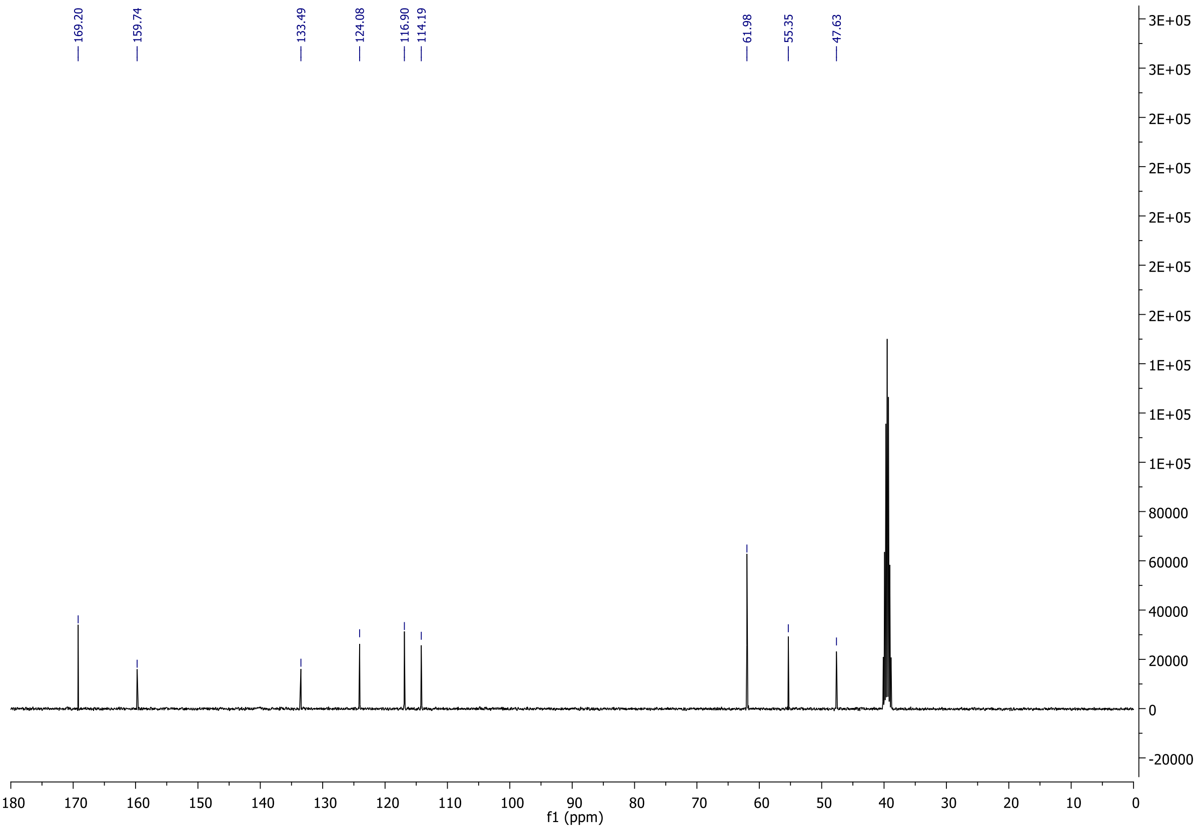

^{11}B NMR of S4

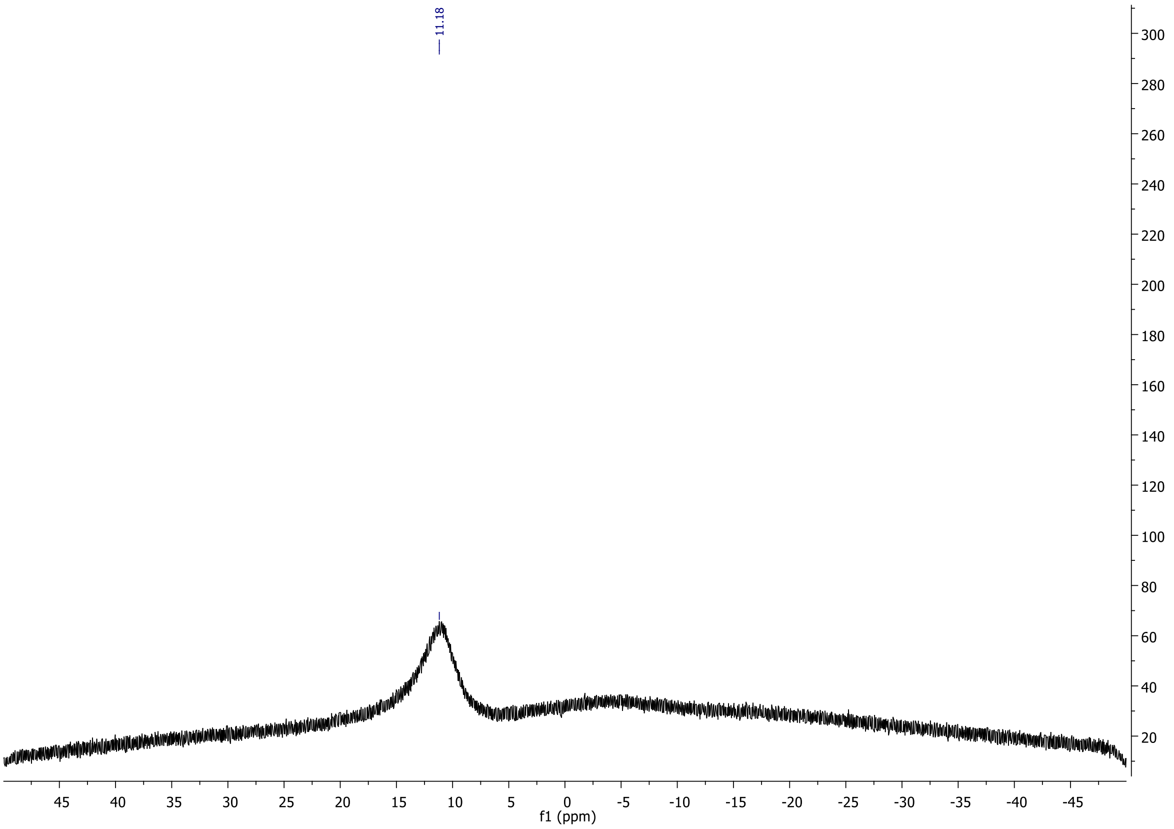

HRMS of S4

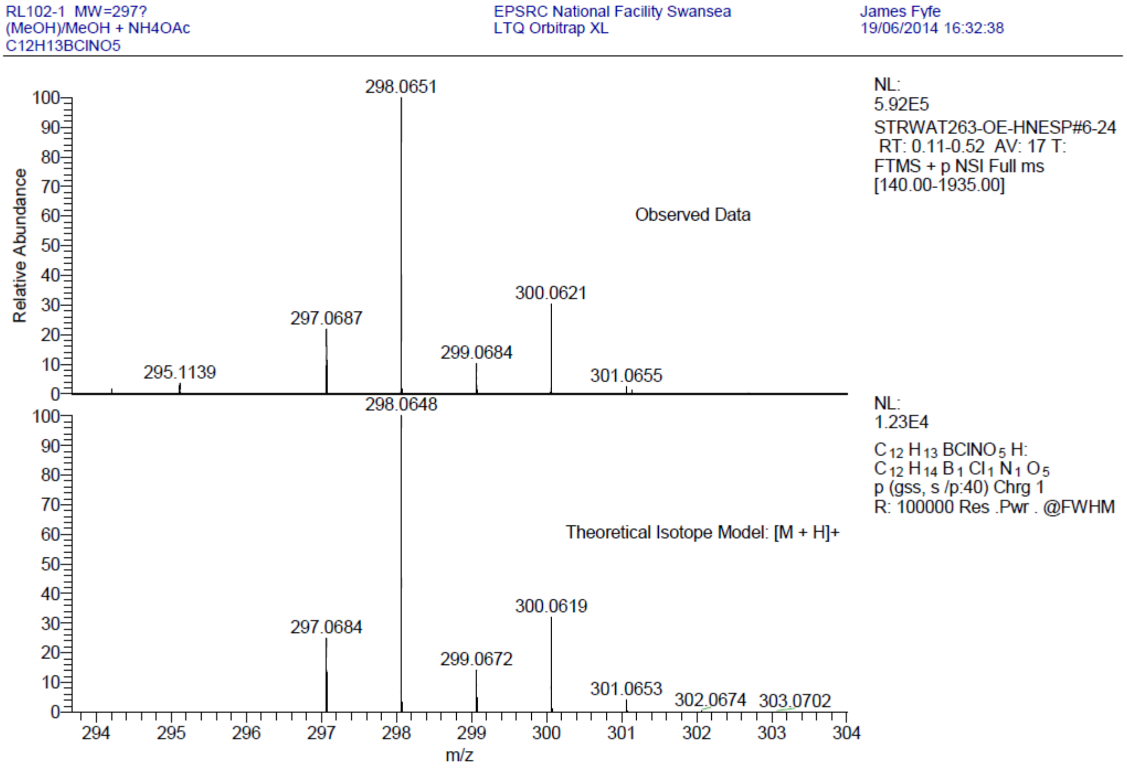

^{1}H NMR of S5

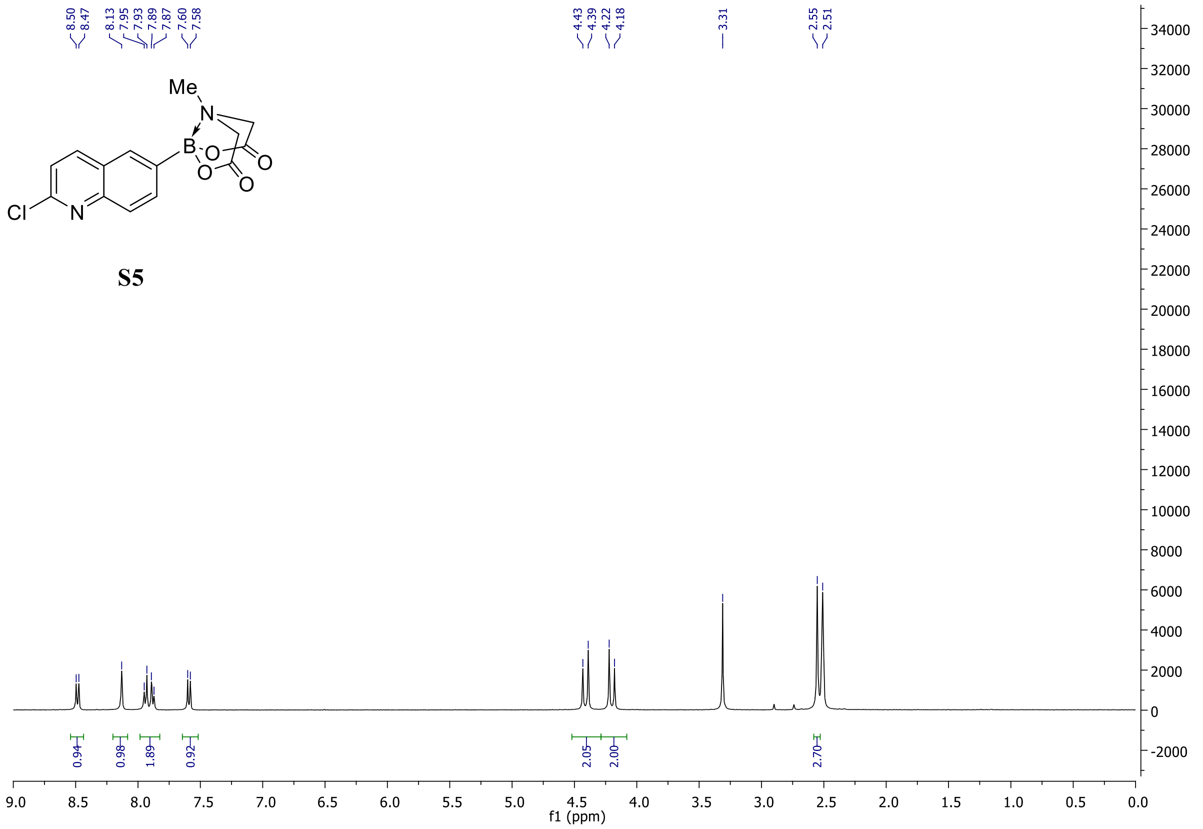

^{13}C NMR of S5

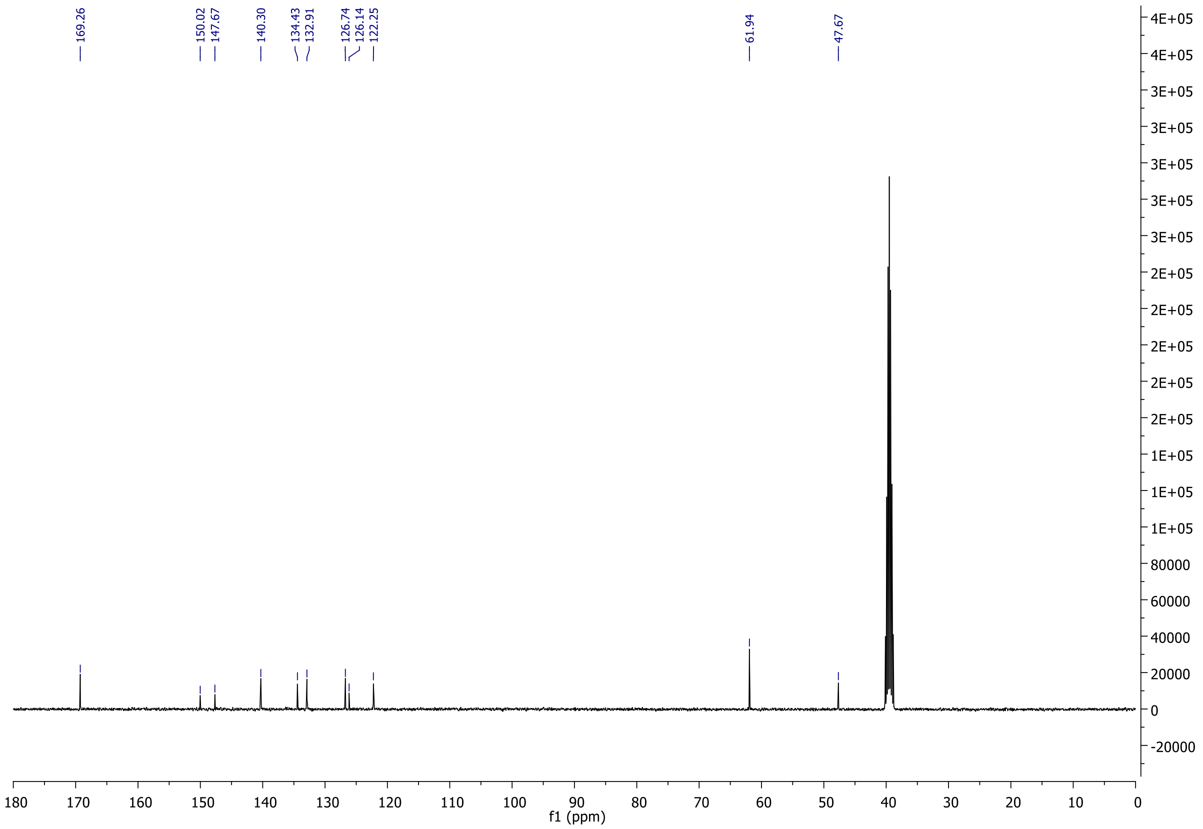

^{11}B NMR of S5

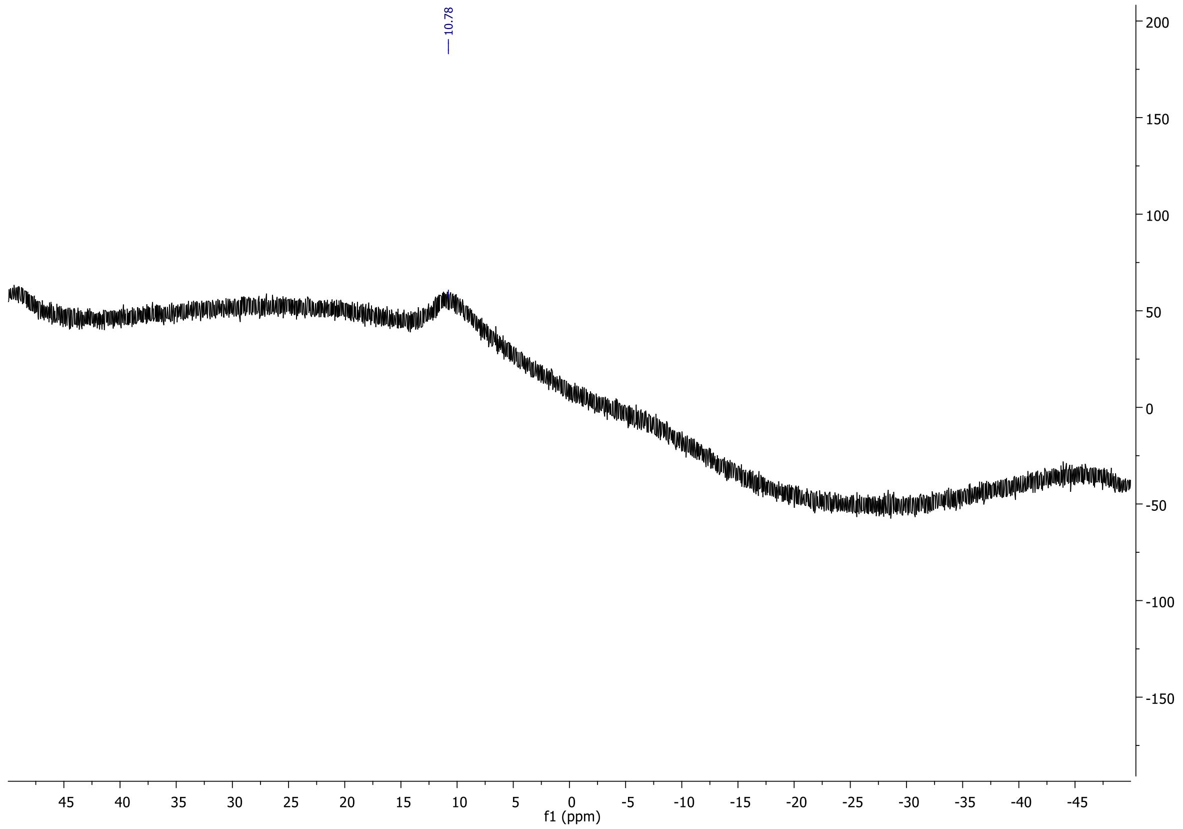

HRMS of S5

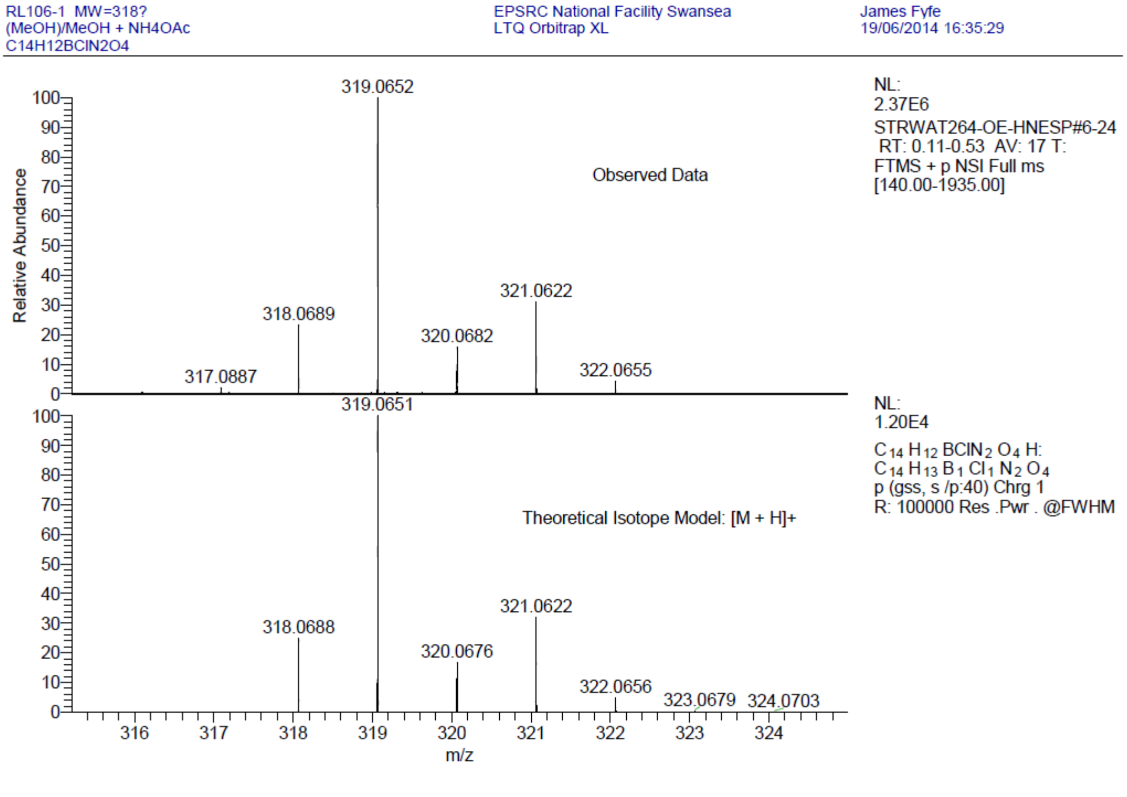

^{1}H NMR of S6

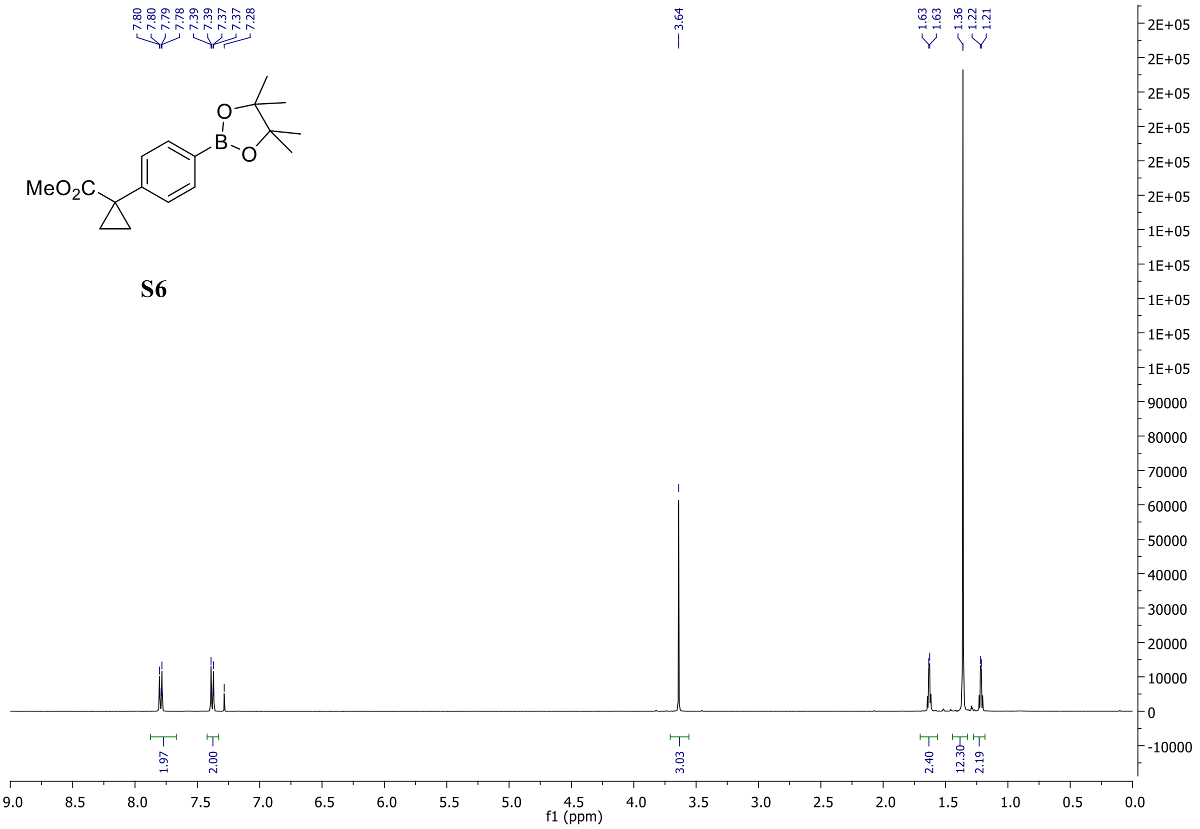

^{13}C NMR of S6

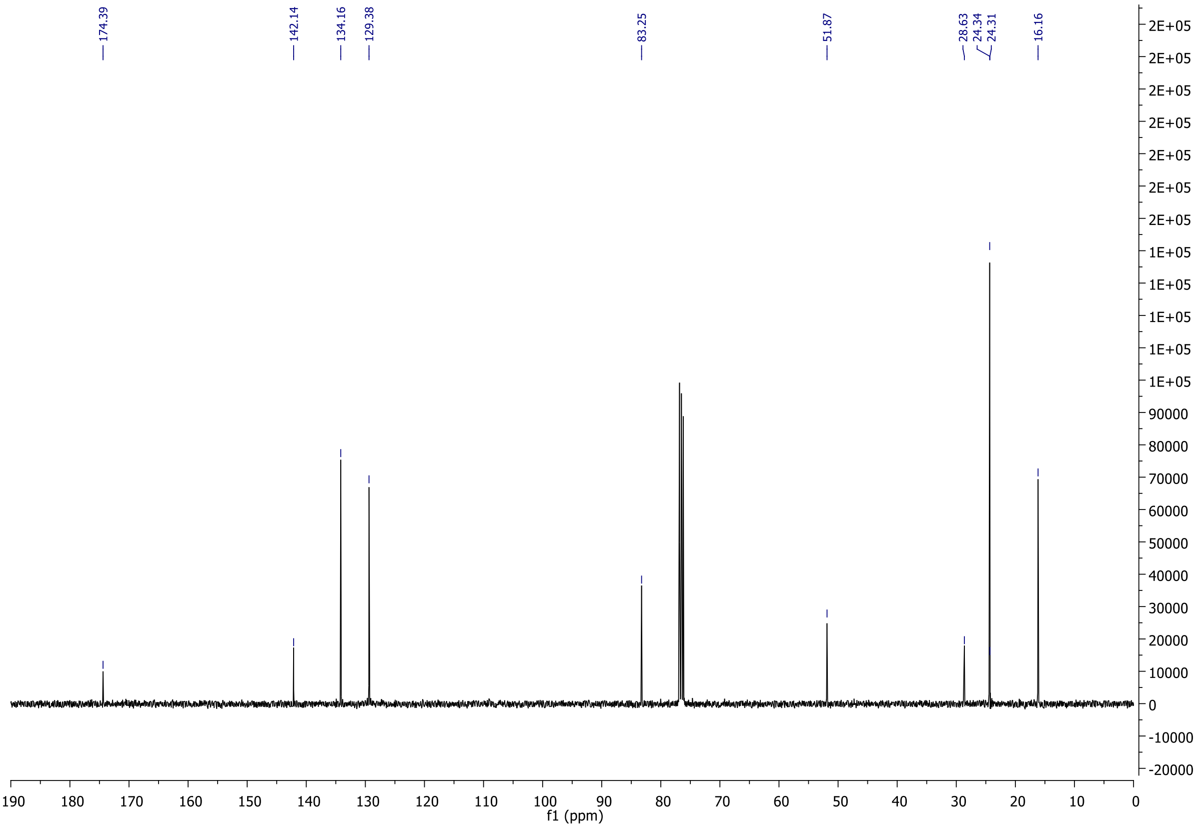

^{11}B NMR of S6

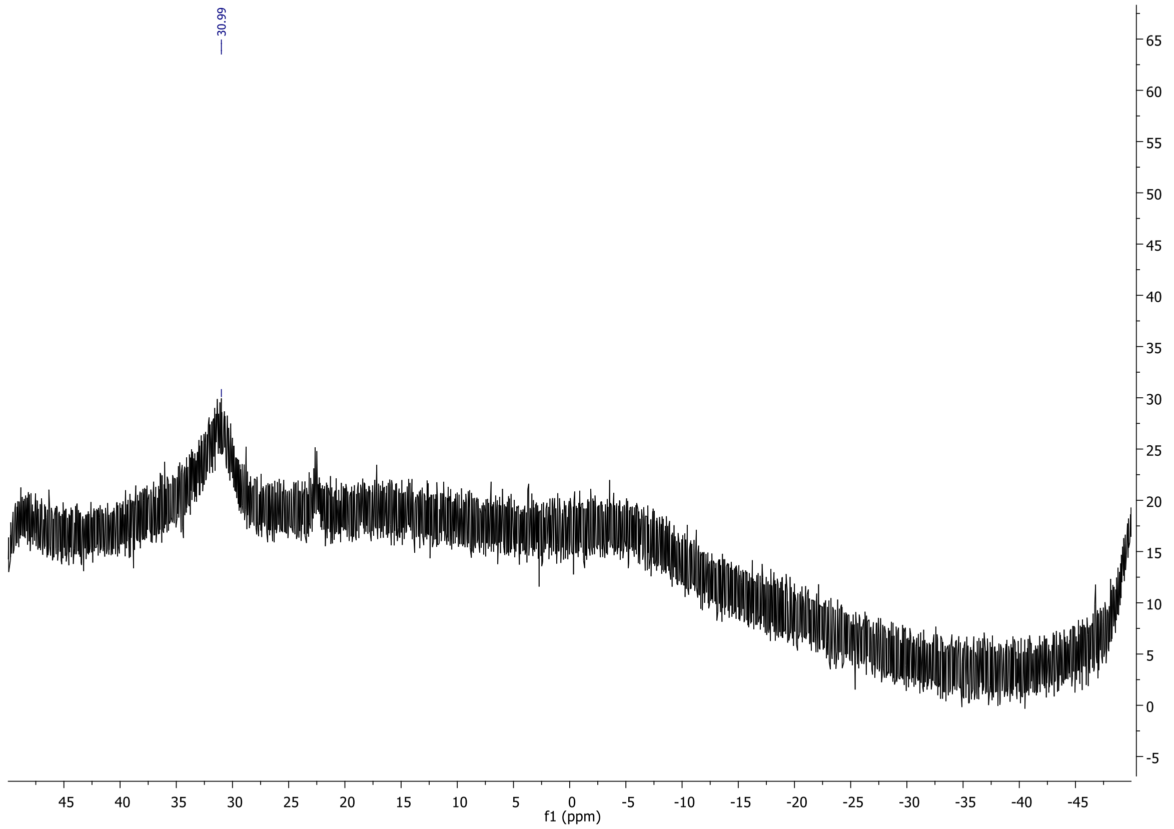

HRMS of S6

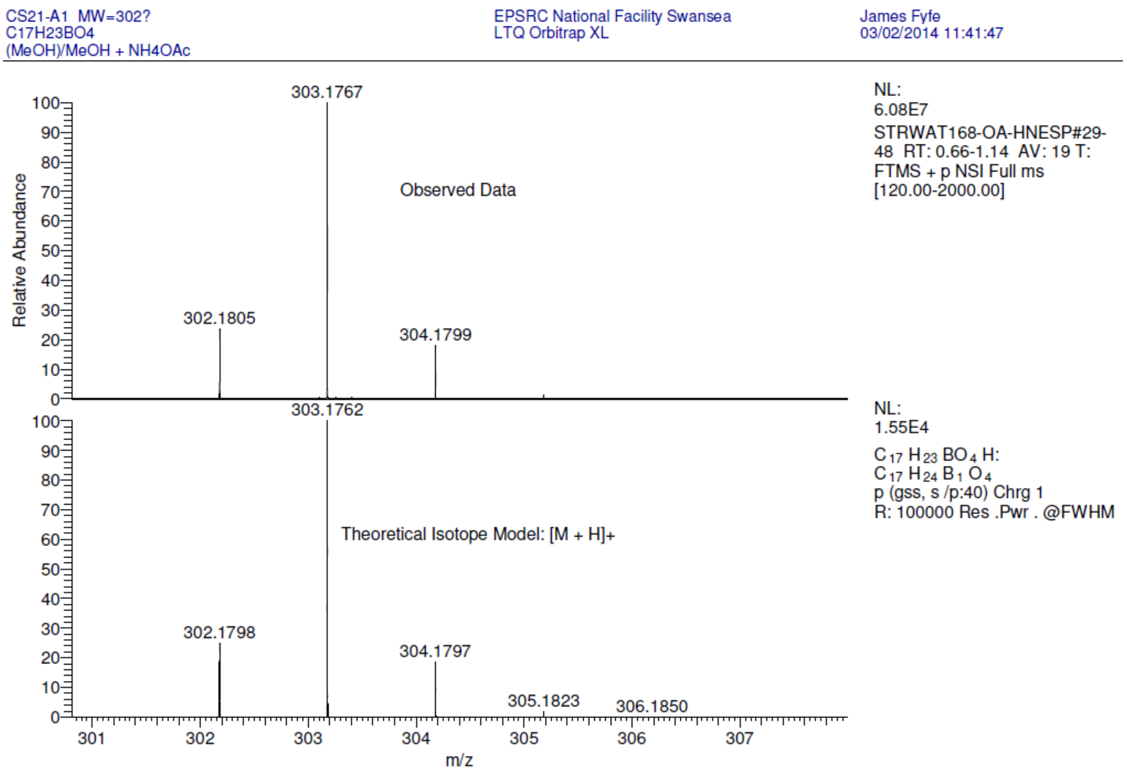

^{1}H NMR of S7

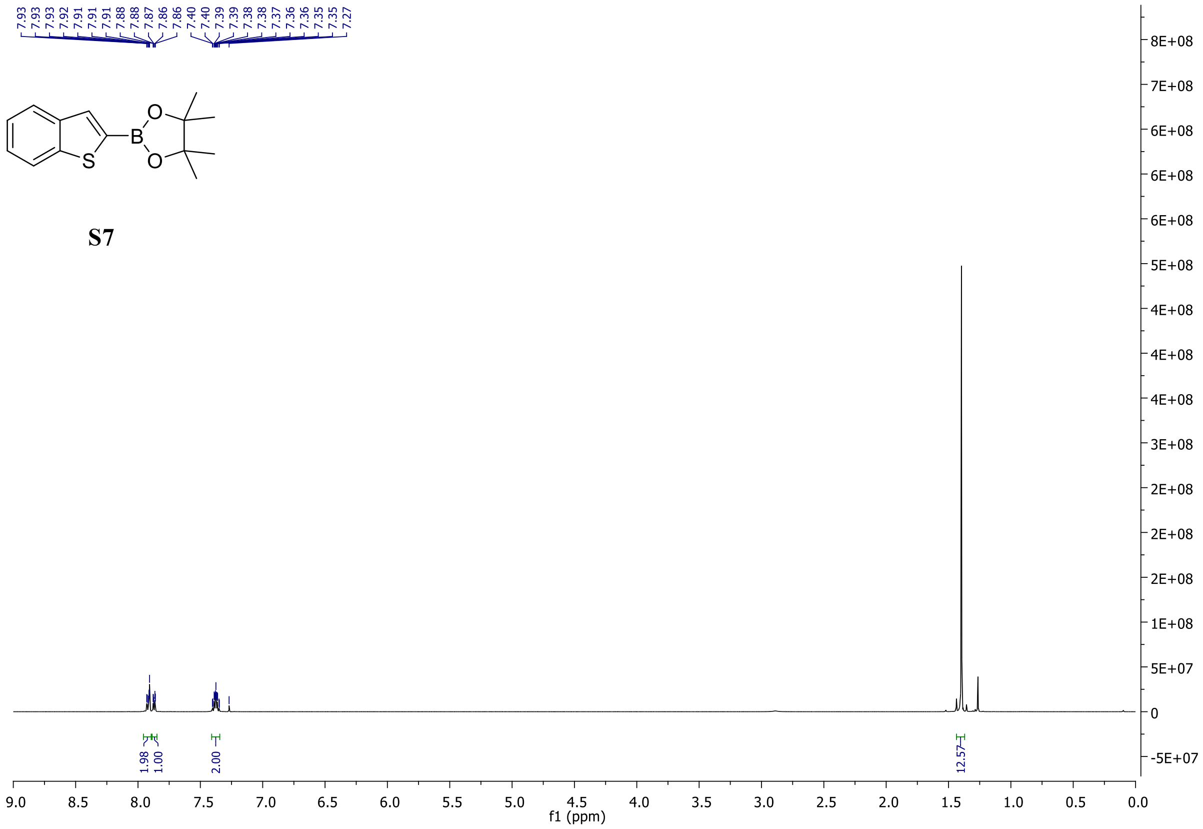

^{13}C NMR of S7

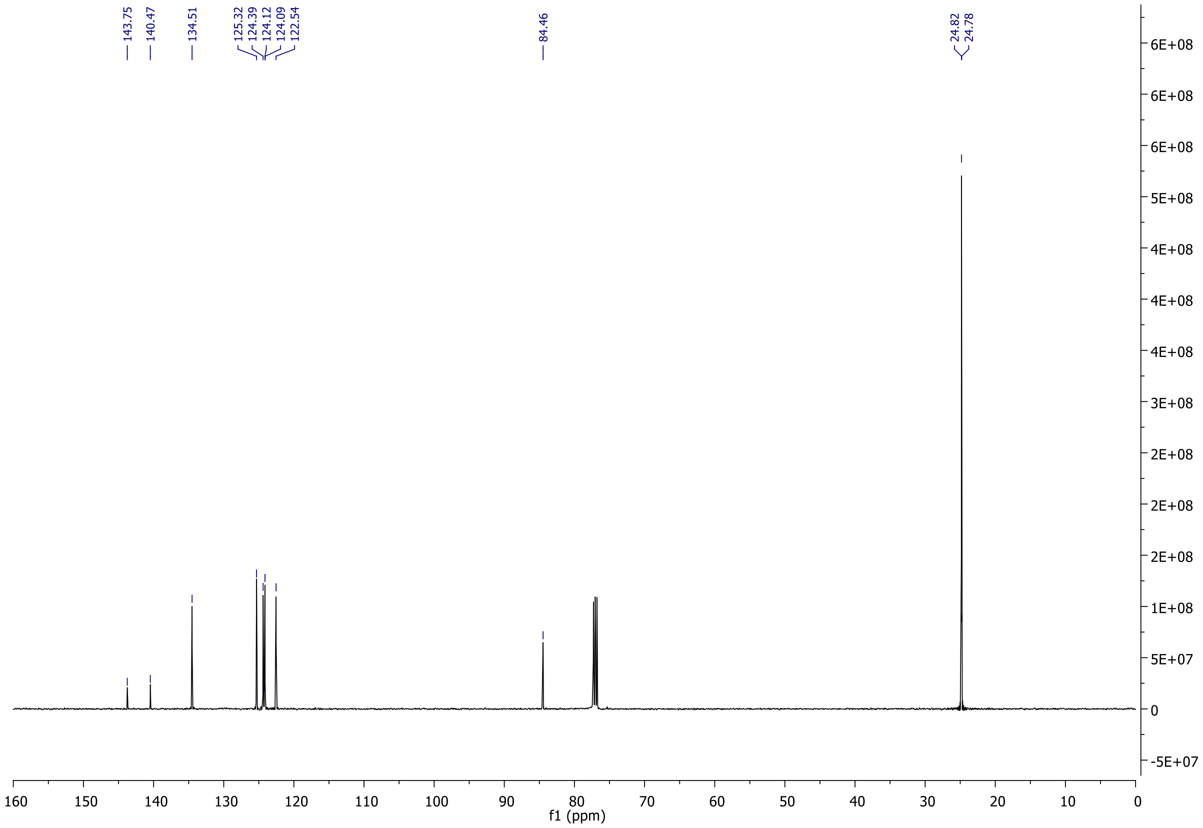

^{11}B NMR of S7

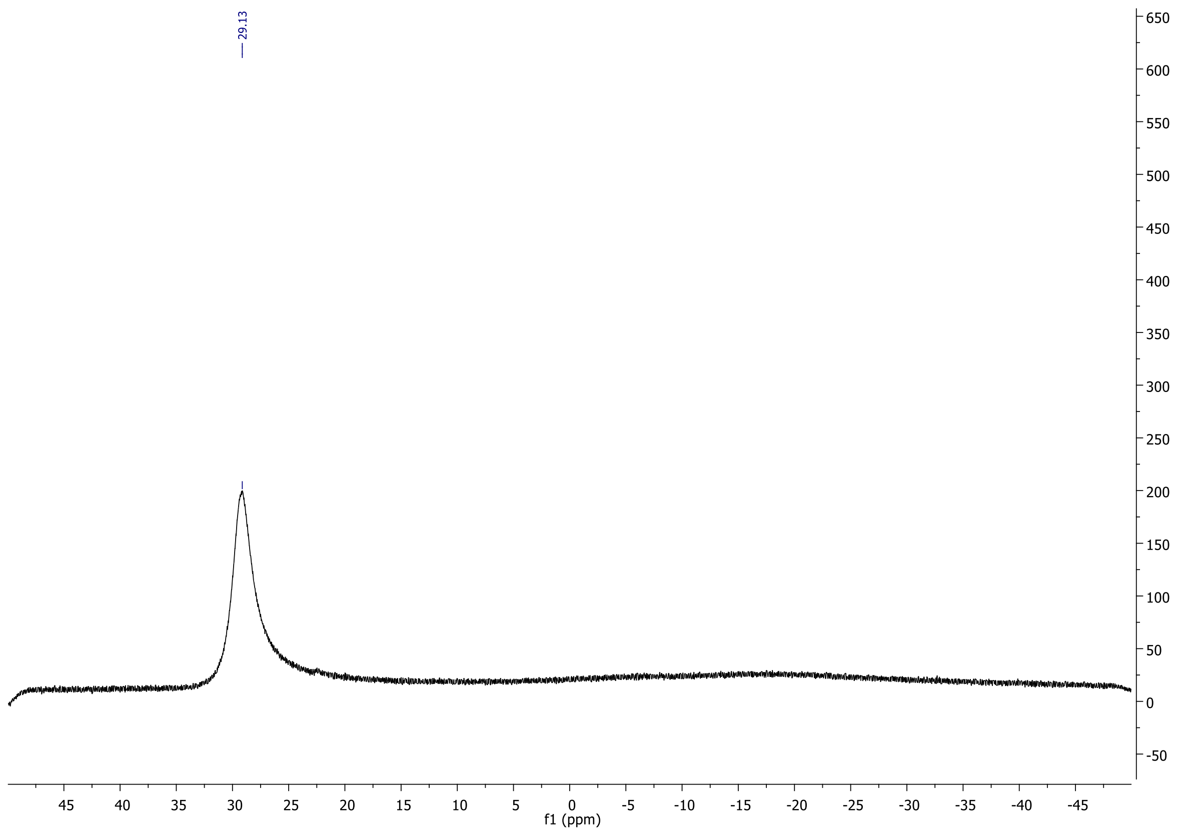

HRMS of S7

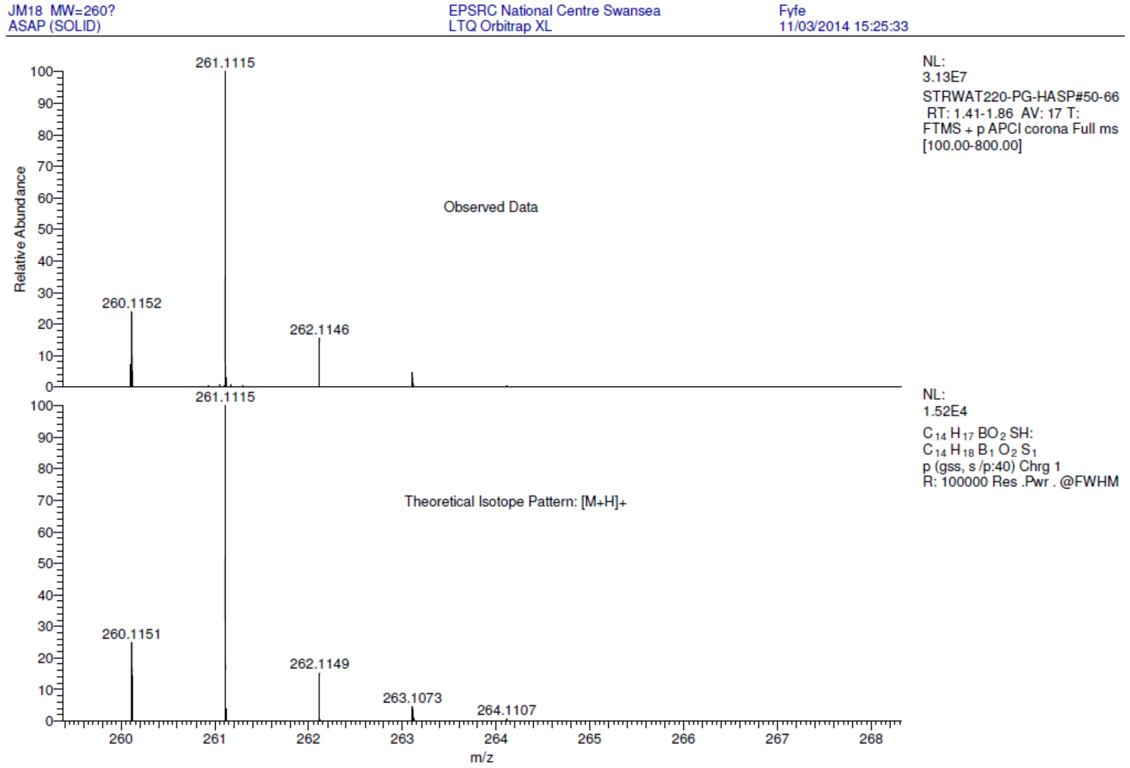

^{1}H NMR of S8

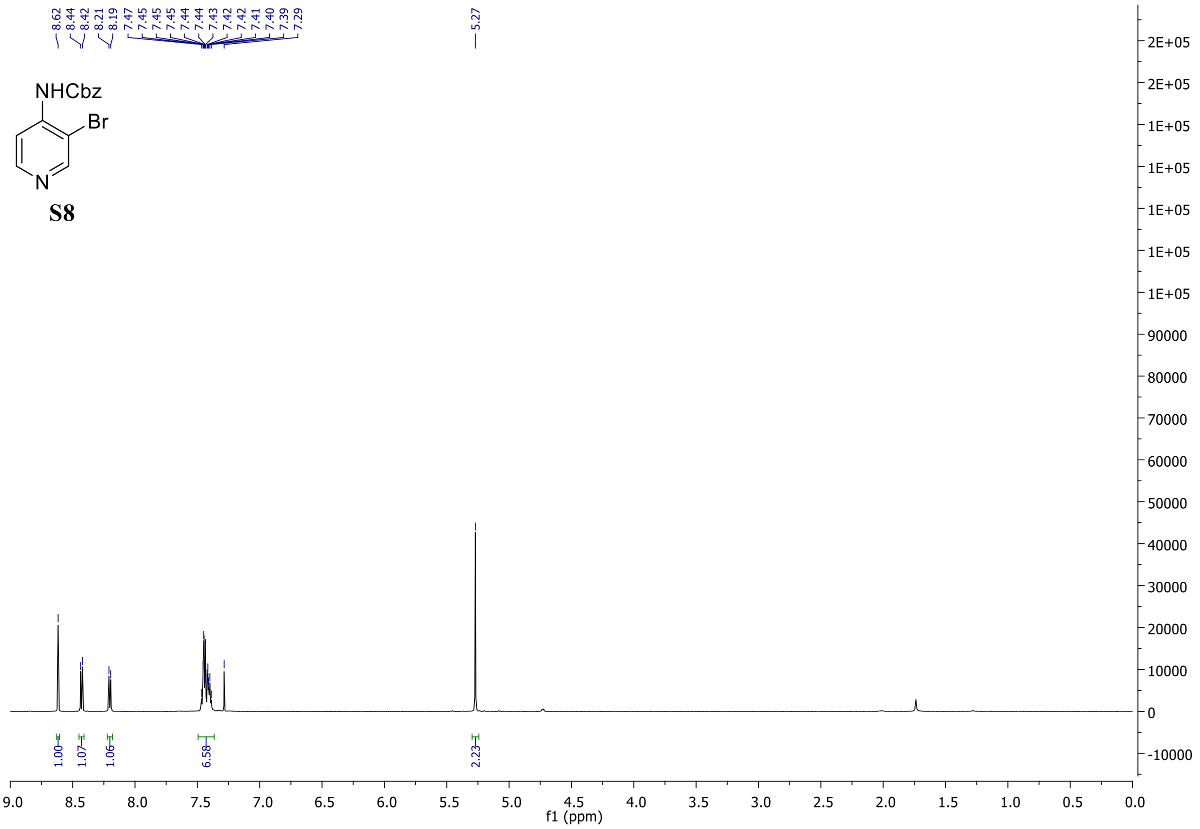

^{13}C NMR of S8

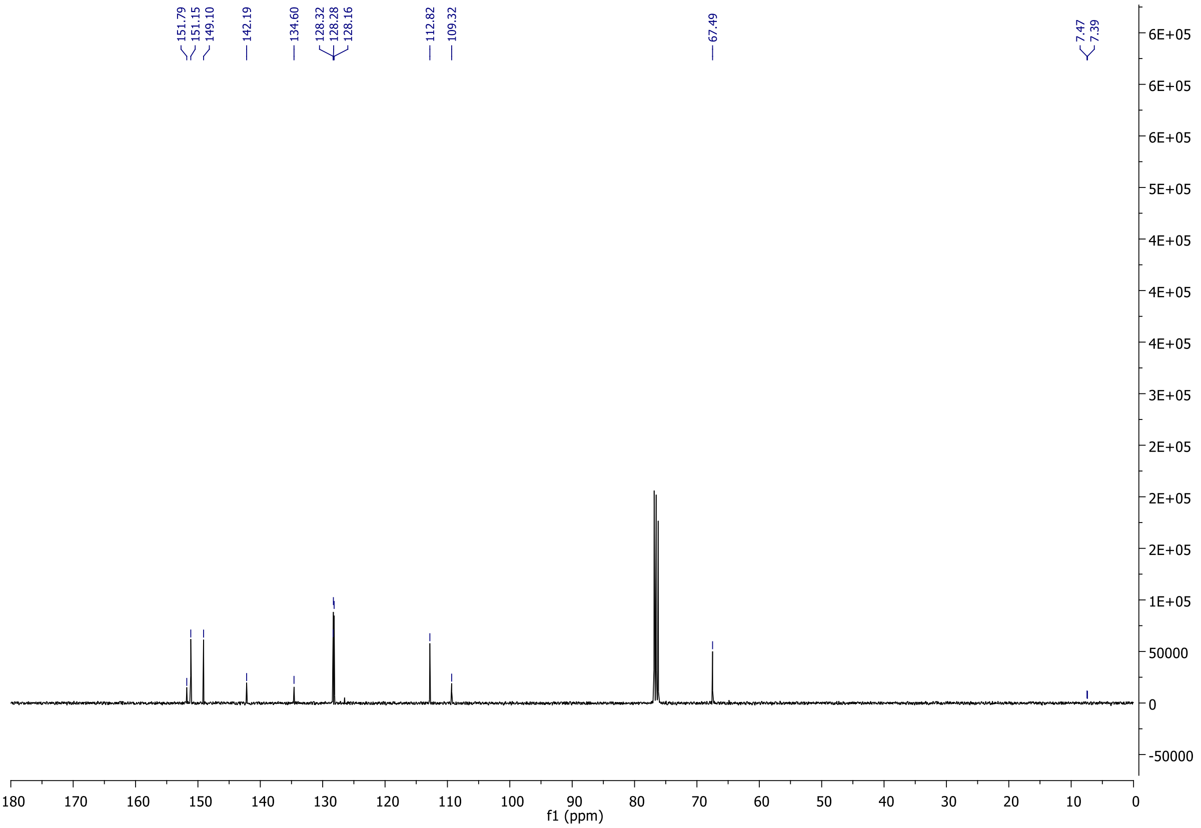

HRMS of S8

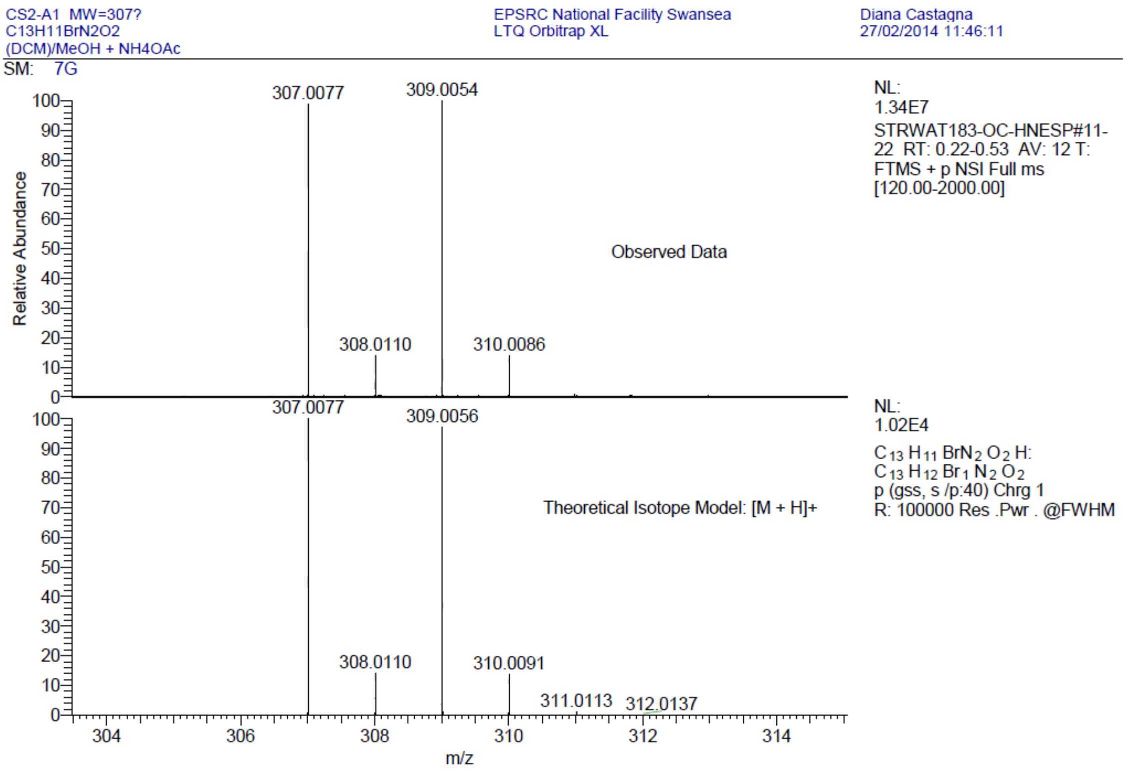

^{1}H NMR of 3a

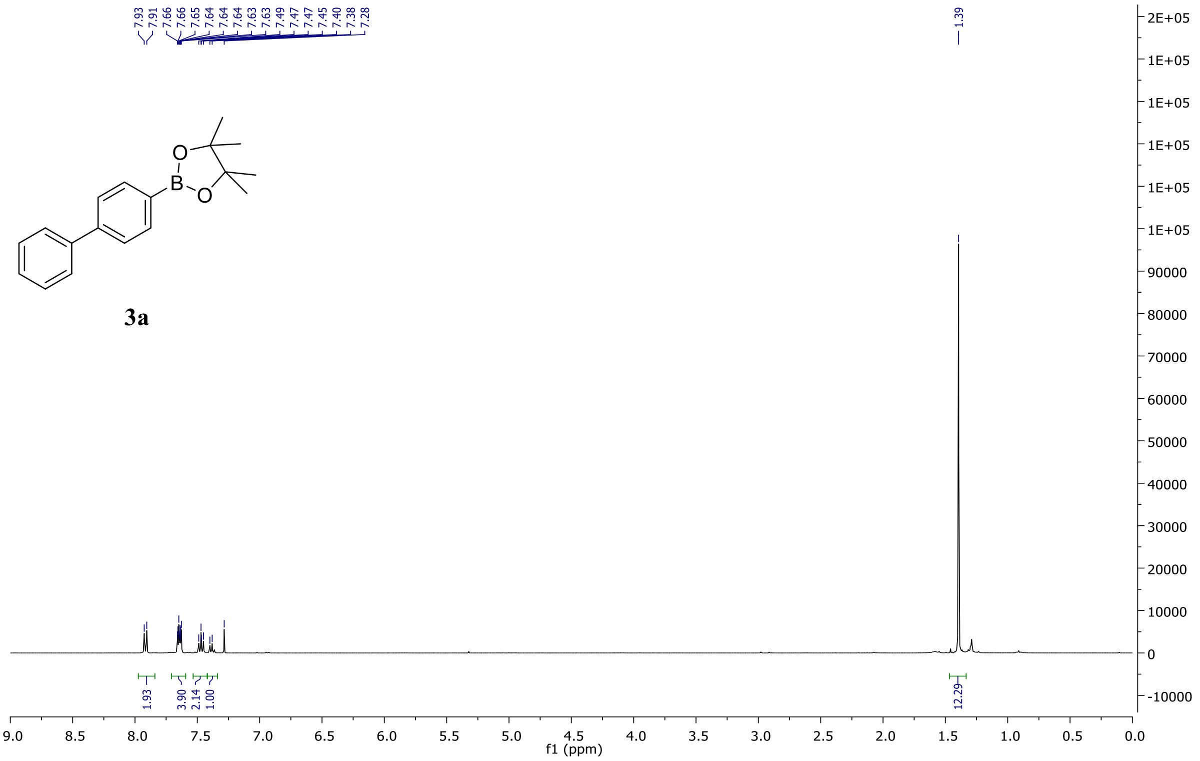

^{13}C NMR of 3a

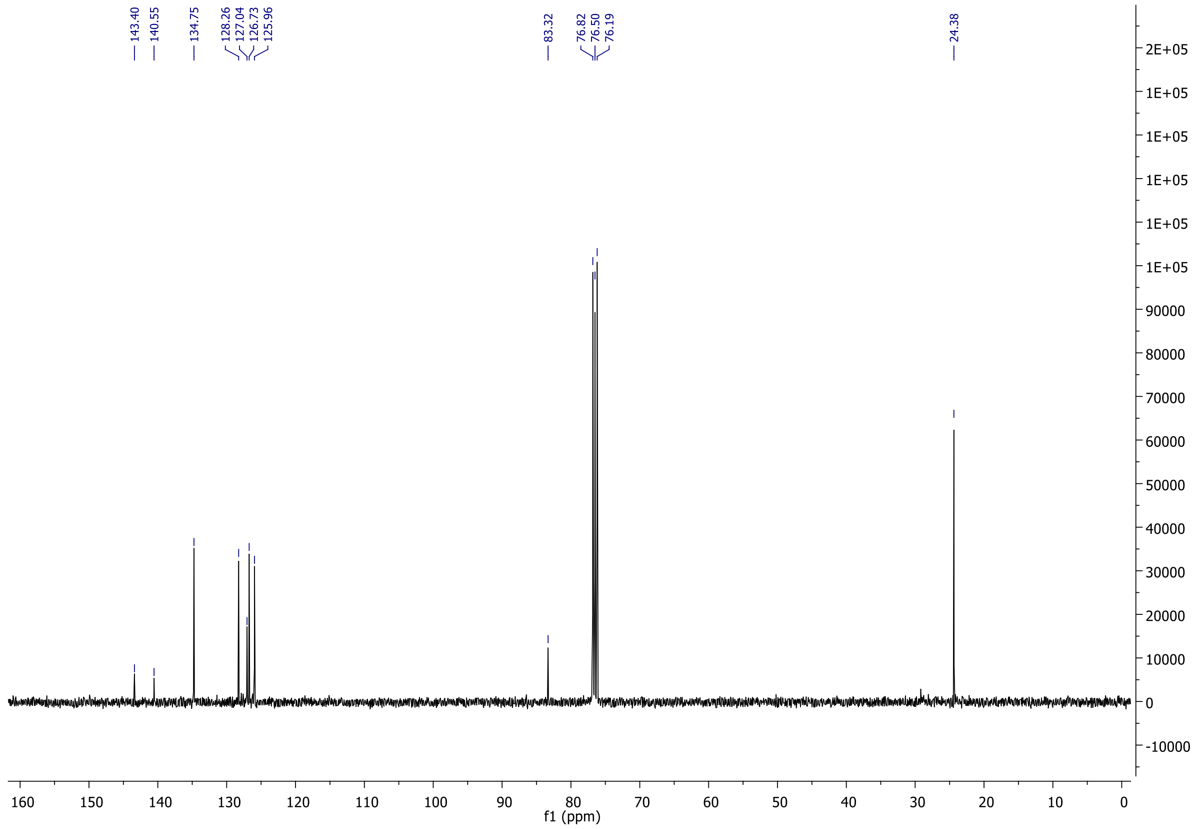

^{11}B NMR of 3b

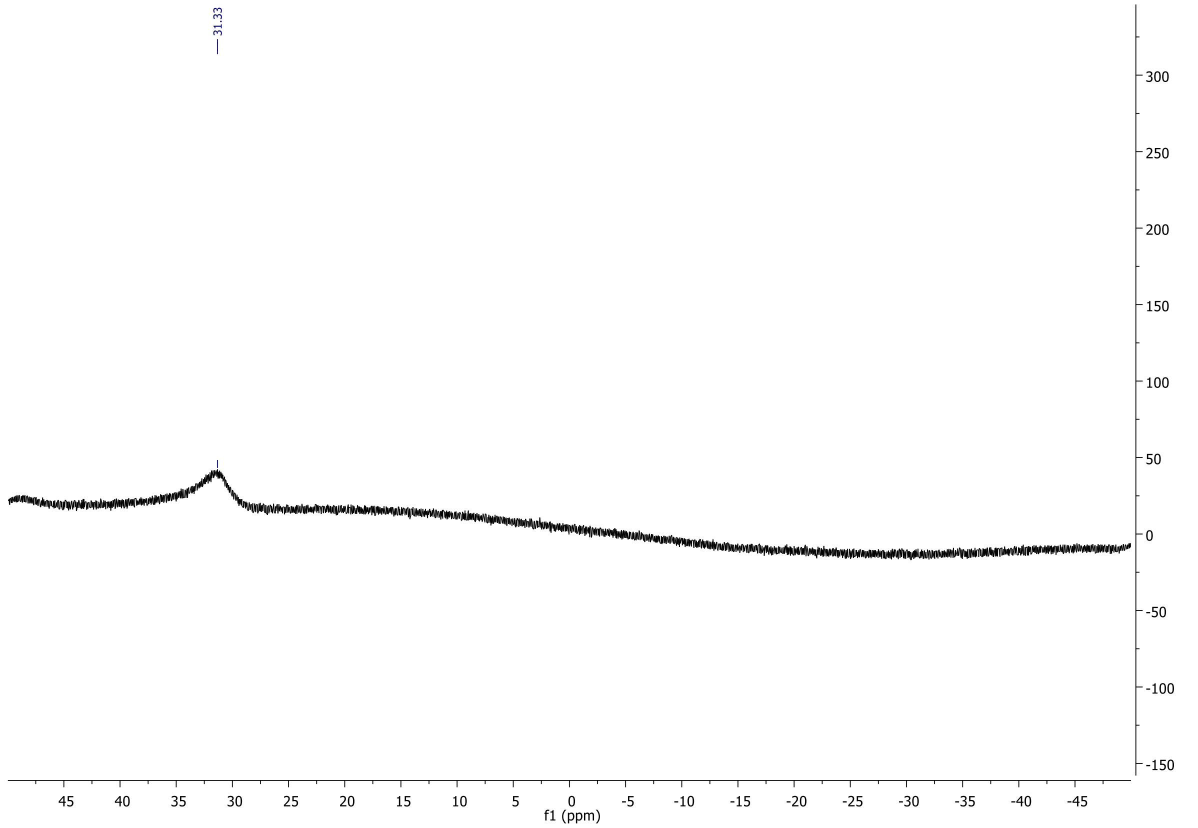

HRMS of 3b

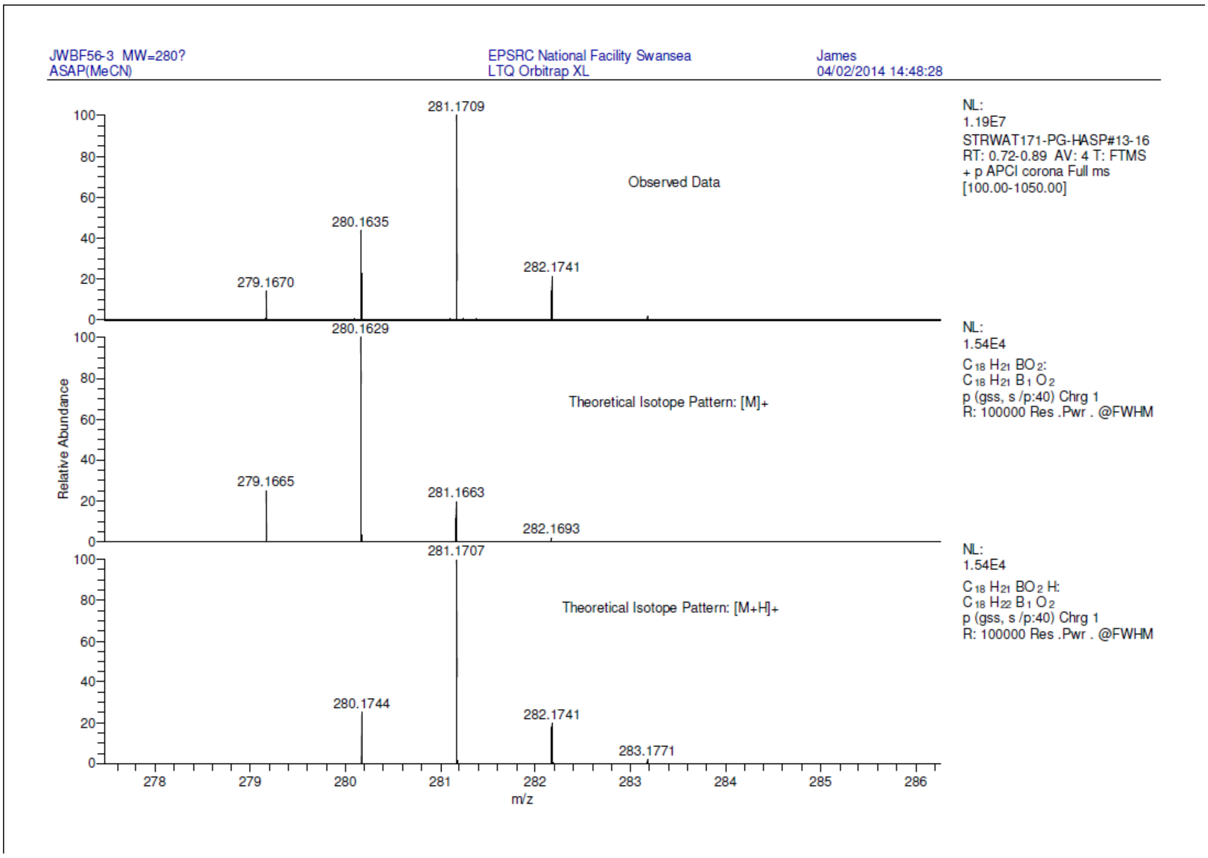

^{1}H NMR of 3b

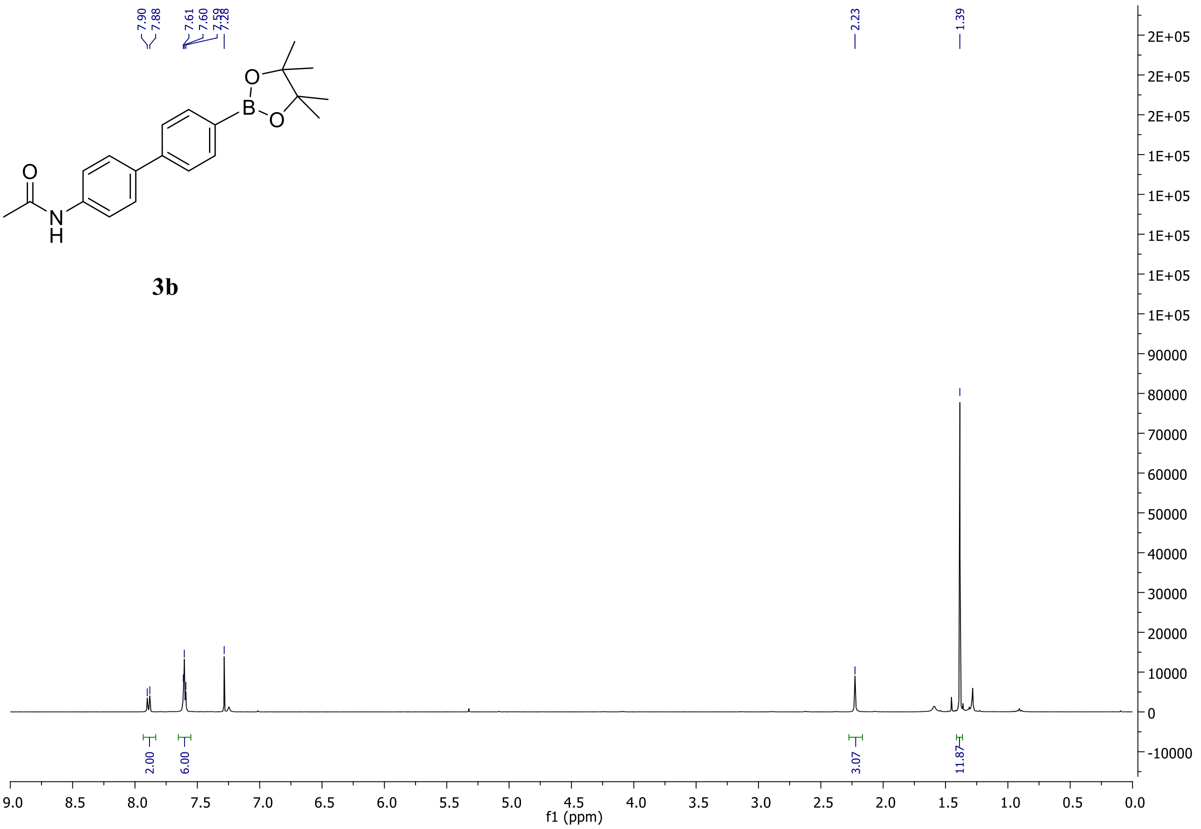

3b

^{13}C NMR of 3b

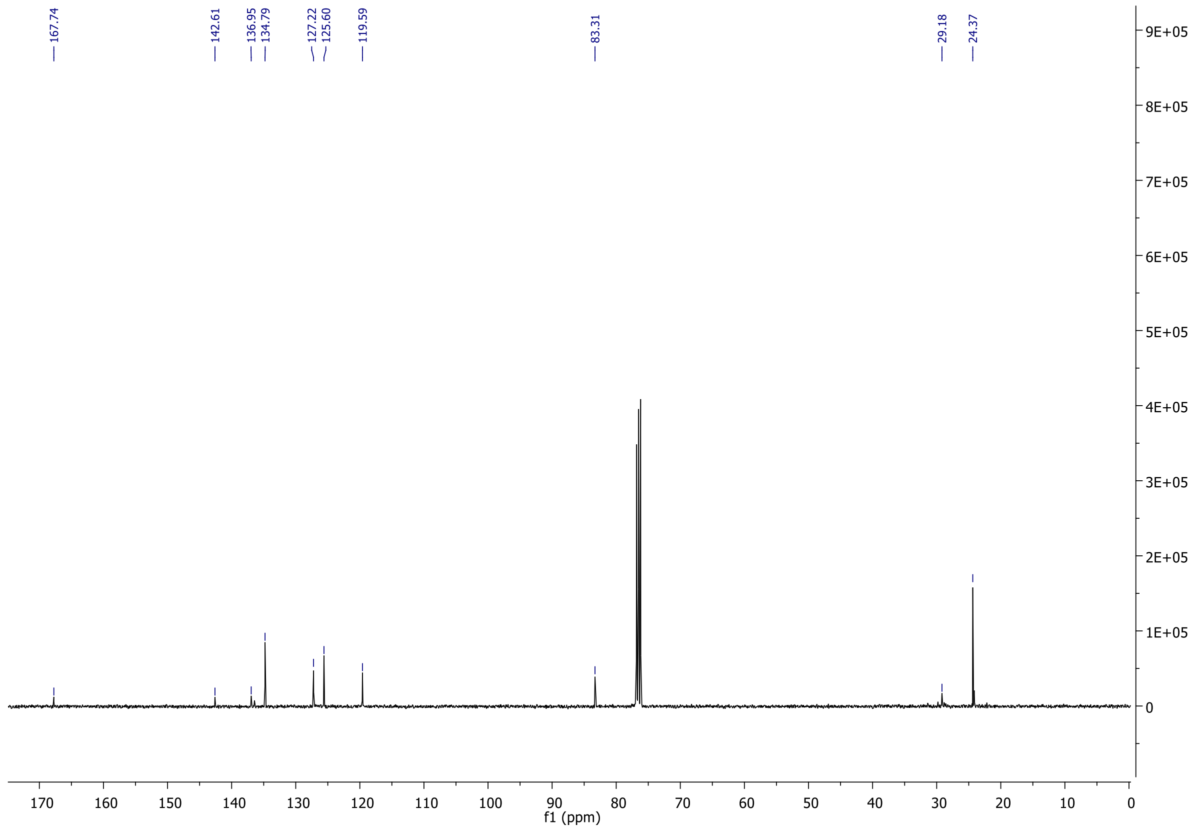

^{11}B NMR of 3b

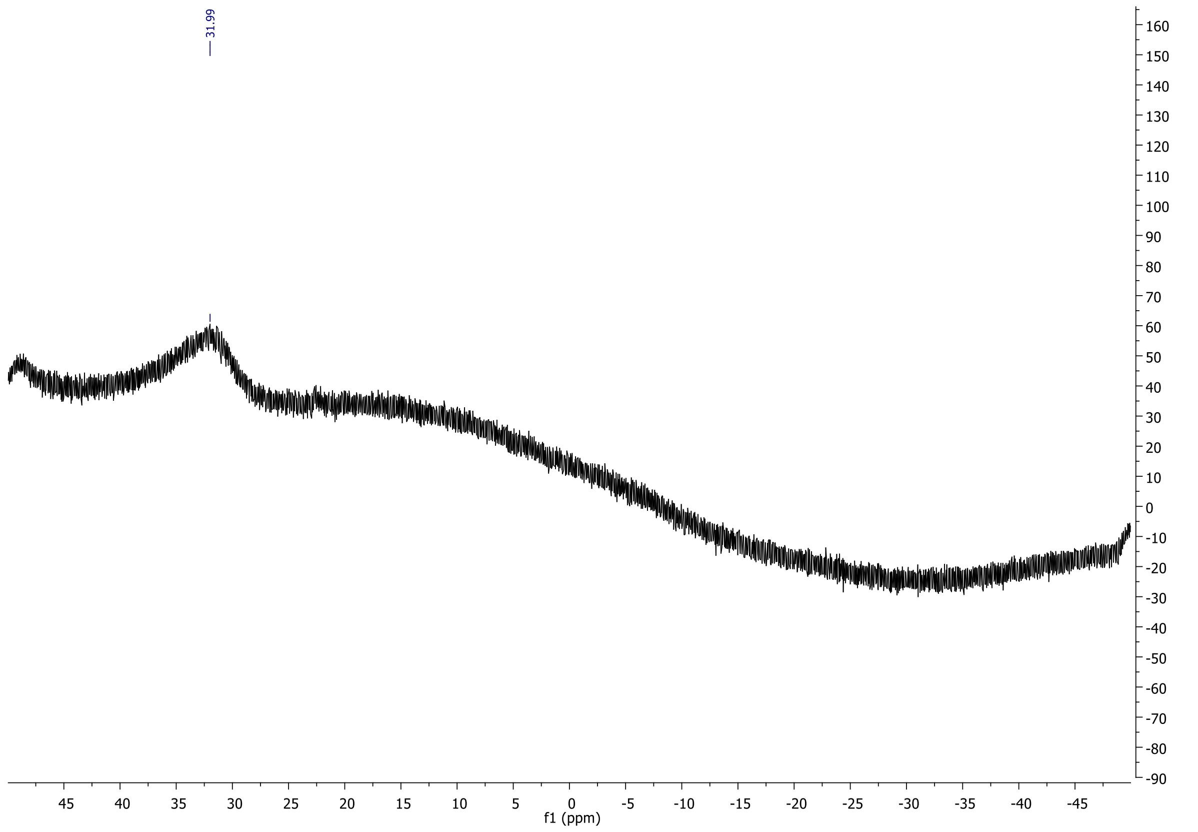

HRMS of 3b

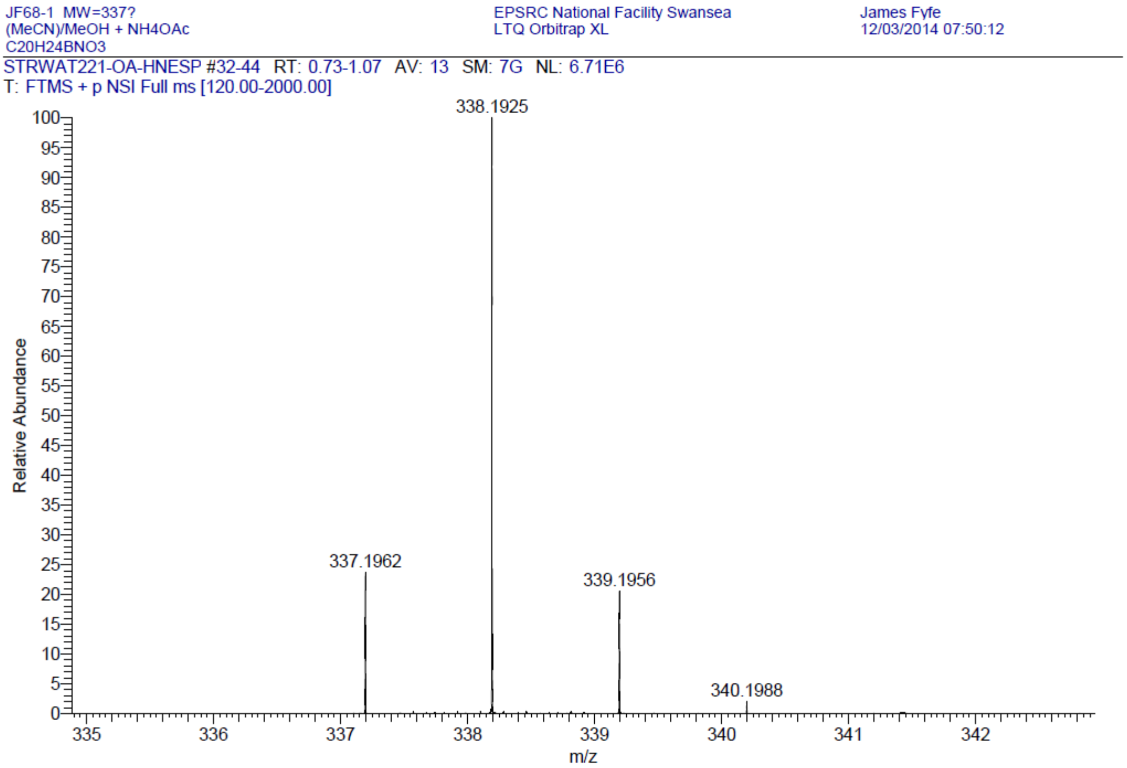

^{1}H NMR of 3c

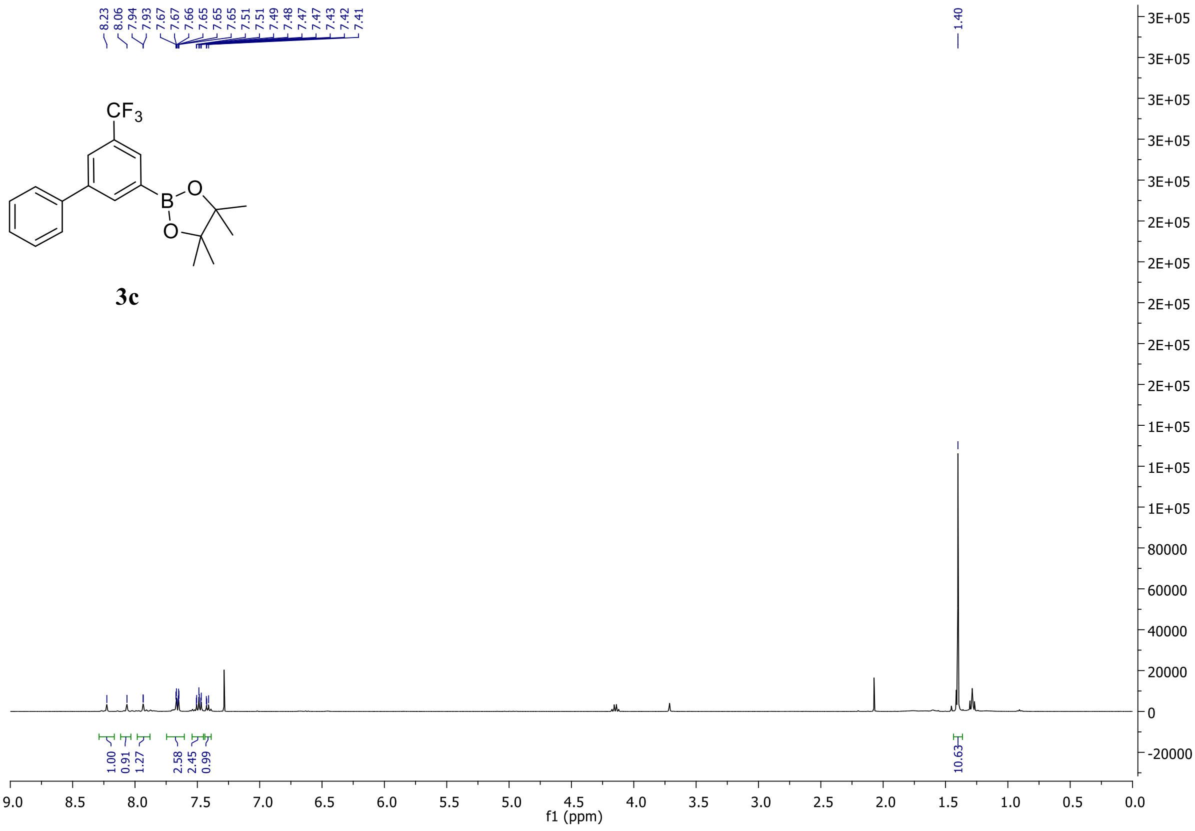

^{13}C NMR of 3c

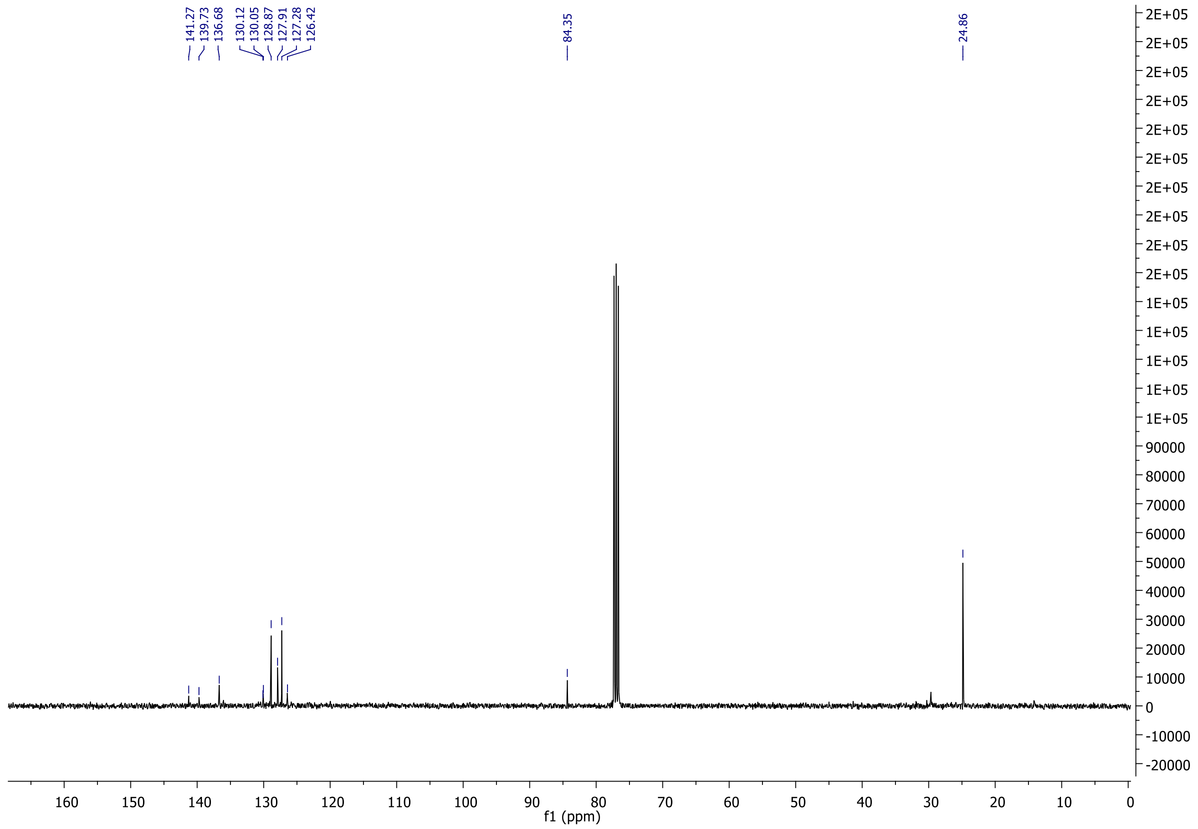

^{11}B NMR of 3c

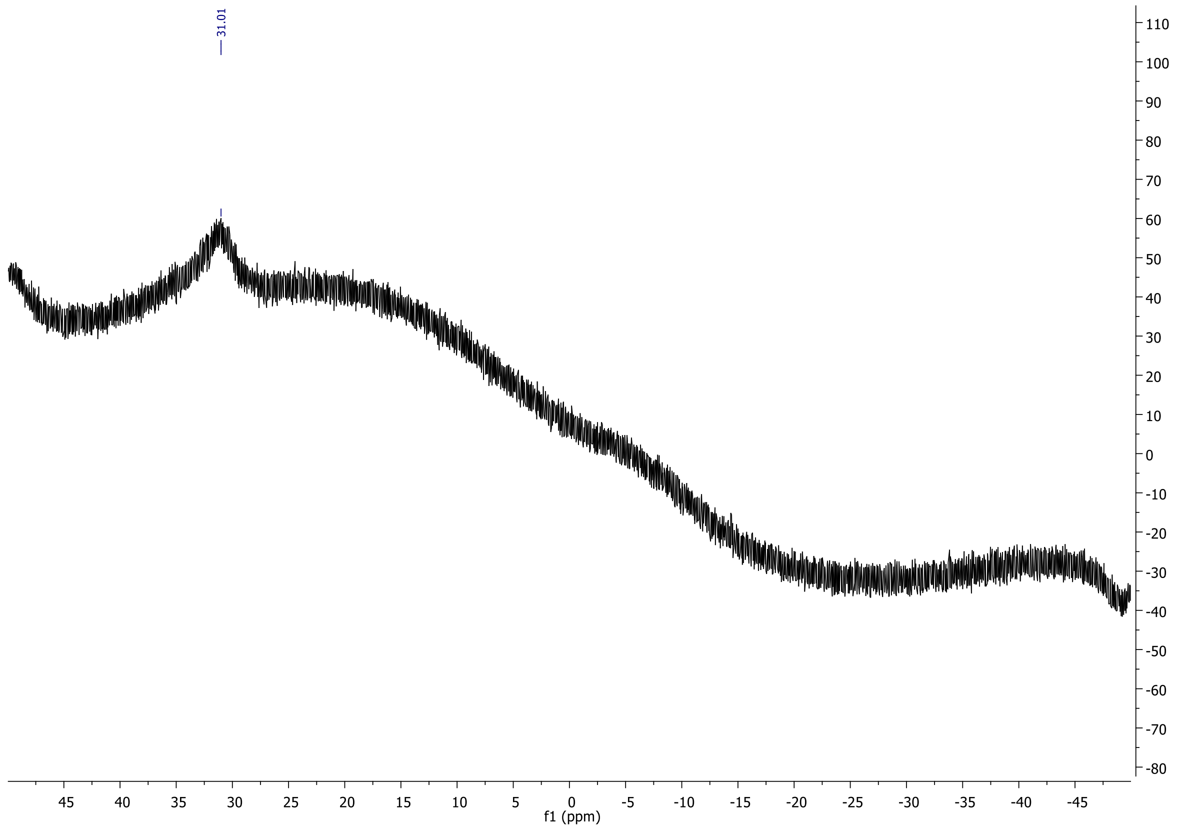

^{19}F NMR of 3c

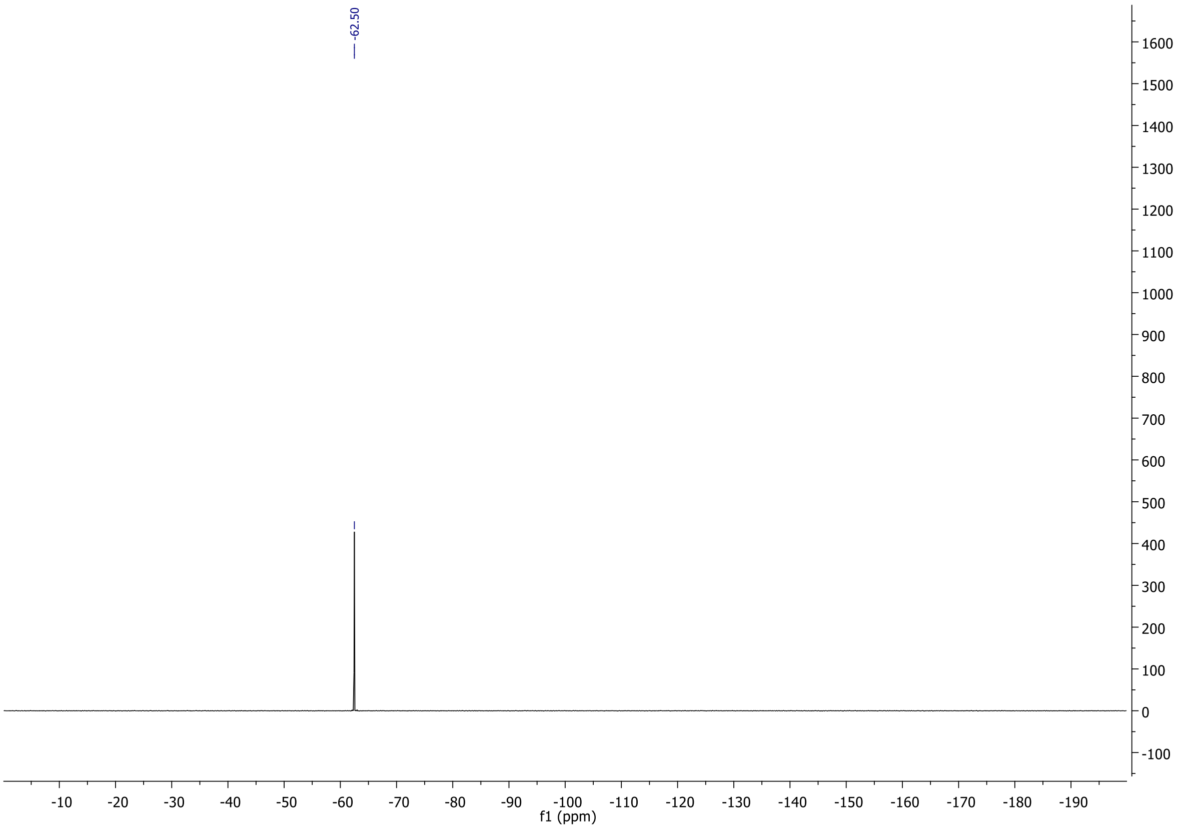

HRMS of 3c

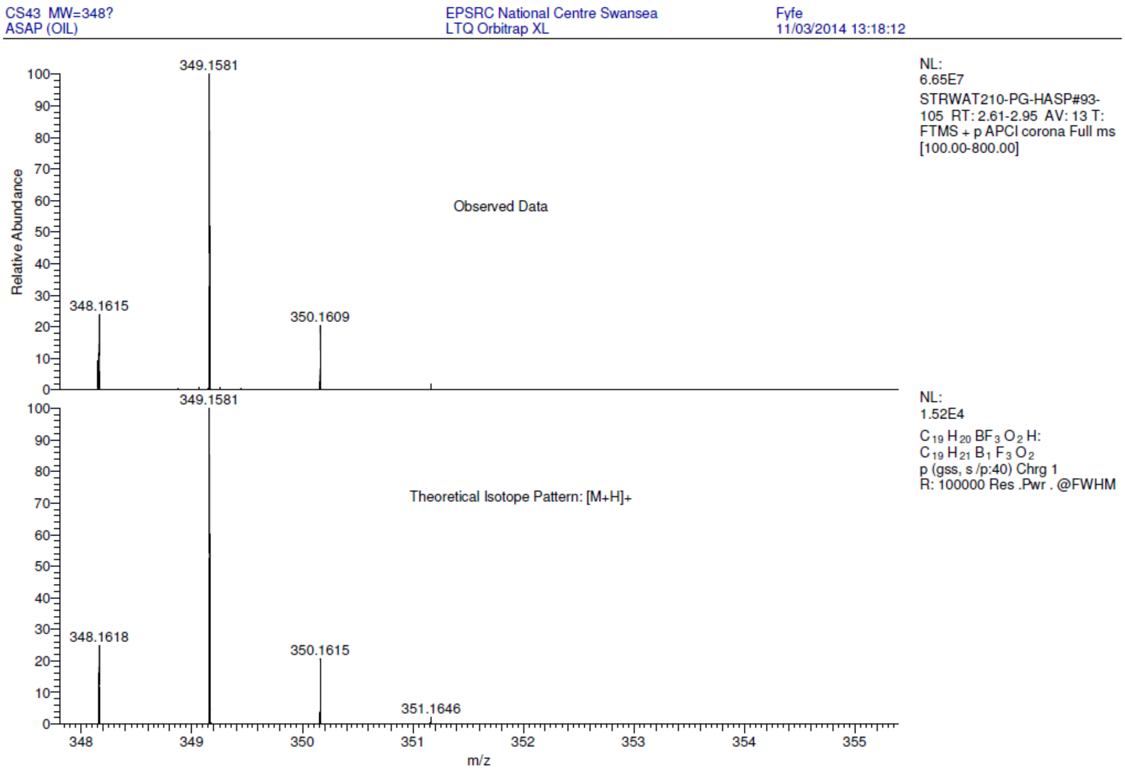

^{1}H NMR of 3d

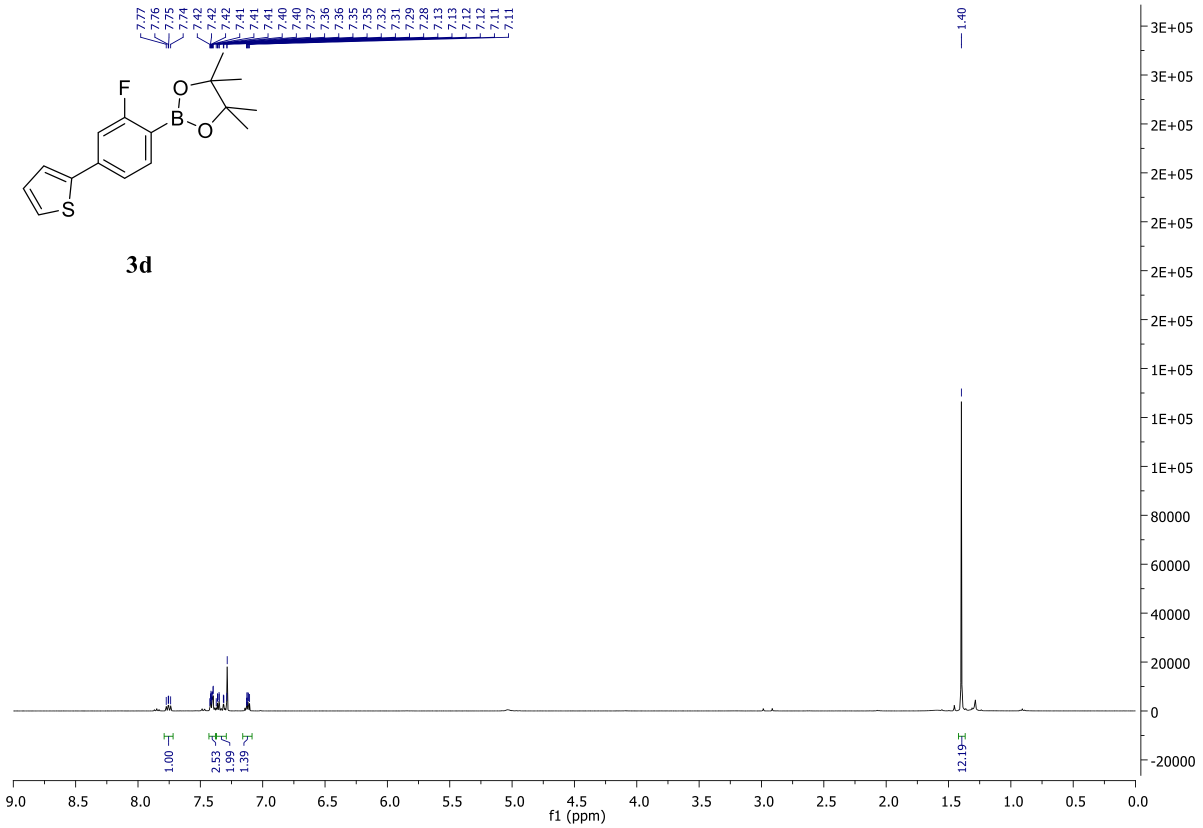

^{13}C NMR of 3d

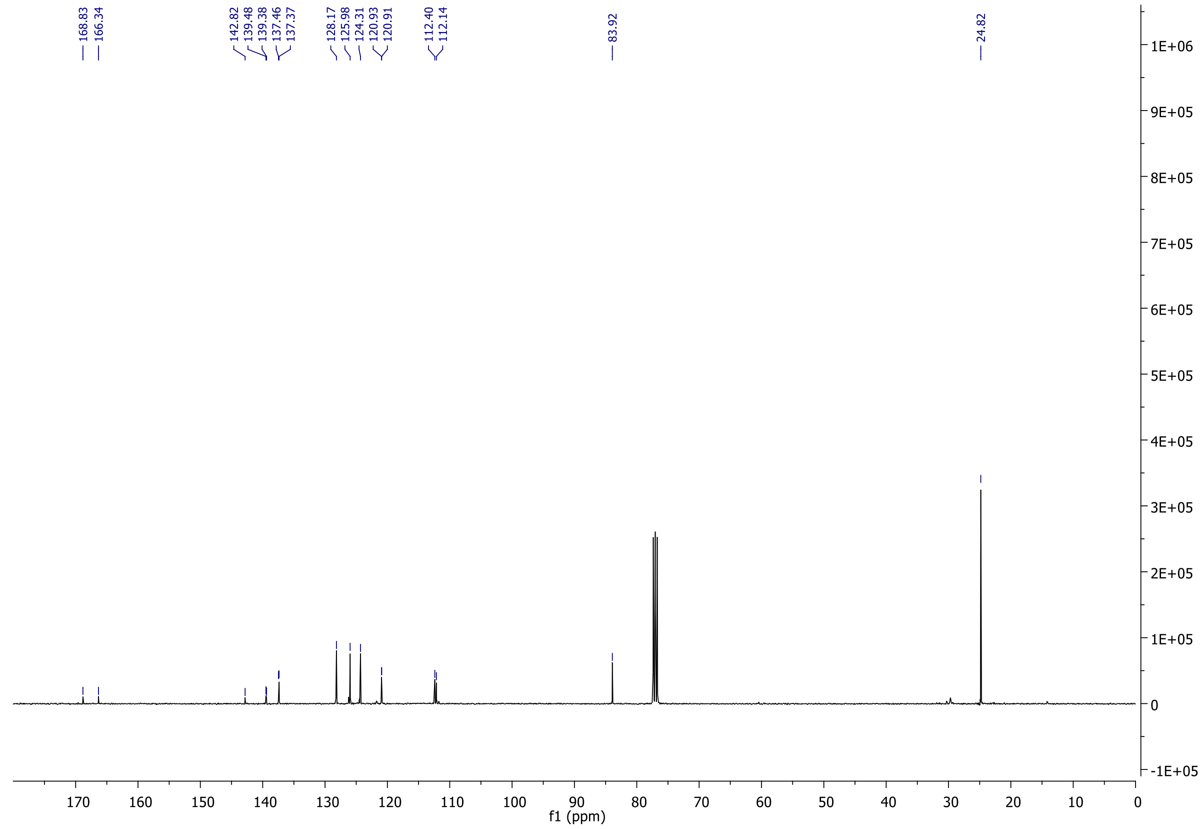

^{11}B NMR of 3d

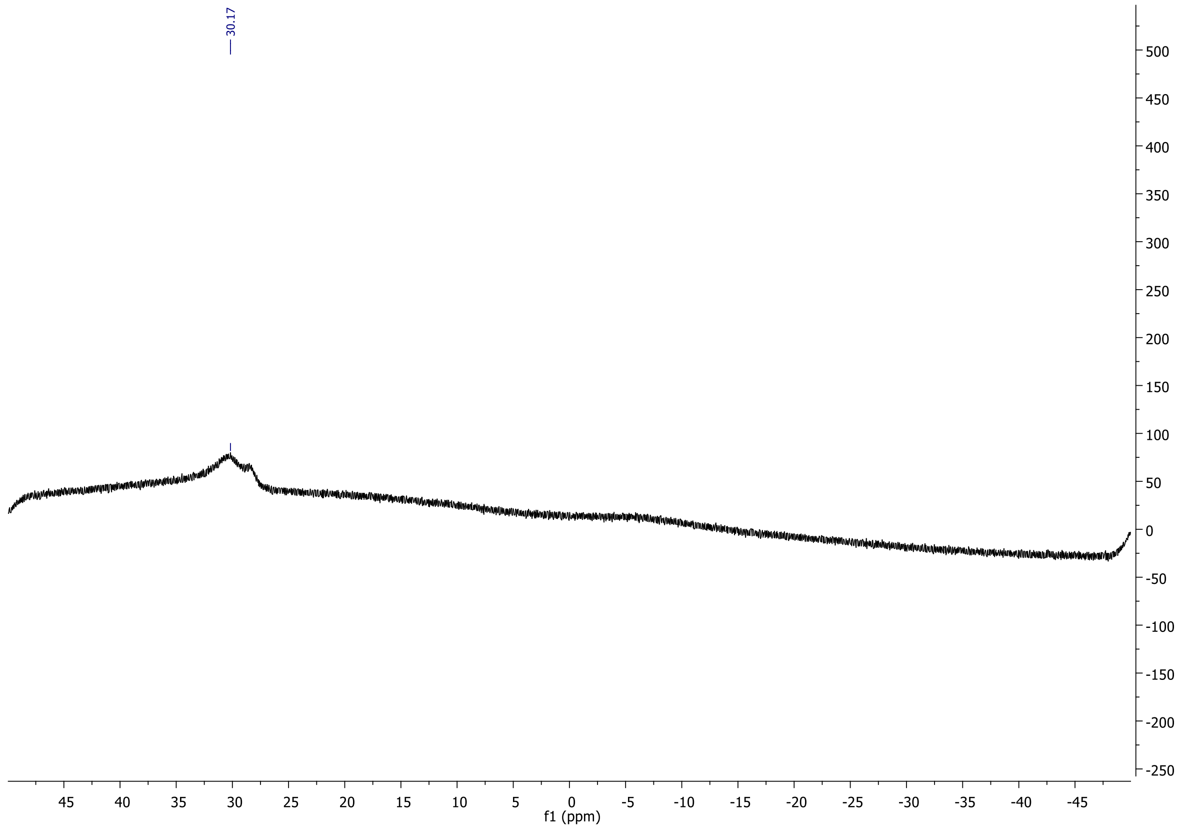

^{19}F NMR of 3d

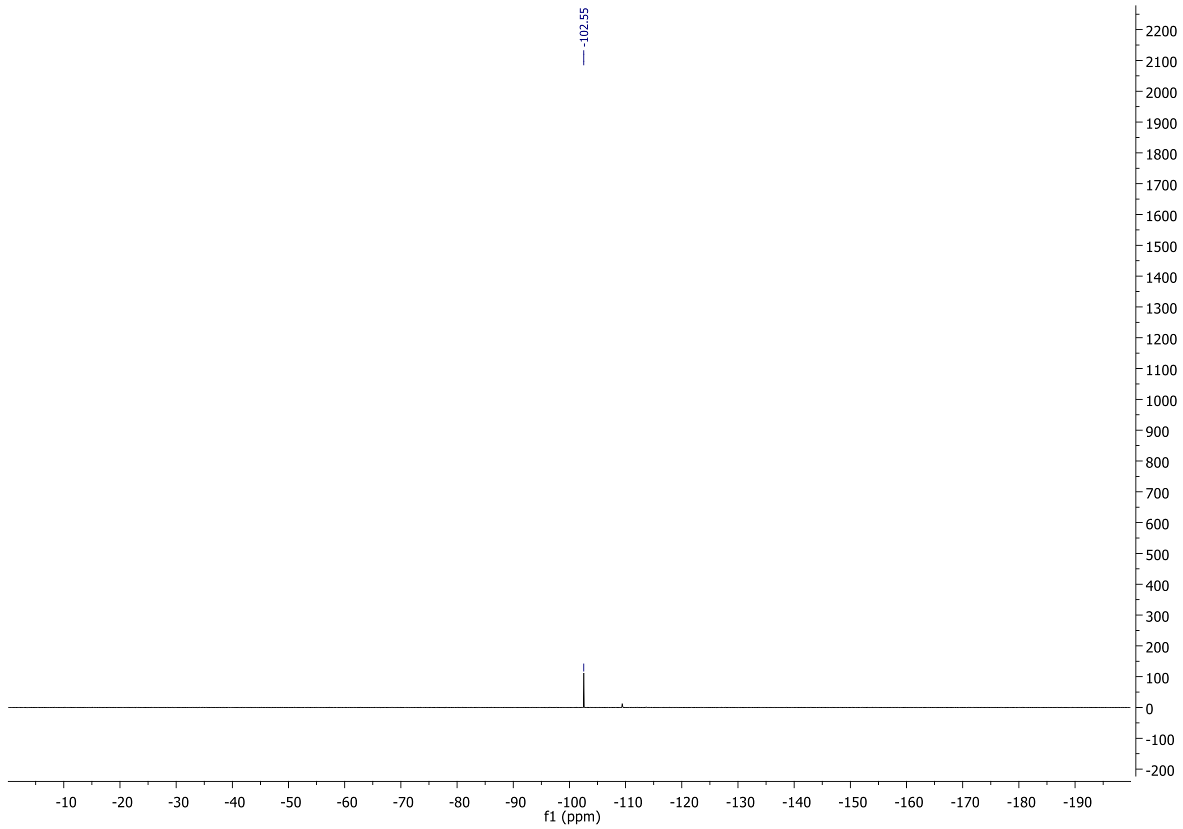

HRMS of 3d

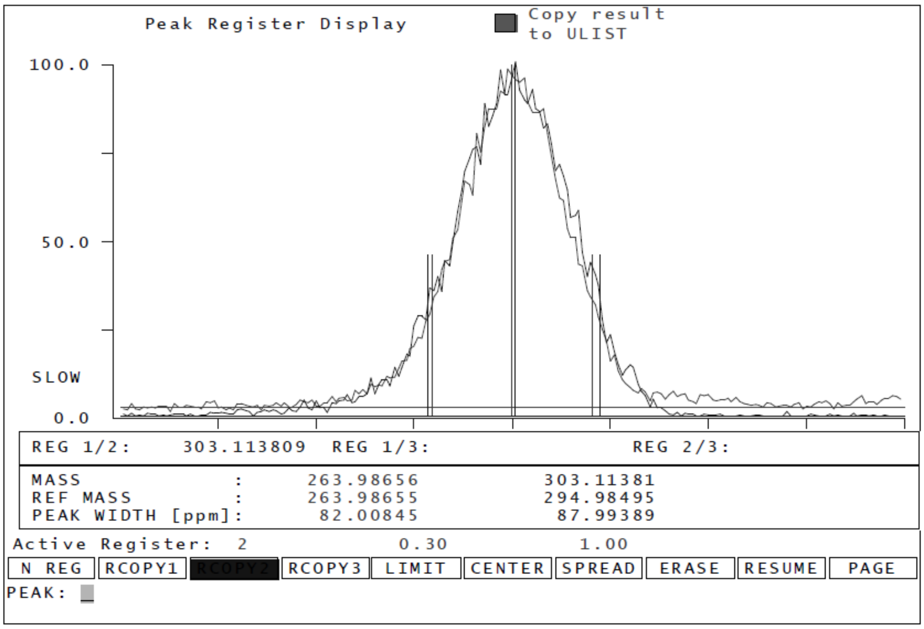

12/05/2014 10:29:45 CMEP755MAT95 MAT 95 XP

^{1}H NMR of 3e

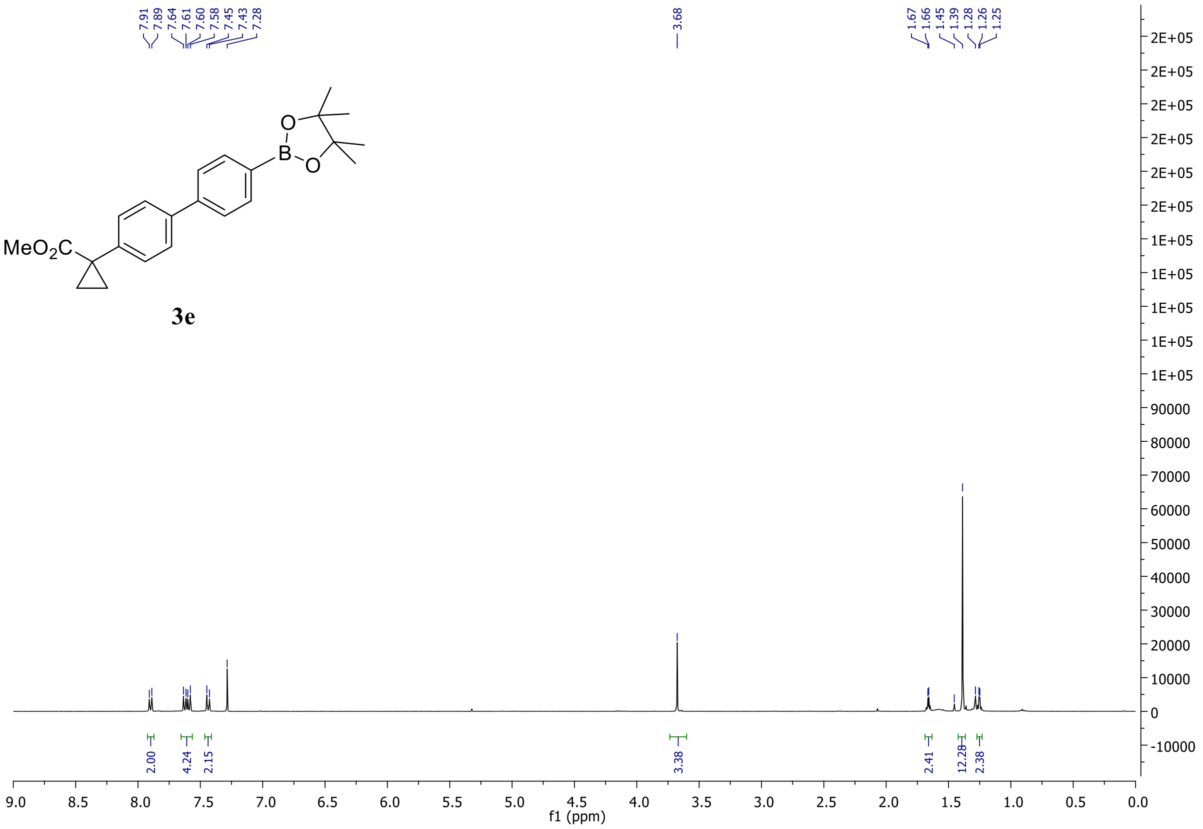

^{13}C NMR of 3e

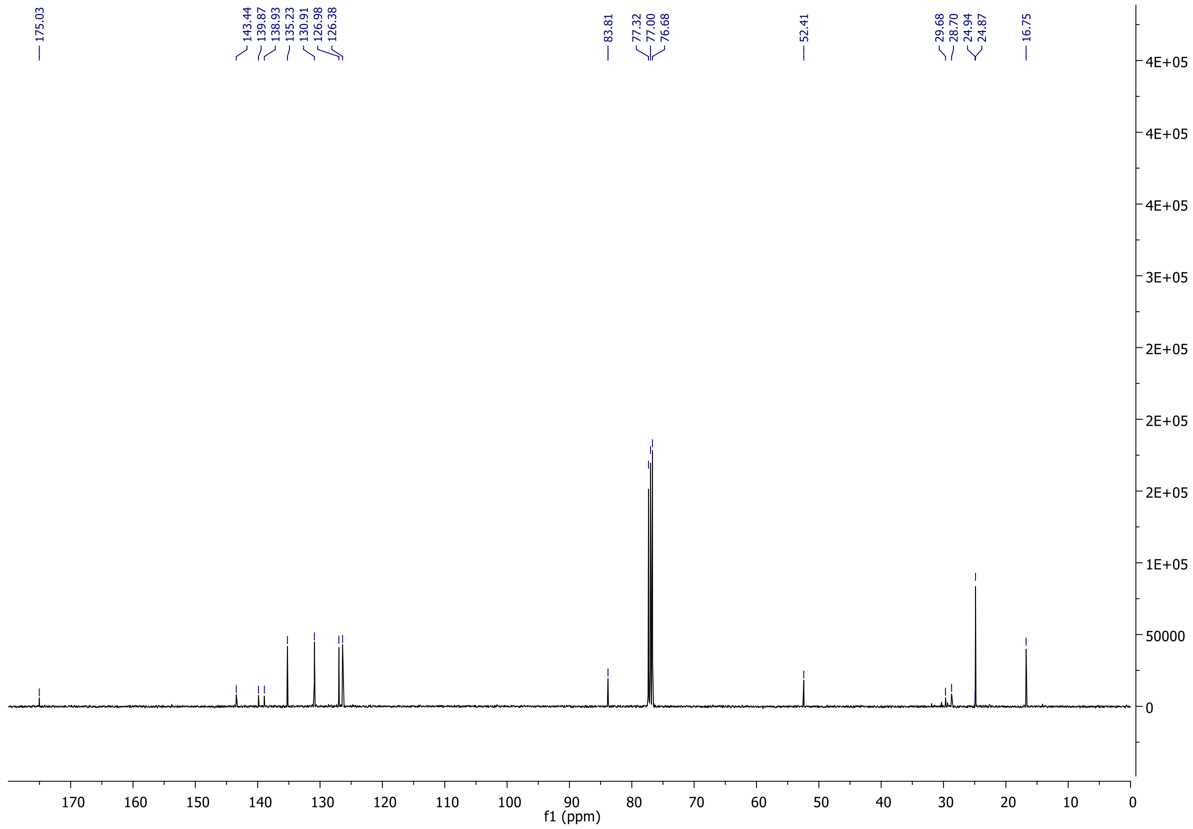

^{11}B NMR of 3e

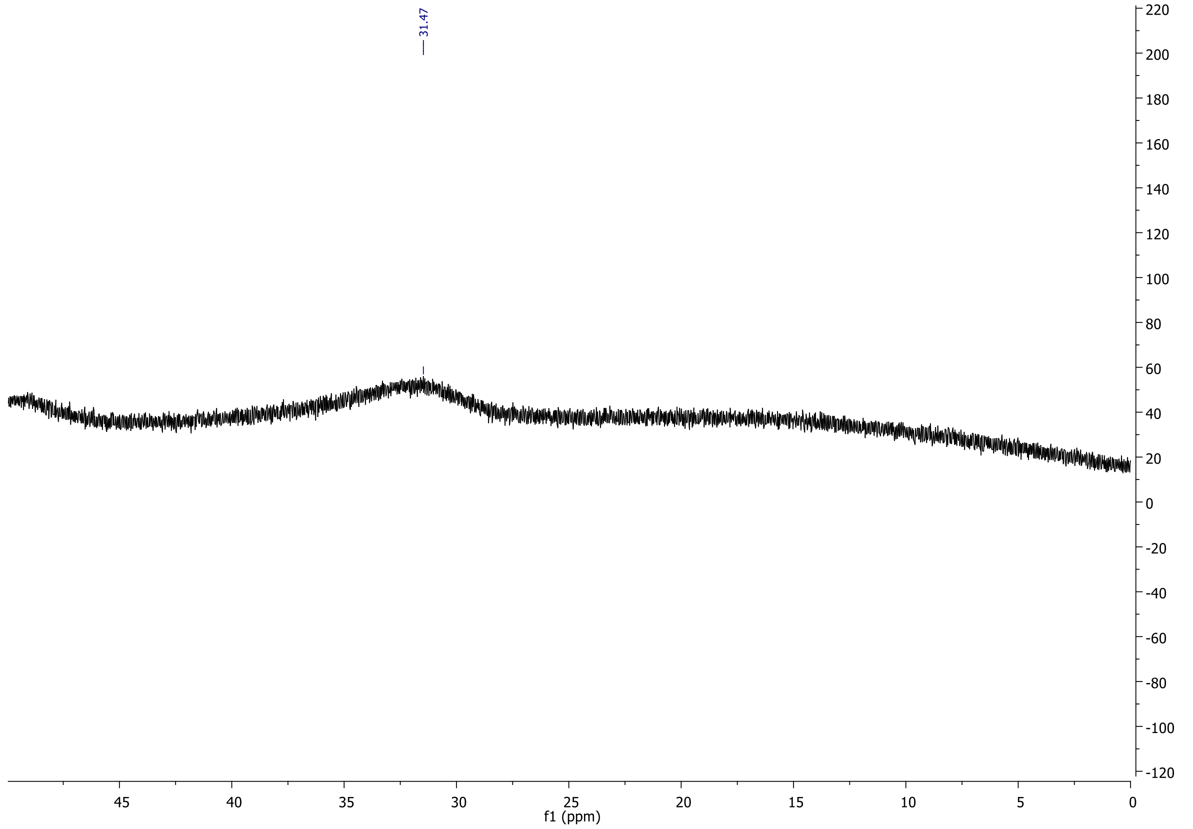

HRMS of 3e

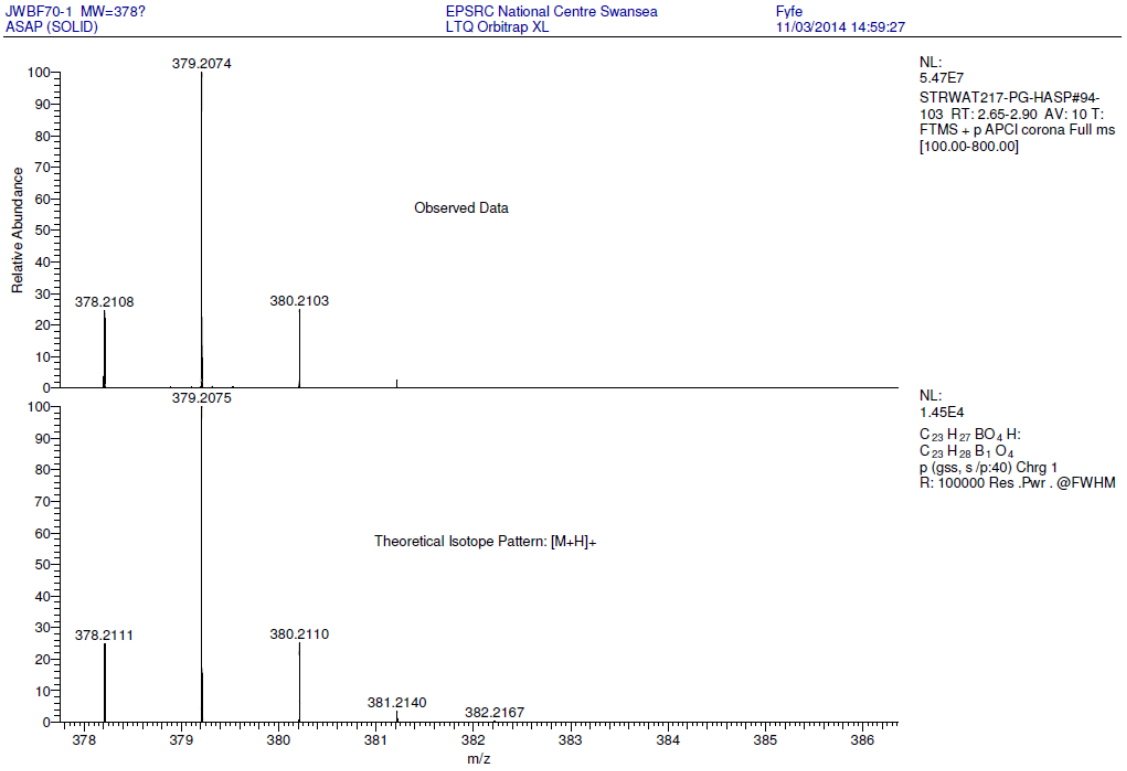

^{1}H NMR of 3f

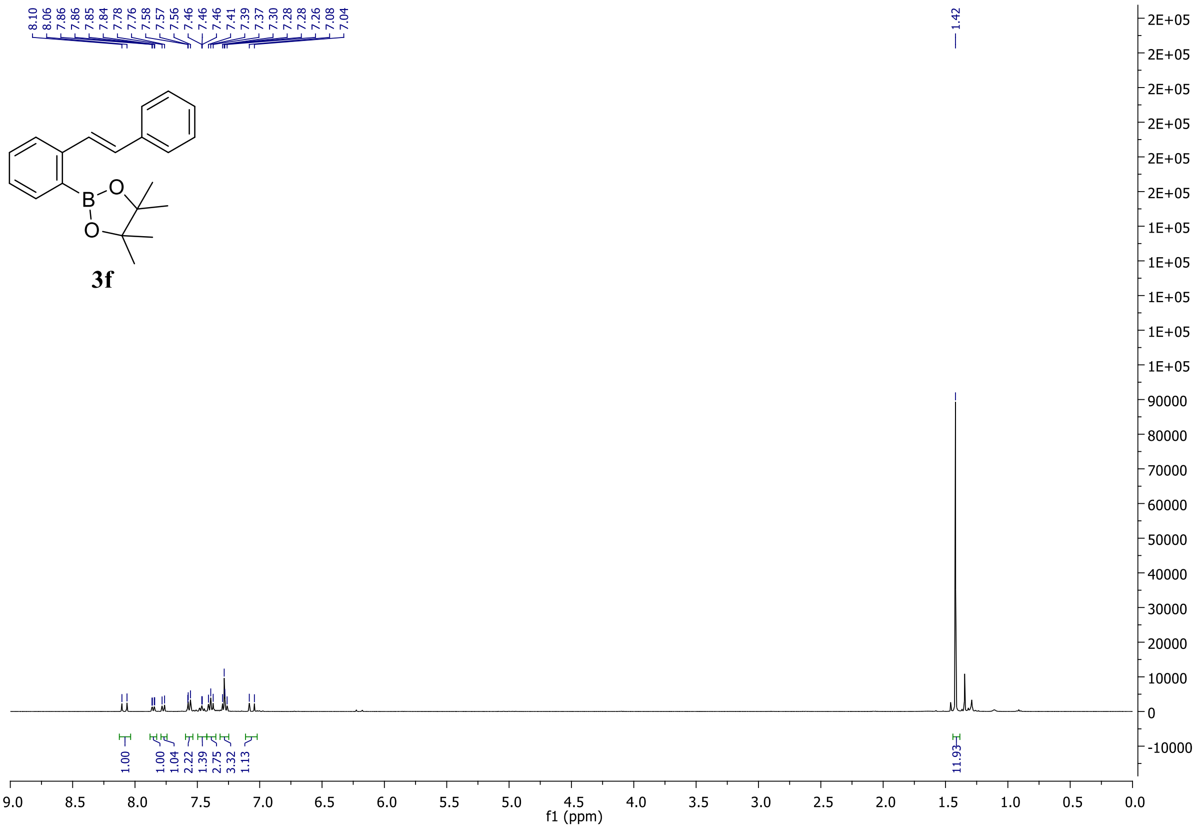

^{13}C NMR of 3f

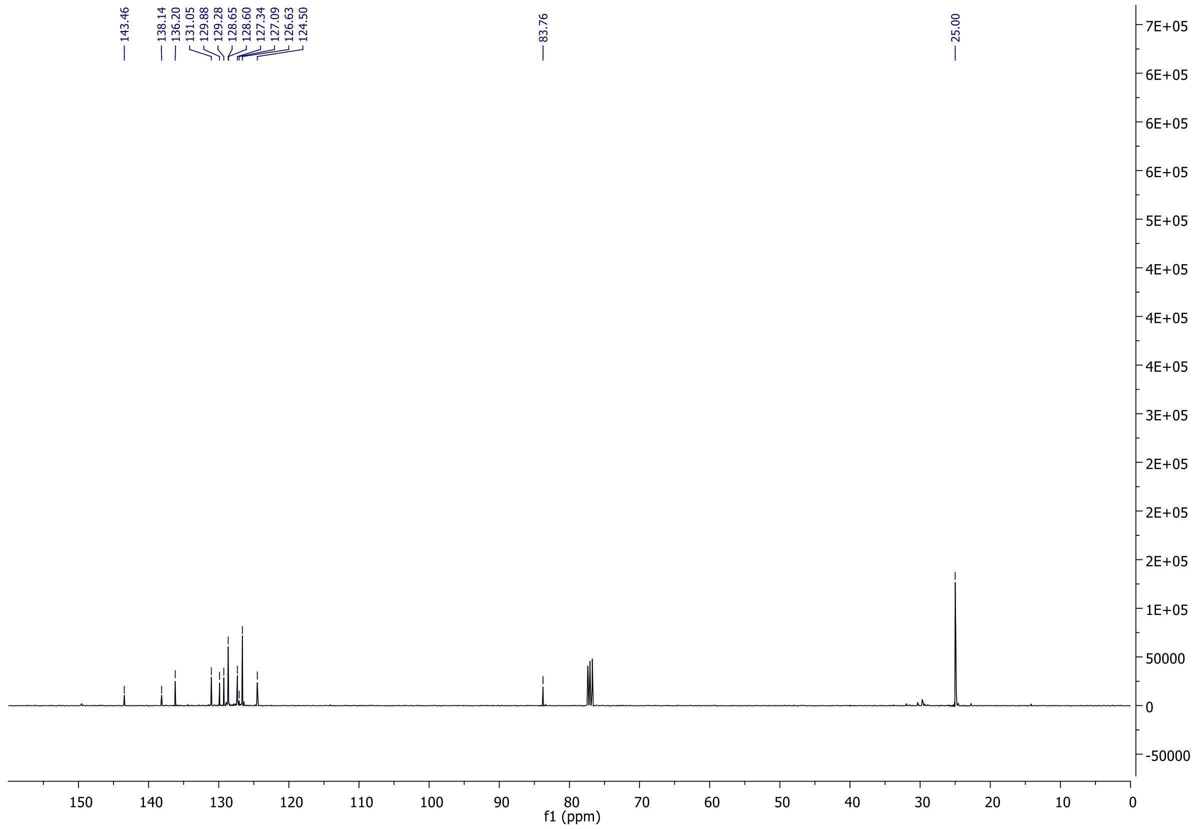

^{11}B NMR of 3f

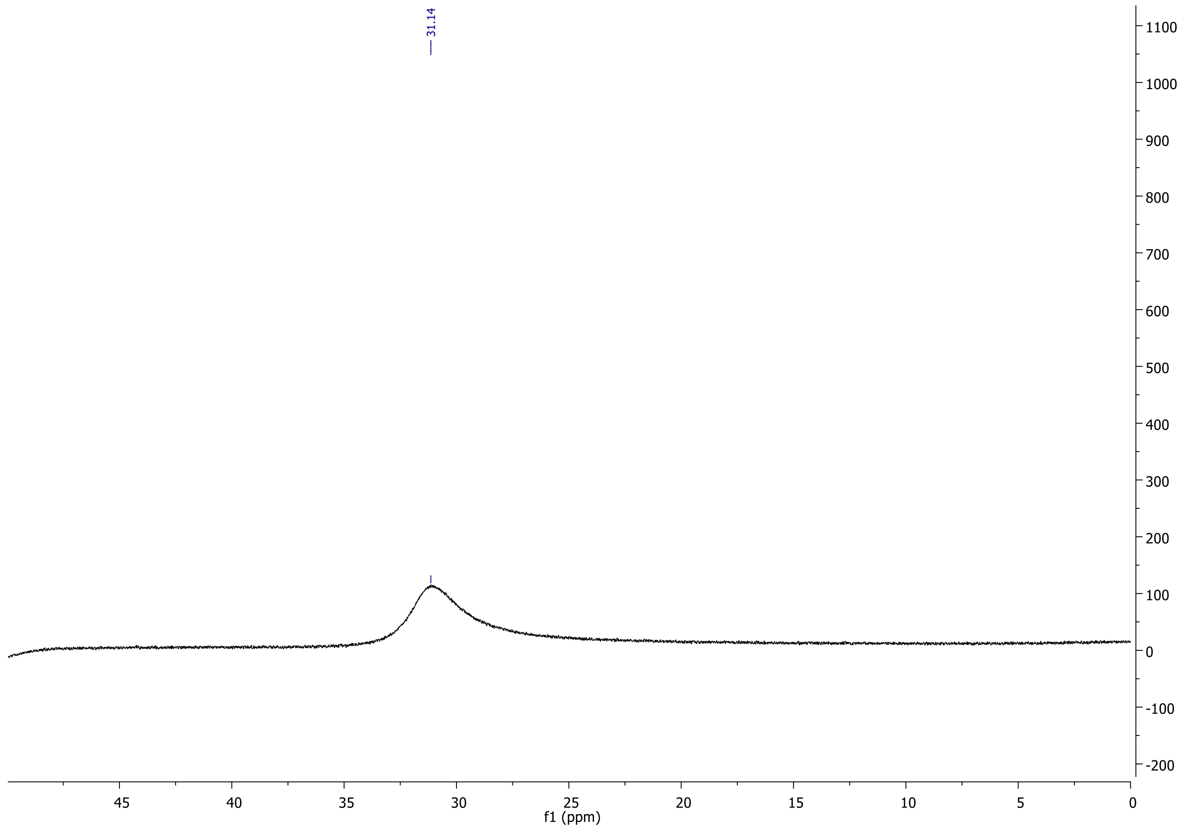

HRMS of 3f

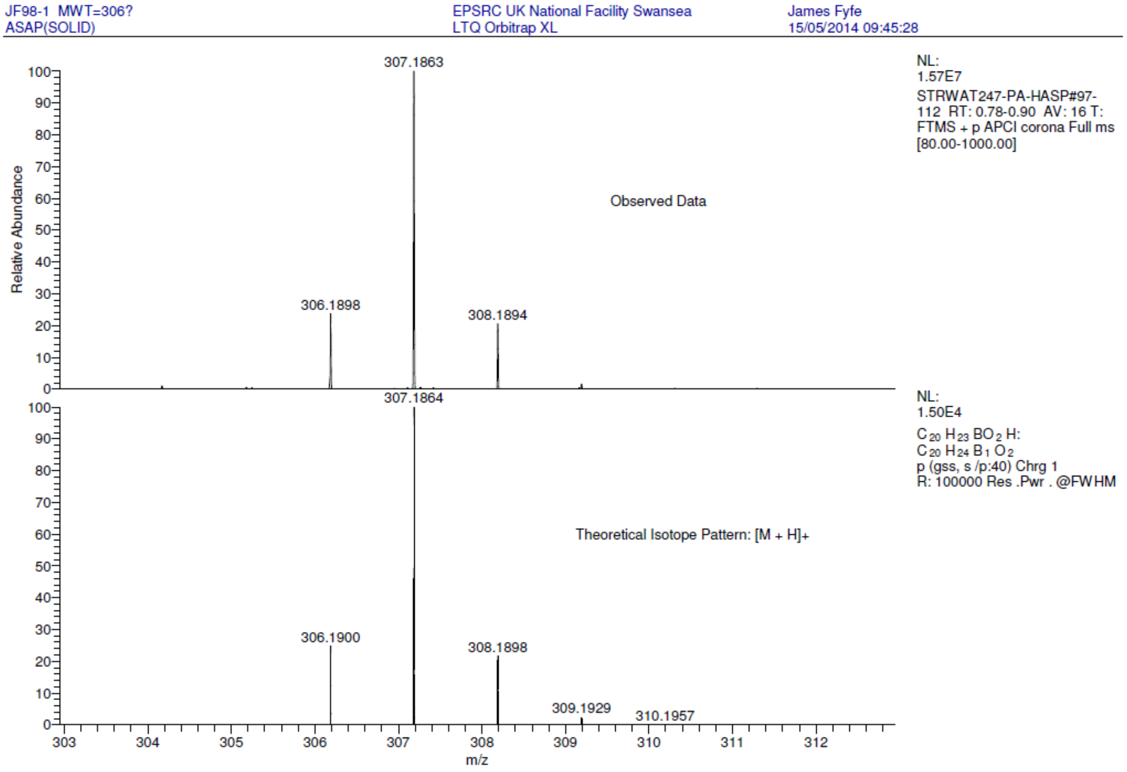

^{1}H NMR of 3g

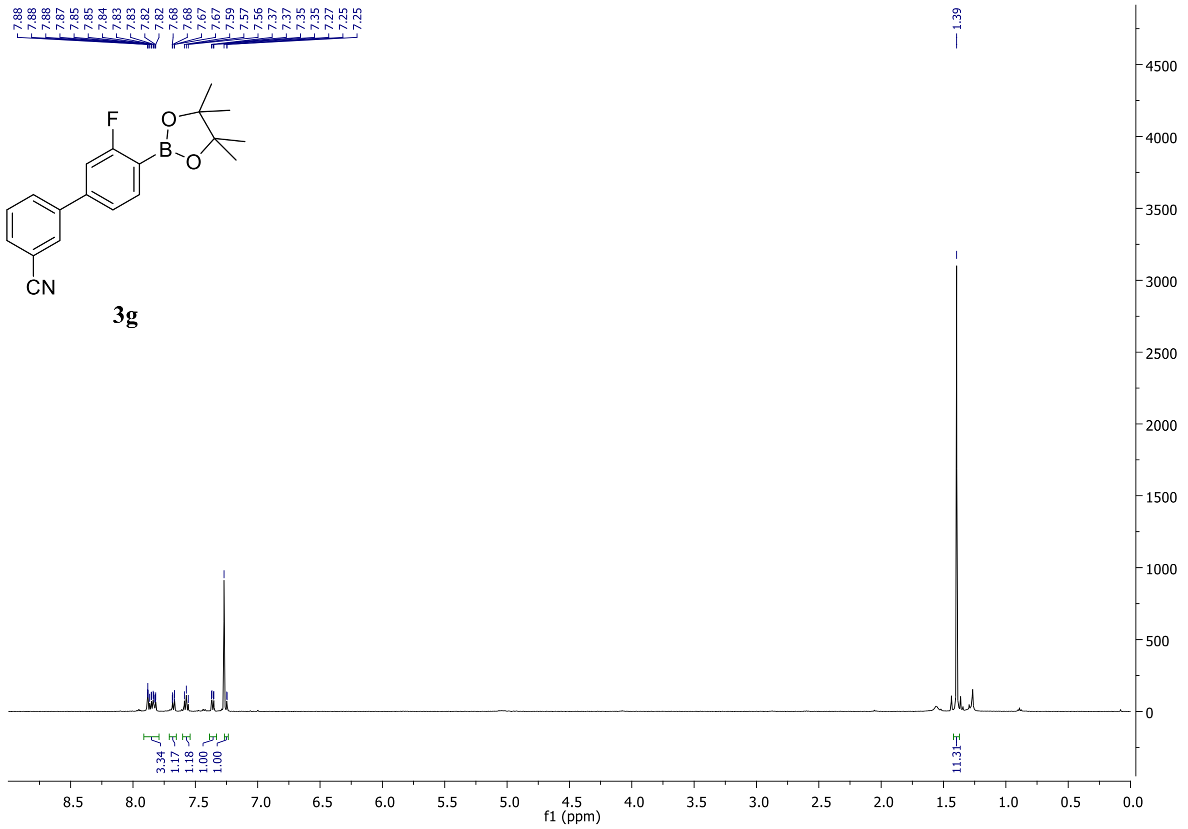

^{13}C NMR of 3g

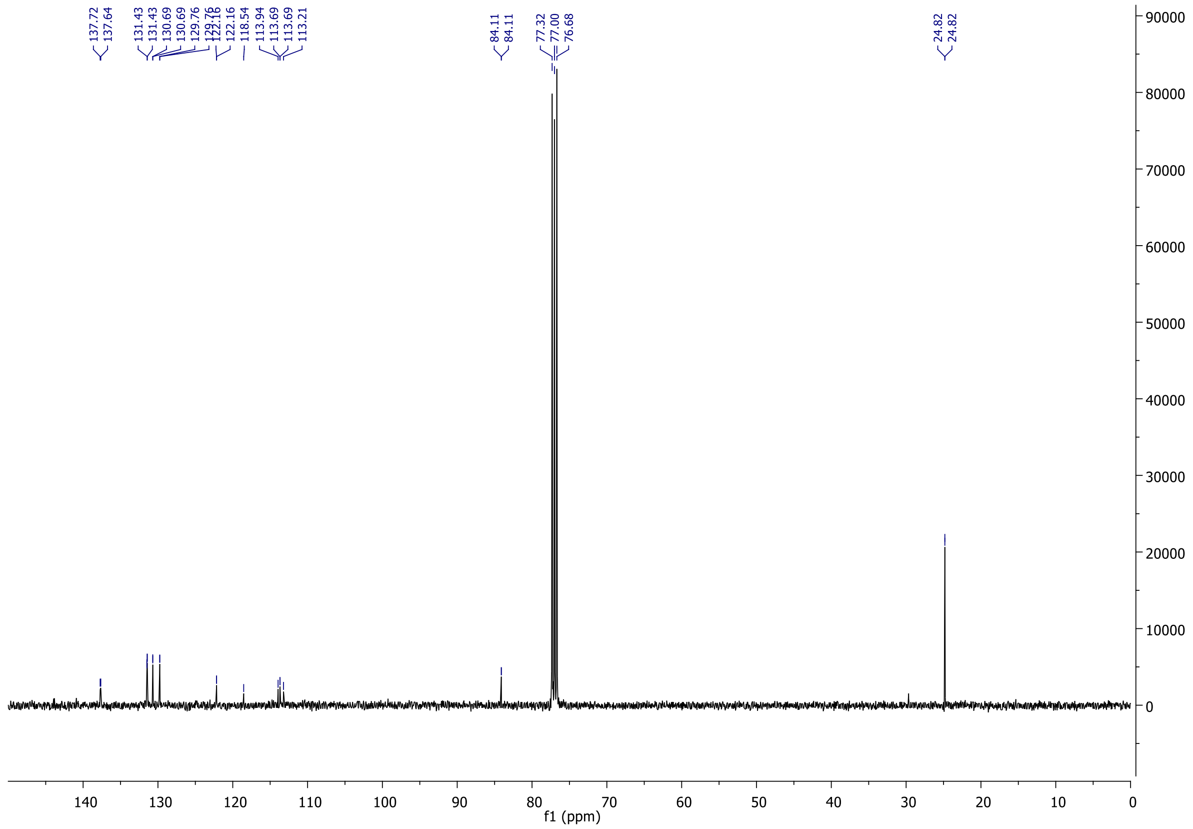

^{11}B NMR of 3g

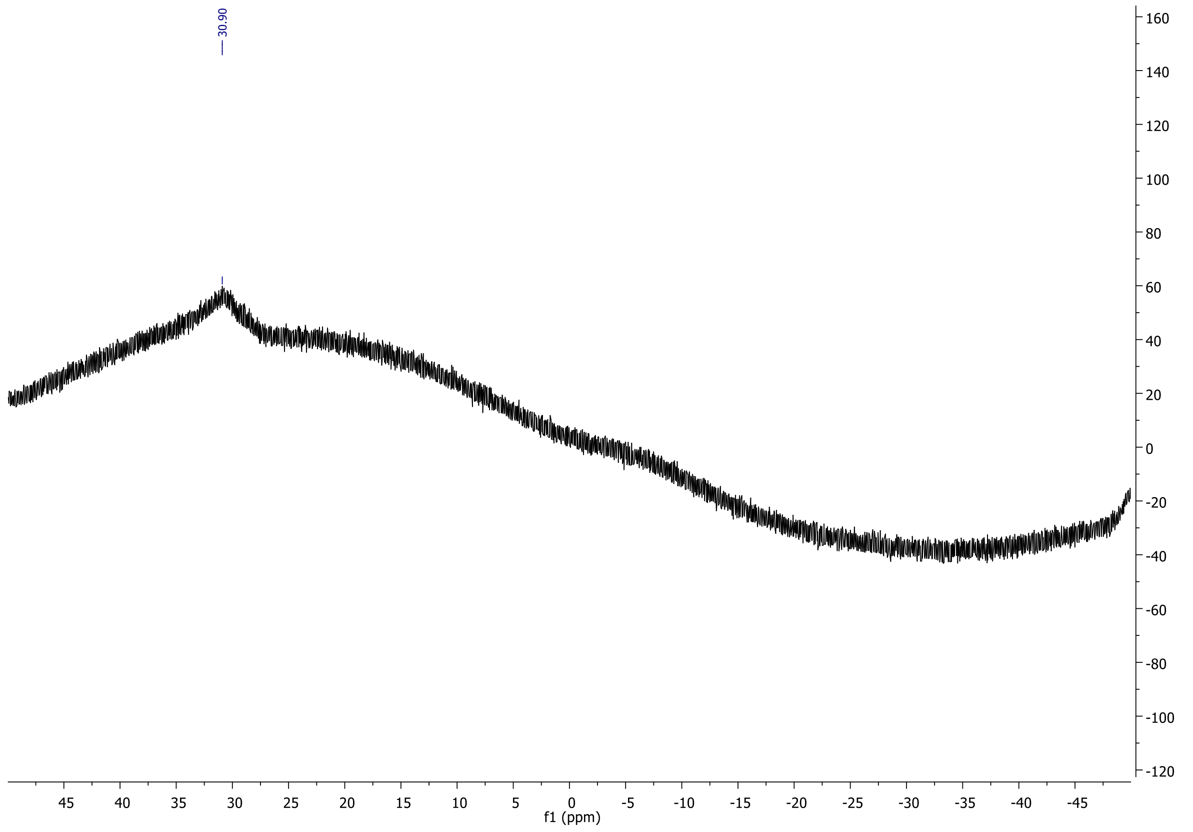

^{19}F NMR of 3g

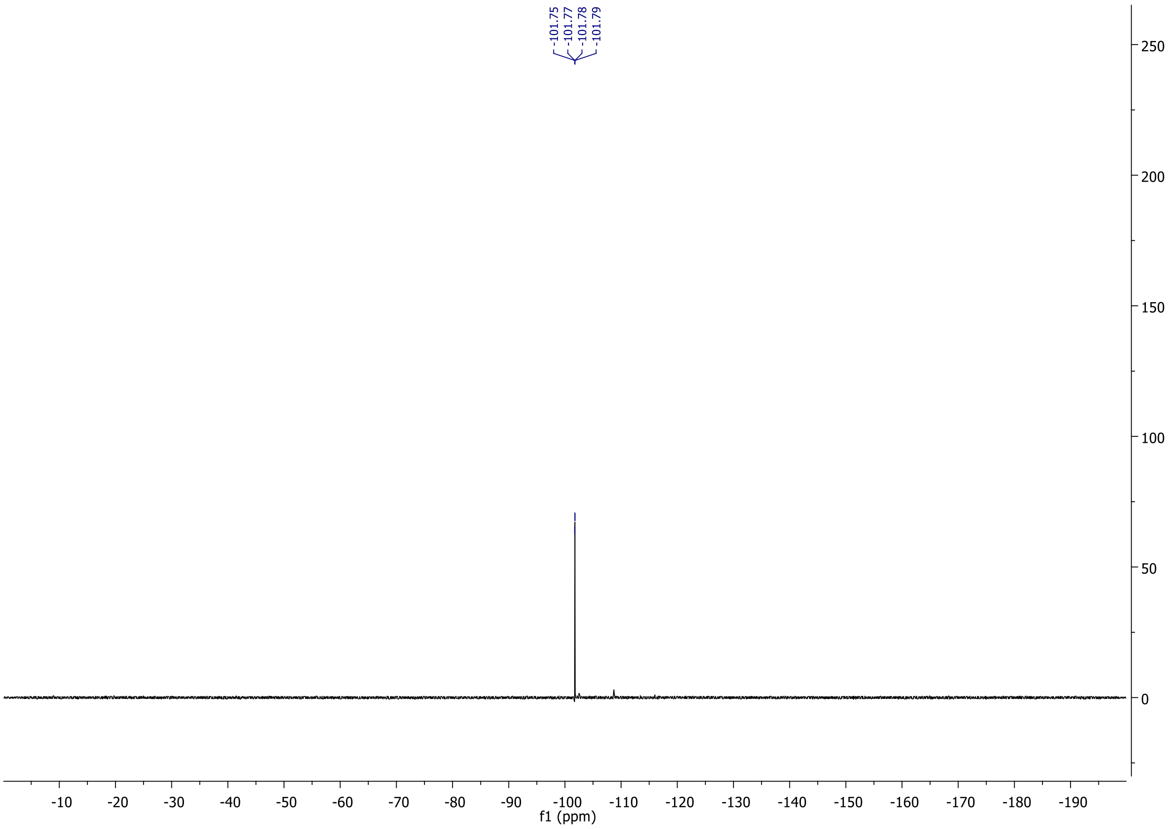

HRMS of 3g

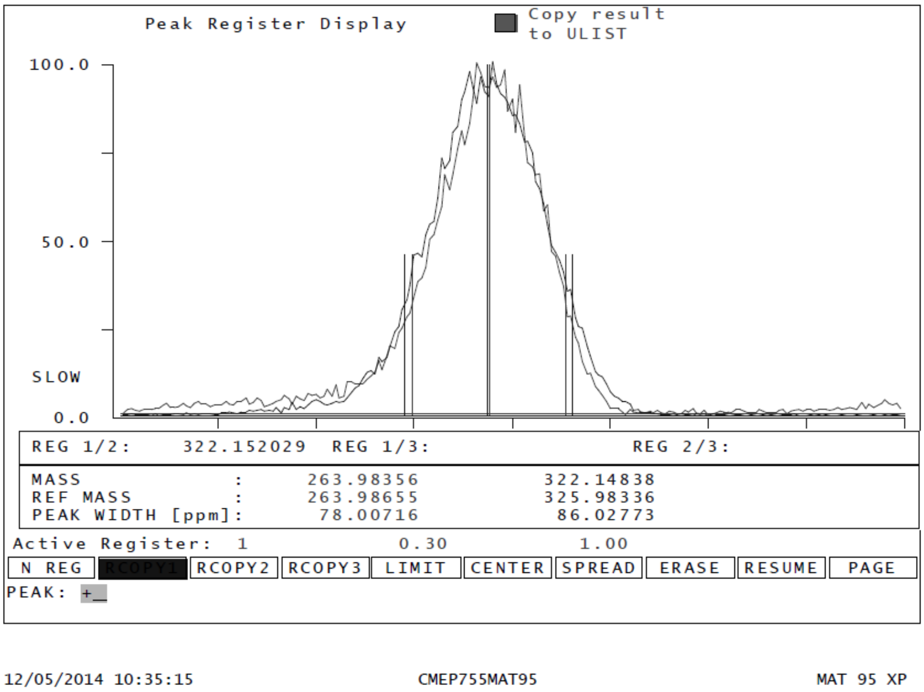

^{1}H NMR of 3h

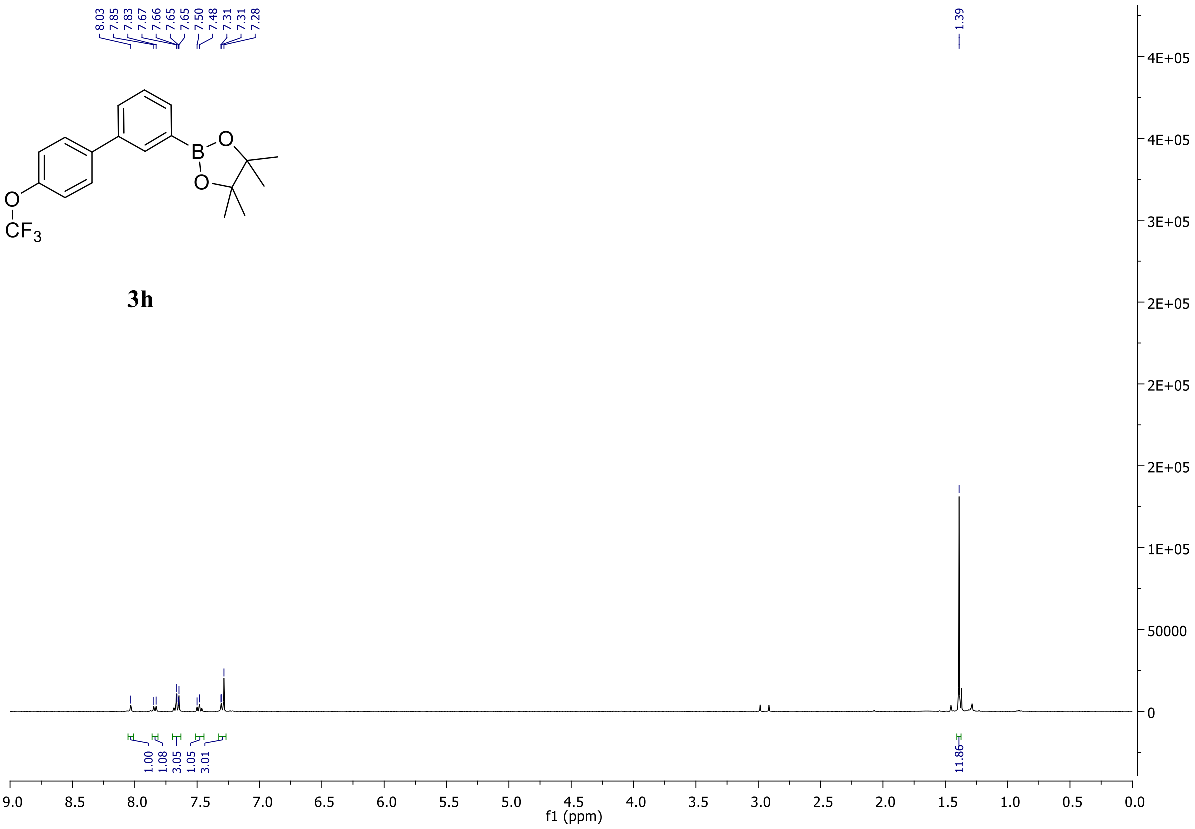

^{13}C NMR of 3h

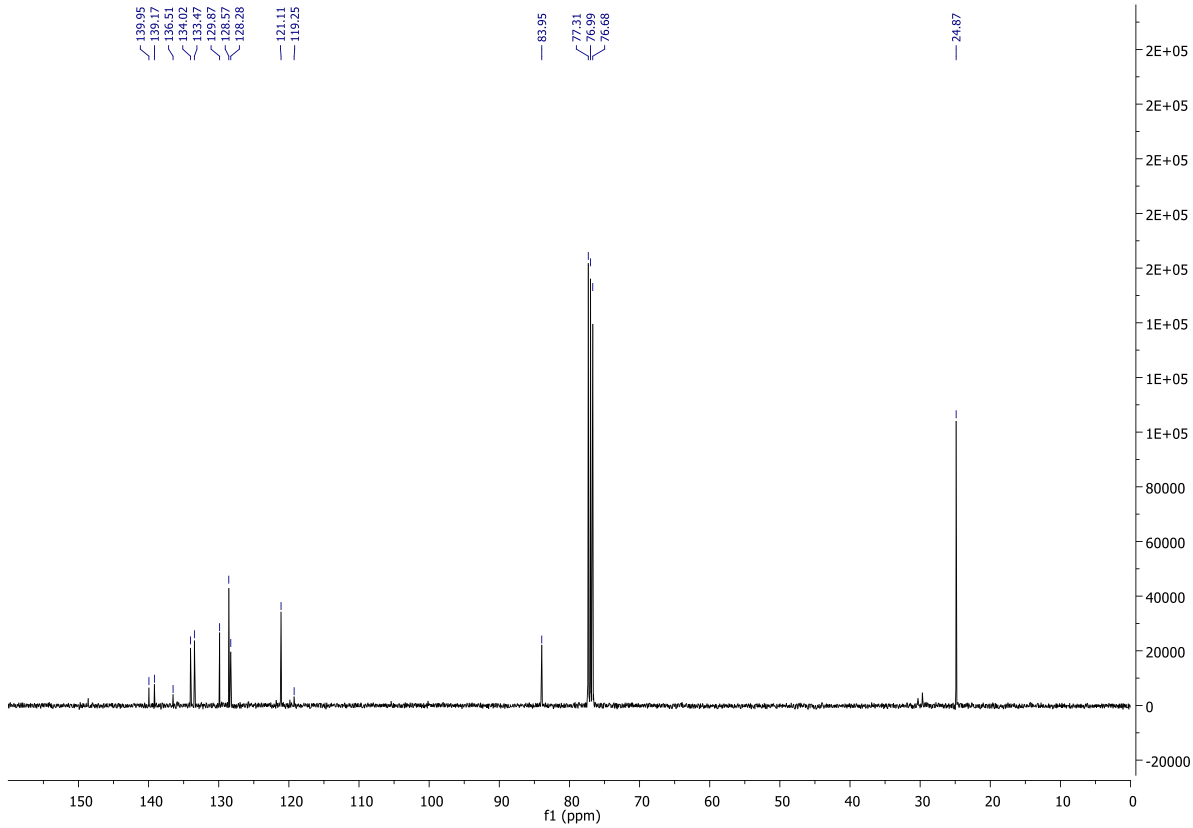

^{11}B NMR of 3h

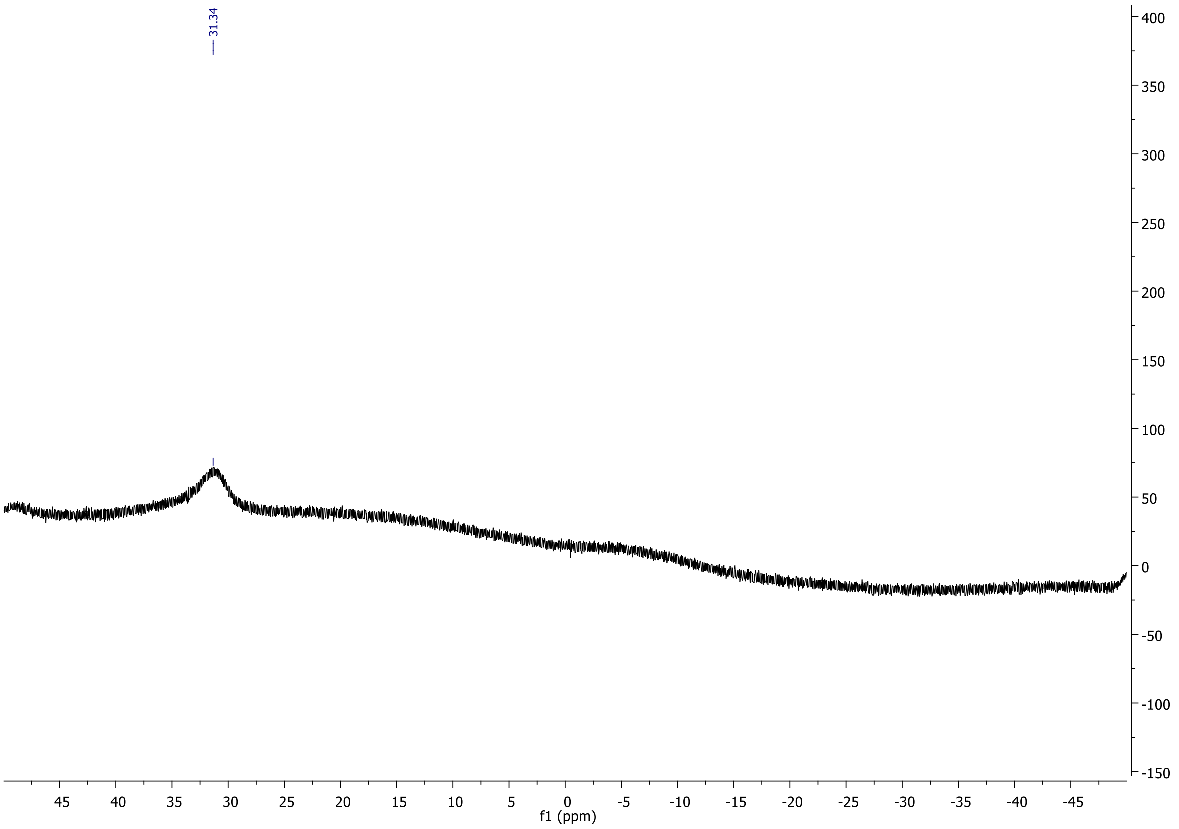

^{19}F NMR of 3h

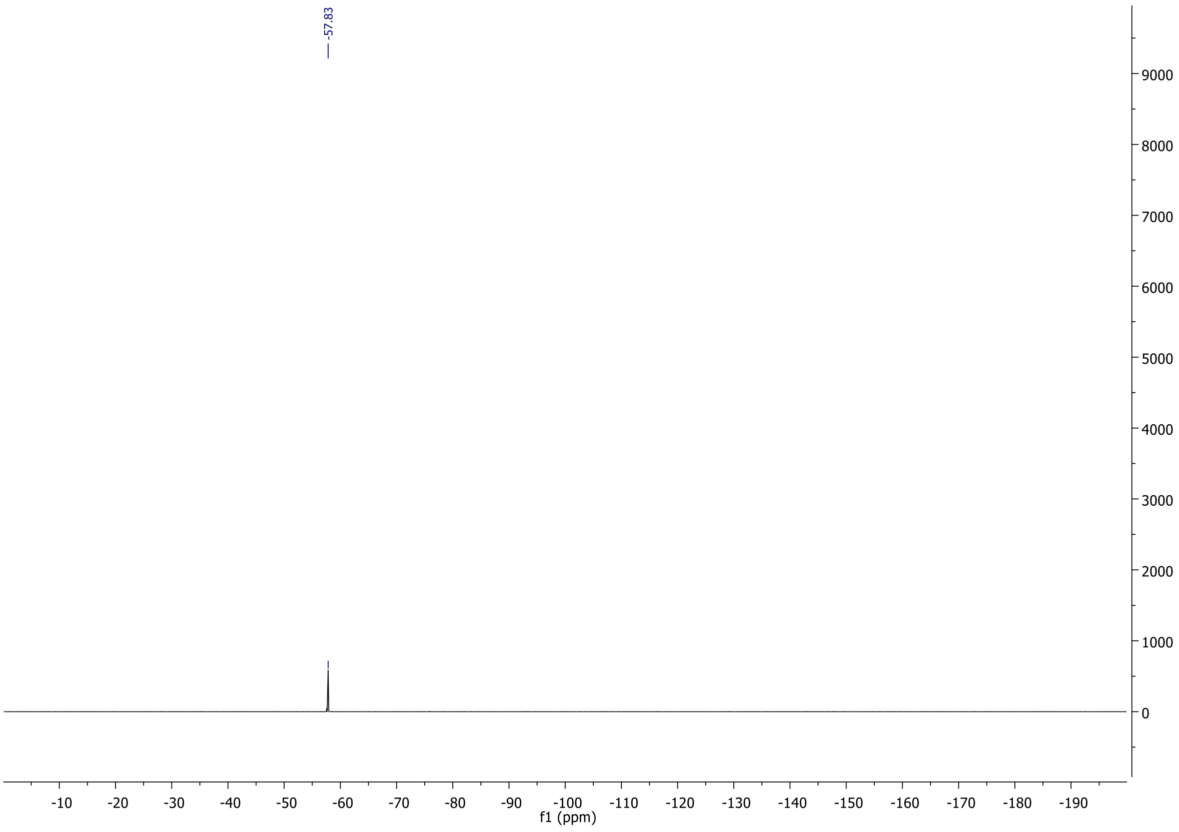

HRMS of 3h

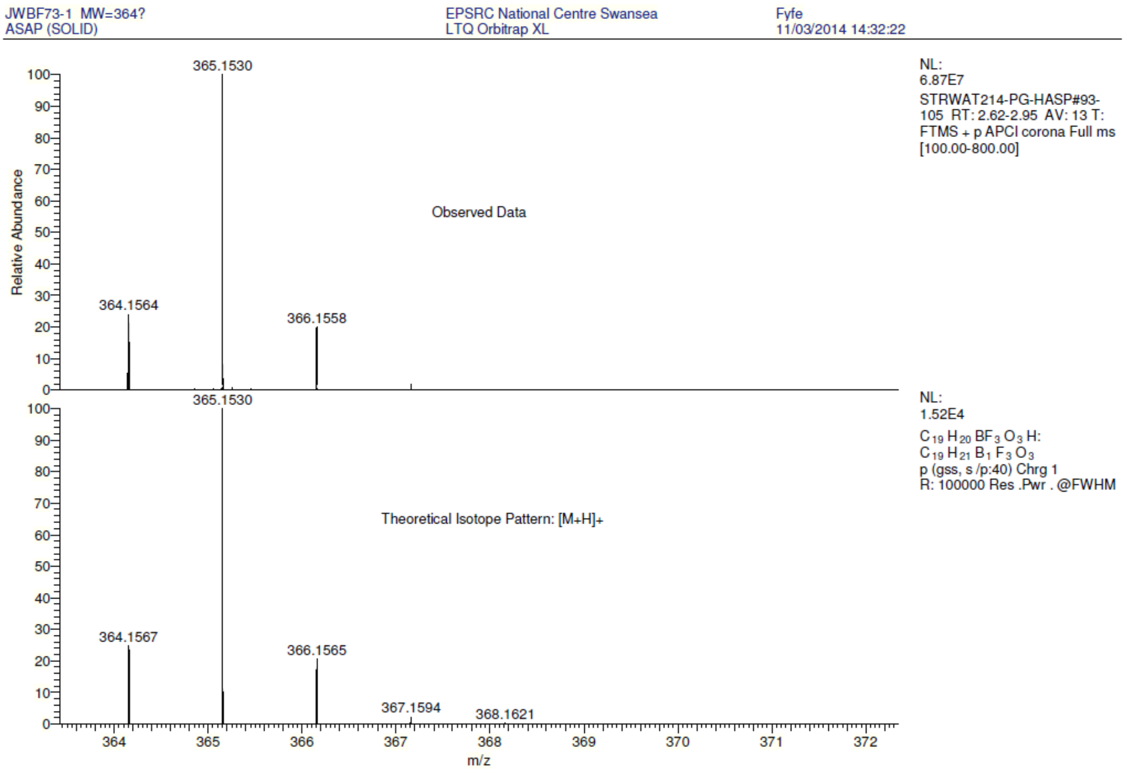

^{1}H NMR of 3i

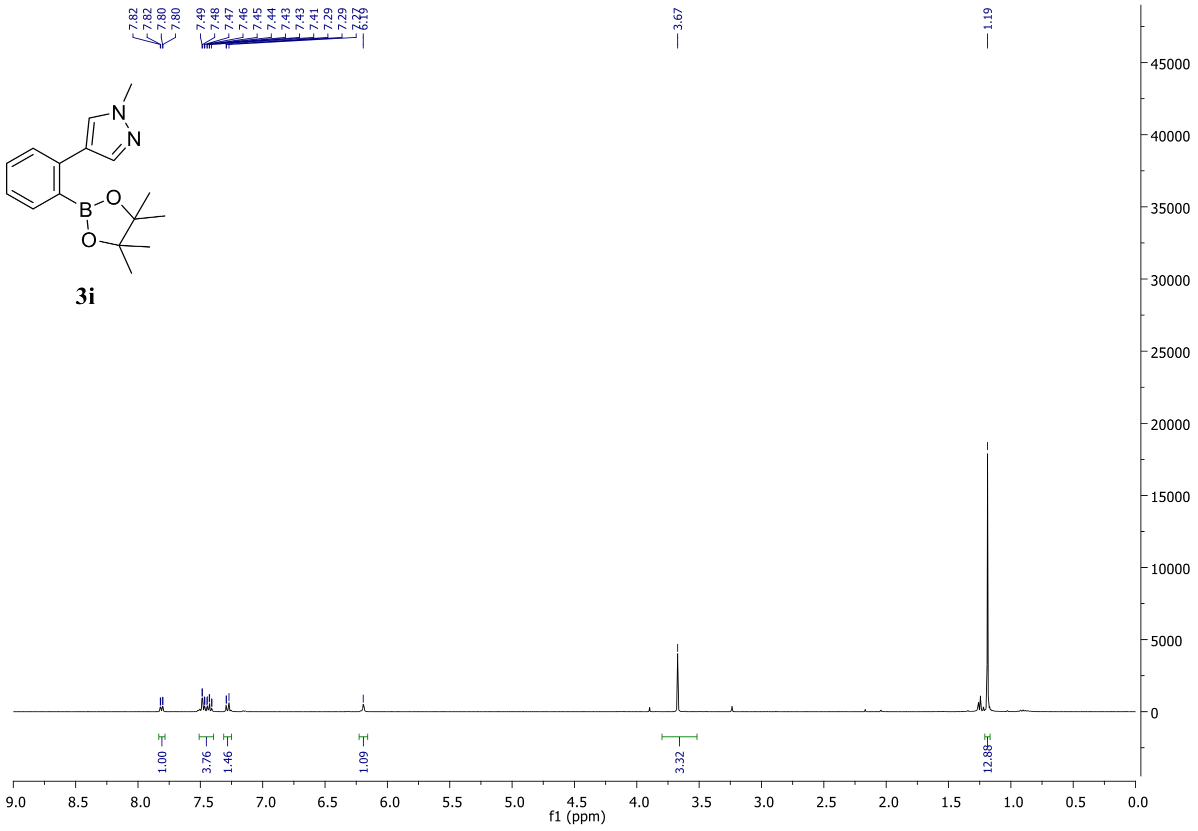

^{13}C NMR of 3i

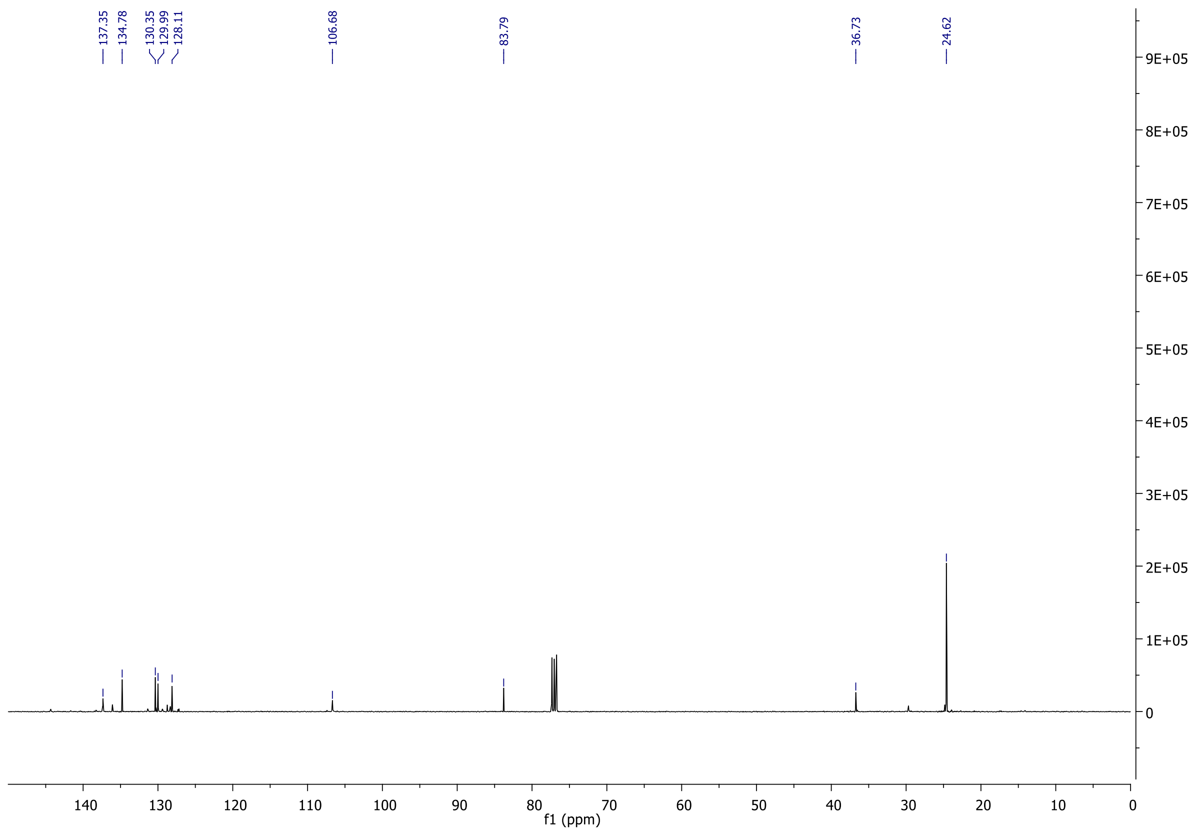

^{11}B NMR of 3i

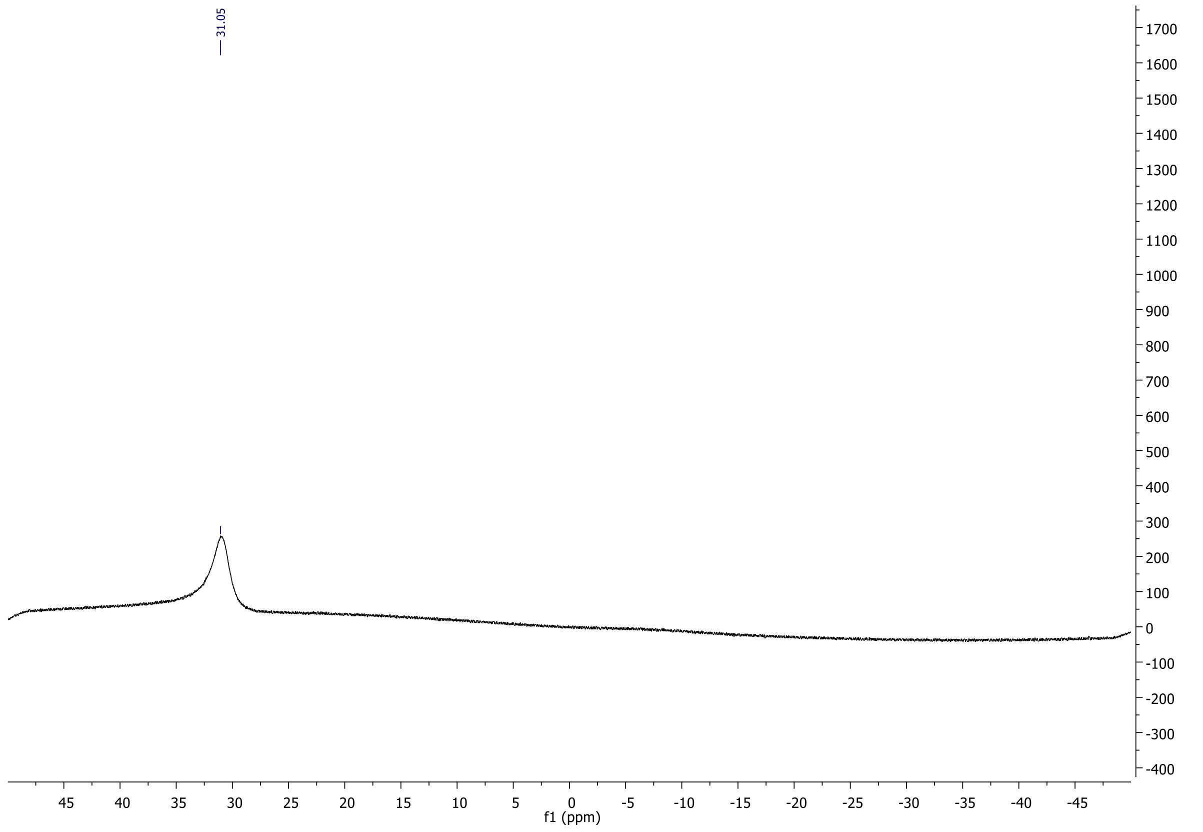

HRMS of 3i

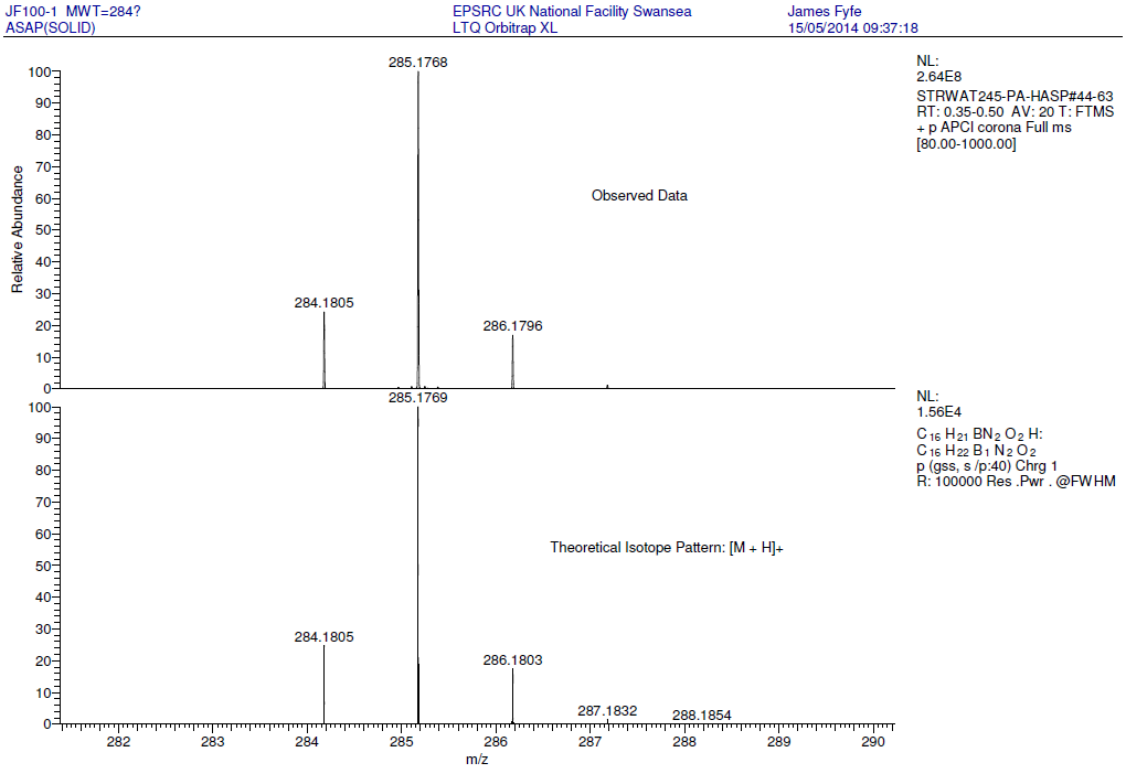

^{1}H NMR of 3j

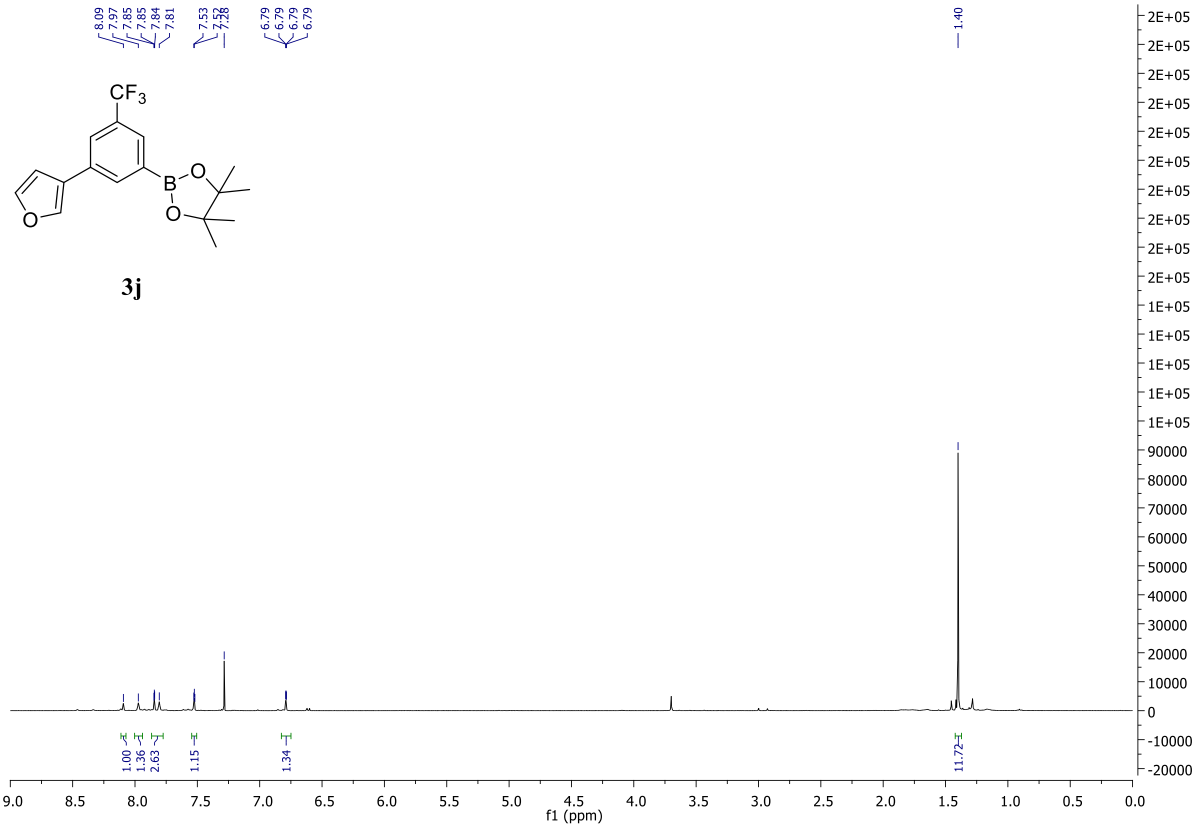

^{13}C NMR of 3j

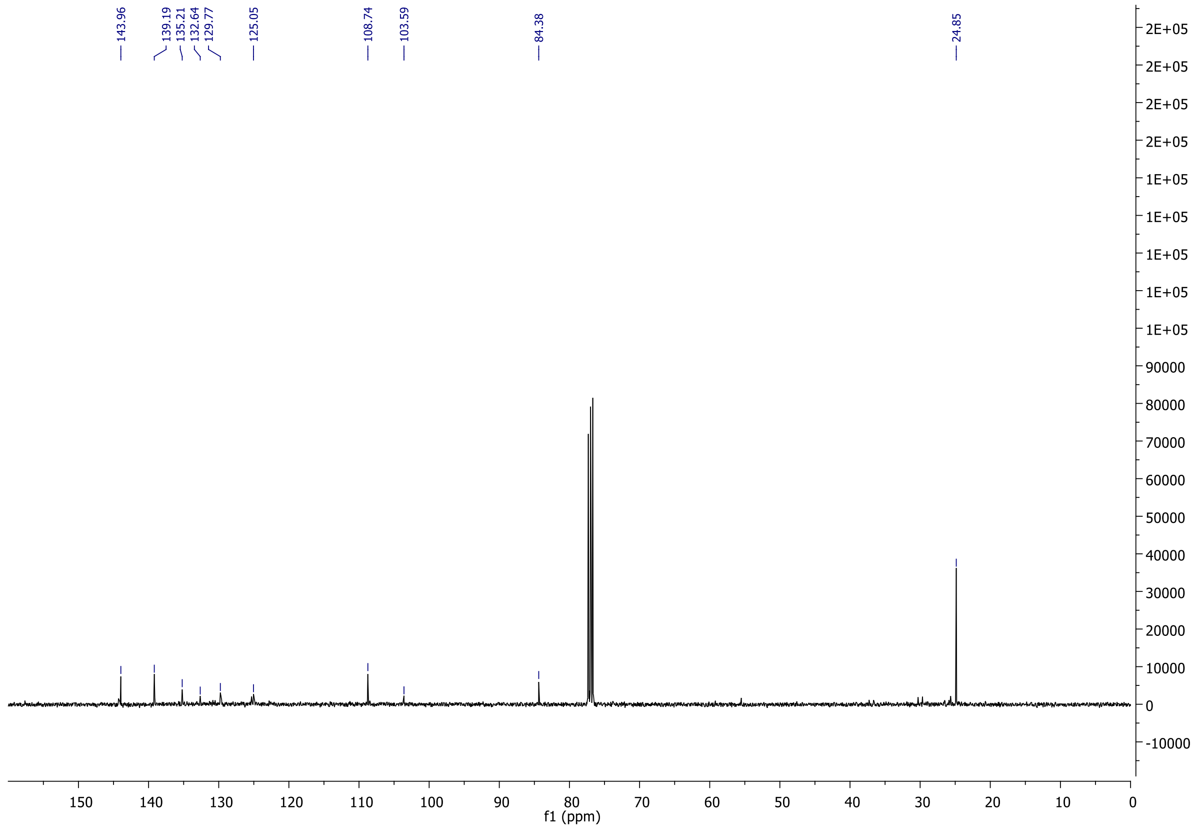

^{11}B NMR of 3j

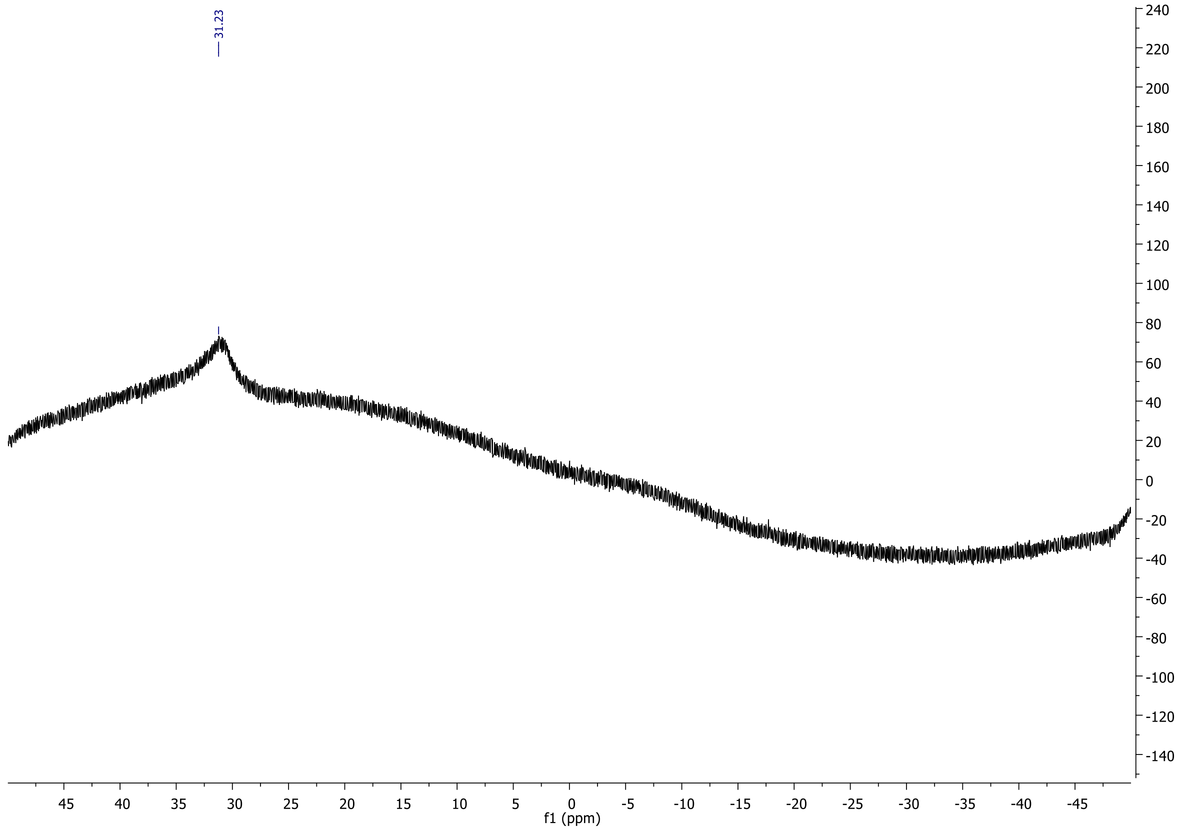

^{19}F NMR of 3j

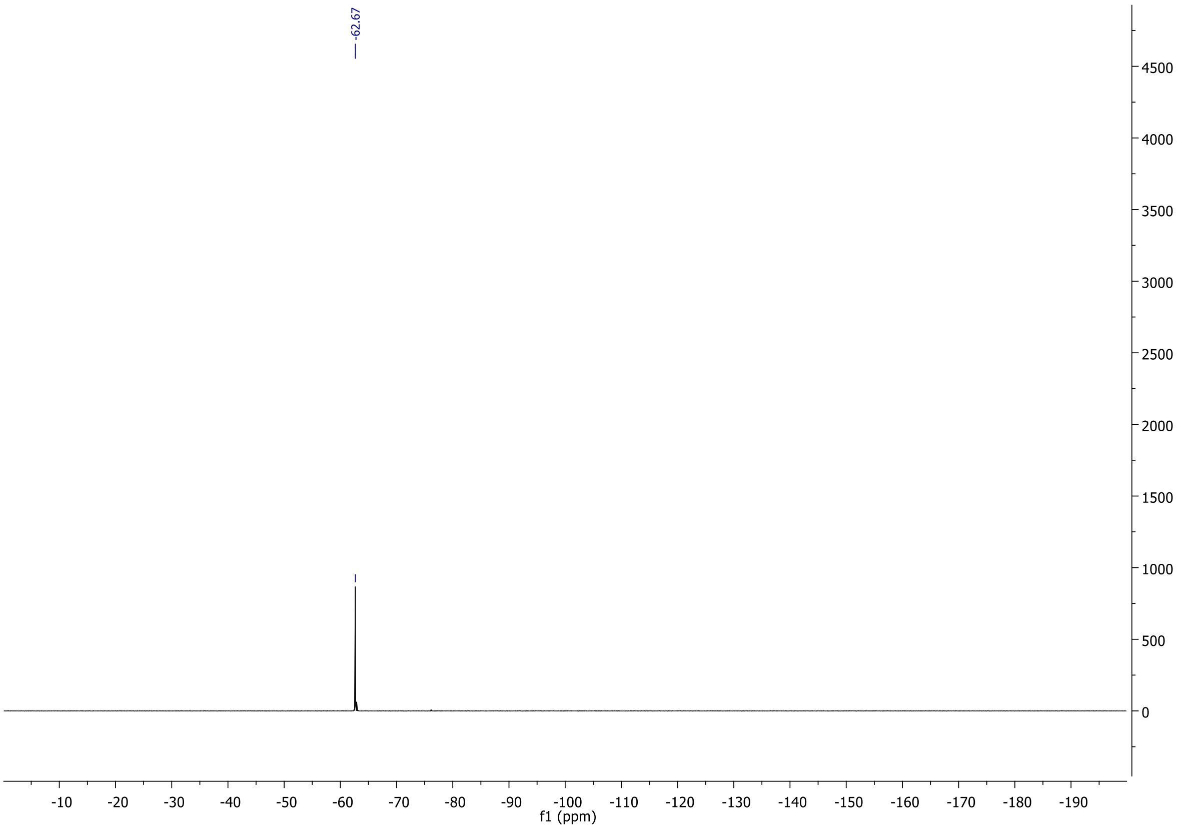

HRMS of 3j

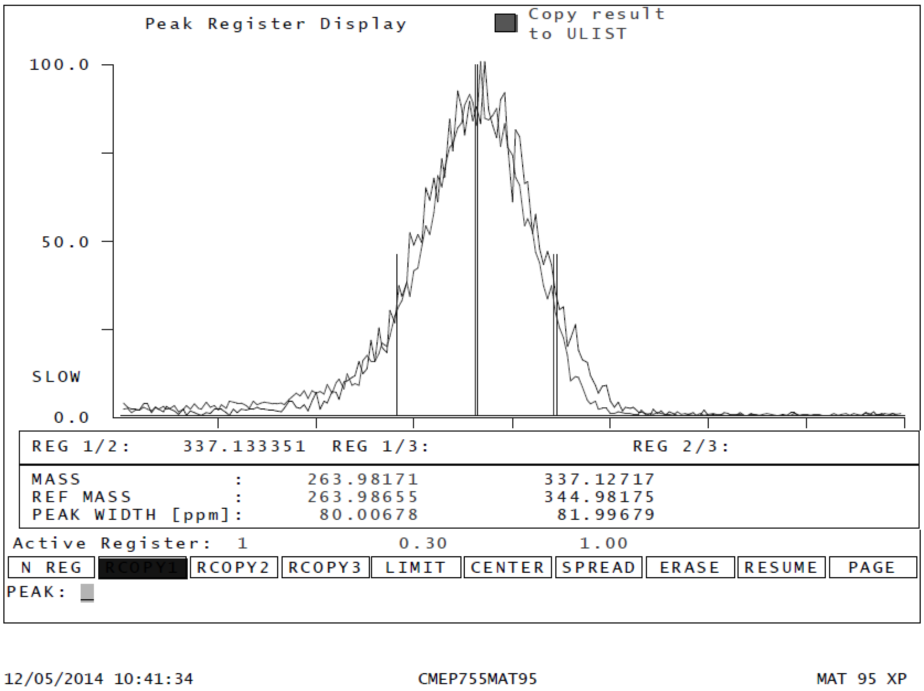

^{1}H NMR of 3k

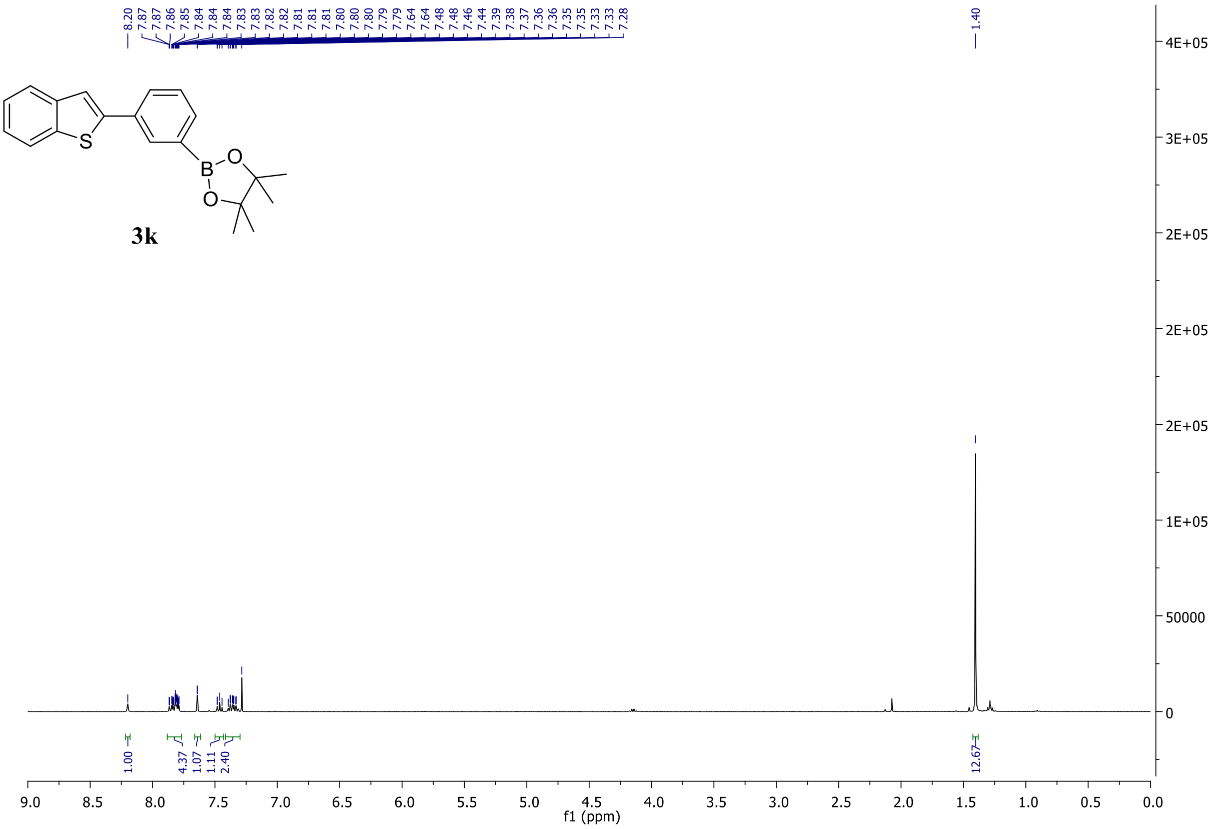

^{13}C NMR of 3k

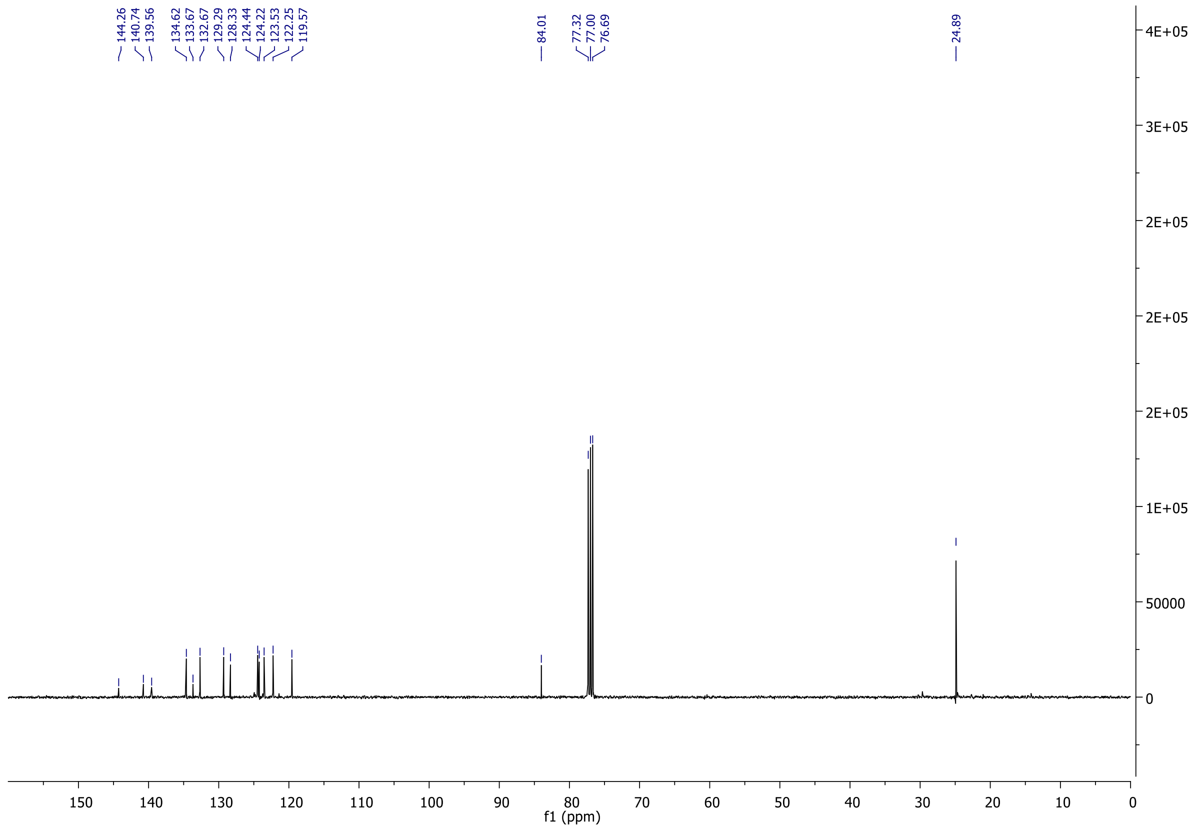

^{11}B NMR of 3k

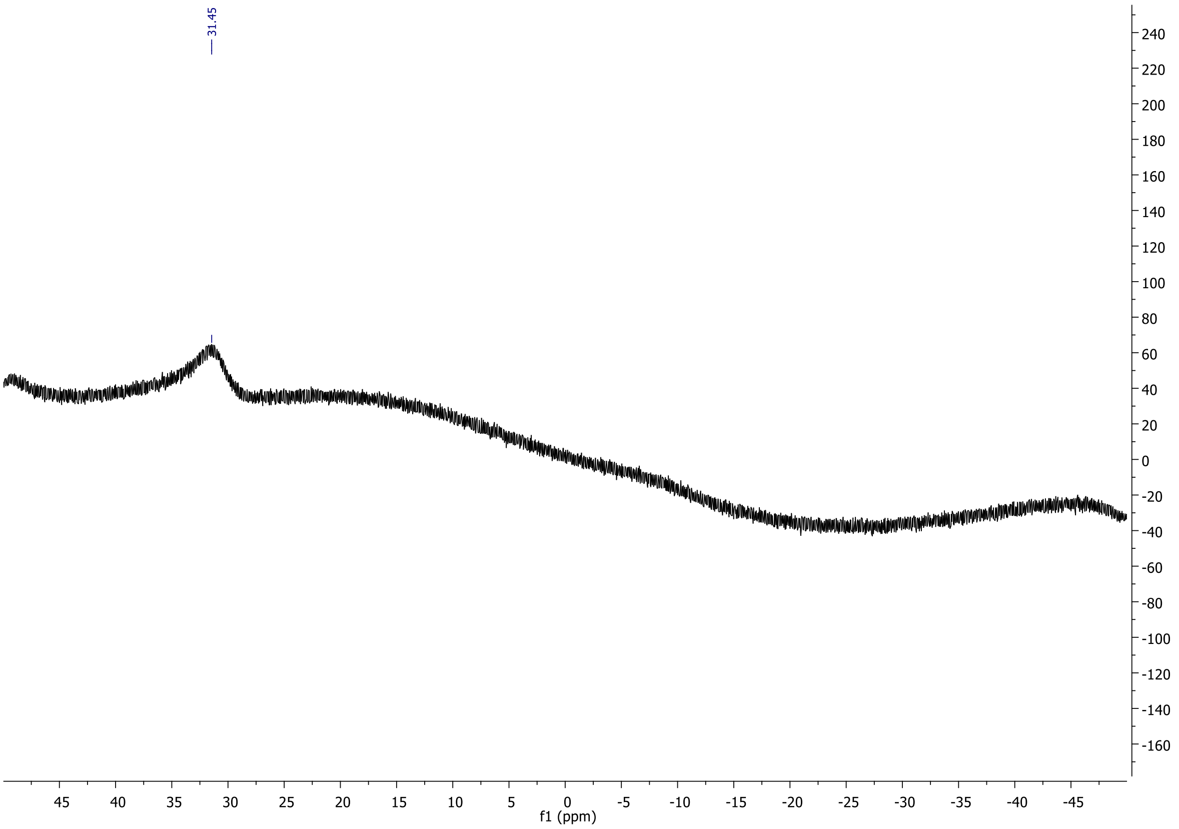

HRMS of 3k

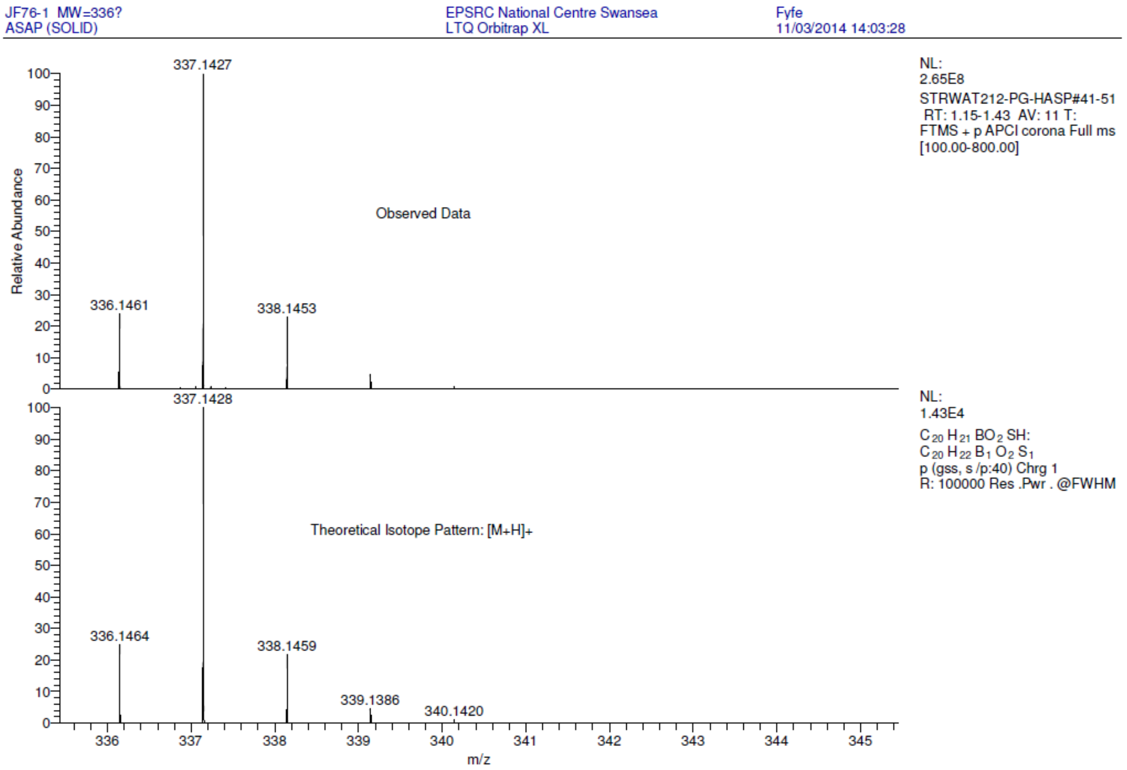

^{1}H NMR of 3l

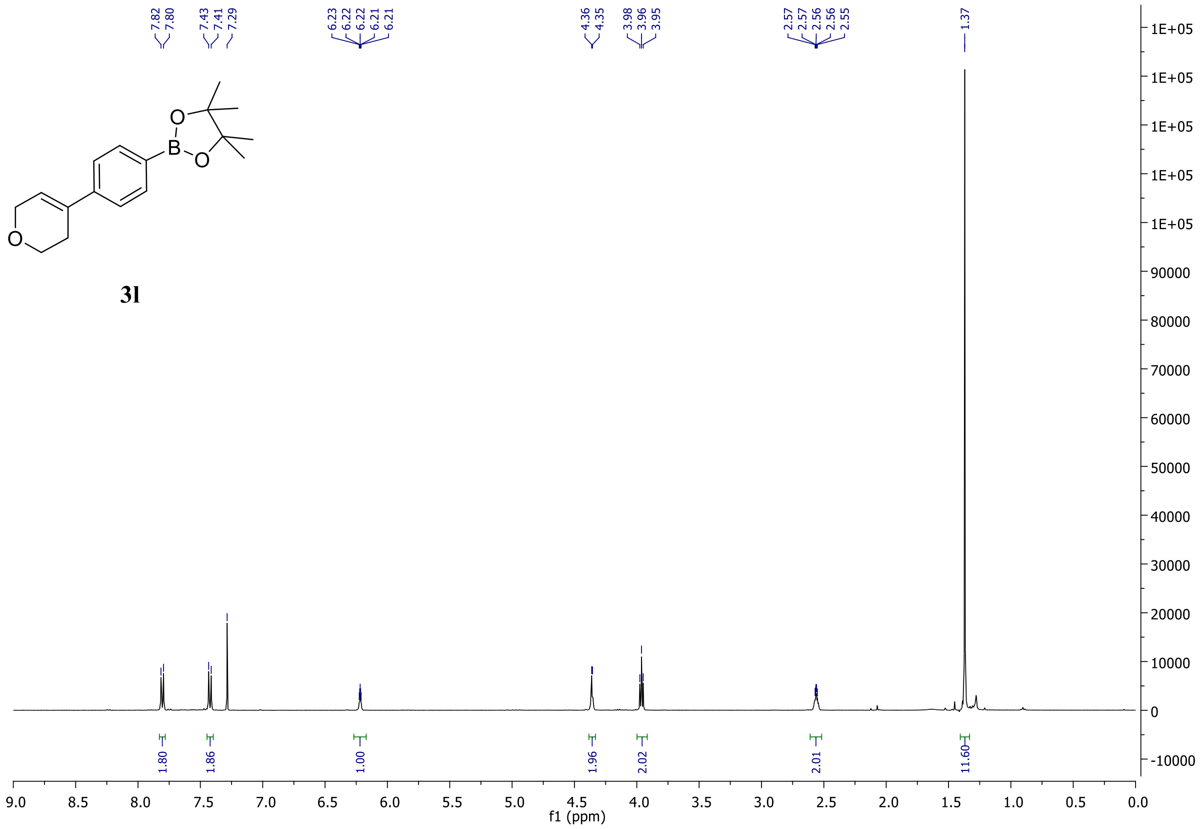

^{13}C NMR of 3l

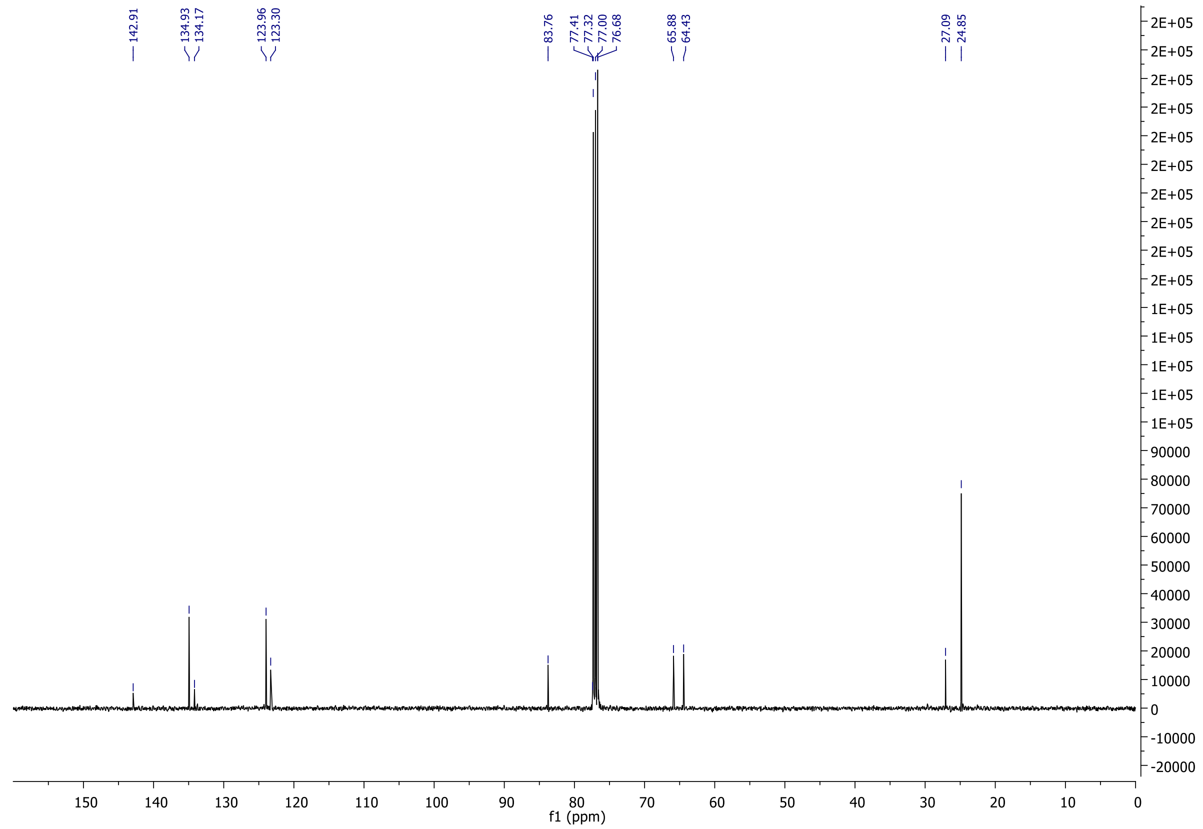

^{11}B NMR of 3l

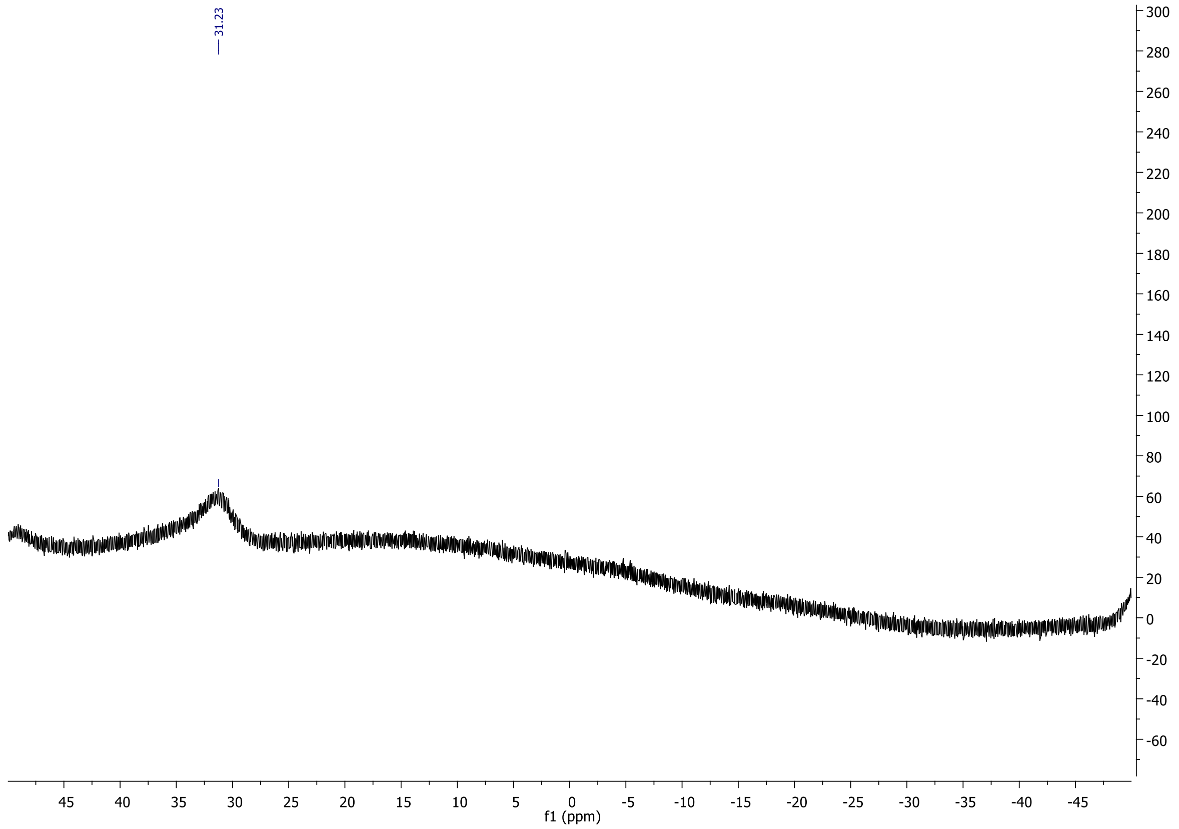

HRMS of 3l

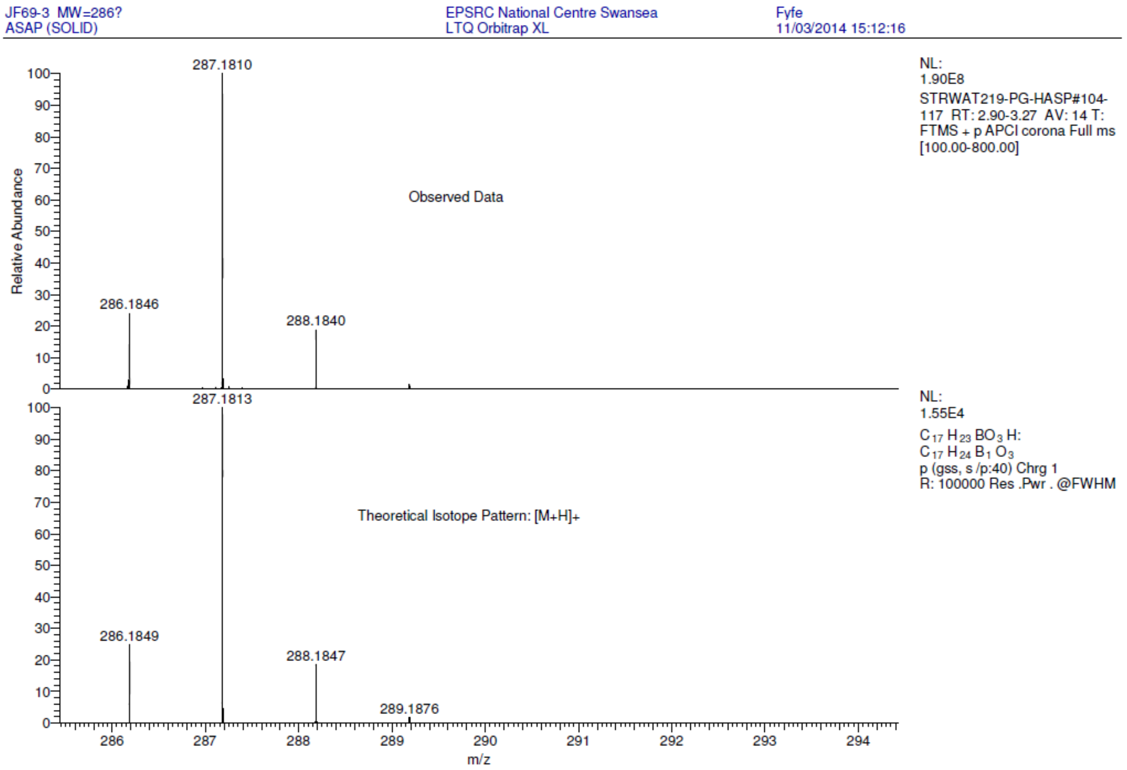

^{1}H NMR of 3m

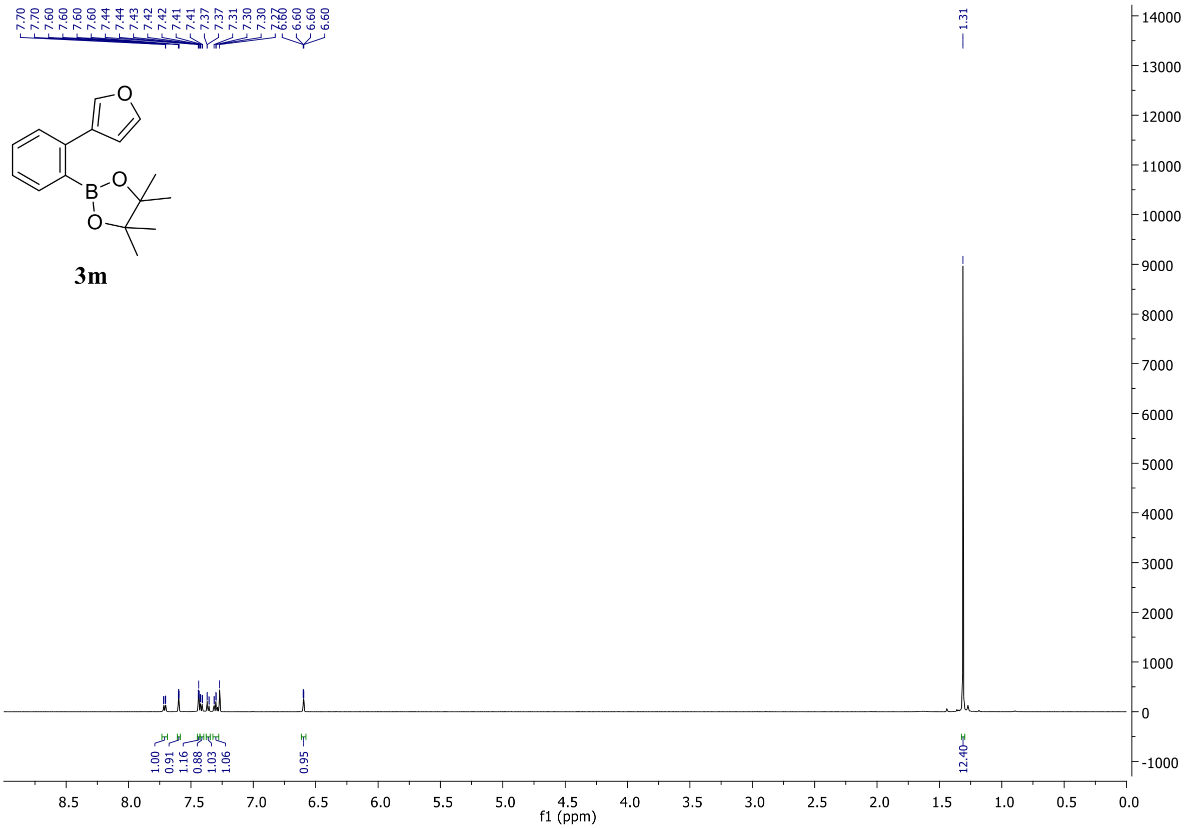

^{13}C NMR of 3m

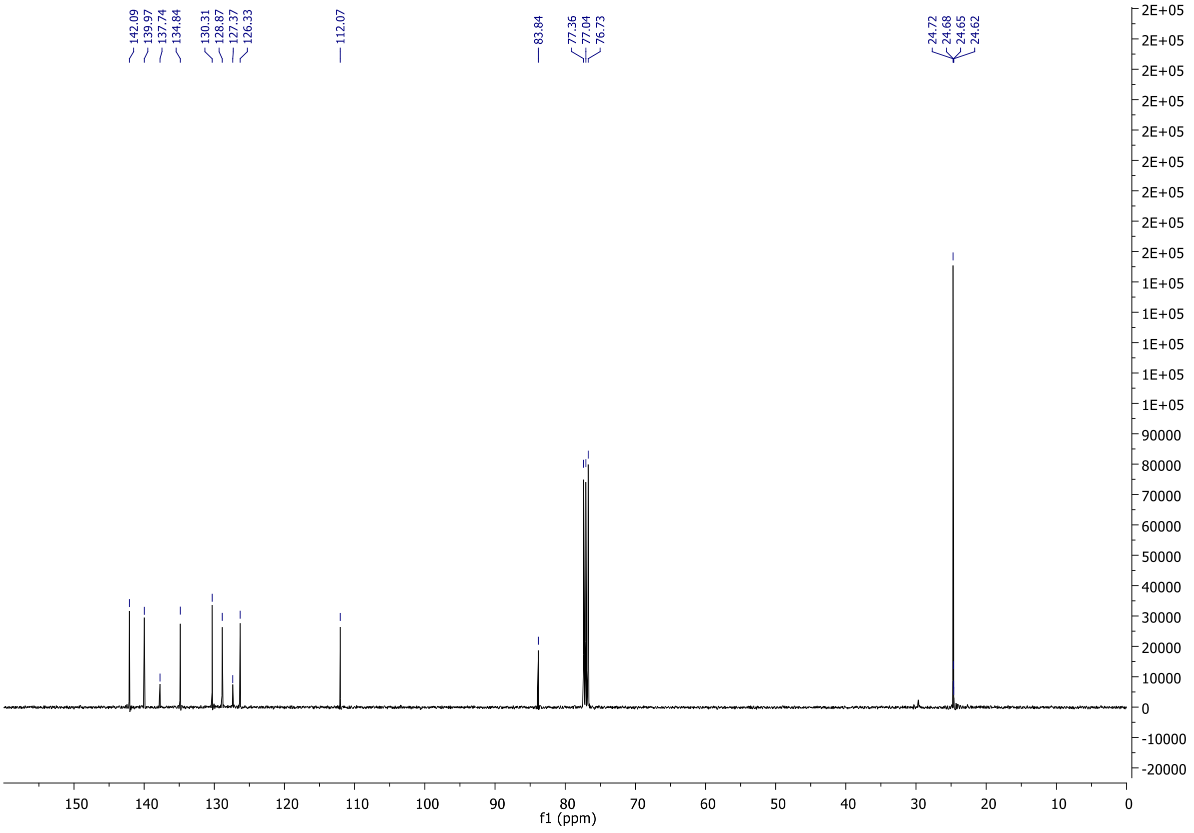

^{11}B NMR of 3m

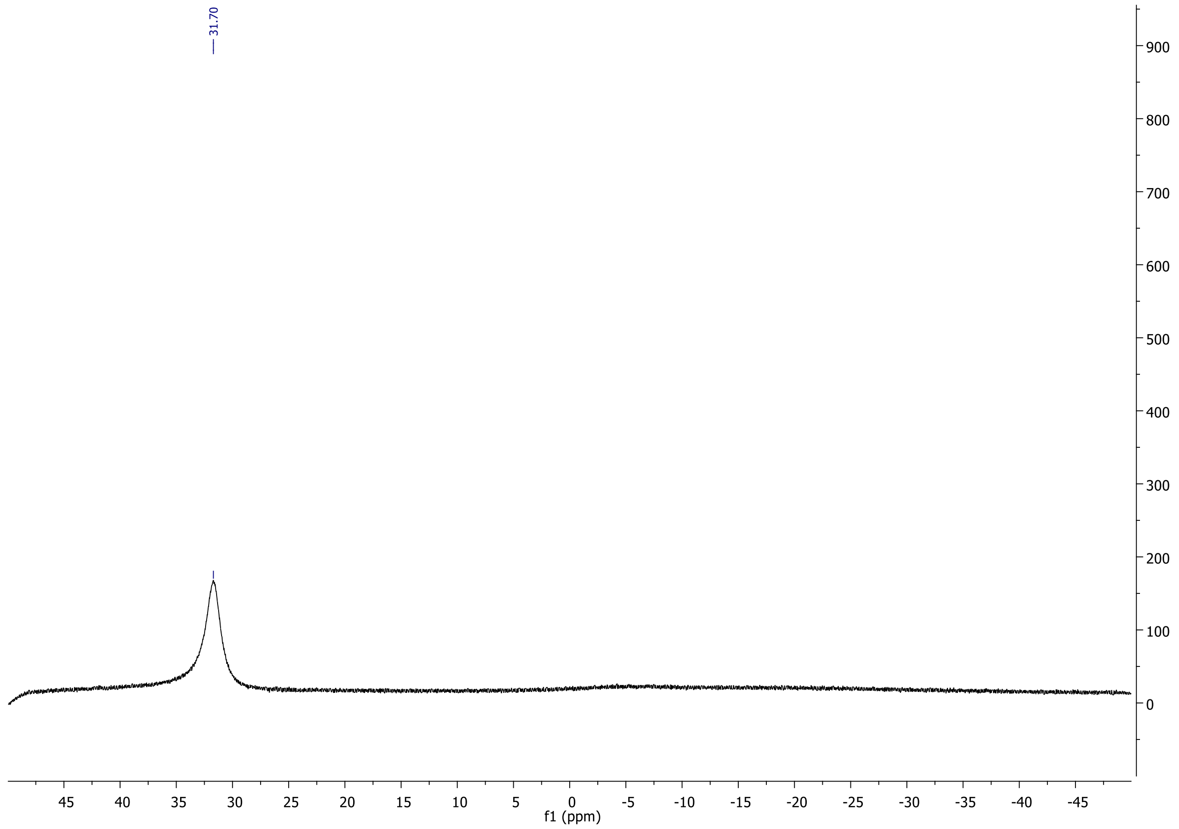

HRMS of 3m

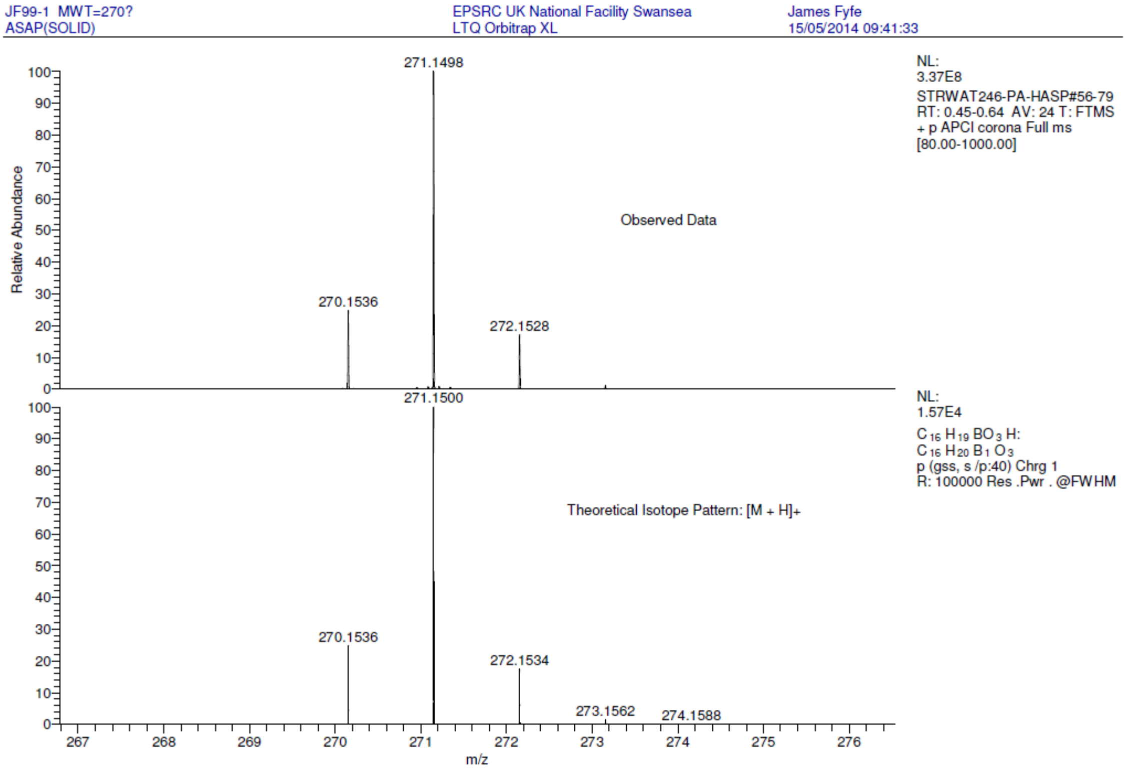

^{1}H NMR of 3n

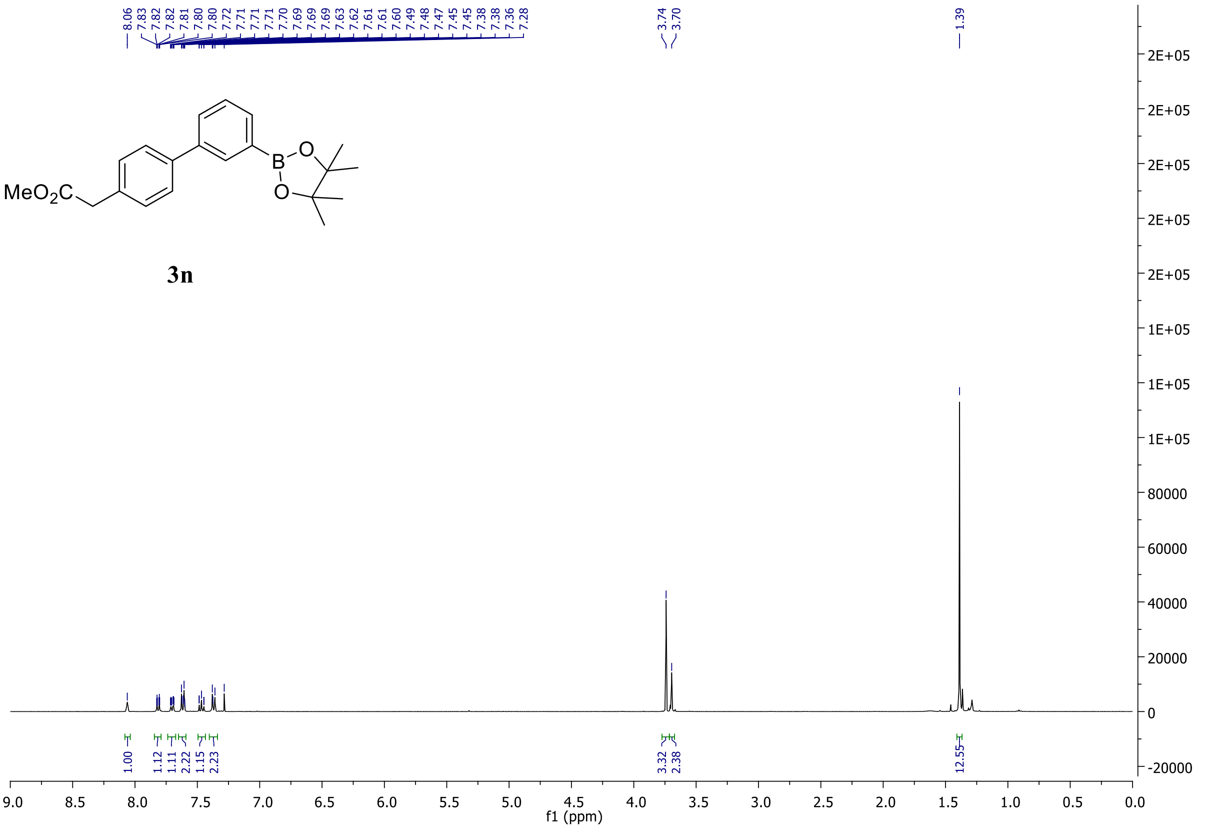

^{13}C NMR of 3n

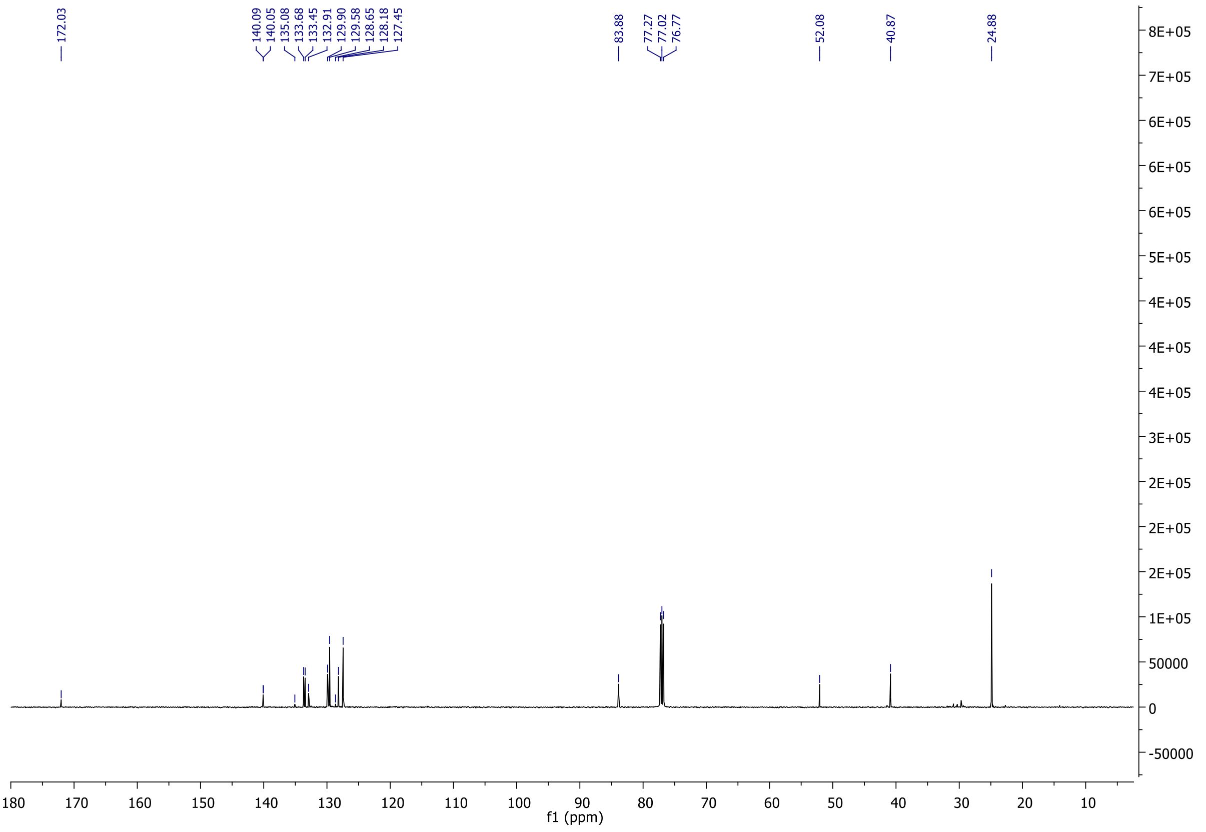

^{11}B NMR of 3n

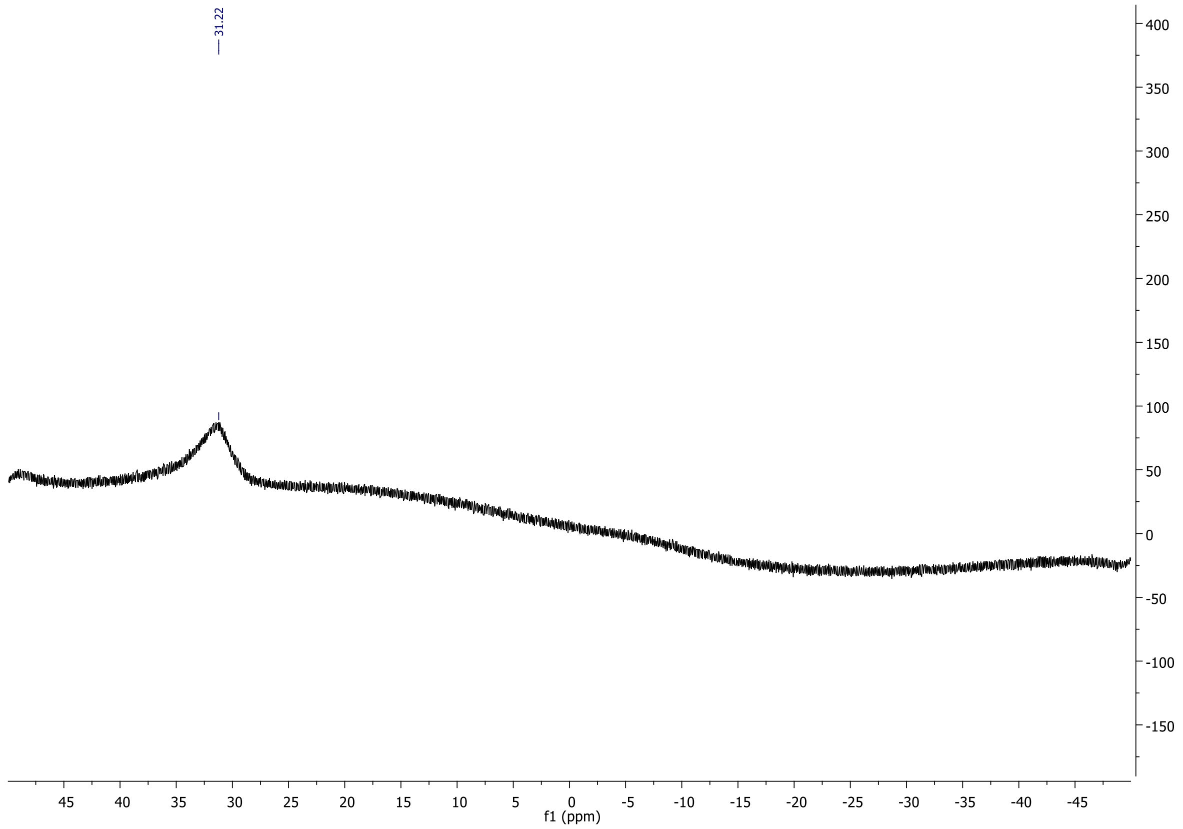

HRMS of 3n

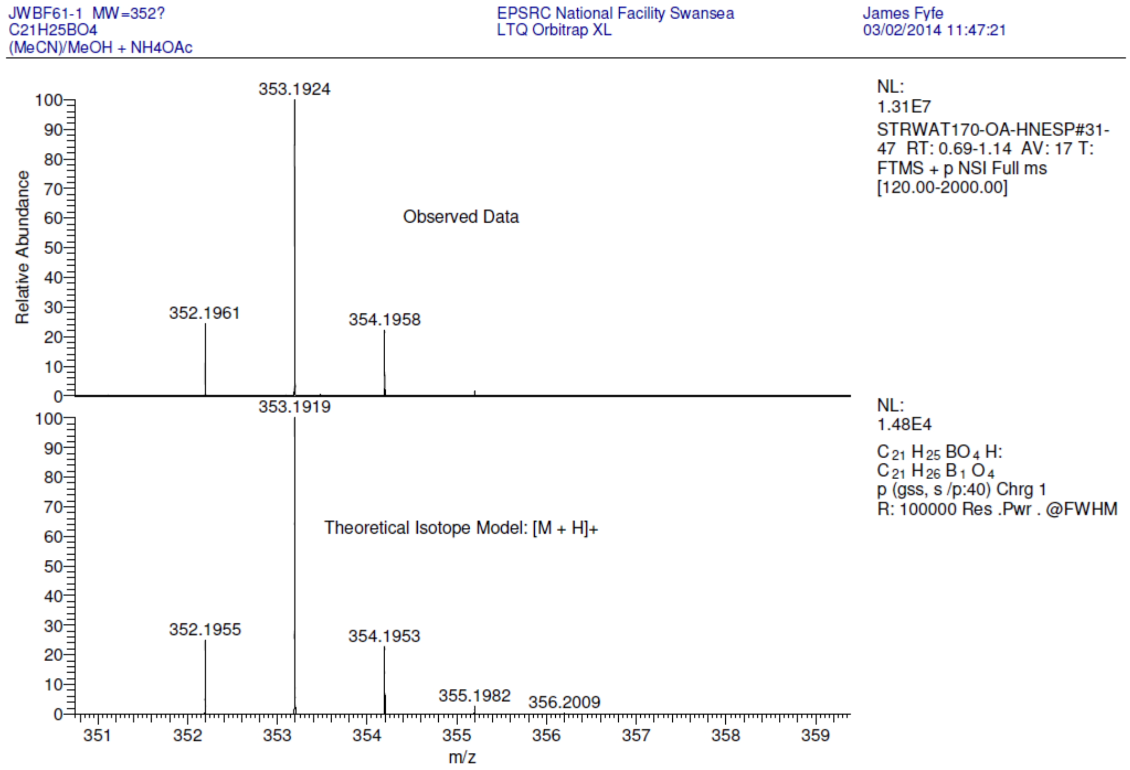

^{1}H NMR of 3o

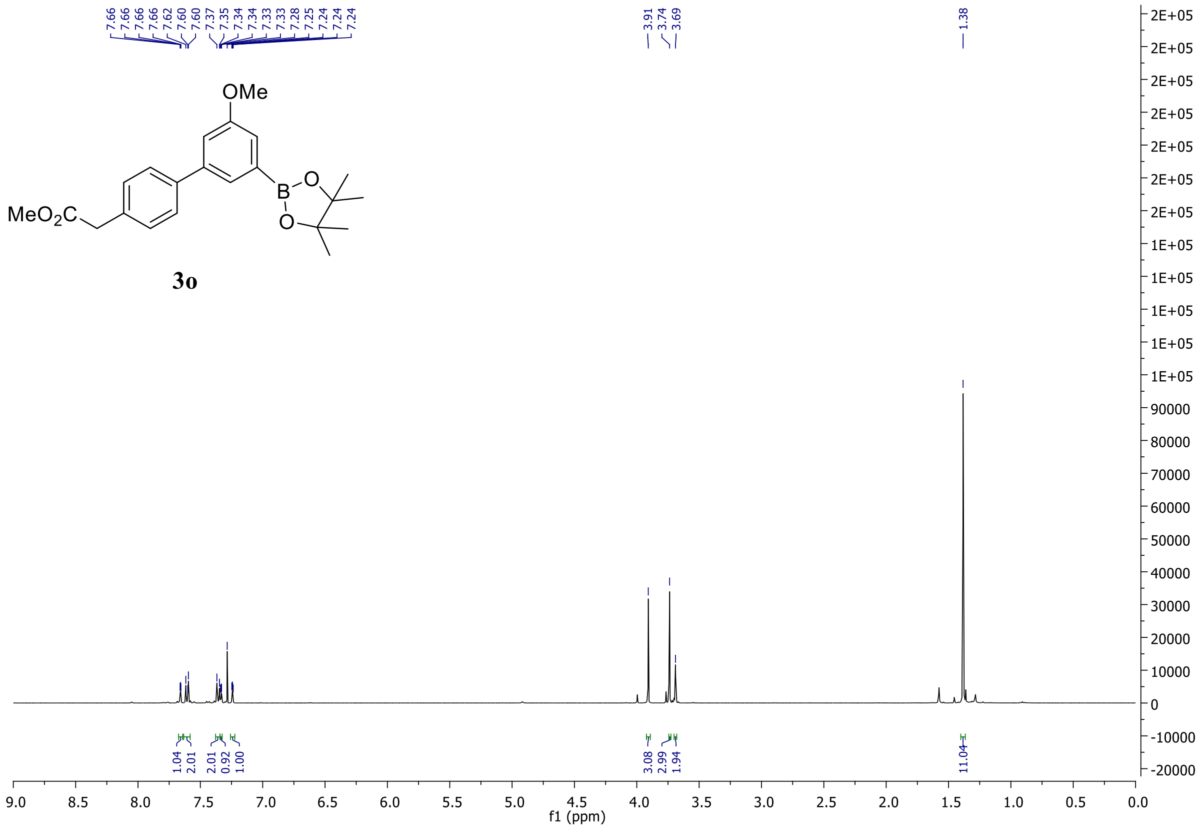

3o

^{13}C NMR of 3o

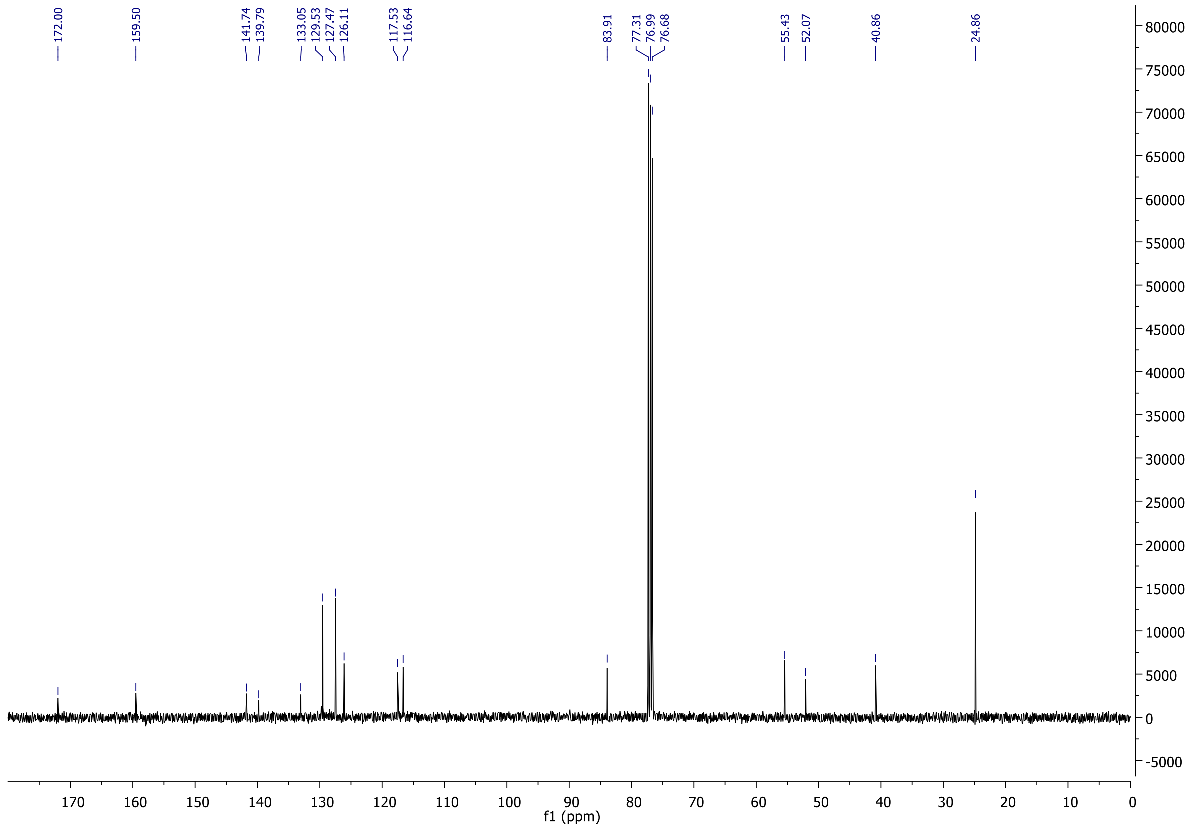

^{11}B NMR of 3o

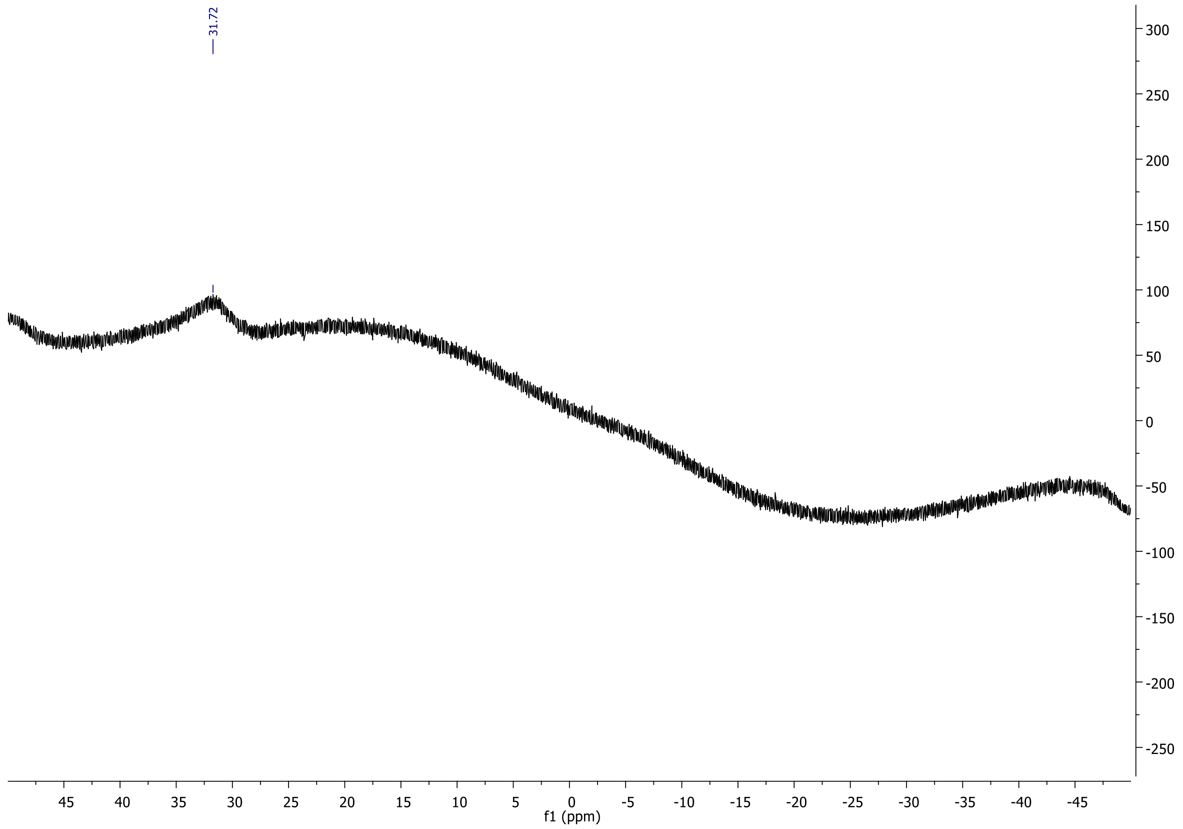

HRMS of 3o

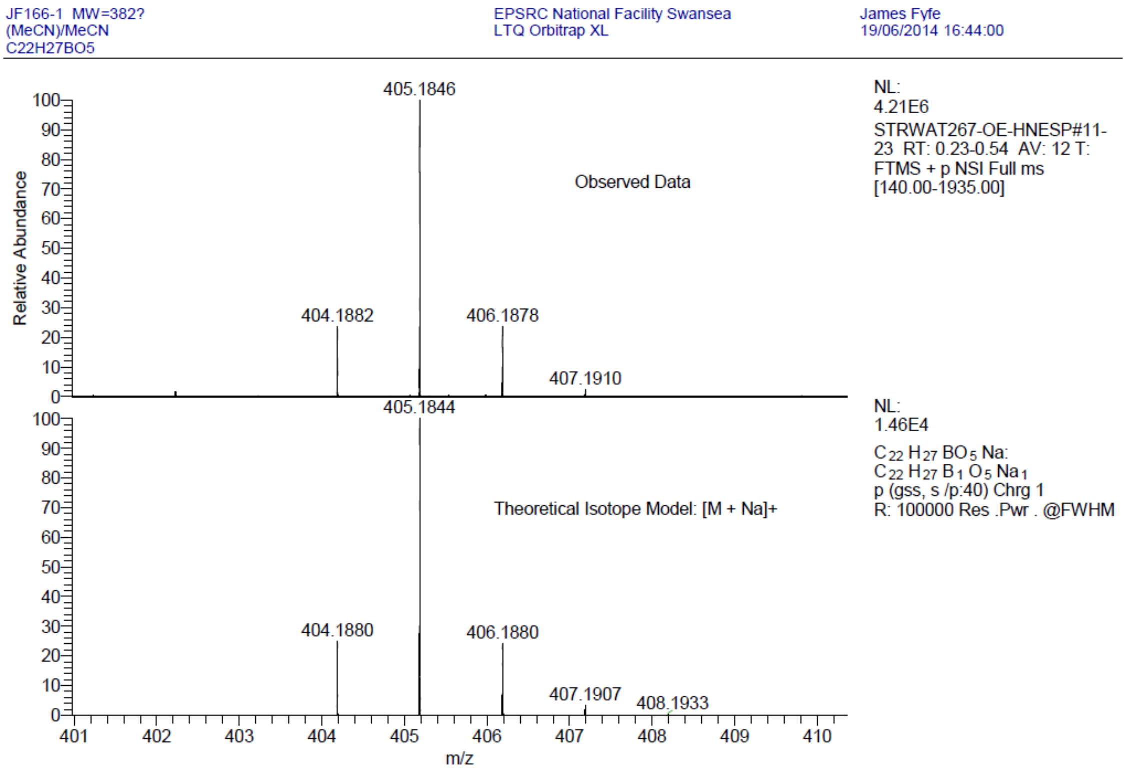

^{1}H NMR of 3p

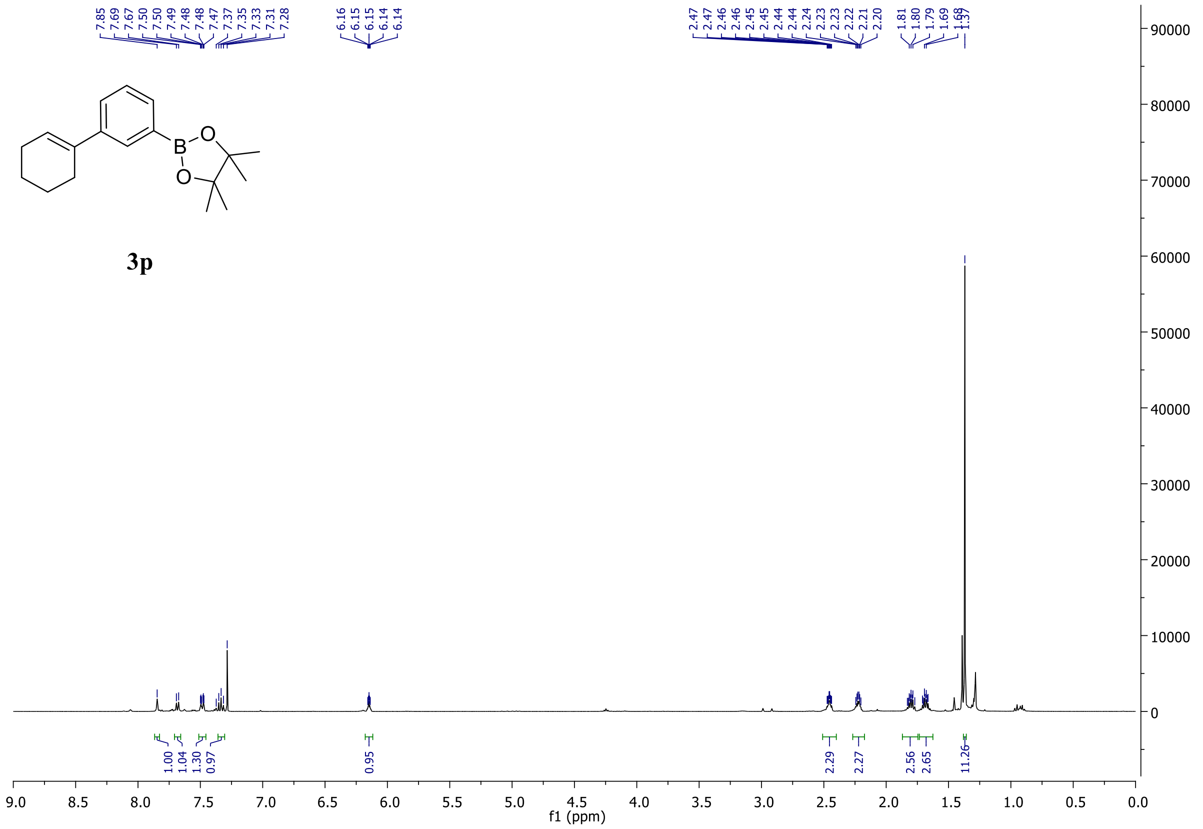

^{13}C NMR of 3p

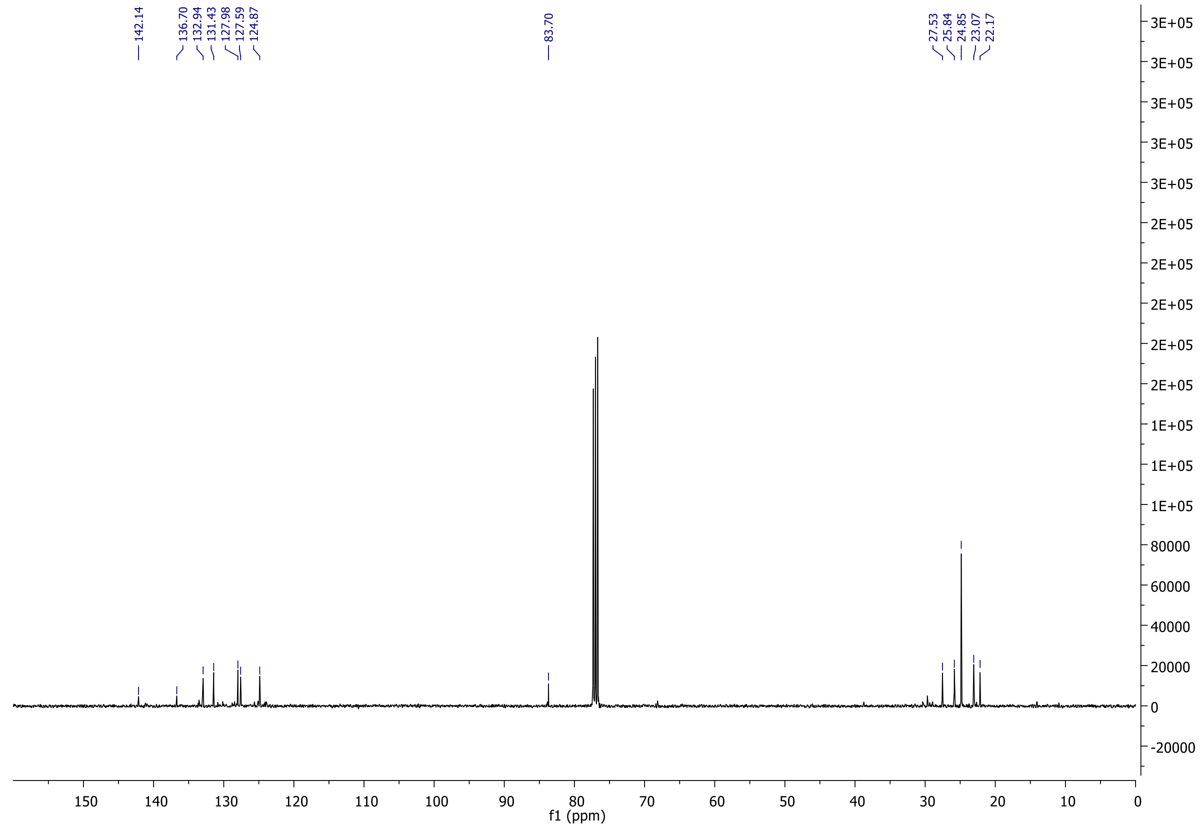

^{11}B NMR of 3p

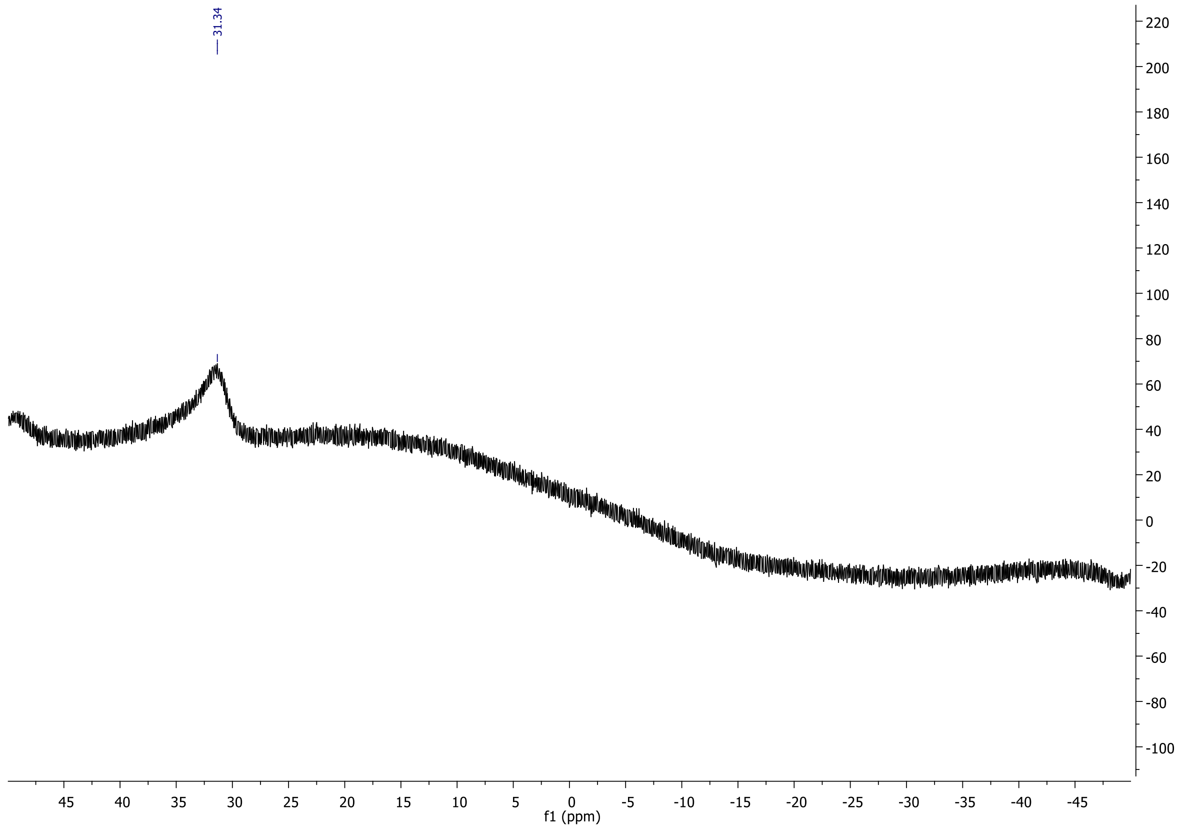

HRMS of 3p

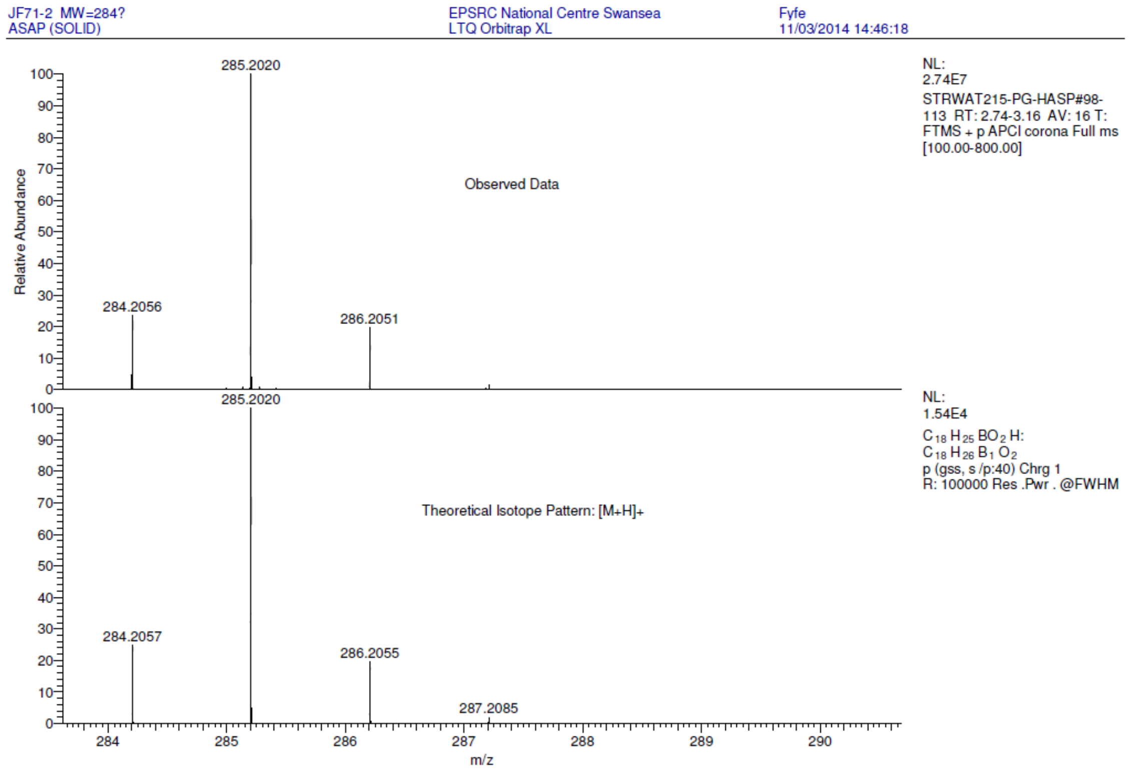

^{1}H NMR of 3q

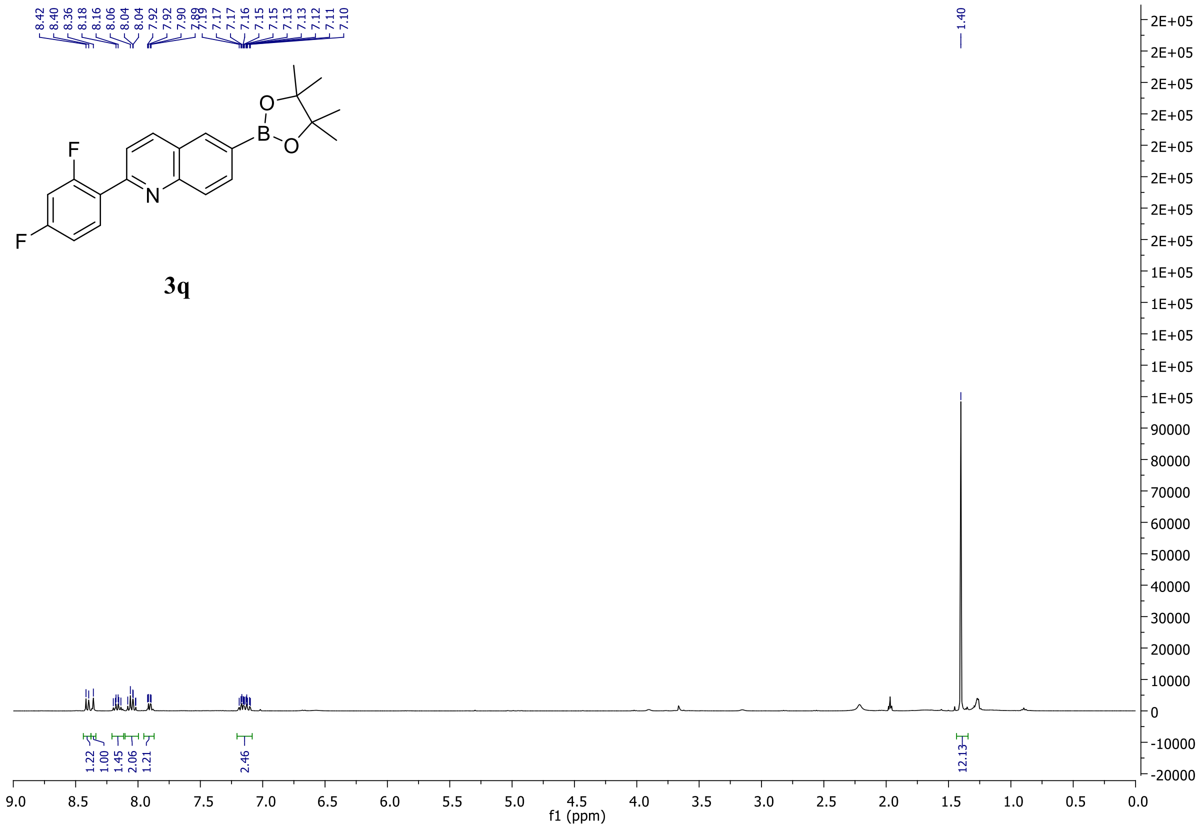

^{13}C NMR of 3q

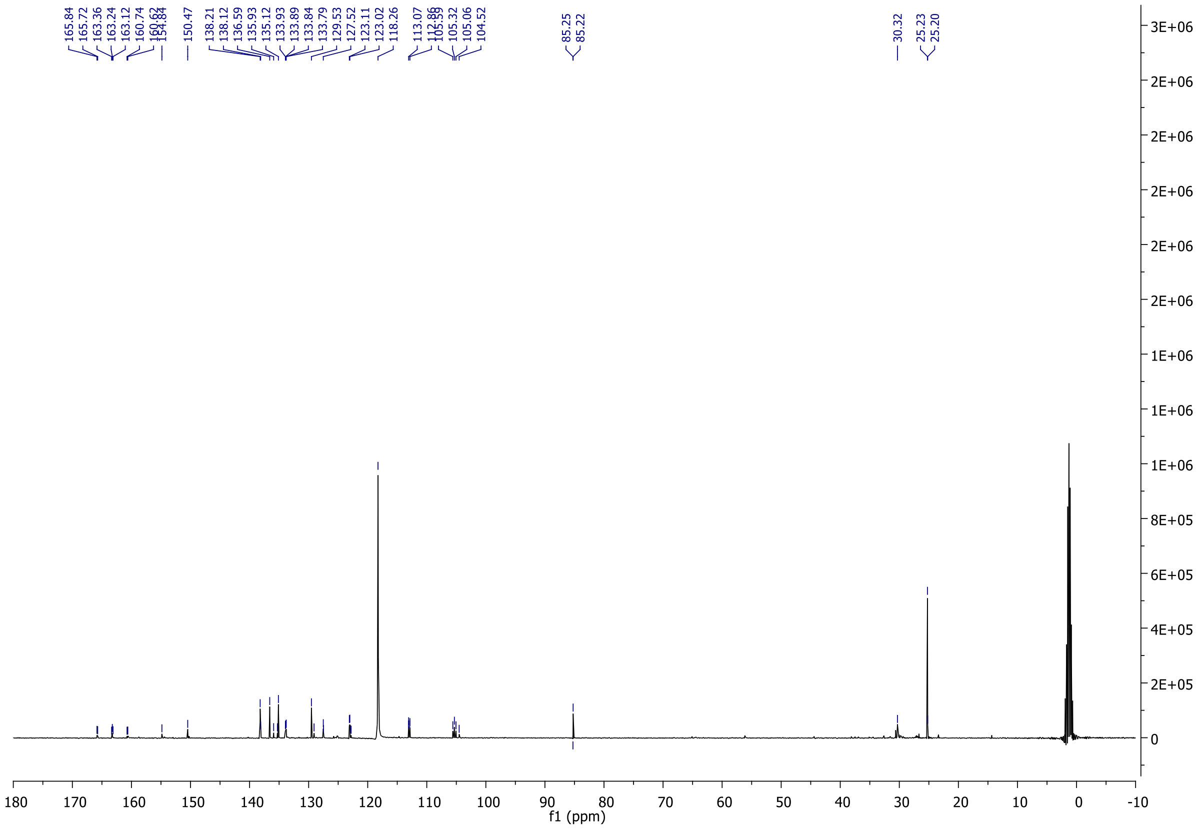

^{11}B NMR of 3q

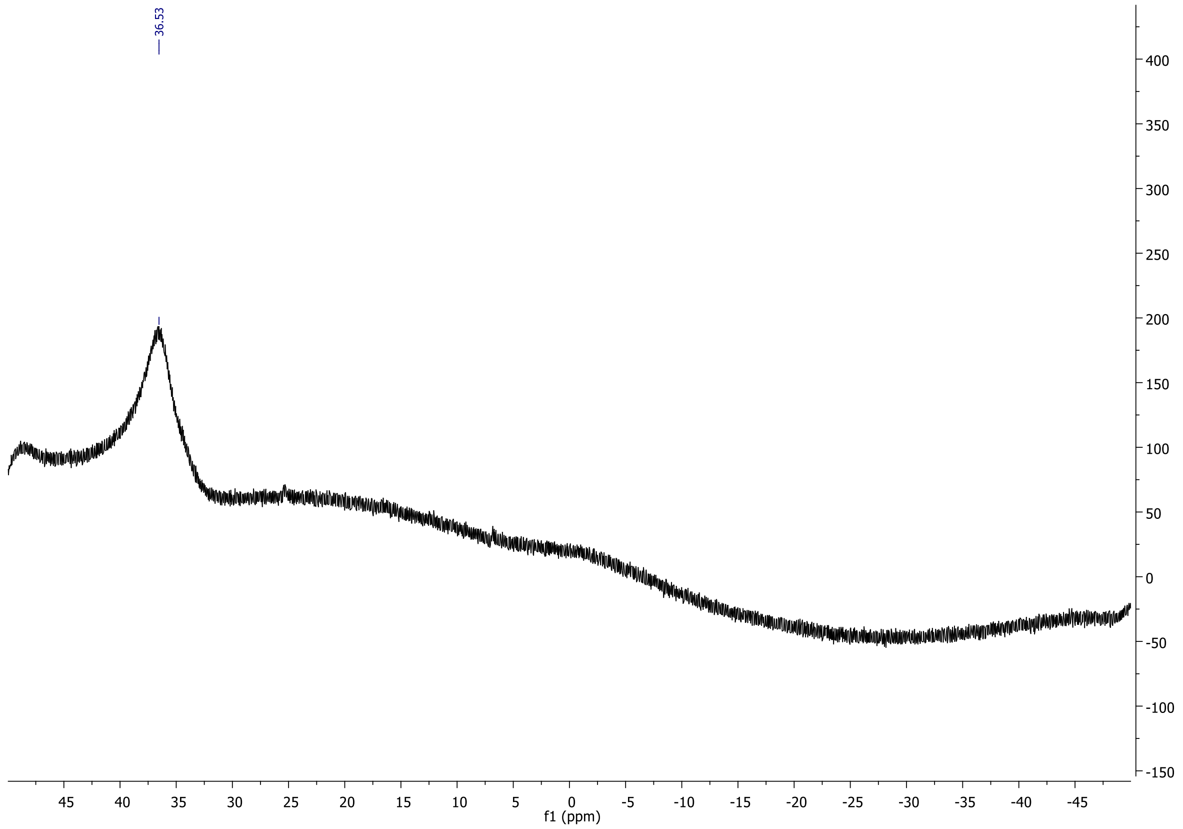

^{19}F NMR of 3q

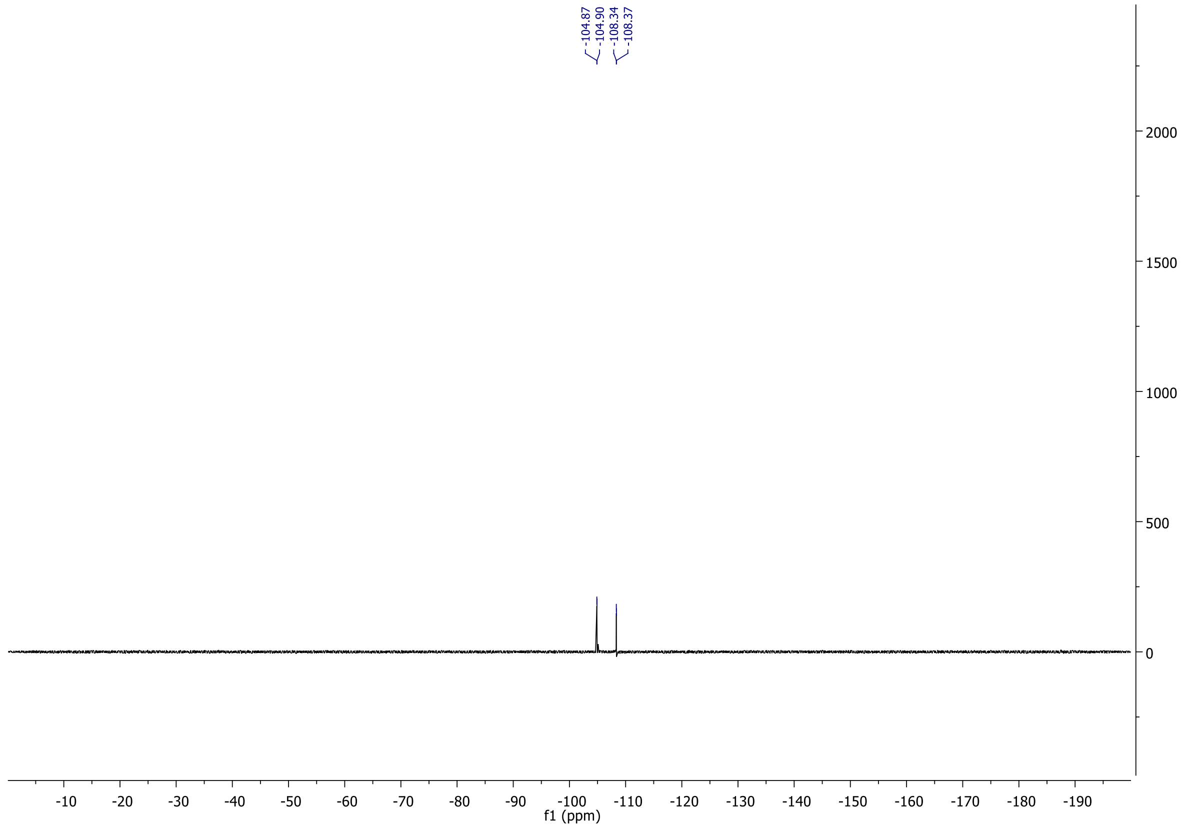

HRMS of 3q

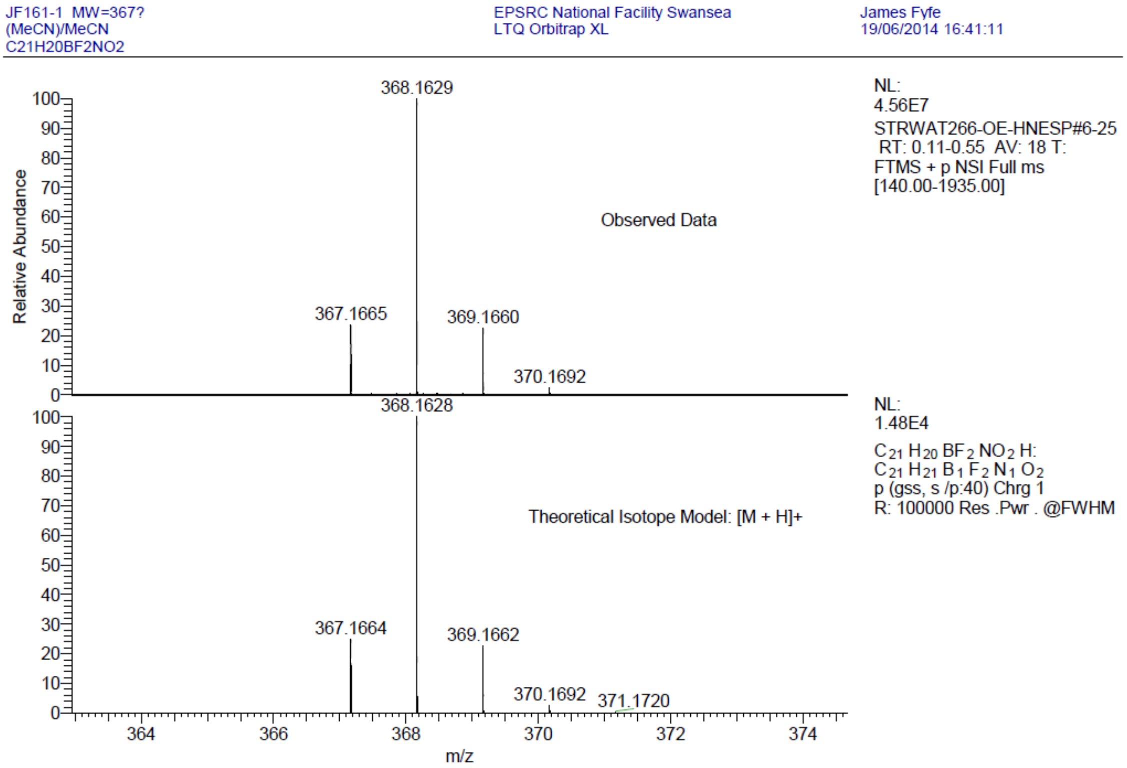

^{1}H NMR of 3r

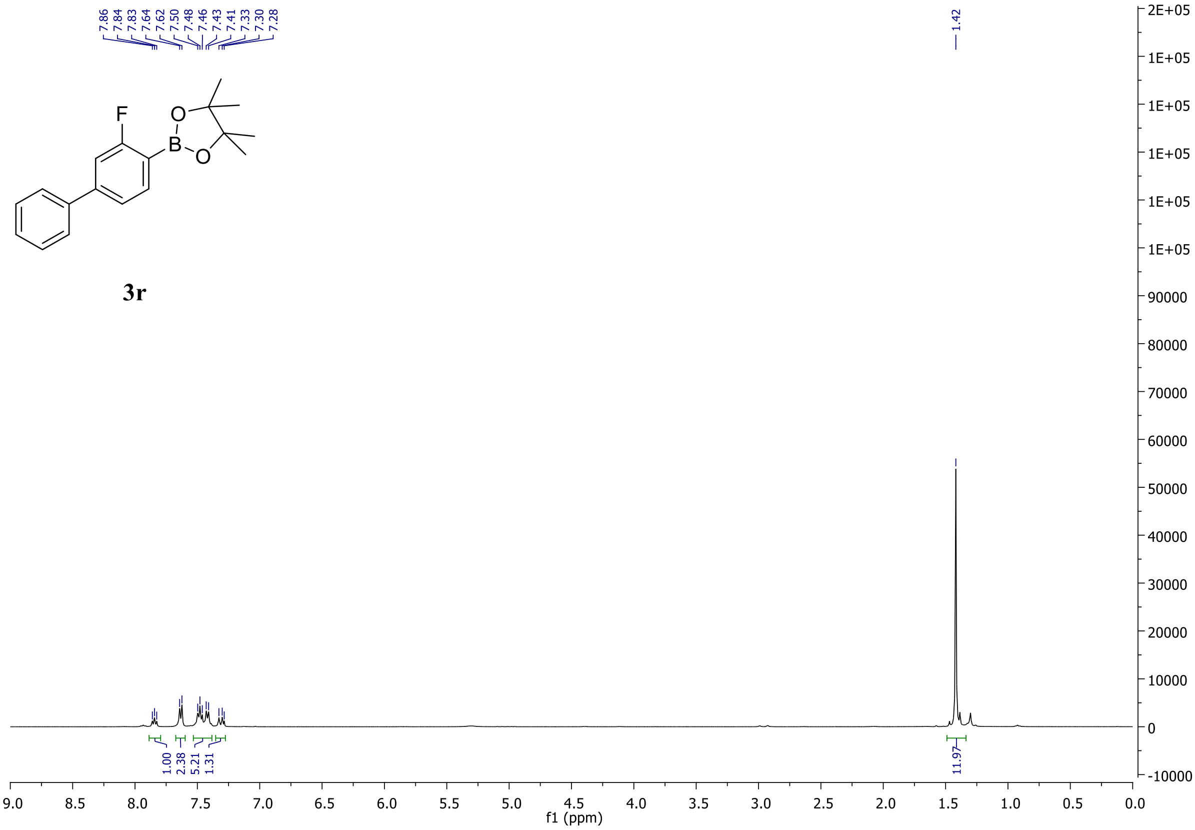

3r

^{13}C NMR of 3r

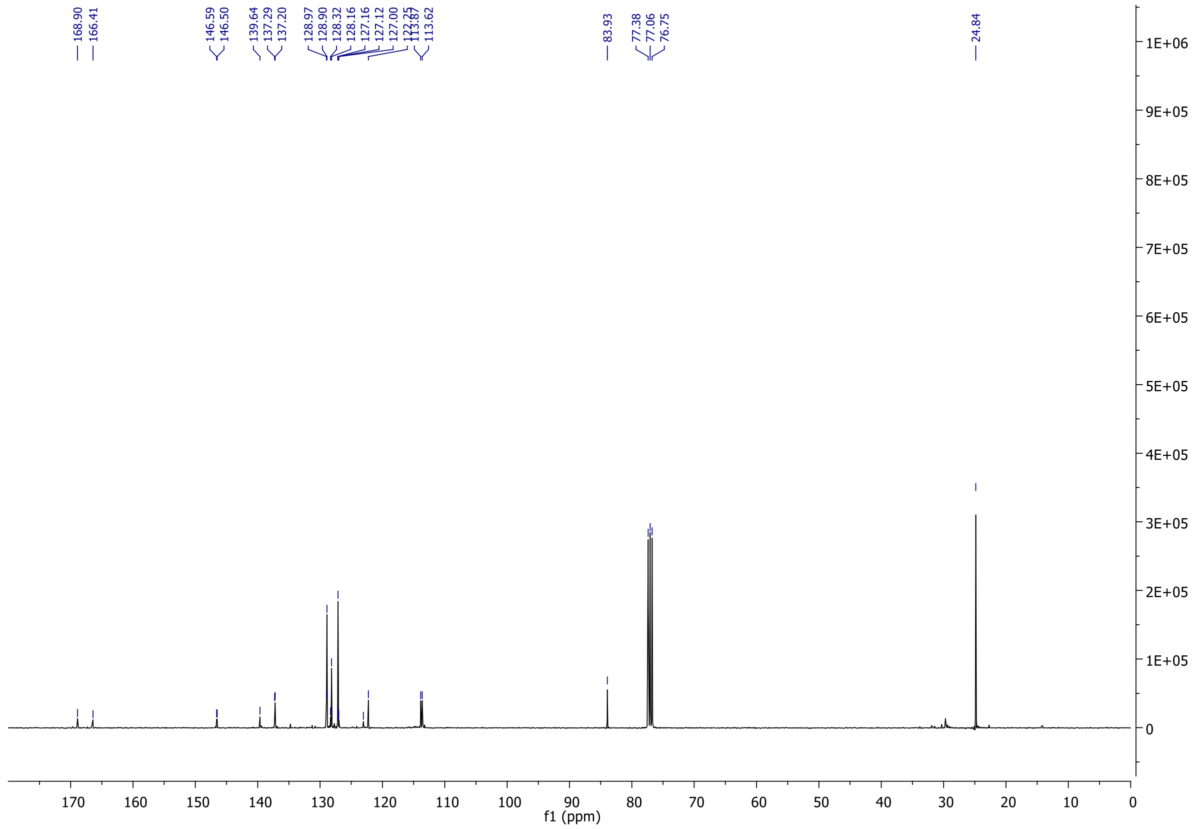

^{11}B NMR of 3r

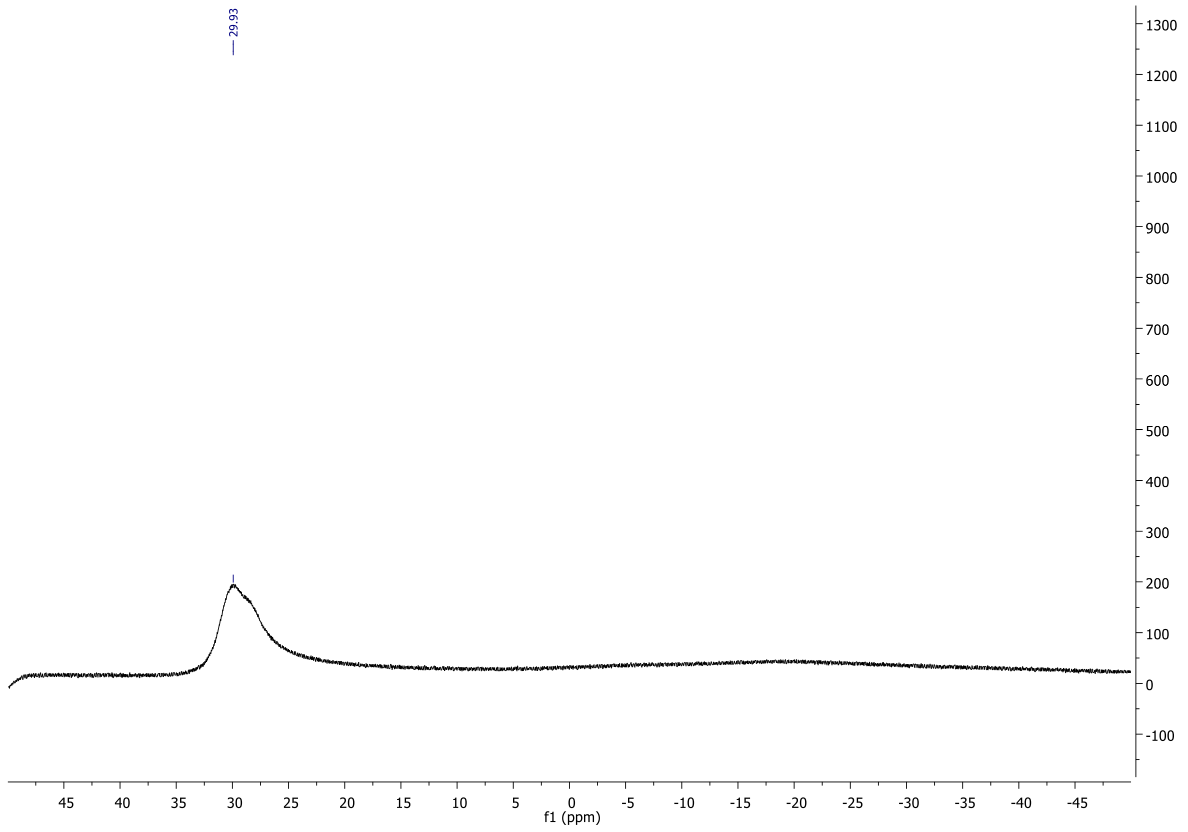

^{19}F NMR of 3r

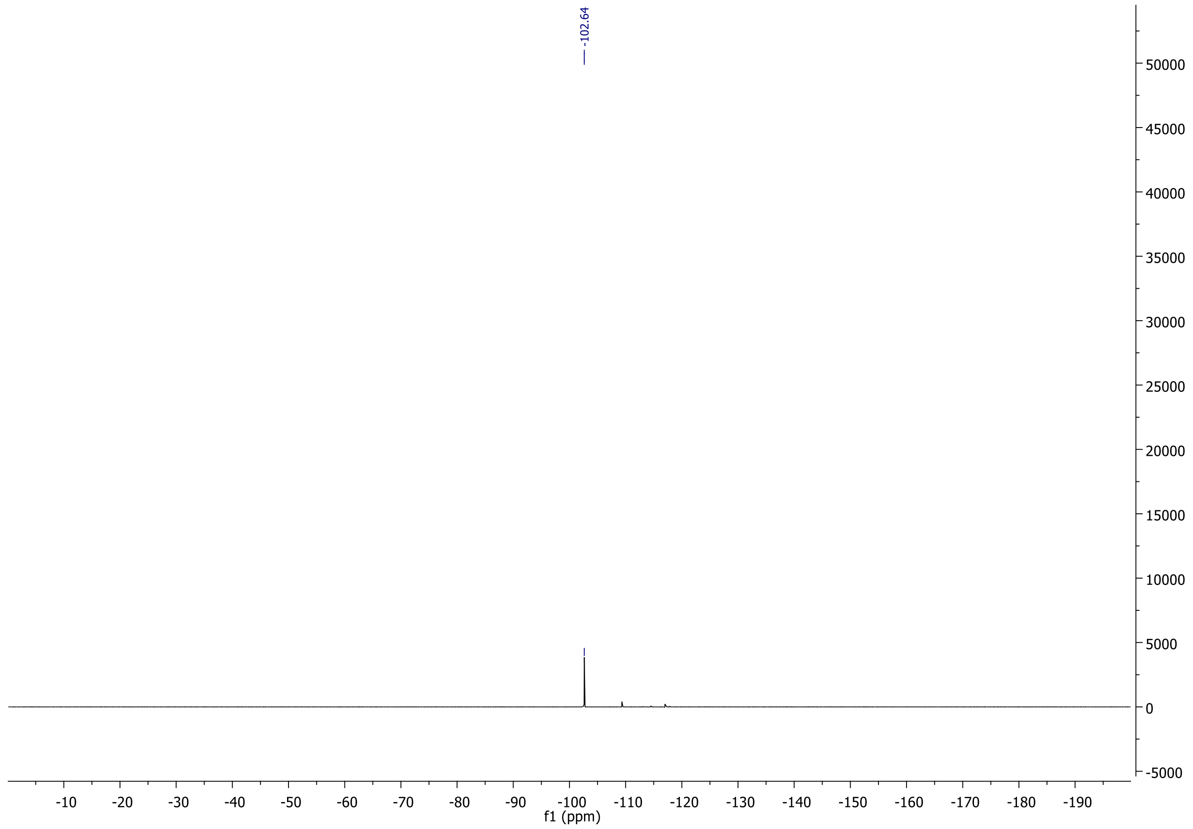

HRMS of 3r

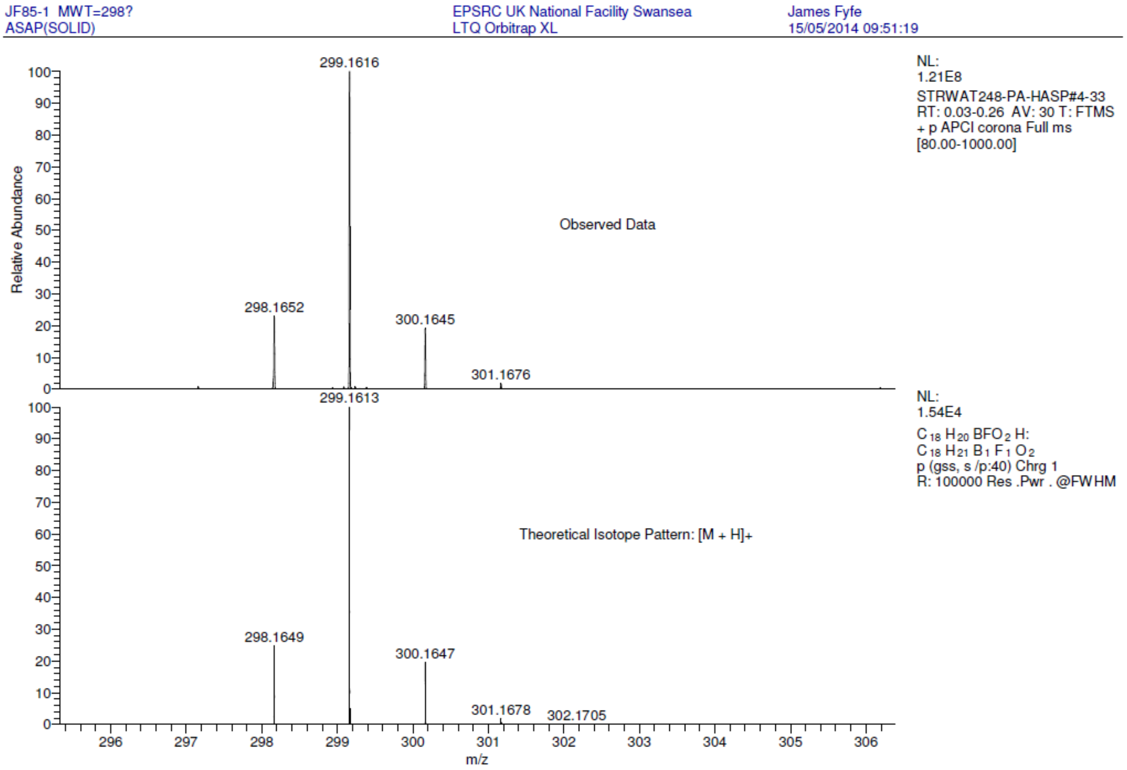

^{1}H NMR of 3s

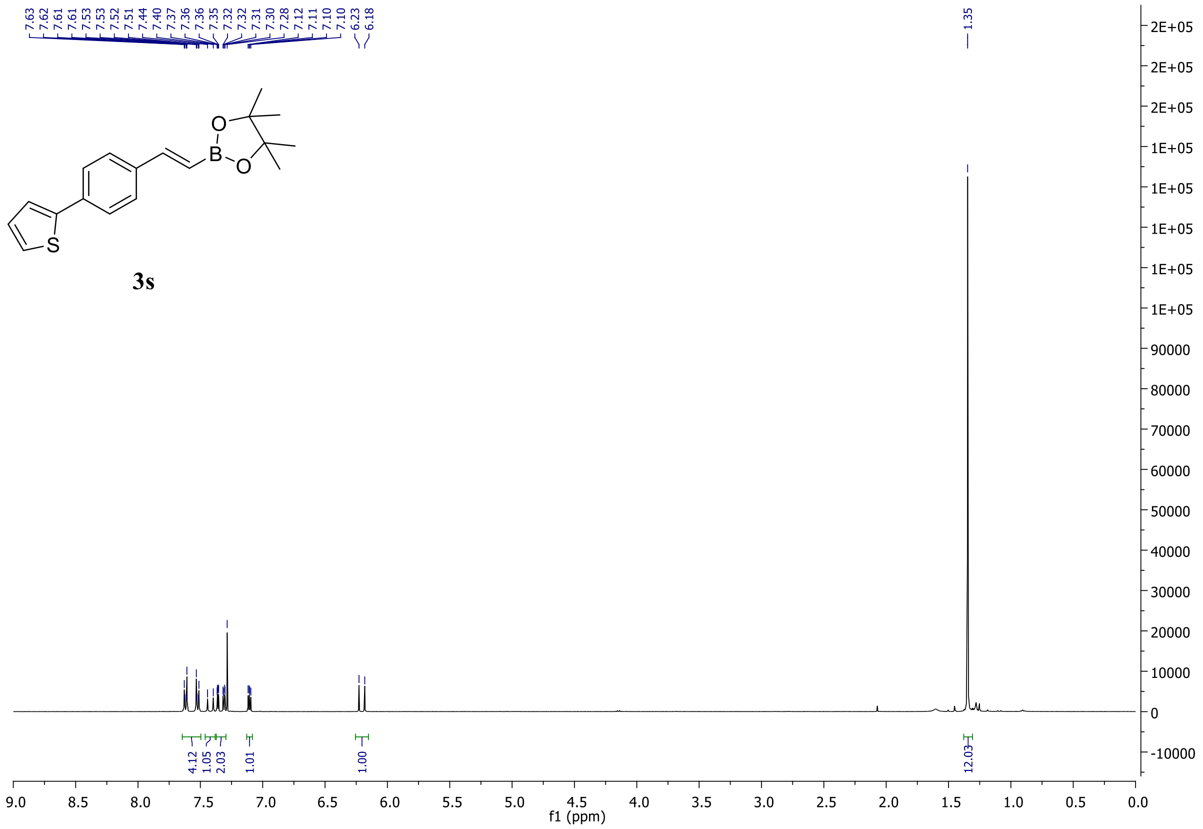

^{13}C NMR of 3s

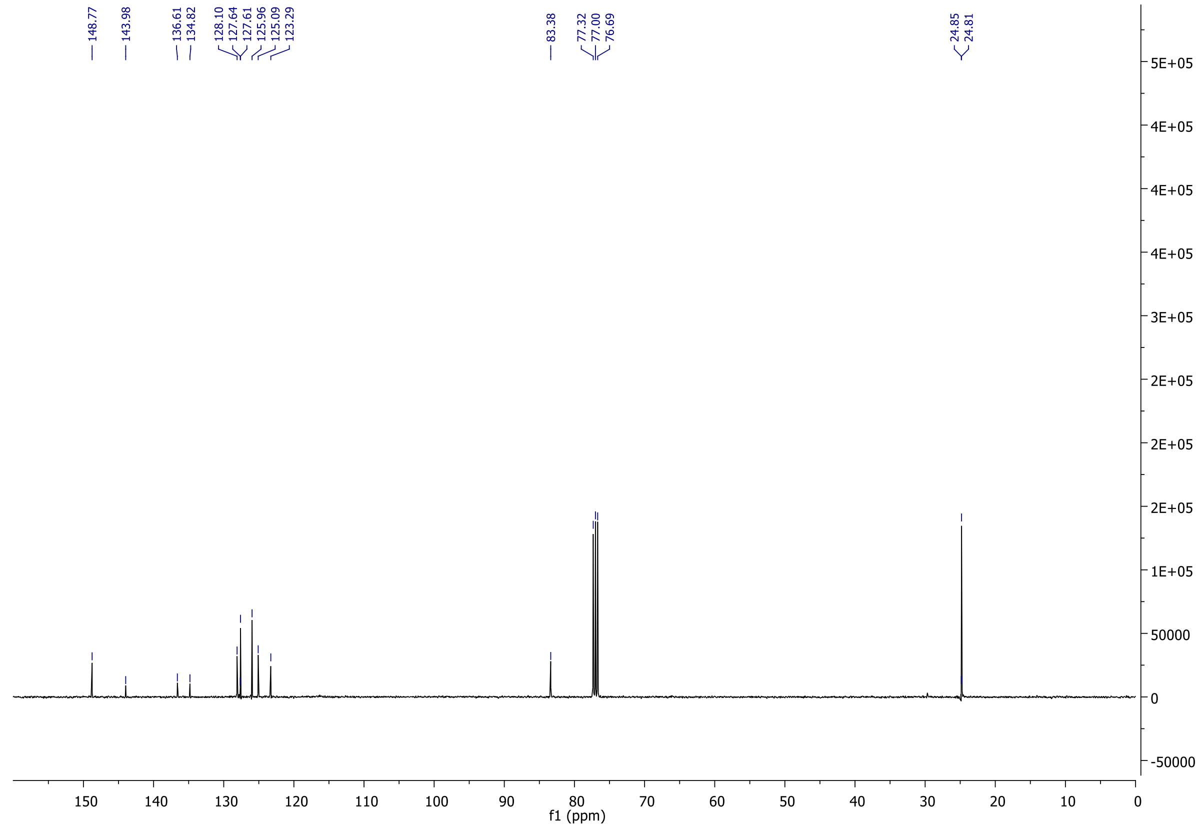

^{11}B NMR of 3s

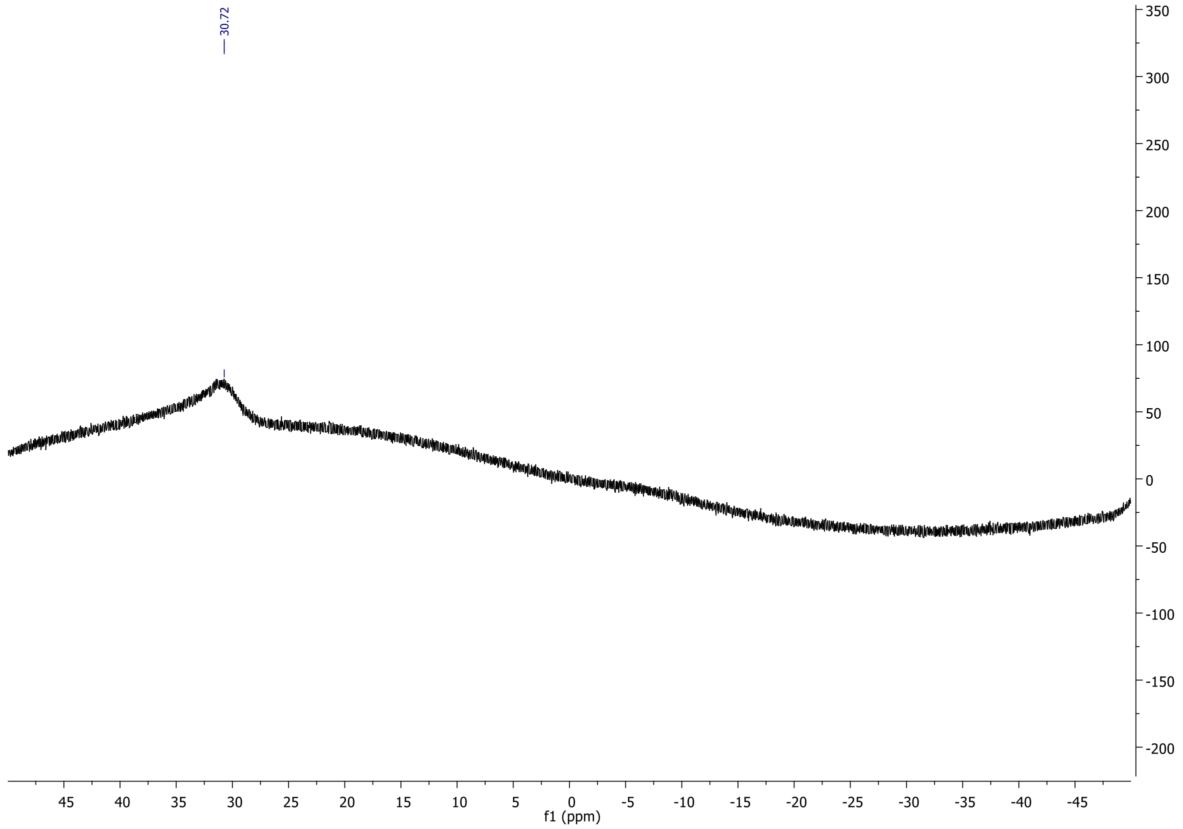

HRMS of 3s

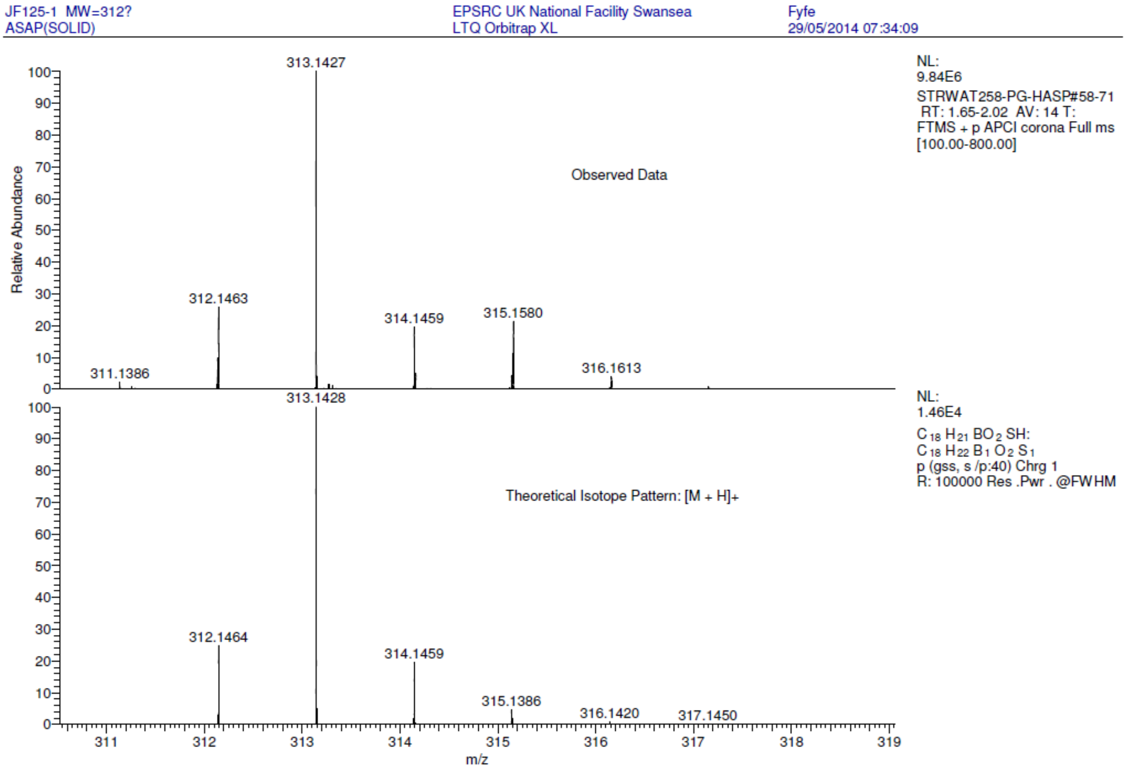

^{1}H NMR of 3t

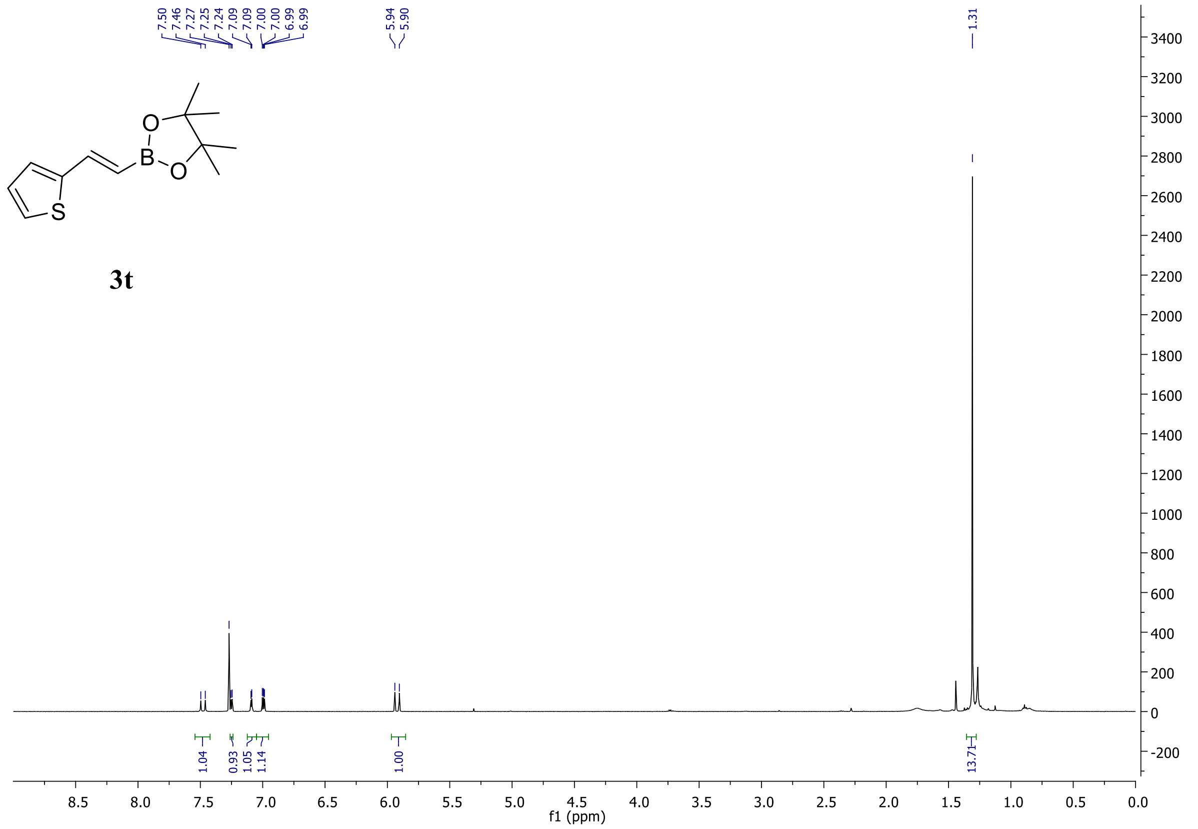

3t

^{13}C NMR of 3t

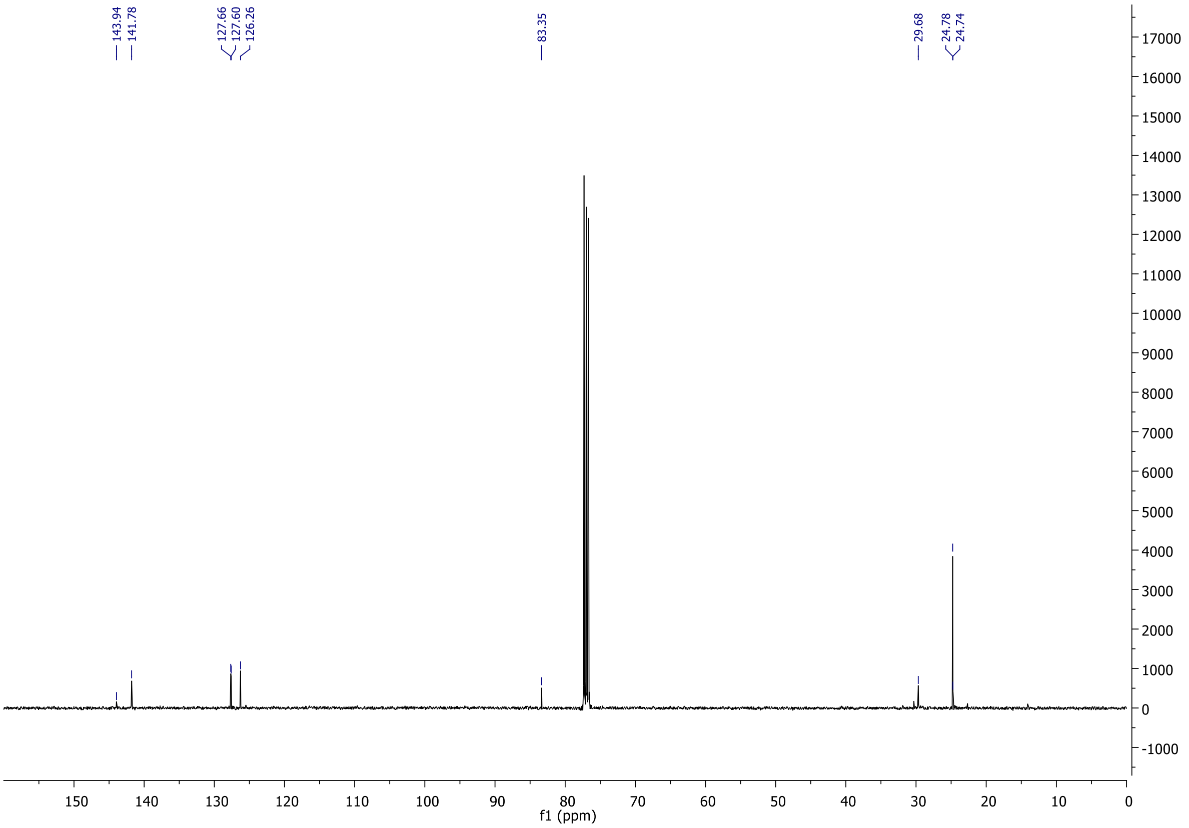

^{11}B NMR of 3t

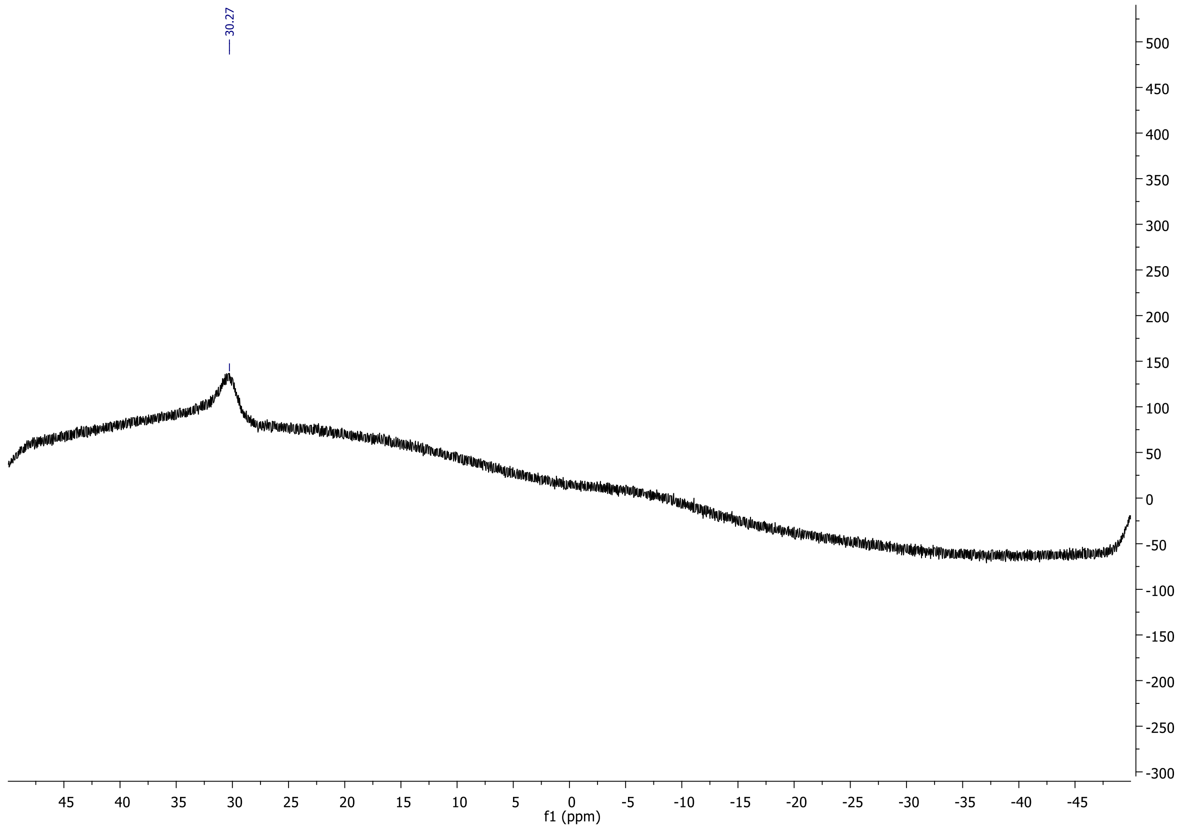

HRMS of 3t

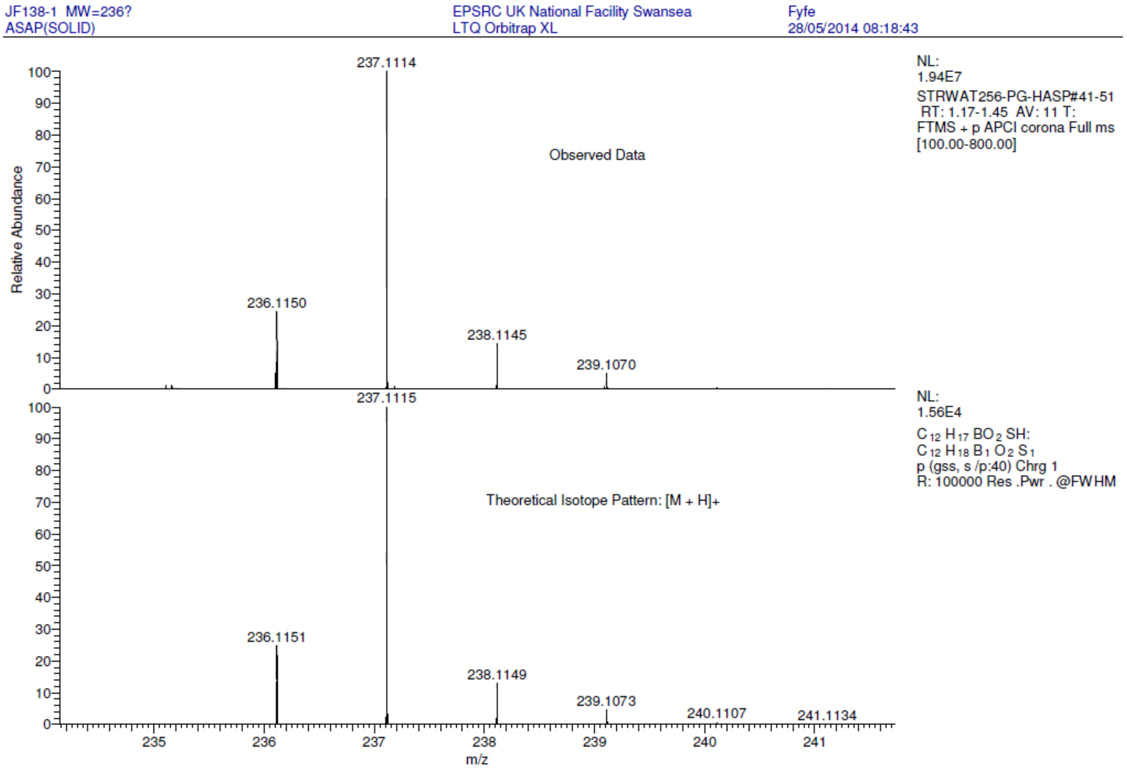

^{1}H NMR of 3u

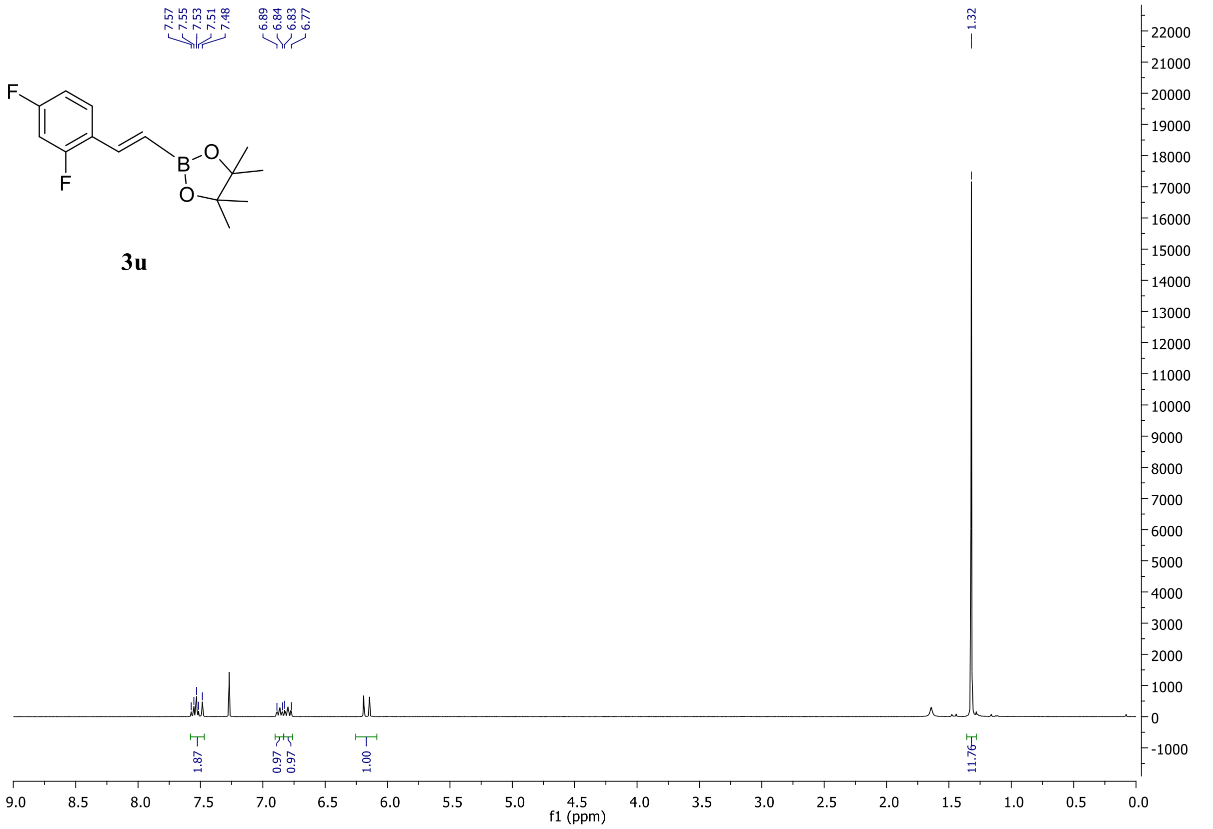

^{13}C NMR of 3u

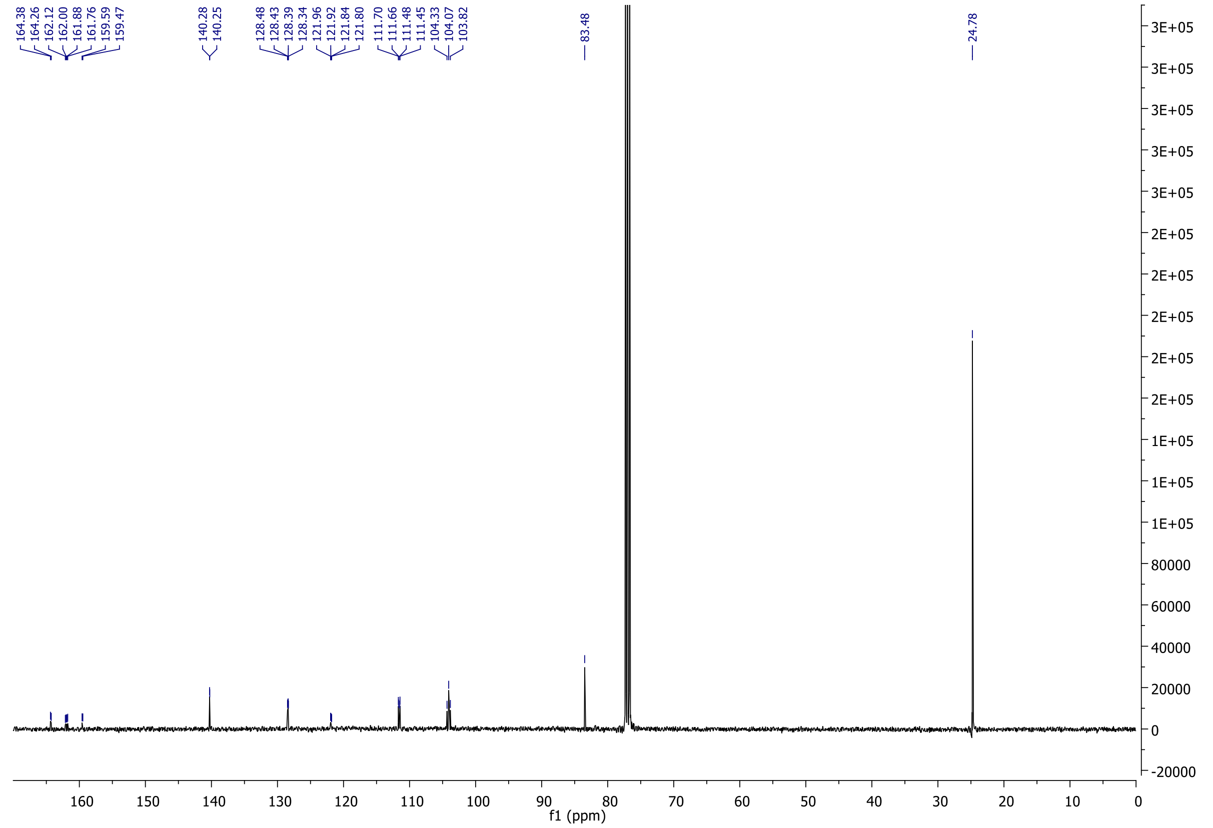

^{11}B NMR of 3u

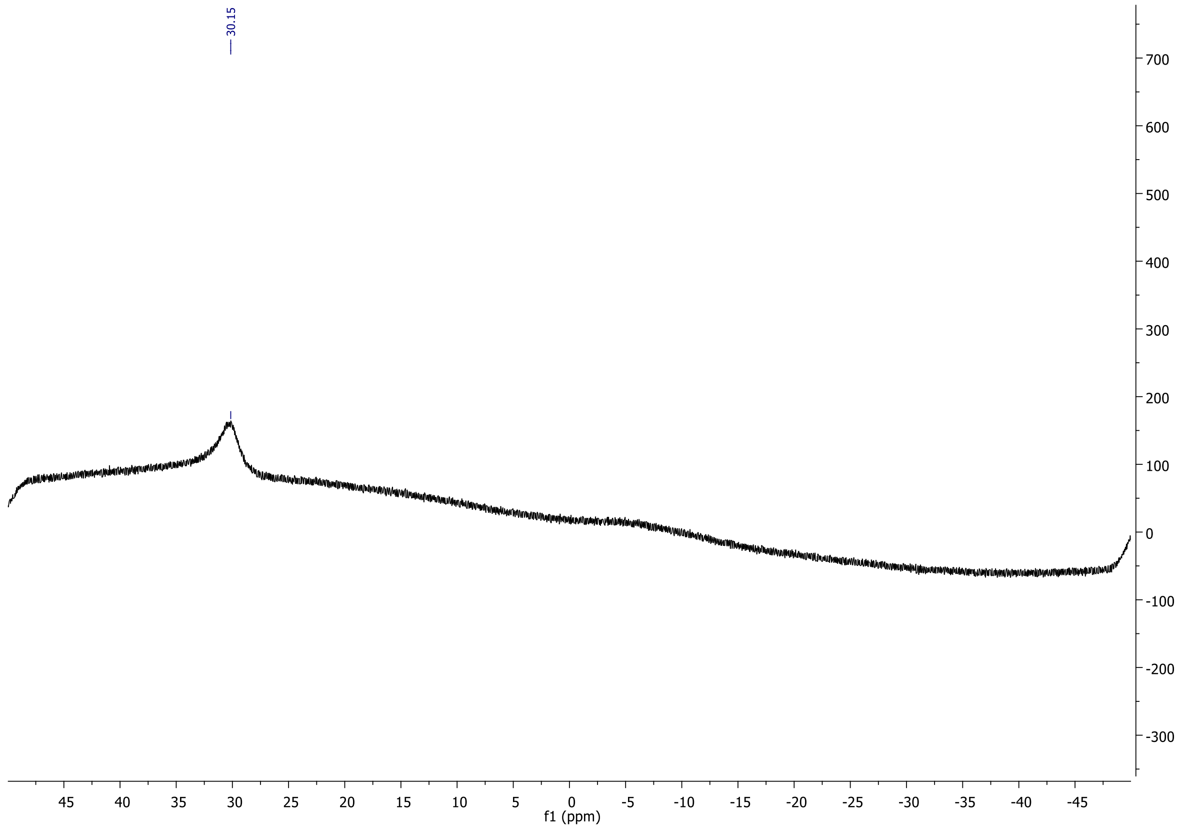

^{19}F NMR of 3u

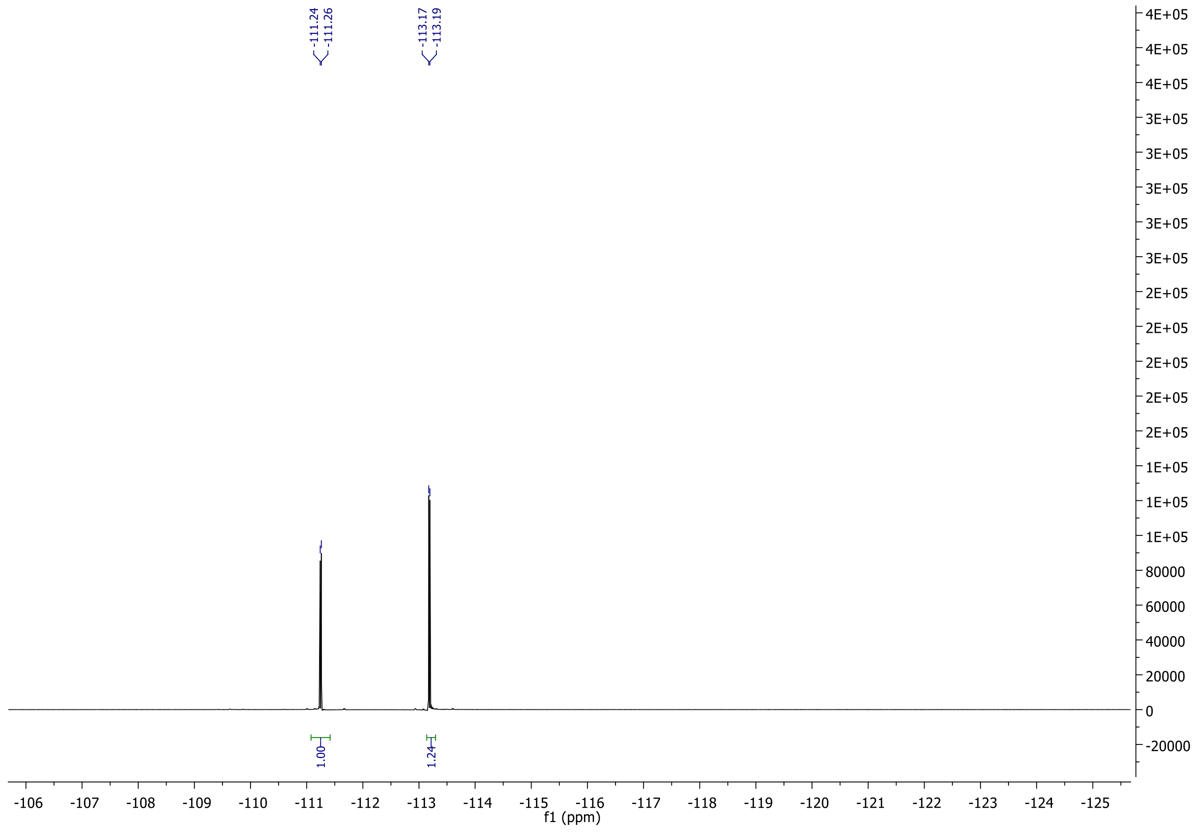

HRMS of 3u

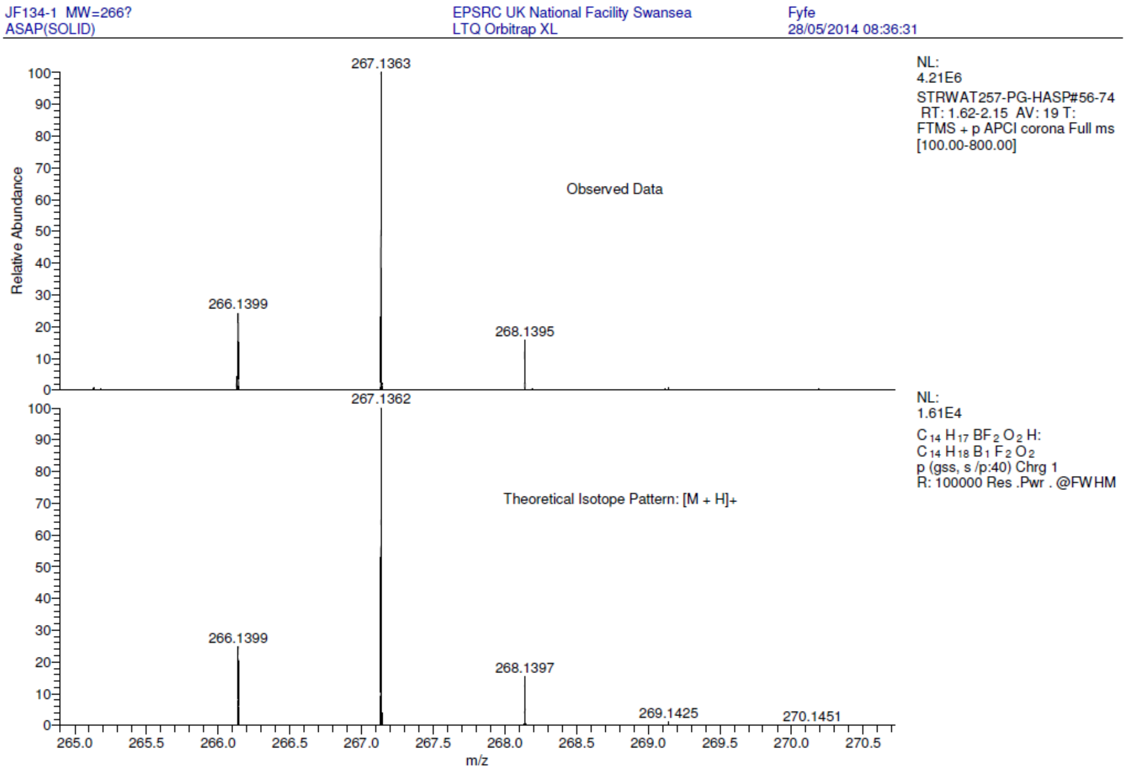

^{1}H NMR of 3v

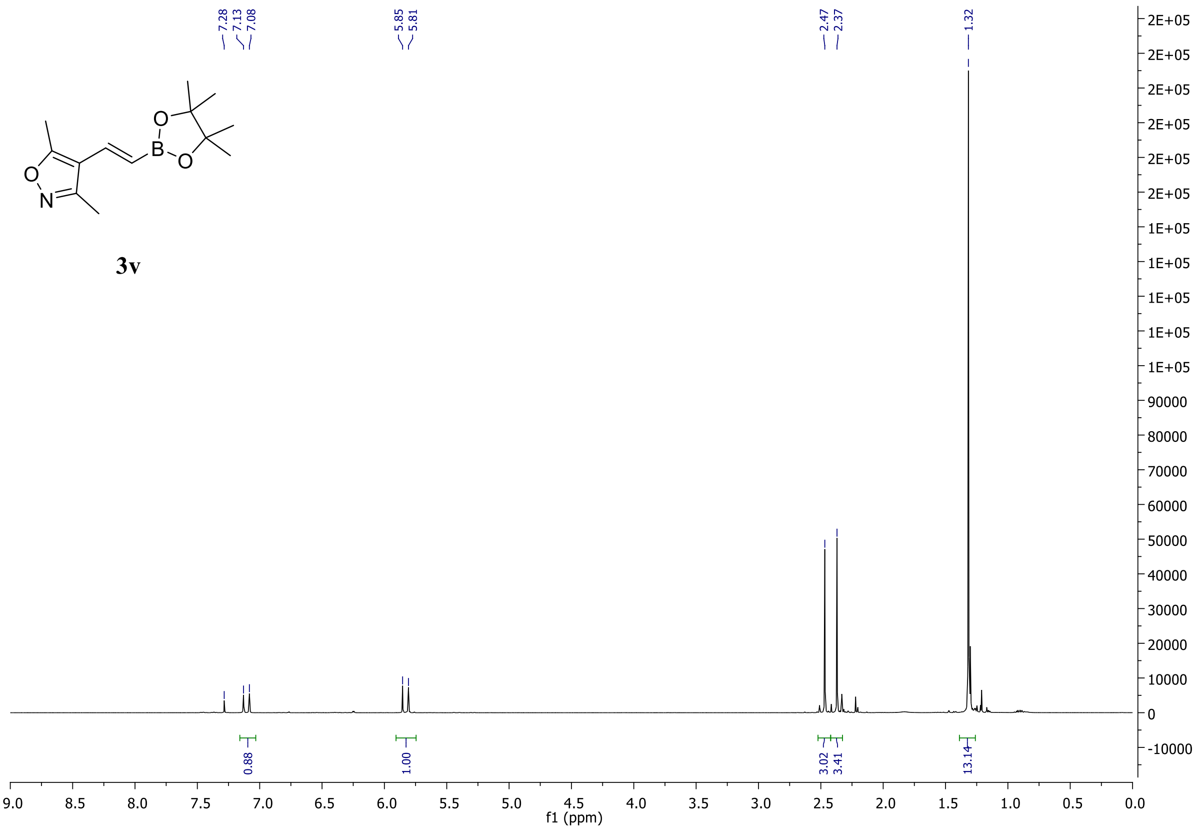

^{13}C NMR of 3v

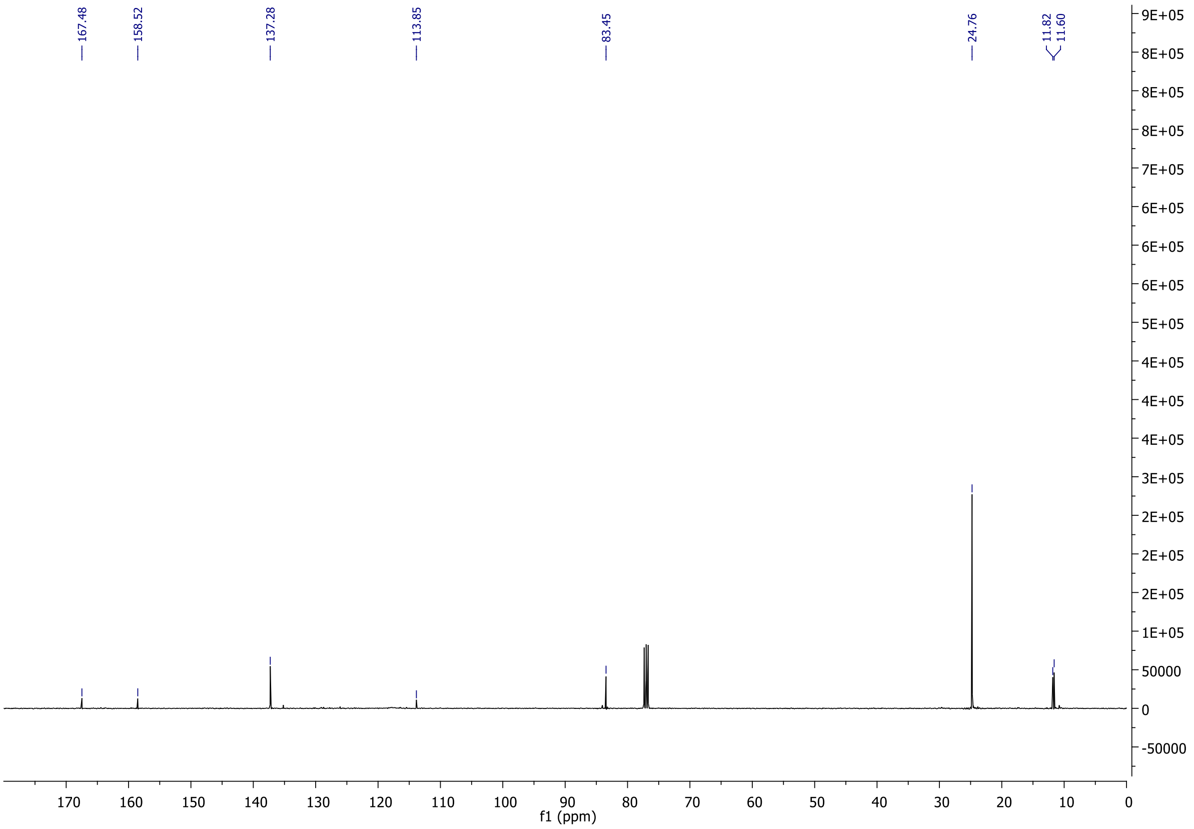

^{11}B NMR of 3v

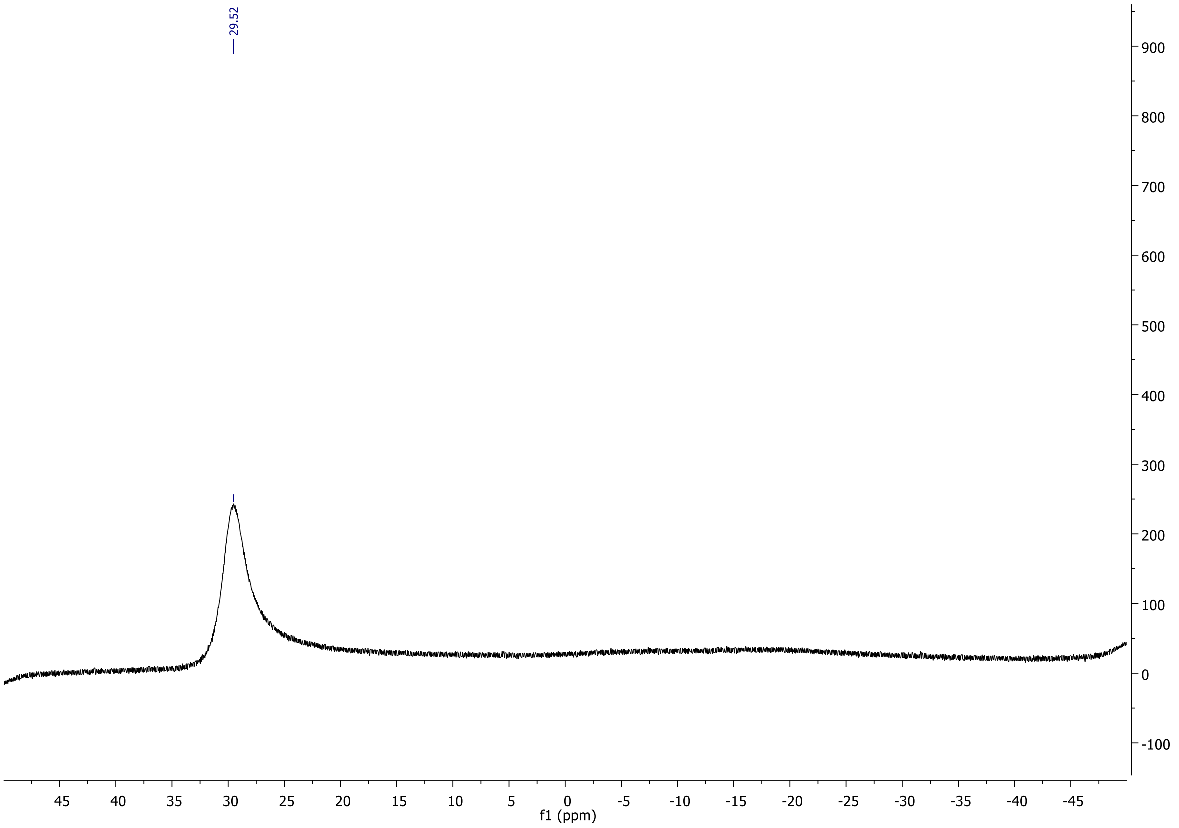

HRMS of 3v

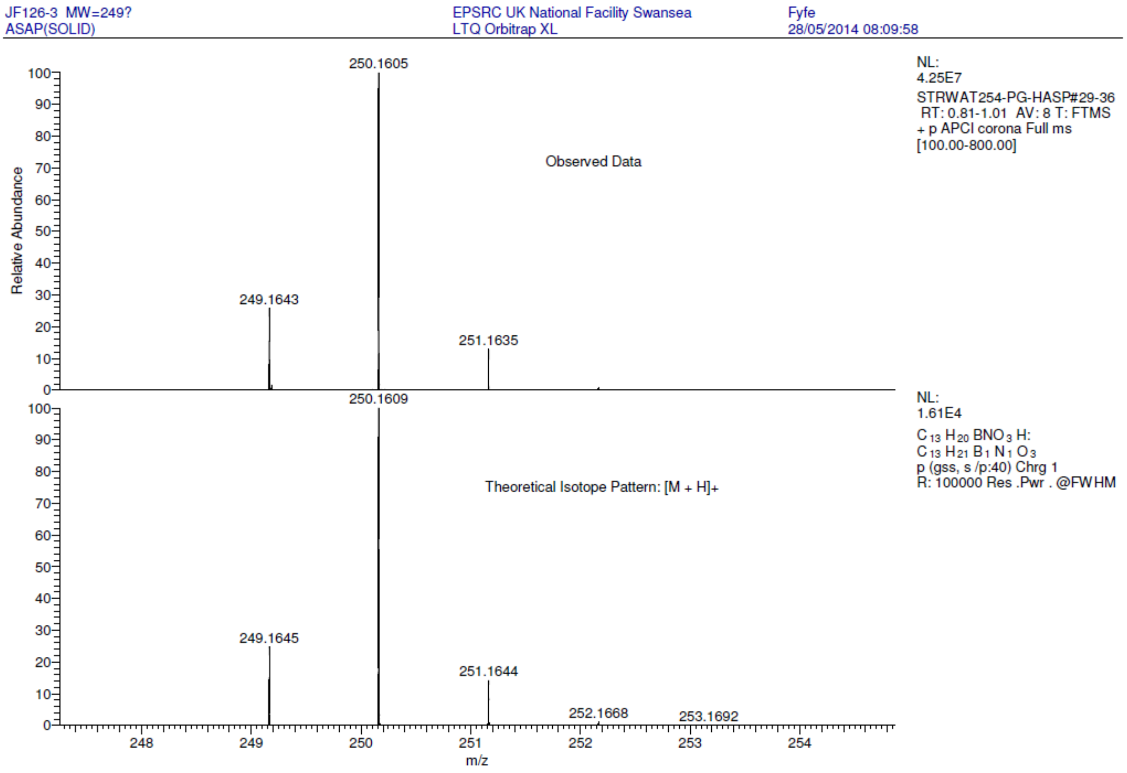

^{1}H NMR of 3w

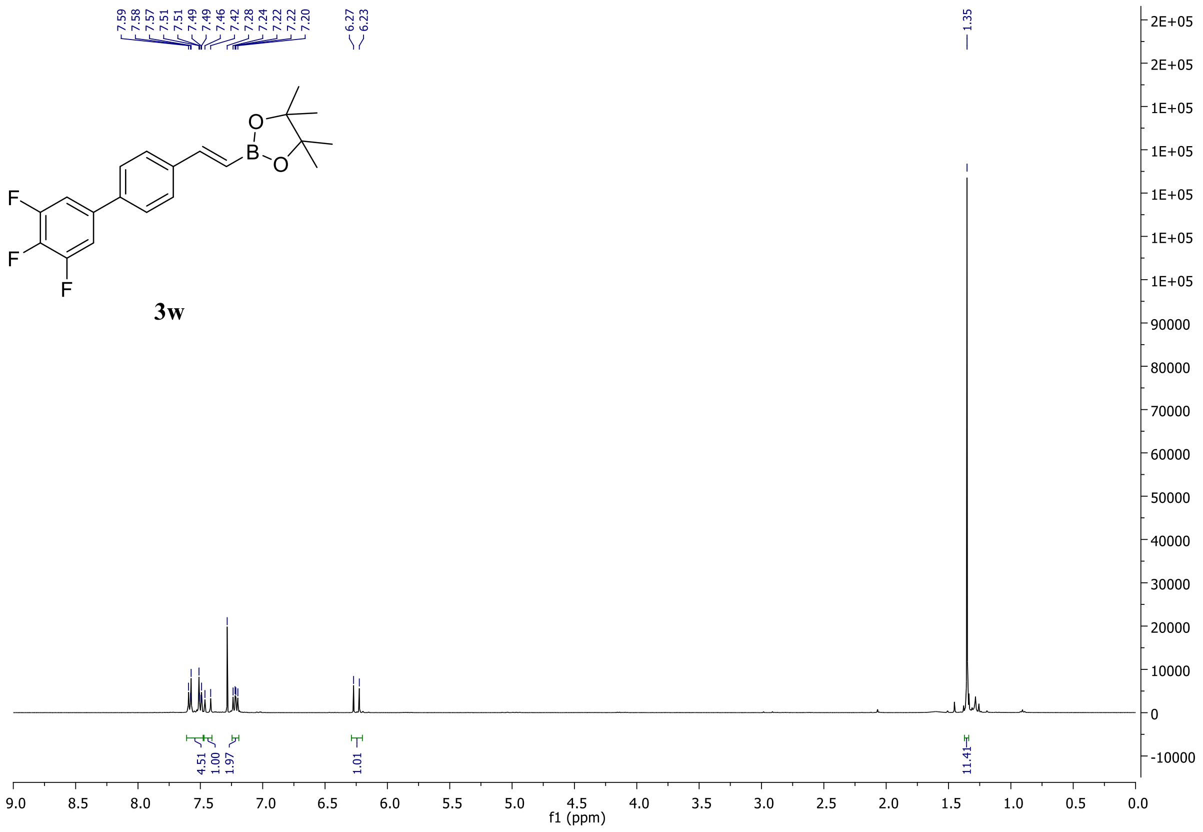

^{13}C NMR of 3w

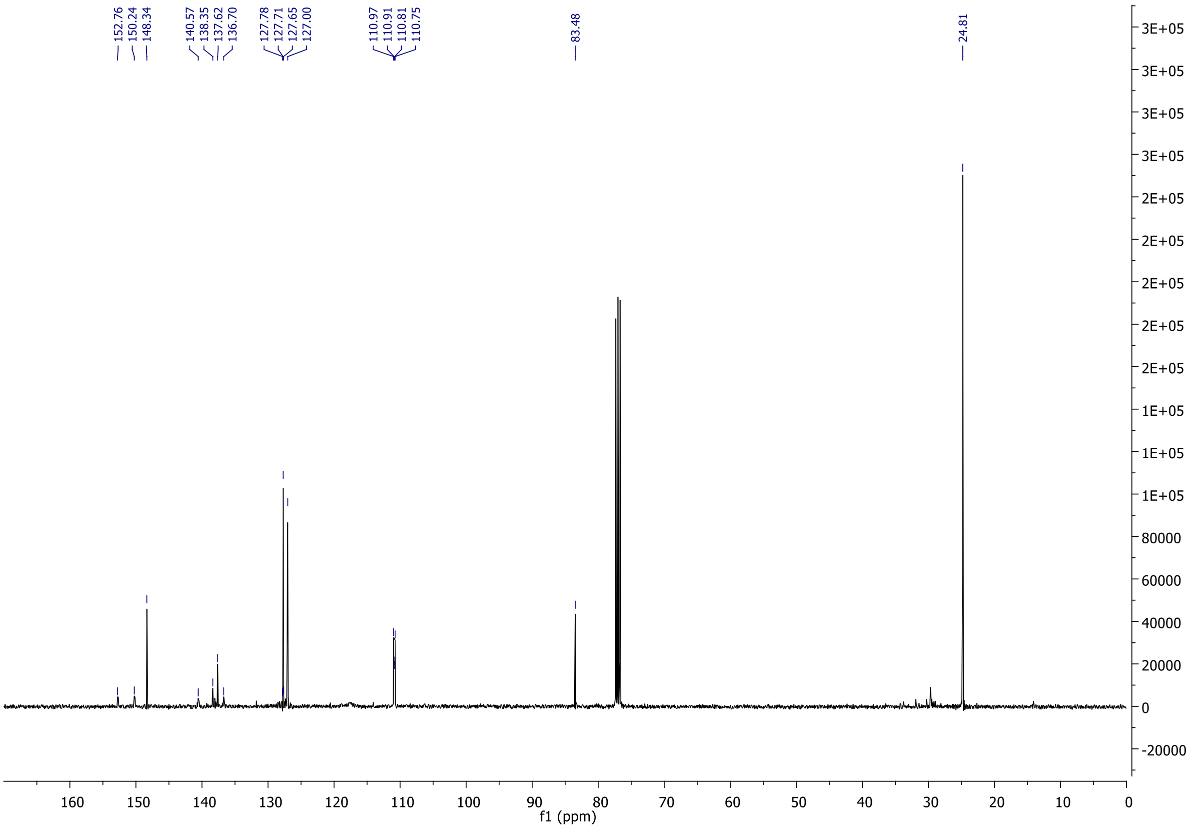

^{11}B NMR of 3w

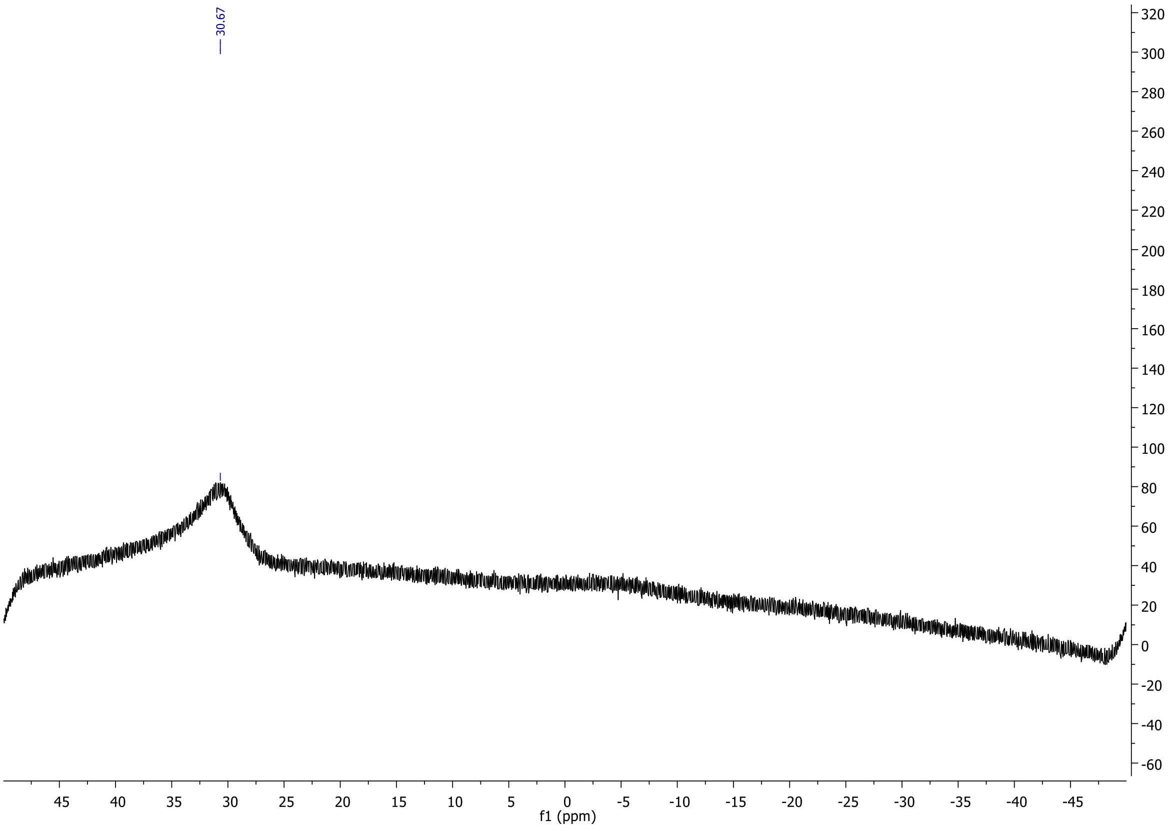

^{19}F NMR of 3w

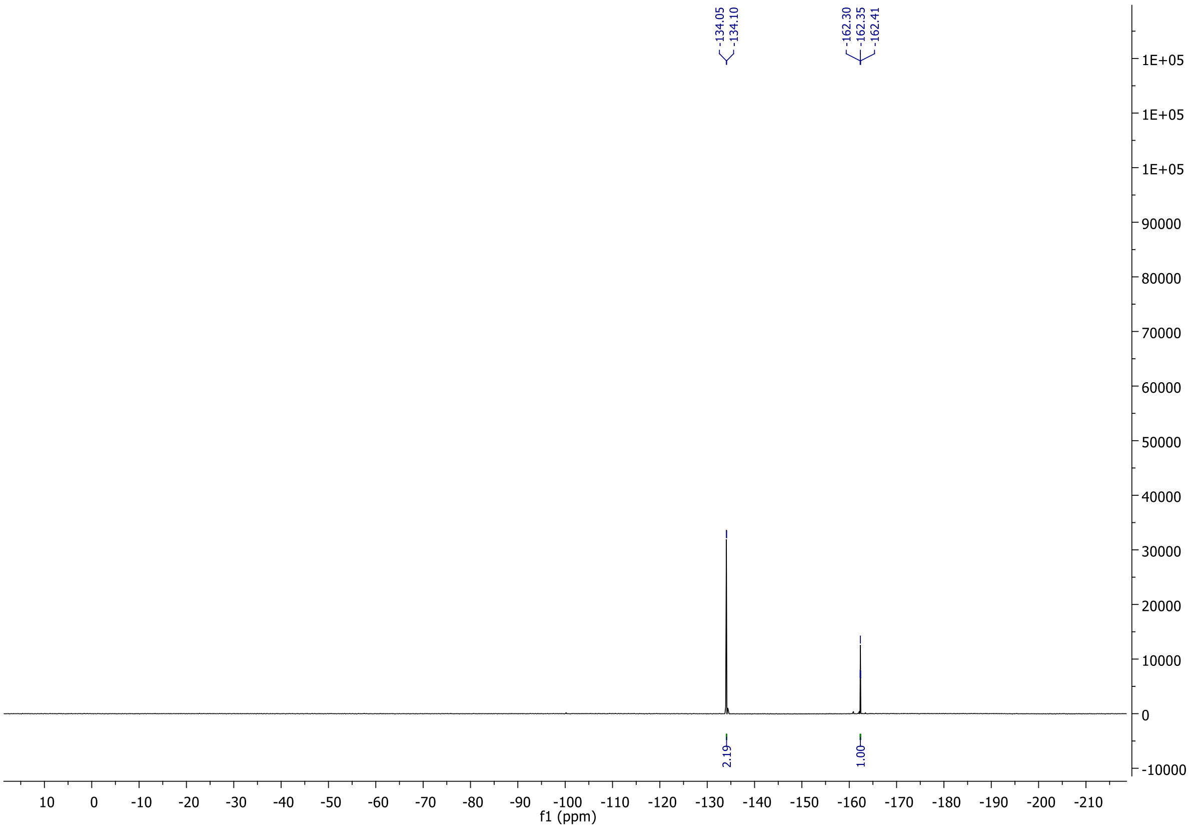

HRMS of 3w

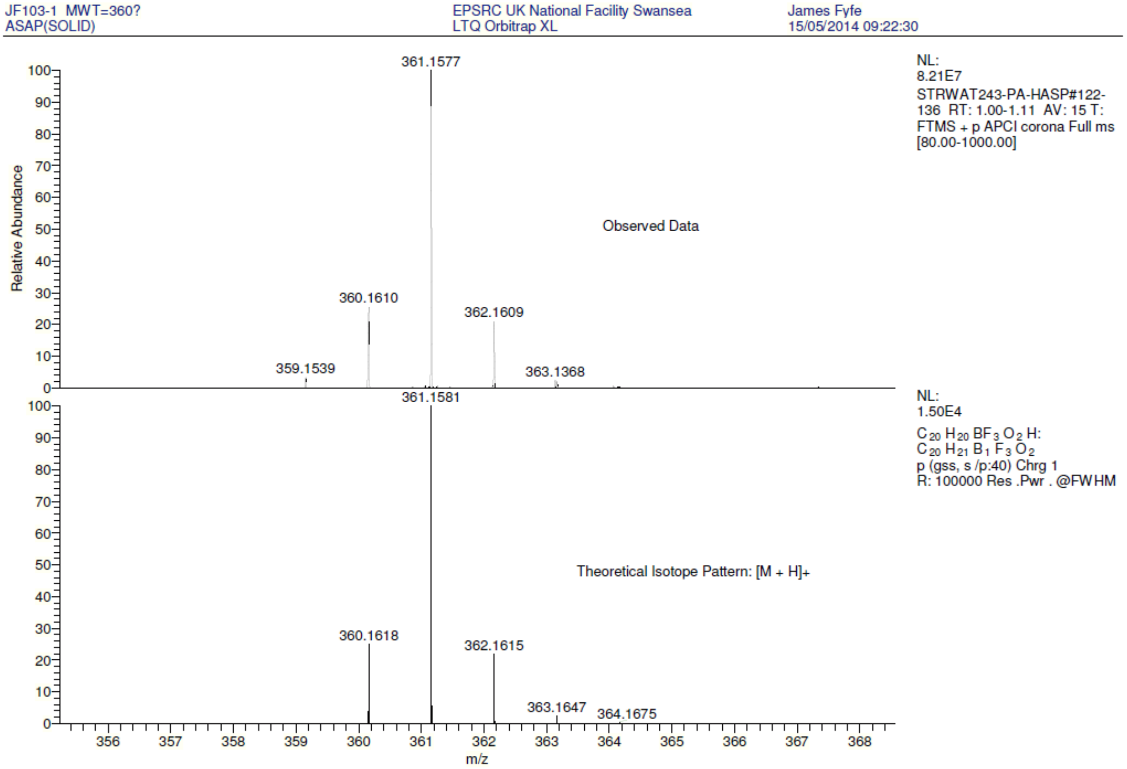

^{1}H NMR of 3x

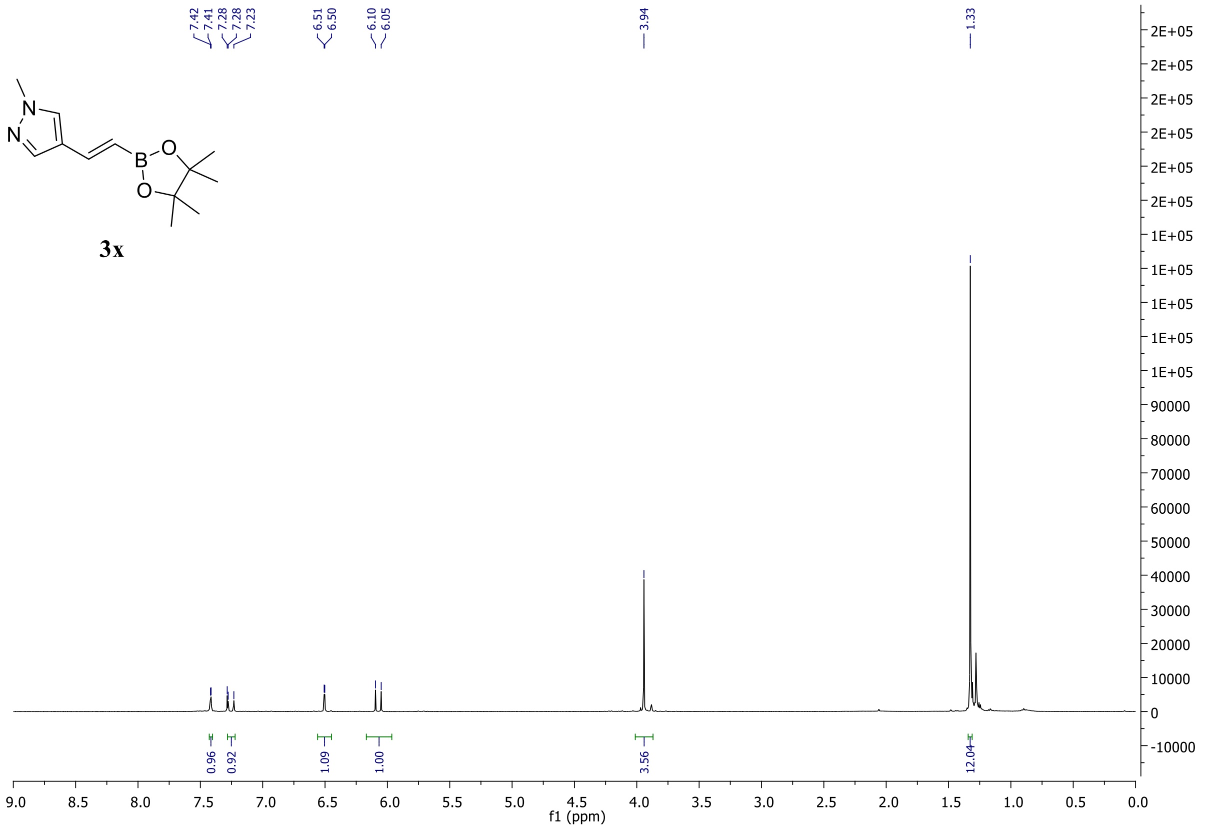

3x

^{13}C NMR of 3x

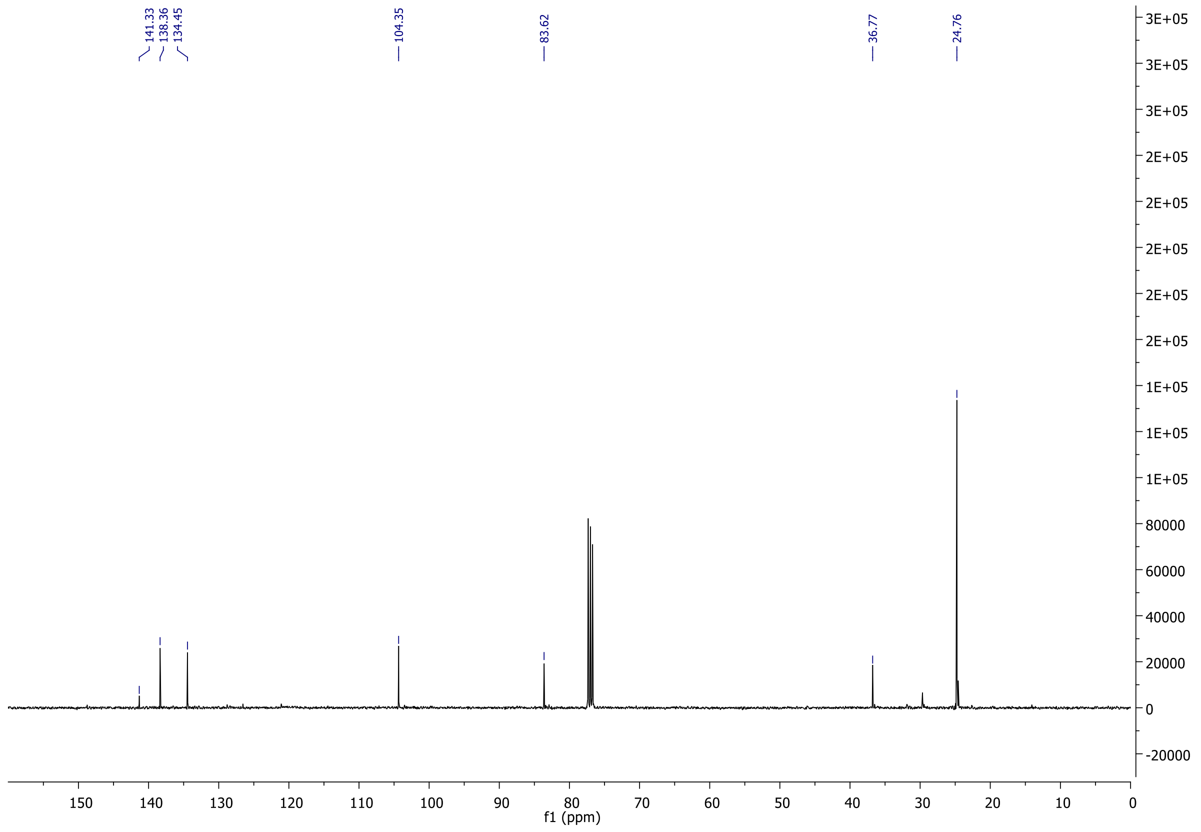

^{11}B NMR of 3x

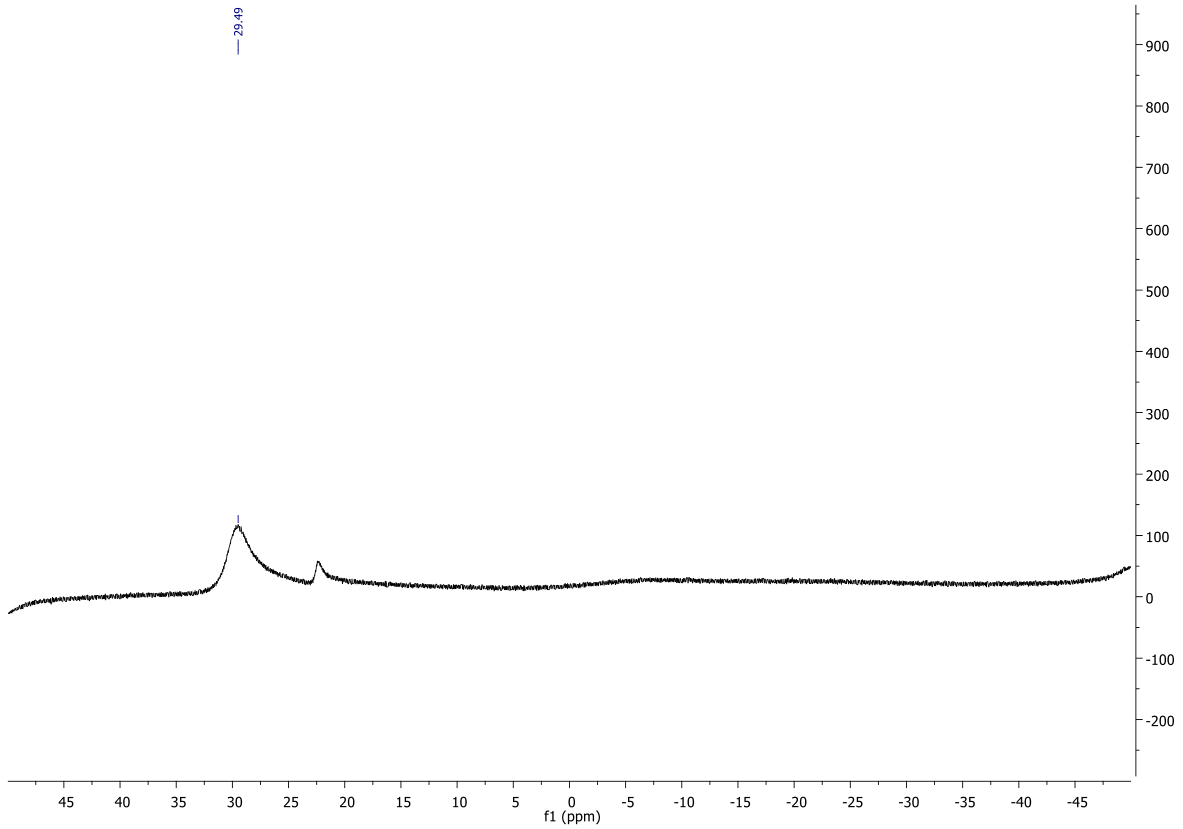

HRMS of 3x

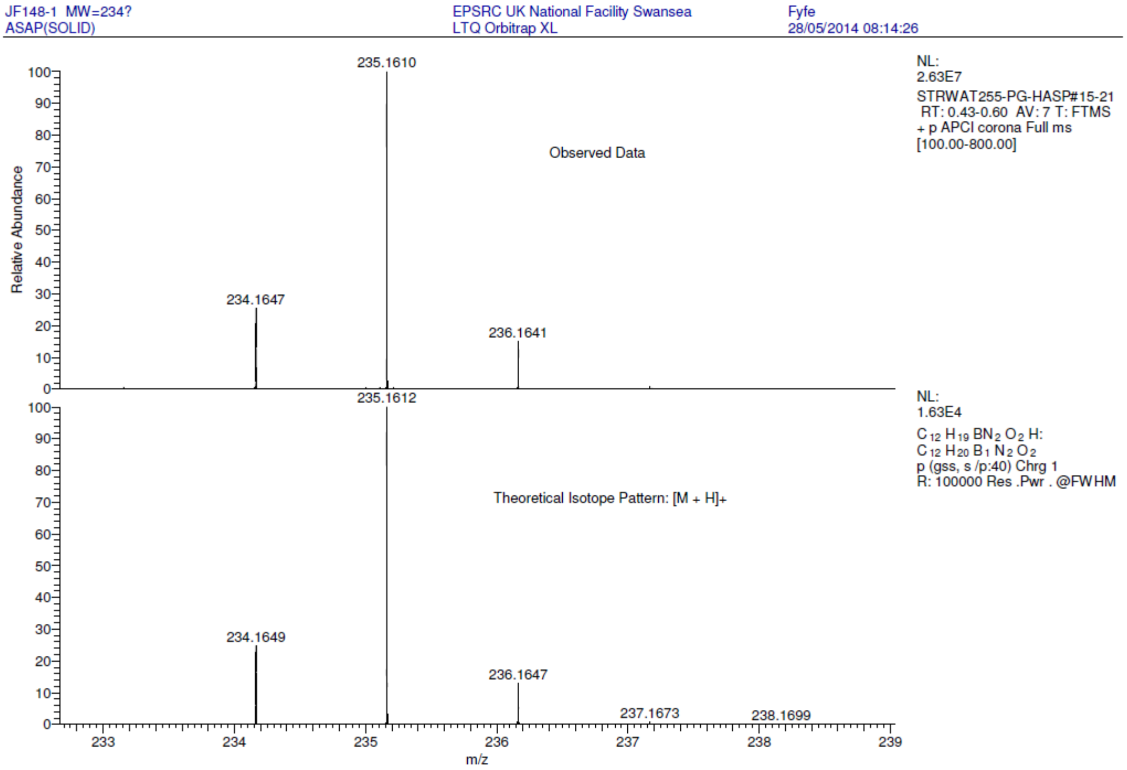

^{1}H NMR of 7a

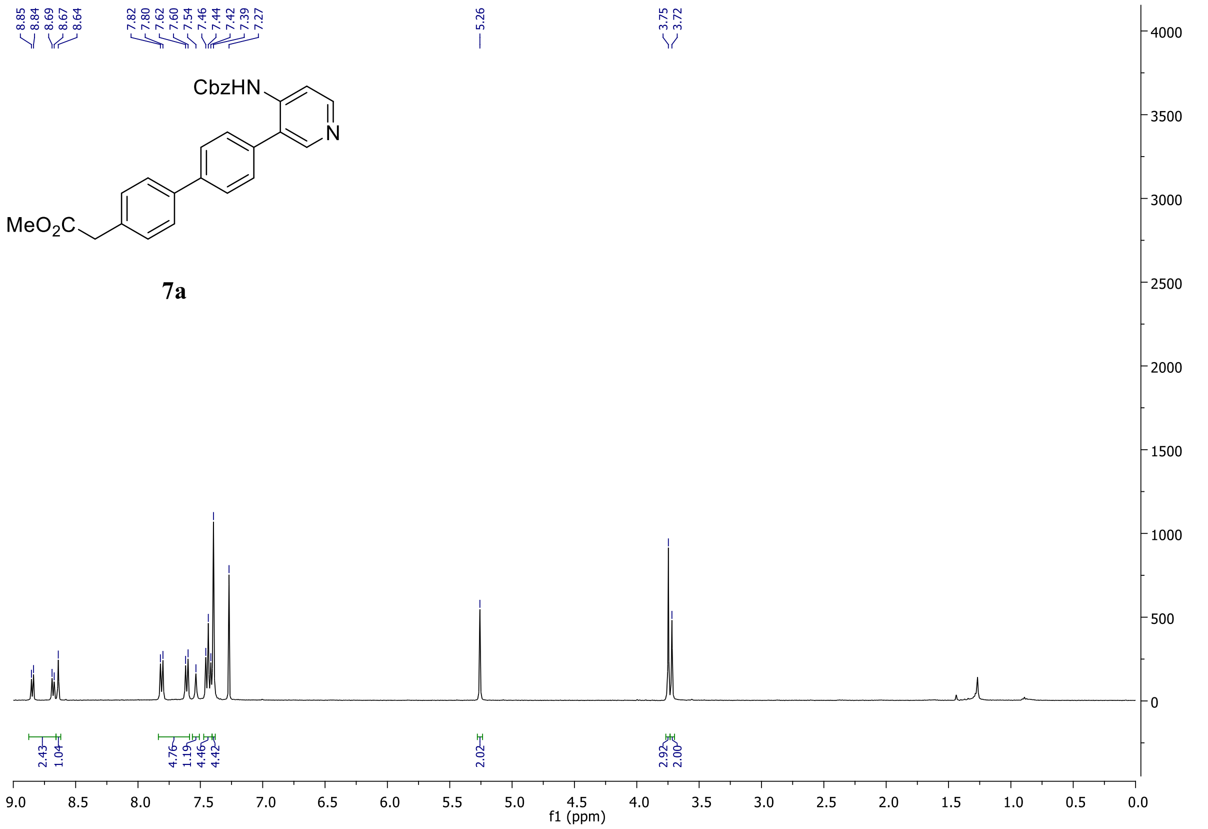

^{13}C NMR of 7a

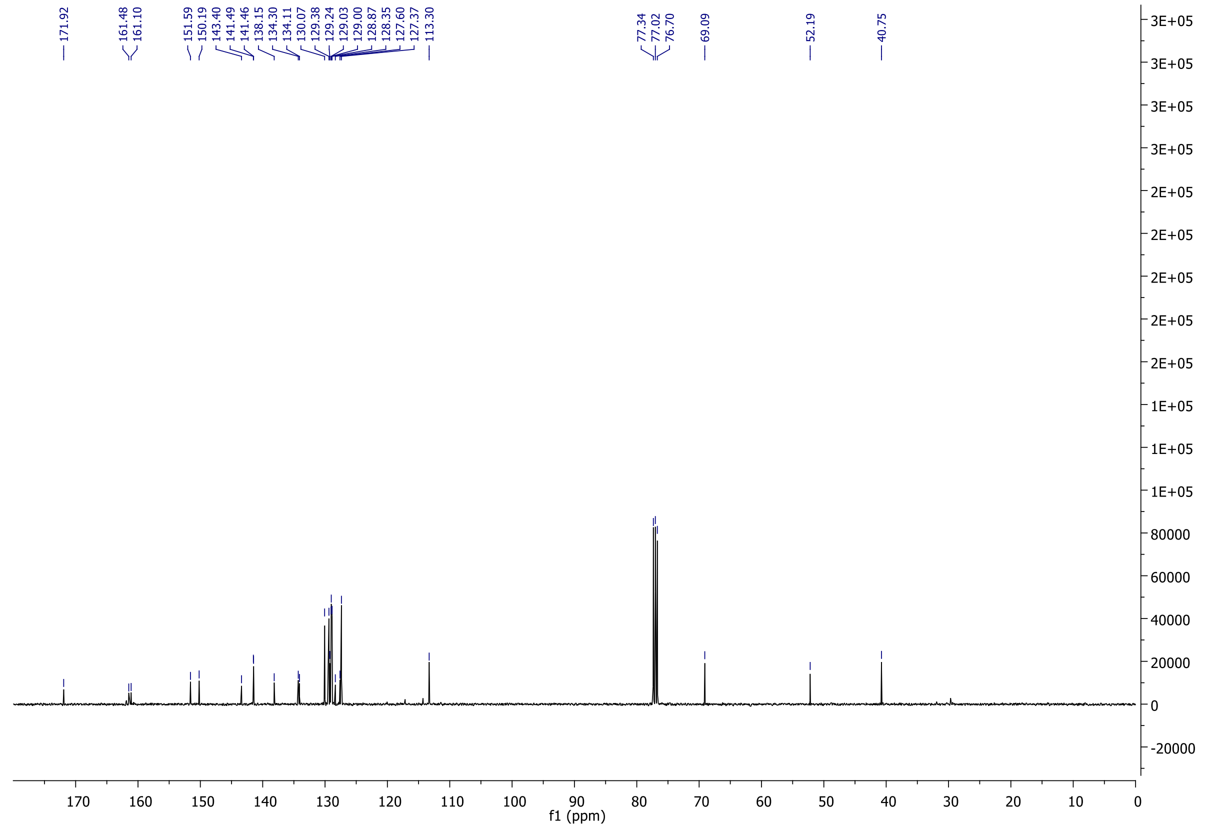

HRMS of 7a

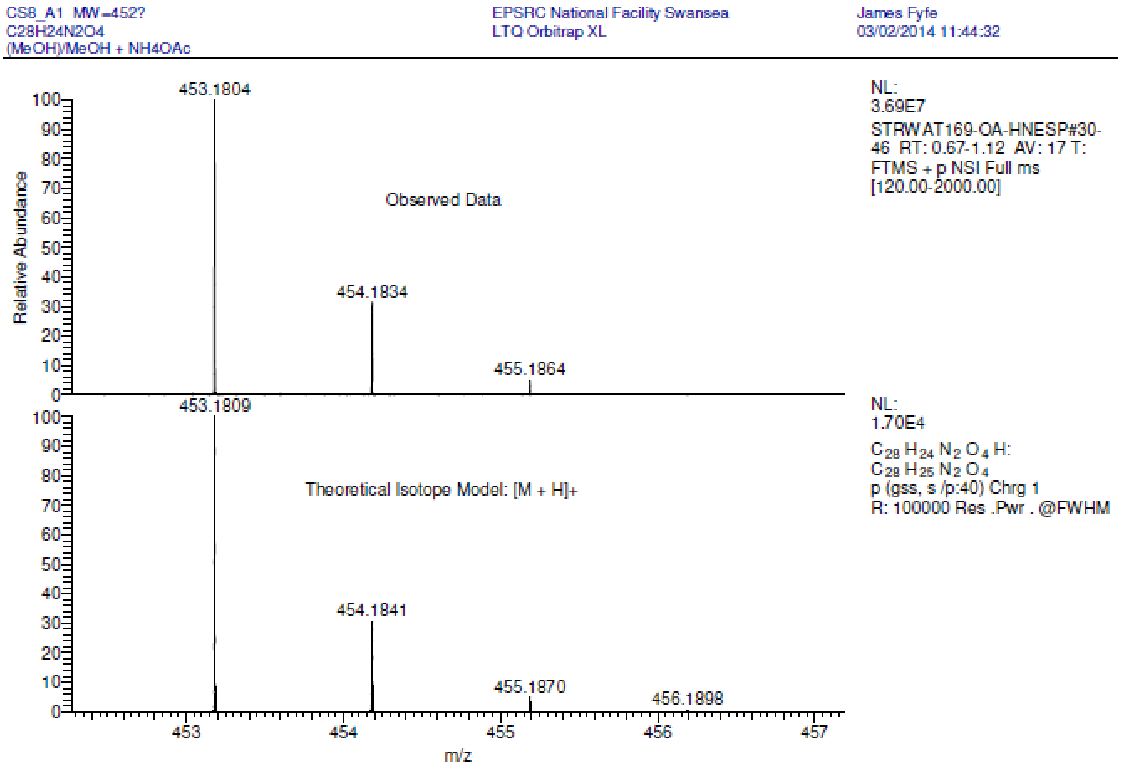

¹H NMR of 7b

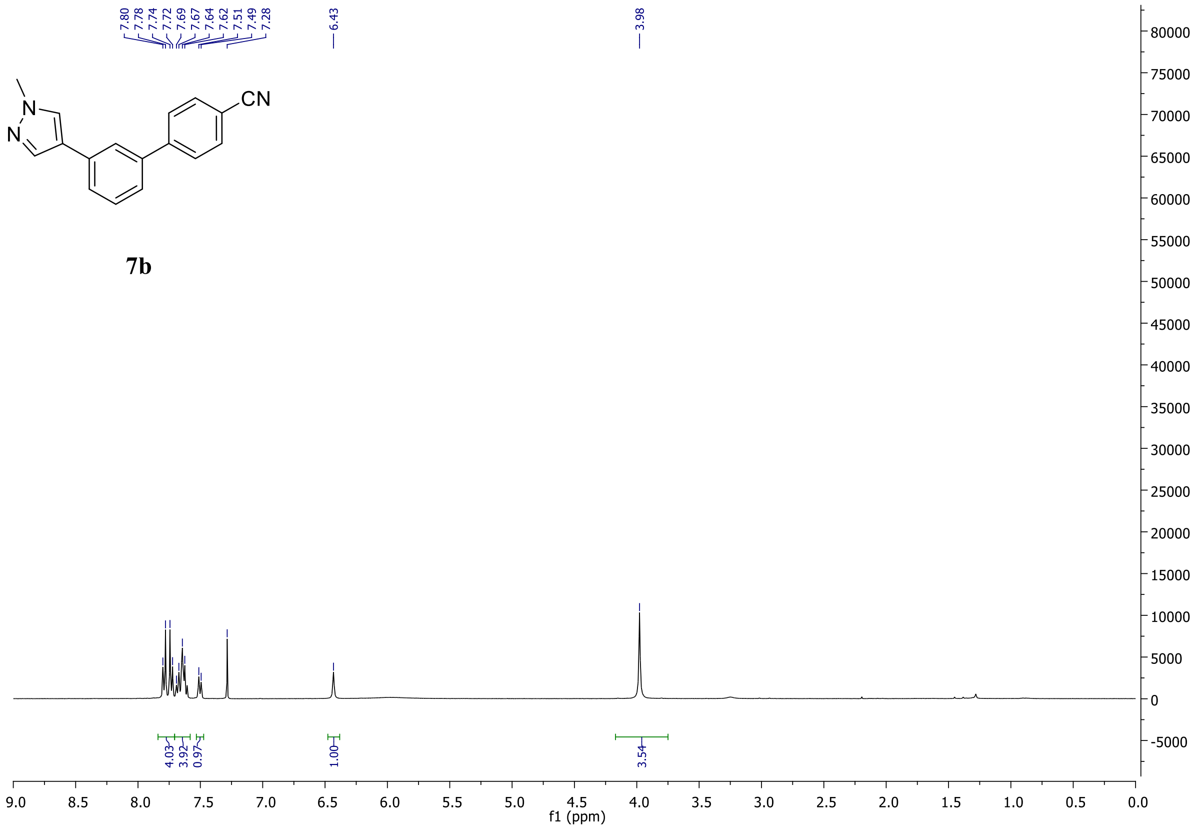

^{13}C NMR of 7b

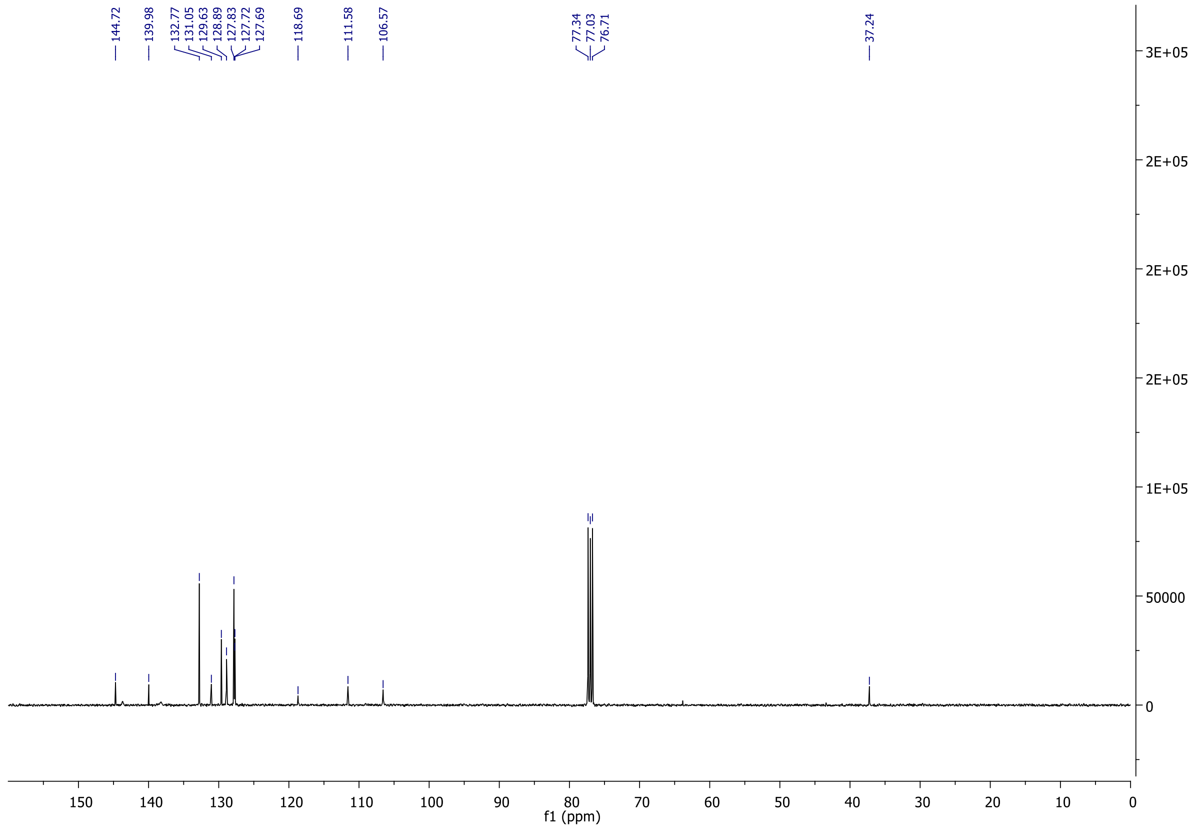

HRMS of 7b

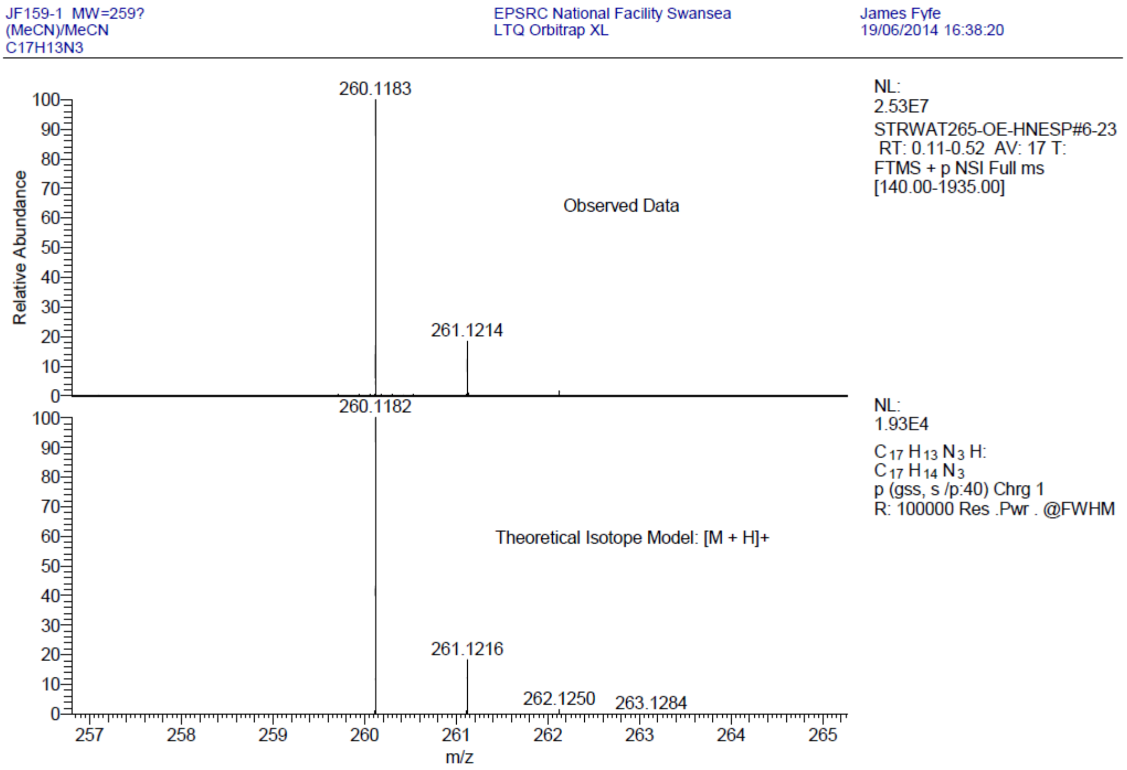

^{1}H NMR of 8

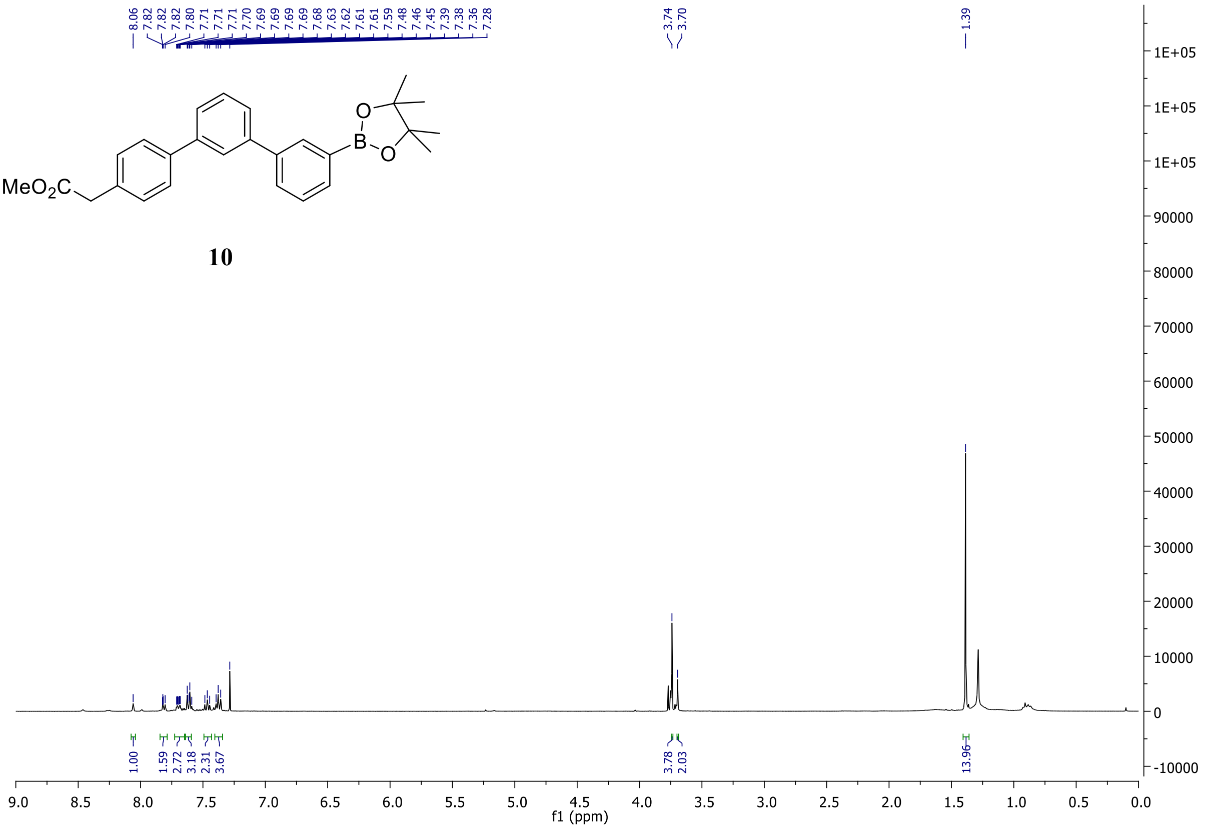

^{13}C NMR of 8

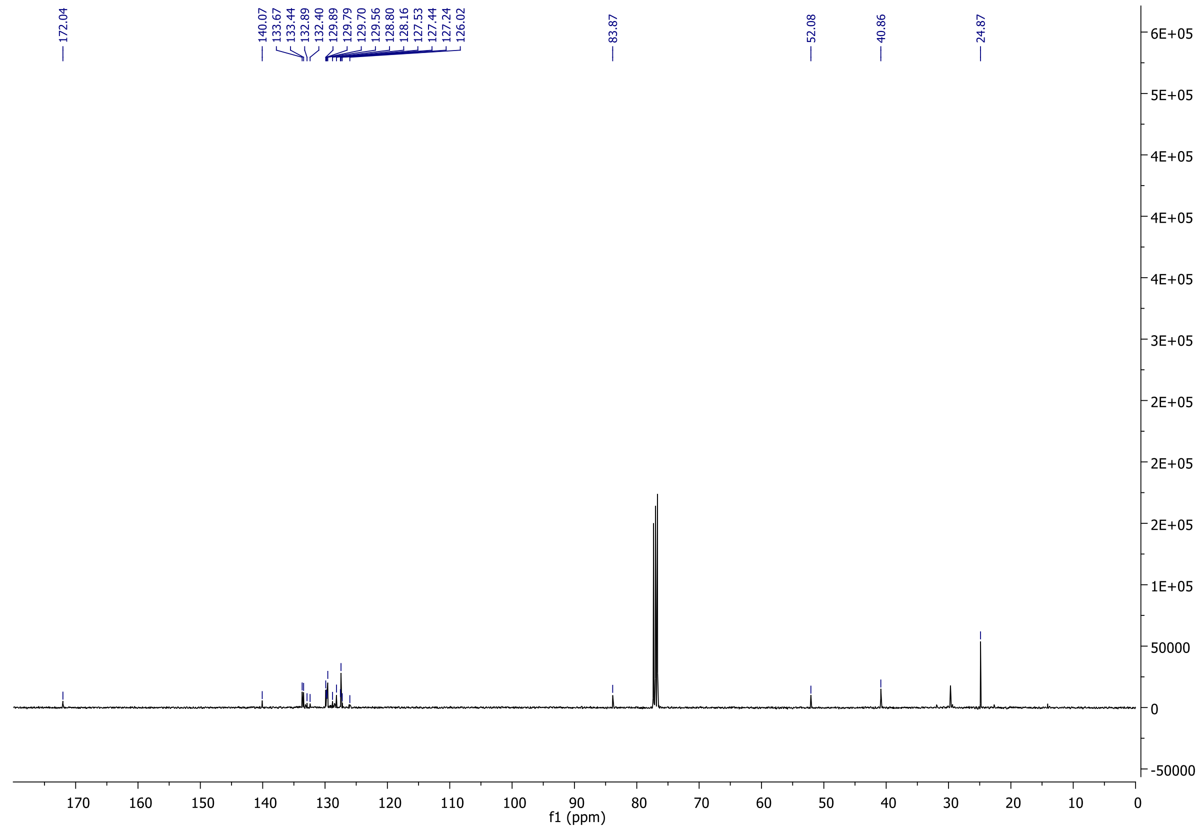

^{11}B NMR of 8

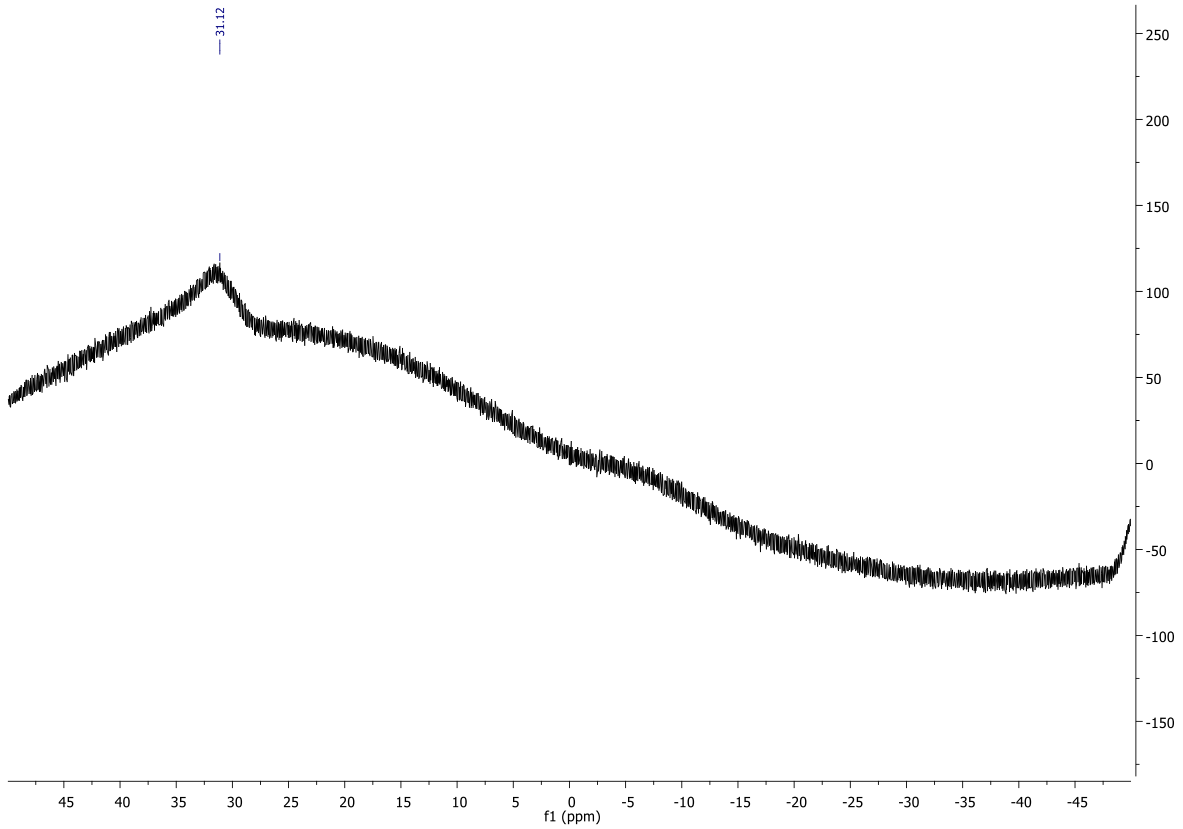

HRMS of 8

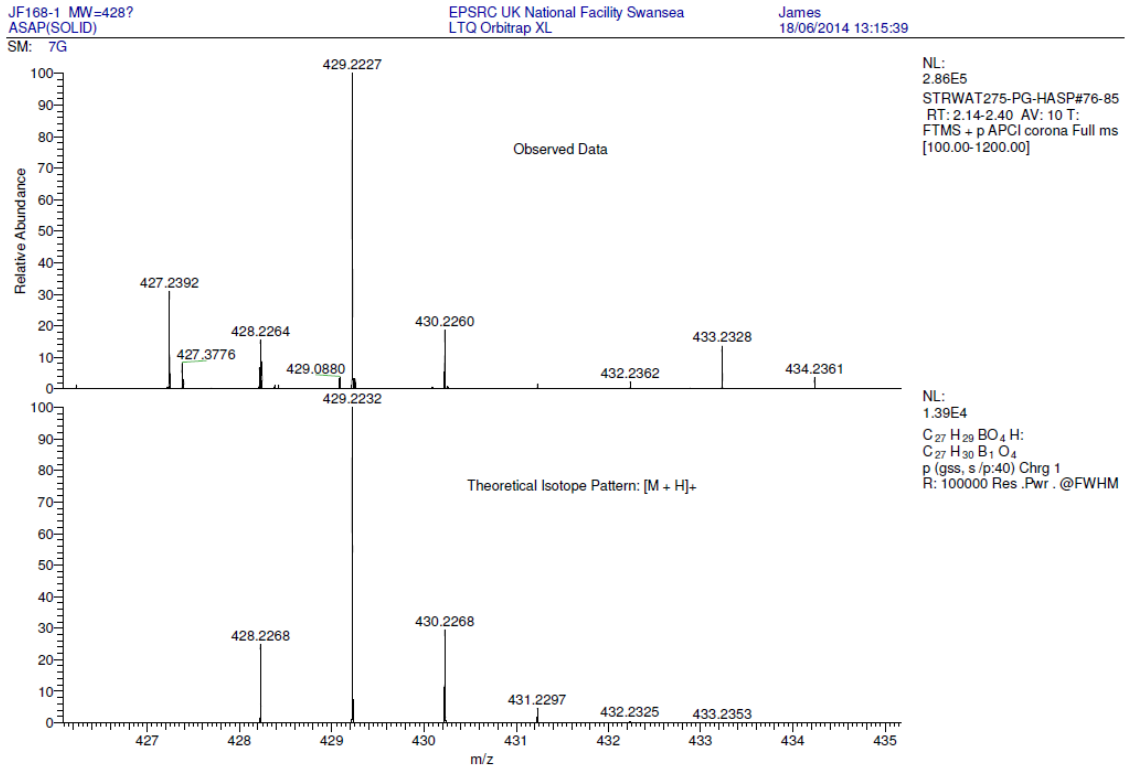

^{1}H NMR of 9

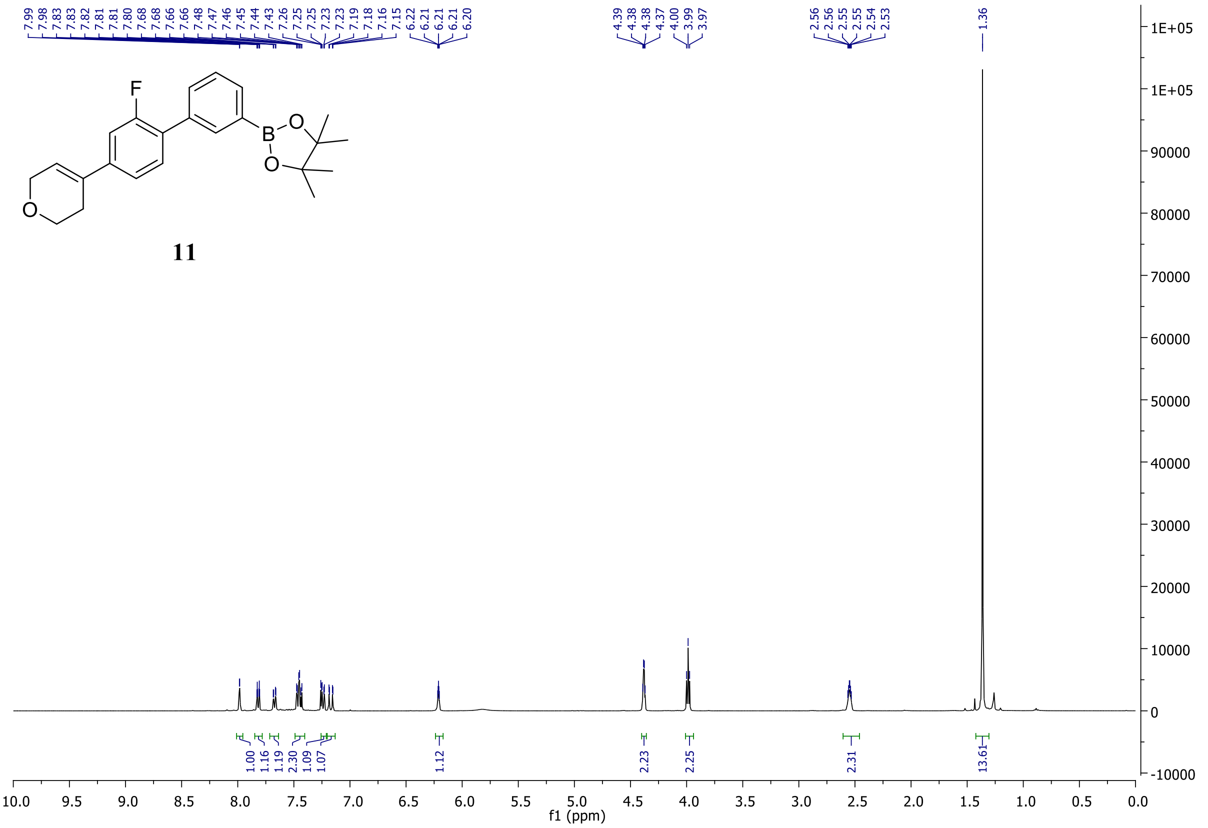

^{13}C NMR of 9

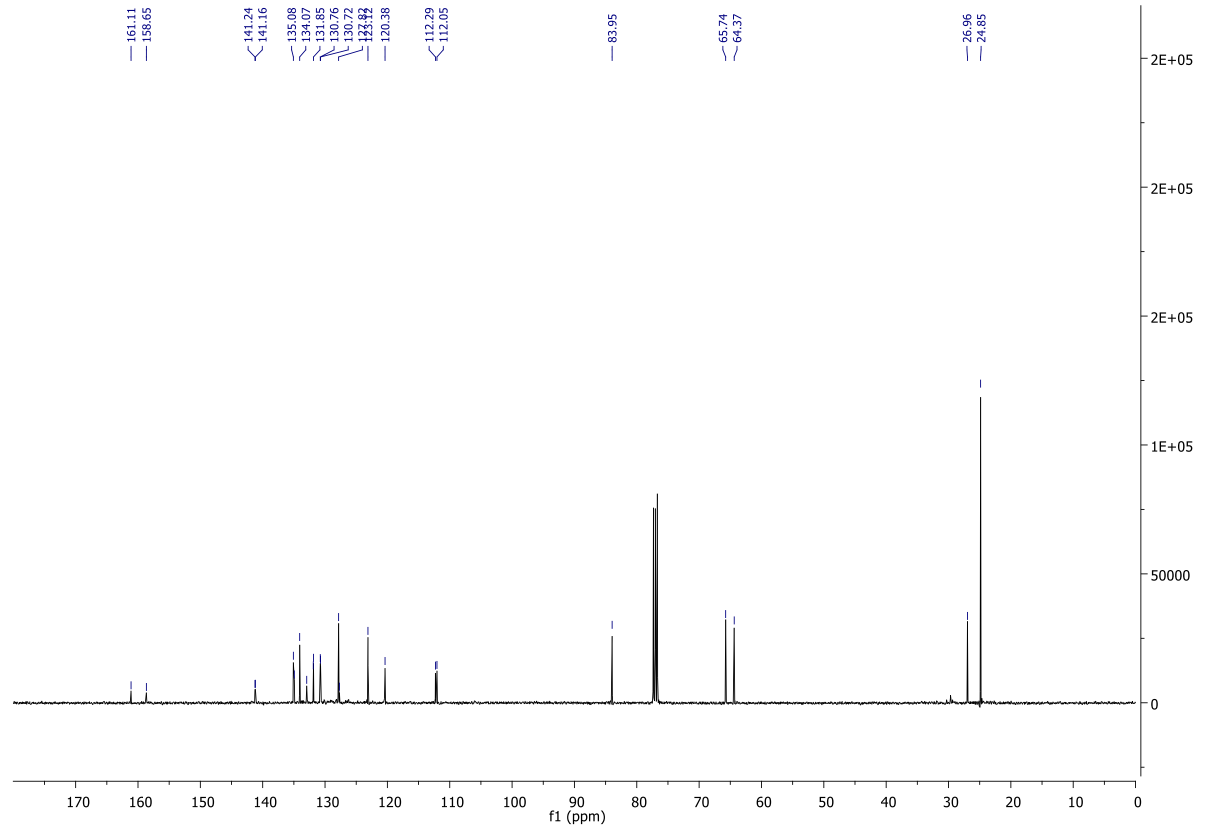

^{11}B NMR of 9

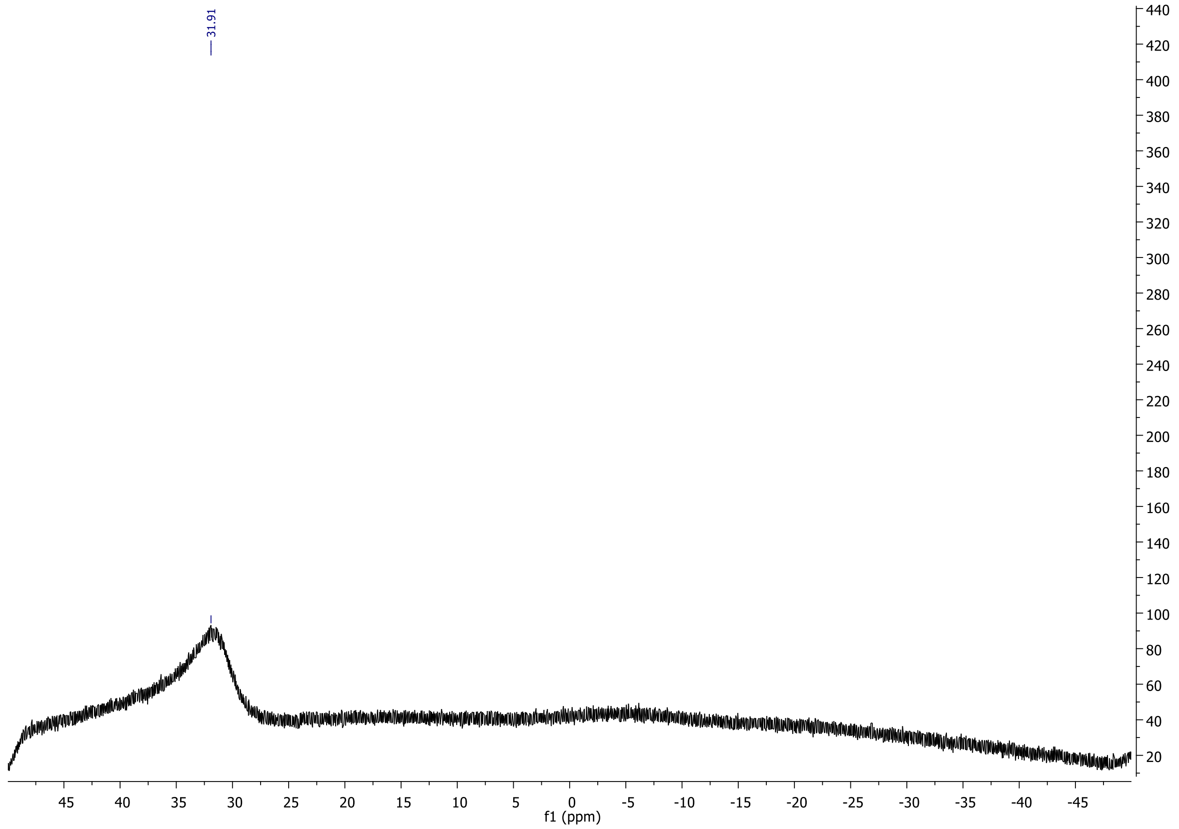

^{19}F NMR of 9

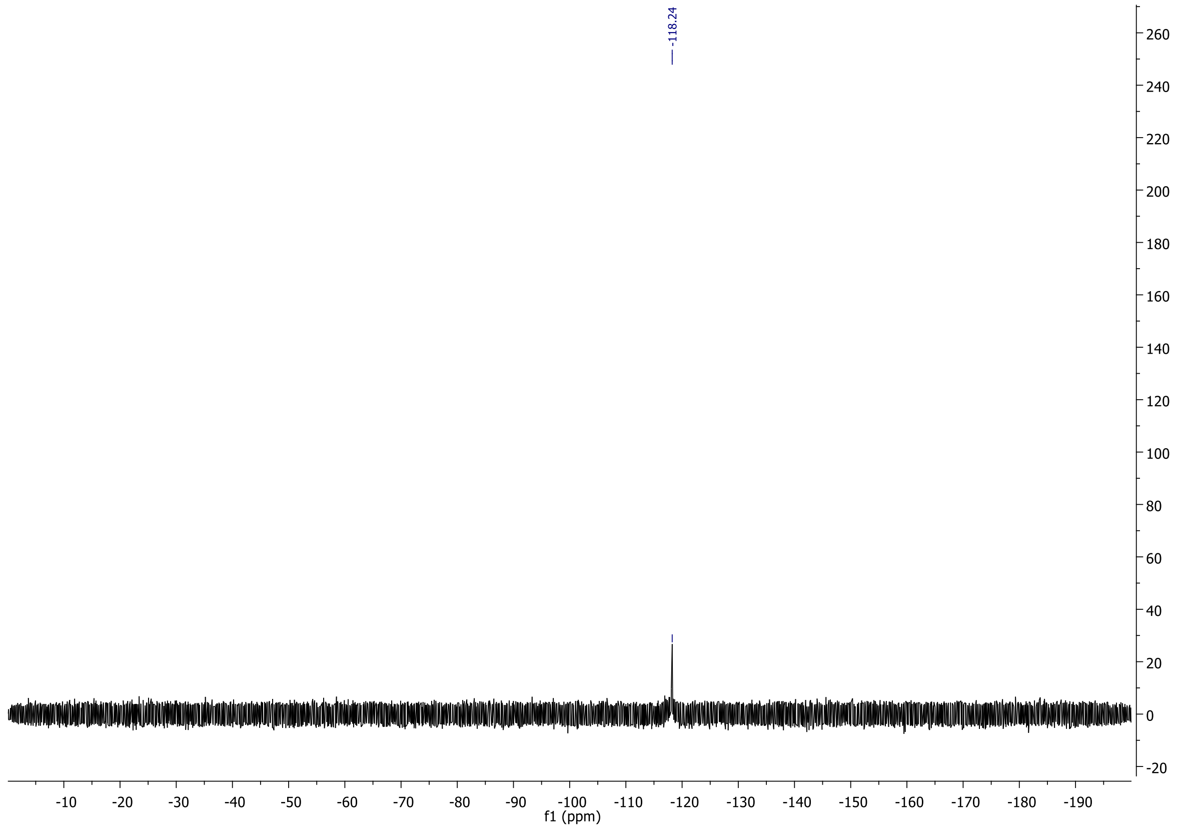

HRMS of 9

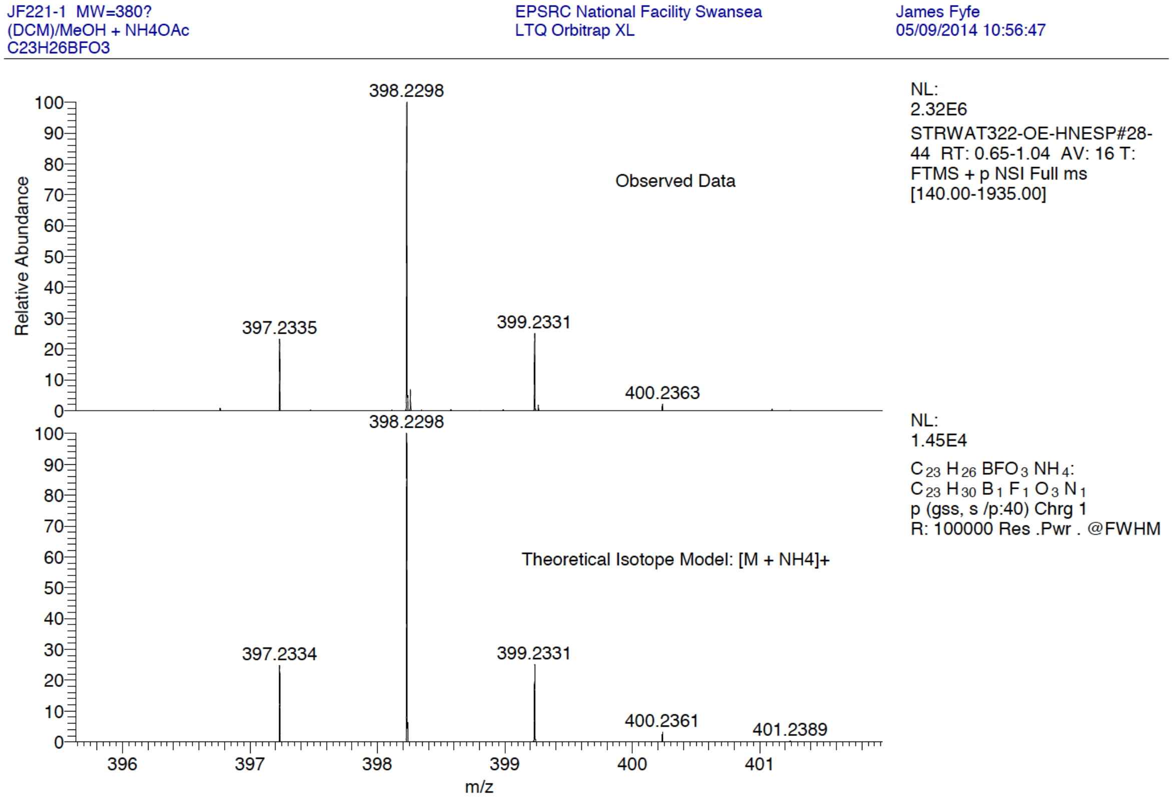
JF221-1 MW=380?
(DCM)/MeOH + NH4OAc
C23H26BFO3
EPSRC National Facility Swansea
LTQ Orbitrap XL
James Fyfe
05/09/2014 10:56:47
NL:
2.32E6
STRWAT322-OE-HNESP#28-44 RT: 0.65-1.04 AV: 16 T: FTMS + p NSI Full ms [140.00-1935.00]
Observed Data
398.2298
397.2335
399.2331
400.2363
NL:
1.45E4
C23 H26 BFO3 NH4:
C23 H30 B1 F1 O3 N1
p (gss, s /p:40) Chrg 1
R: 100000 Res .Pwr . @FWHM
Theoretical Isotope Model: [M + NH4]+
398.2298
397.2334
399.2331
400.2361
401.2389
Relative Abundance
m/z
